# Supplementary material for: Drugit: crowd-sourcing molecular design of non-peptidic VHL binders
Source: Nat Commun. 2025 Apr 14;16:3548. doi: 10.1038/s41467-025-58406-0 (PMC11997059; doi:10.1038/s41467-025-58406-0)
Supplement: Supplementary file 1 — Supplementary Information [file 41467_2025_58406_MOESM1_ESM.pdf]

Supplementary Information

Drugit: Crowd-sourcing molecular design of non-peptidic VHL binders

Supplementary Figures

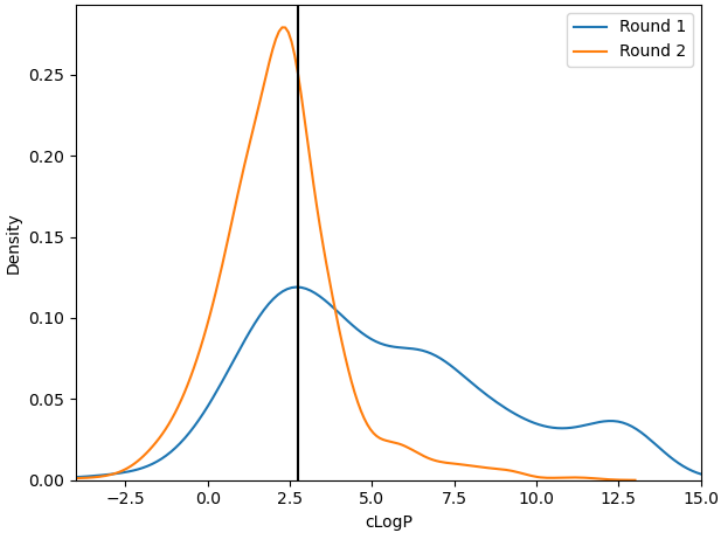

Supplementary Figure 1: Effect of adding penalties for cLogP. Round 1 contained no explicit penalties for cLogP, resulting in many heavily hydrophobic compounds (distribution in blue). Round 2 introduced a penalty for compounds which had cLogP values above 2.75 (black line). The distribution of compounds for the second round including the cLogP penalty matches the desired range much more closely (orange distribution). Source data are provided as a Source Data file.

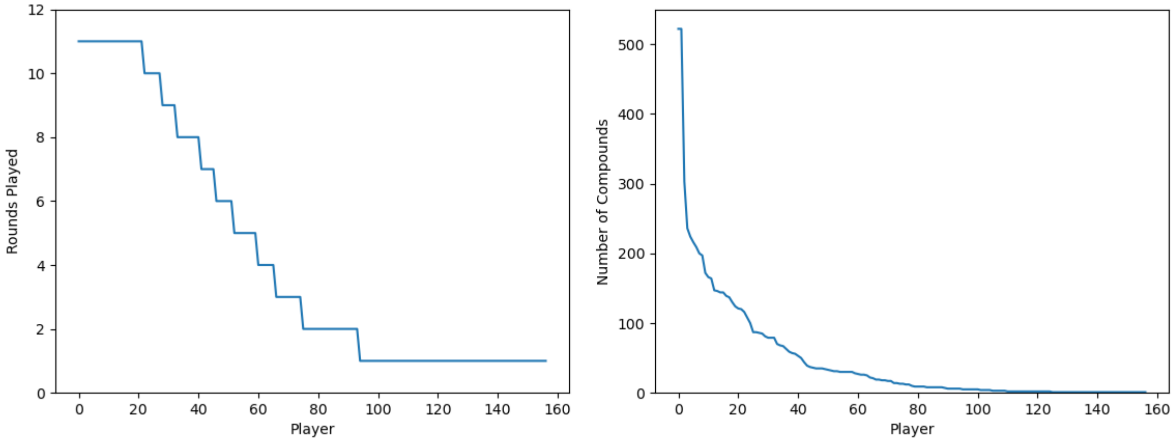

Supplementary Figure 2: Distribution of player participation. Left: The number of rounds in which each player submitted a new molecular structure. The maximum value is 11, due to round 5 being repeated for technical difficulties. Right: The distribution of the total number of novel compounds submitted by each player across all rounds. The Foldit client regularly sends snapshots of the game state to the server, resulting in multiple compounds per round. Source data are provided as a Source Data file.

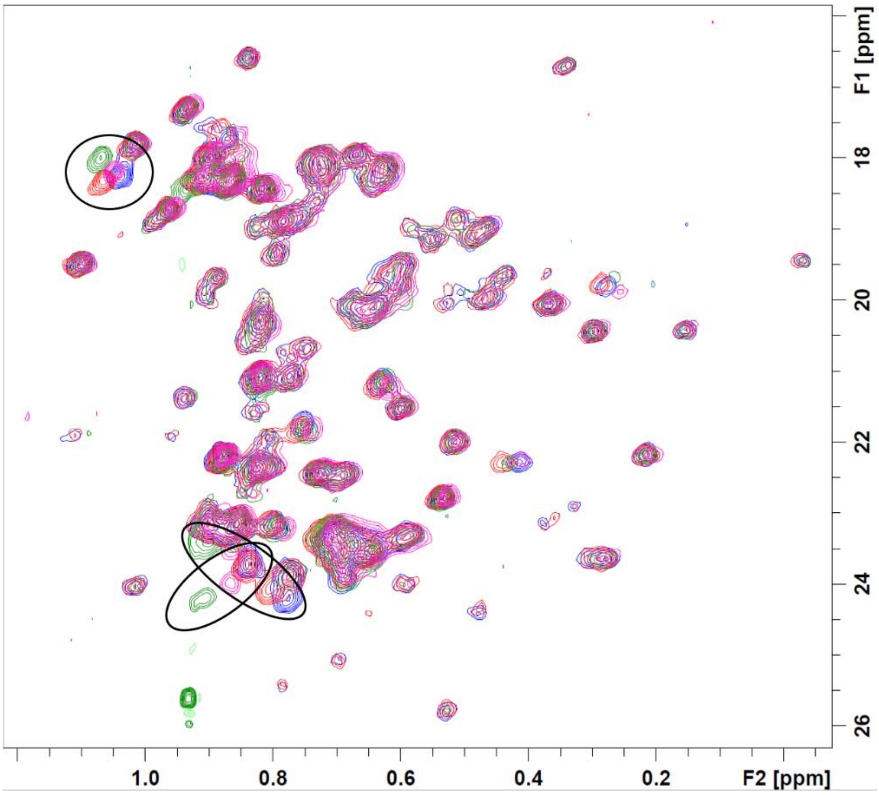

Supplementary Figure 3: Val and Leu region of ^{13}C labeled VCB protein at 200 µM. Apo protein shown in blue, + 500 µM of VH298 shown in green + 500 µM of reference molecule 2 shown in red, and +1000 µM of **1** shown in magenta. The induced CSPs of the three compounds is the same, indicating that they interact with the same binding region.

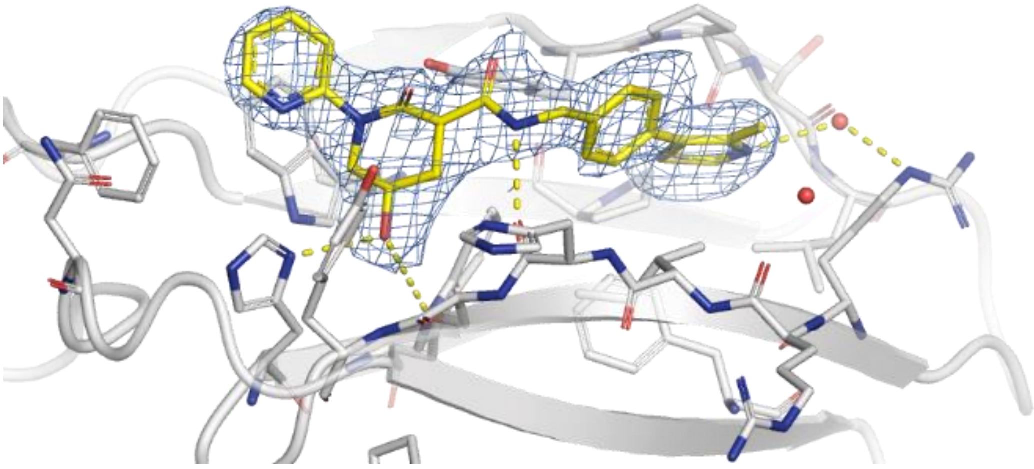

Supplementary Figure 4a: 2FoFc electron density map at 1σ of compound **1** as observed in the co-crystal structure with VHL (PDB code: 8P0F).

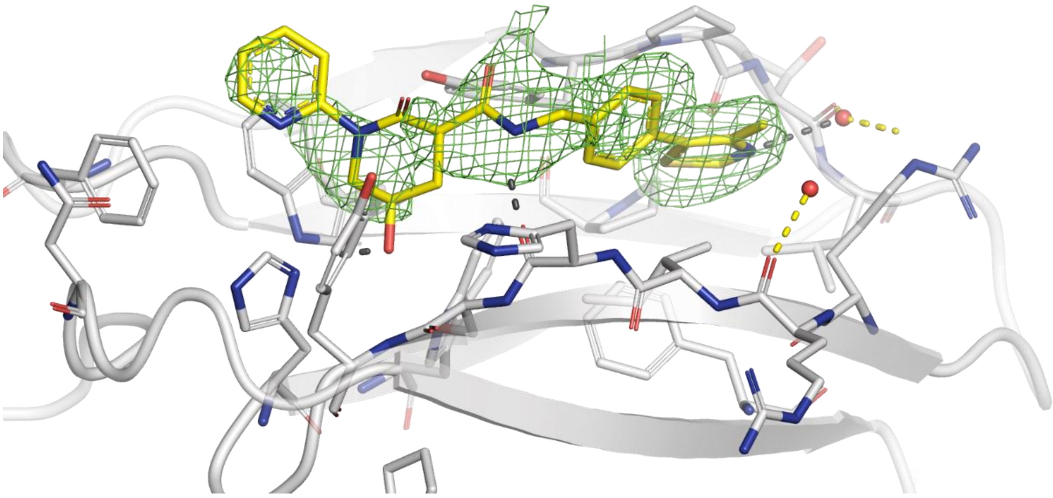

Supplementary Figure 4b: Omit map at 3σ of compound **1** as observed in the co-crystal structure with VHL (PDB code: 8P0F).

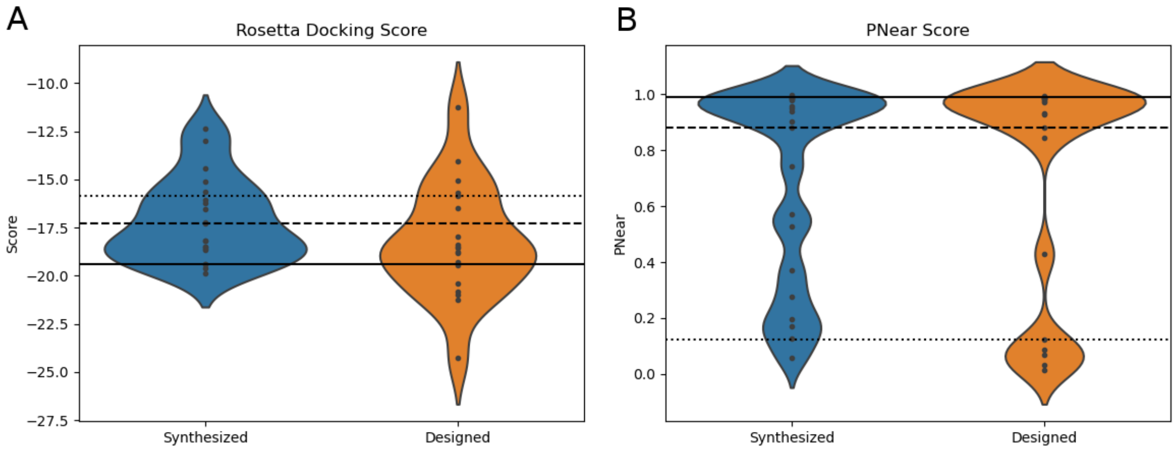

Supplementary Figure 5: RosettaLigand redocking of tested and corresponding parent compounds. A) The distribution of the lowest interface energies found for the compounds as-synthesized (blue) and as-designed (orange). The values for the parent reference molecule 1 (solid line), the design-matching diastereomer of compound **1** (dashed lines) and the player design (dotted line) are indicated. B) PNear[1] metrics of RosettaLigand docking funnels, using the from-player orientation as the 'native'/reference structure for rmsd. PNear measures the quality of the 'docking funnel' (how close low-energy structures are to the reference), ranging from 0.0 (poor quality) to 1.0 (high quality). Source data are provided as a Source Data file.

Supplementary Tables

Supplementary Table 1. Summary of conditions for VHL puzzle rounds

	Starting[a]	Waters[b]	Ligand weight[c]	H-bonds[d]	TPSA		H-bond donors		cLogP		Rot. bonds		Penalty	
					Value	Penalty	Value	Penalty	Value	Penalty	Value	Penalty	Amide[e]	Ring[f]
1	full	no	6	none	<139	1	<4	10	none	none	<11	5	0.25	none
2	full	no	6	none	<139	2	<3	20	<2.75	10	<11	5	0.5	yes-
3	minimal	no	6	1	<139	1.5	<3	15	<2.75	8	<10	5	0.5	none
4	no center	no	6	2	<139	1.5	<3	15	<2.75	8	<10	5	none	yes
5	full	yes	6	6	<139	1.5	<3	15	<2.75	8	<10	5	none	yes
6	full	yes	6	6	<125	1.5	<3	15	<4	6	<9	7.5	none	yes
7	full	yes	6	6	<110	1.5	<3	15	<5	6	<9	7.5	none	yes
8	full	yes	6	6	<100	1.5	<3	15	<5	6	<9	10	none	yes
9	full	yes	12	6	<100	1.5	<3	15	<5	6	<9	10	none	yes
10	full	yes	15	6	<100	2	<3	15	<5	6	<10	20	none	yes+

[a] Starting structure (See Table S2).
[b] Contains two crystallographic waters (PDB code: 5NVX).
[c] How much protein-ligand interactions and ligand internal energies are upweighted.
[d] How many of the existing protein-ligand (or protein-water) hydrogen bonds must be recapitulated to receive the full bonus.
[e] Per-group penalty for having an amide group in the designed structure.
[f] Penalties for having a hydroxyproline core: 'yes-': Having a five-membered aliphatic ring with one nitrogen and four carbons. 'yes': Having a five-membered aliphatic ring containing any number of carbon and nitrogen with an attached hydroxyl. 'yes+': Having any five-membered carbon/nitrogen ring with attached hydroxyl, or having a hydroxyl attached to two consecutive aliphatic atoms.

Supplementary Table 2. Starting structures for puzzle rounds

Designation	Structure
full (reference molecule 1)	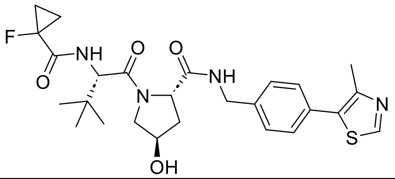
minimal	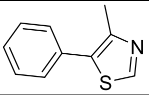
no center	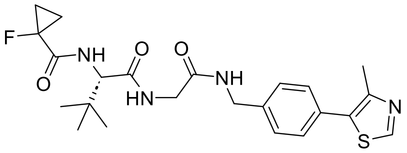

Supplementary Table 3: Player designed compounds and the corresponding synthesized compounds

ID[a]	Designer[b]	Round	Designed Structure	#[c]	Synthesized Structure
404627247	Nicm25	6	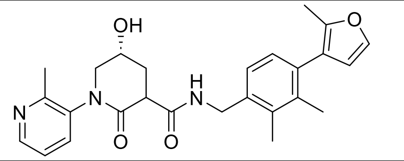	1	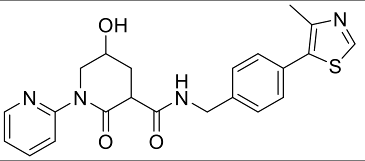
404626702	cas0362[d]	6	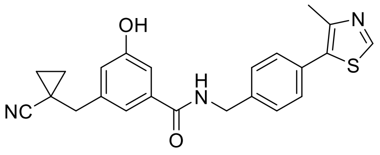	2	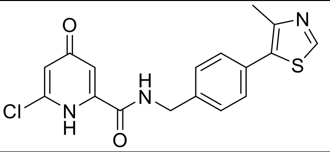
				3	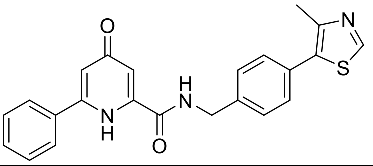
				4	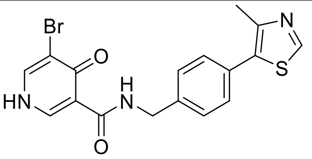
				5	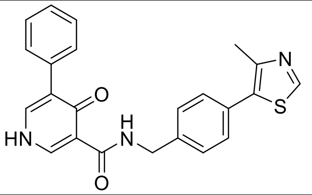
404616583	cas0362[d]	6	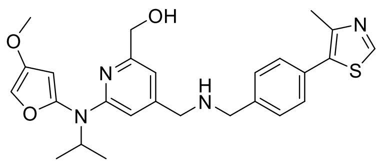	6	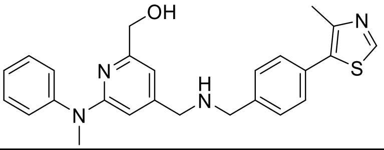
				7	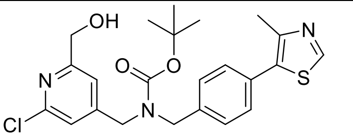
404478307	Bruno Kestemont	4	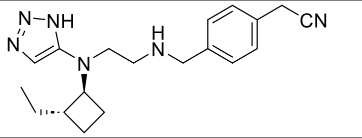	8	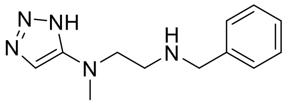
404608790	cas0362[d]	6	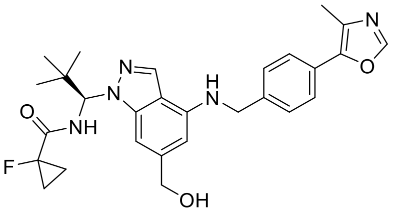	9	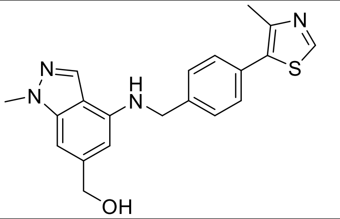

ID[a]	Designer[b]	Round	Designed Structure	#[c]	Synthesized Structure
				10	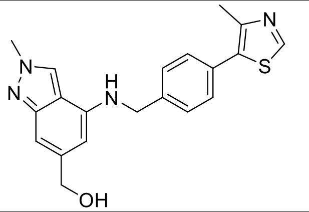
				11	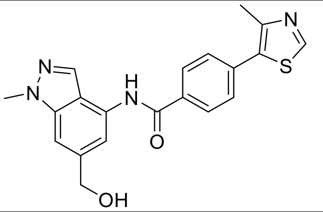
				12	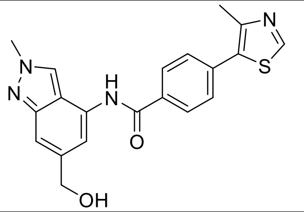
404625599	cas0362[d]	6	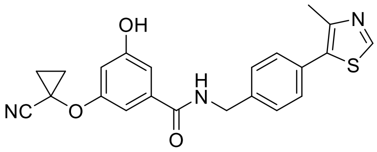	13	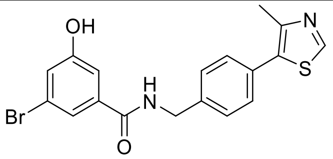
				14	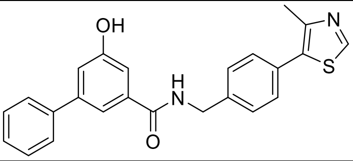
404377707	Bruno Kestemont	3	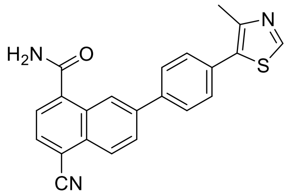	15	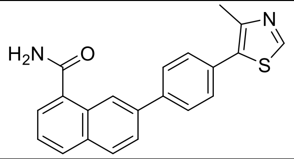
				16	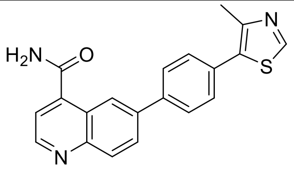
404663868	equilibria	7	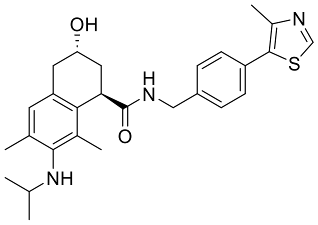	17	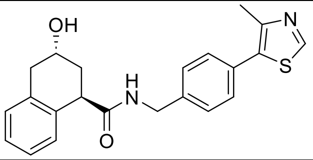
				18	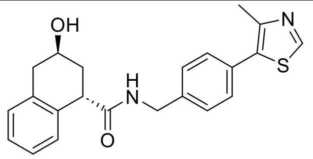
404704247	Nicm25	8		19	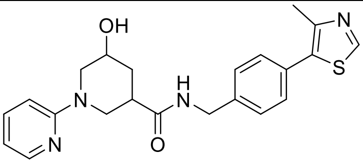

ID[a]	Designer[b]	Round	Designed Structure	#[c]	Synthesized Structure
			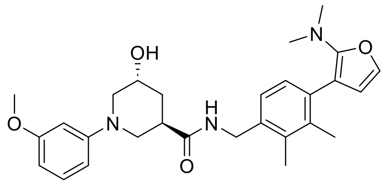	20	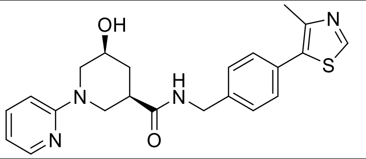
				21	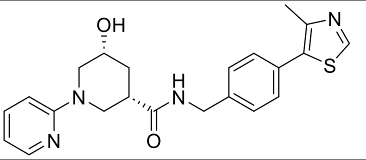
404520999	Borets	5	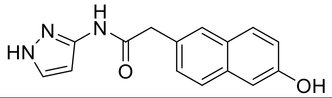	22	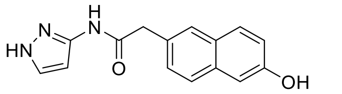
404807783	Bruno Kestemont	10	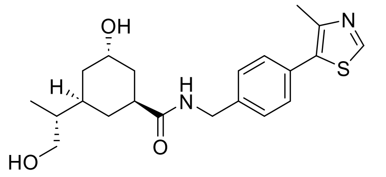	23	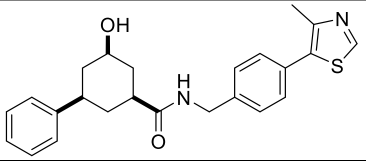
				24	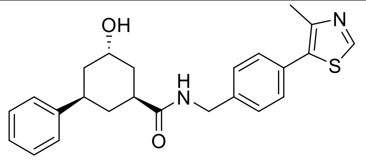
				25	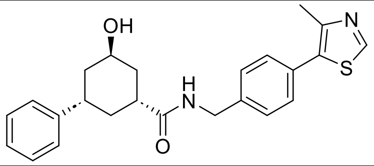
404677401	pr0tfold	7	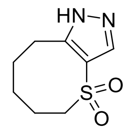	26	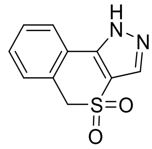
				27	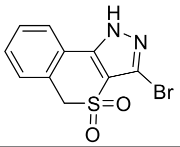
				28	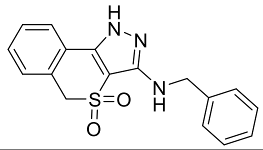
				29	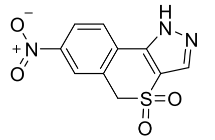
404836689	pr0tfold	10		30	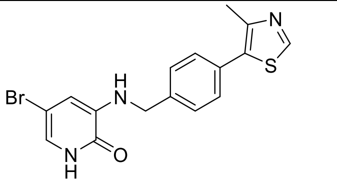

ID[a]	Designer[b]	Round	Designed Structure	#[c]	Synthesized Structure
			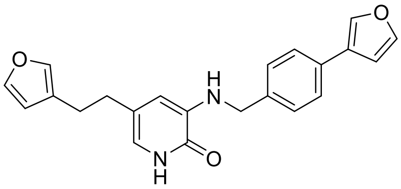	31	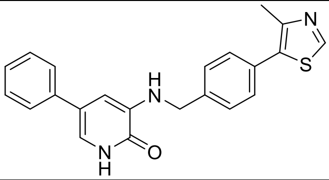
				32	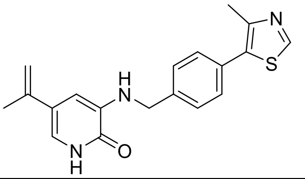
				33	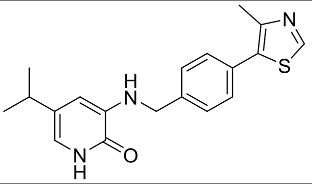
404626730	cas0362[d]	6	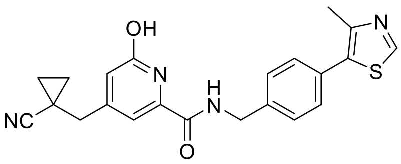	34	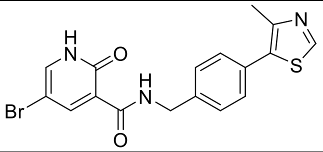
				35	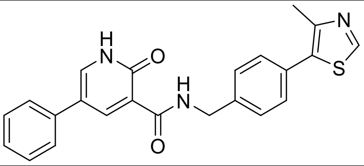
404513771	Borets	5	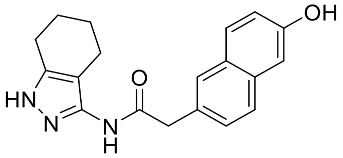	36	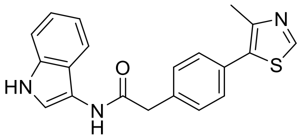
404732188	fiendish ghoul	8	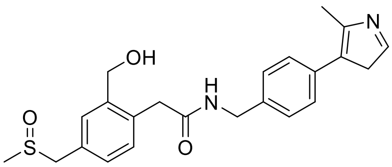	37	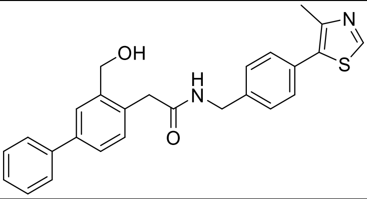
404610051	cas0362[d]	6	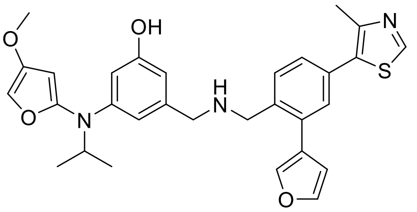	38	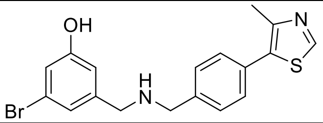
				39	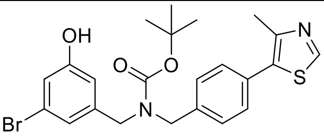
				40	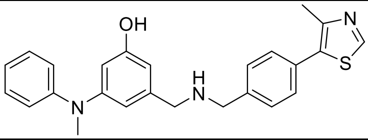
404626730	cas0362[d]	6		41	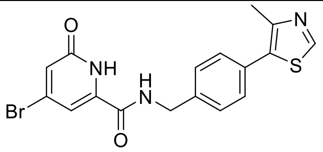

ID[a]	Designer[b]	Round	Designed Structure	#[c]	Synthesized Structure
			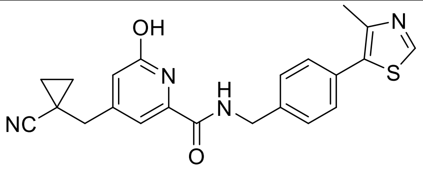	42	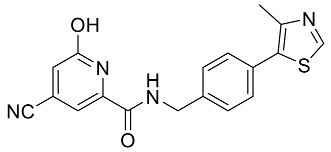
404390726	Anon.[b]	3	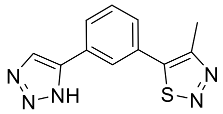	43	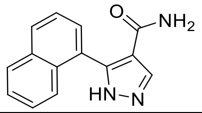

[a] Identifier in the corresponding SDF file.

[b] Username of player designed compound. One player wished to remain anonymous.

[c] Compound number for synthesized structure.

[d] Player cas0362 was involved in selecting (designer-blinded) compounds for synthesis.

Supplementary Table 4: Player-designed compounds and related, synthetically more accessible compounds for which ΔG values were predicted by FEP+.

ID	Designed Structure	ΔG(pred) [kcal/mol]	Structure used in FEP+
404627247	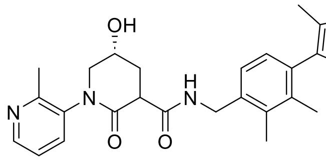	-10.1	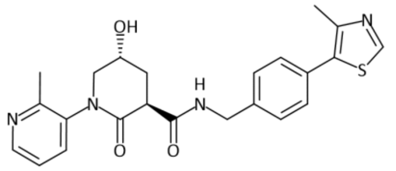
404626702	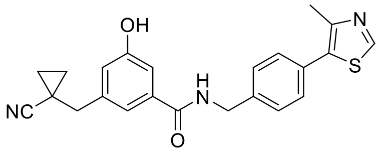	-8.5	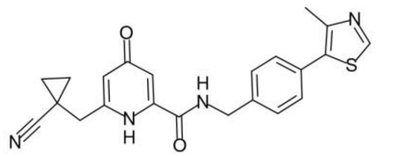
404616583	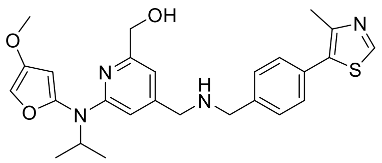	-2.6	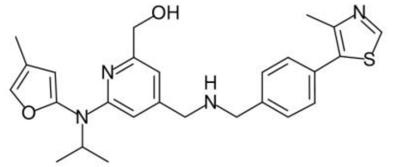
404478307	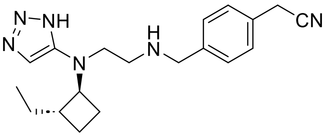	-0.1	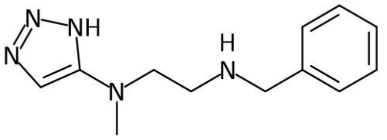
404608790	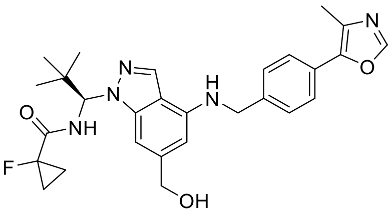	-5.9	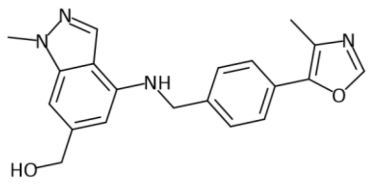
404625599	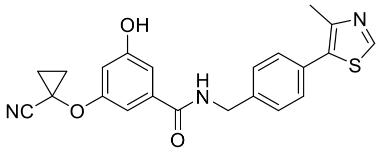	-6.6	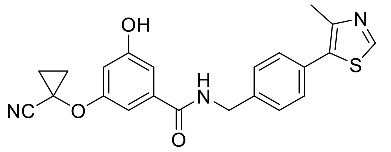
404377707	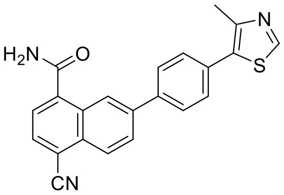	-10.0	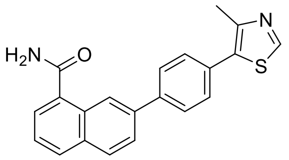
404663868	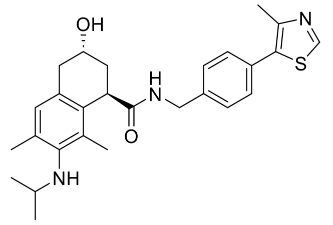	-8.6	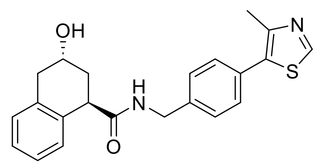

ID	Designed Structure	ΔG(pred) [kcal/mol]	Structure used in FEP+
404704247	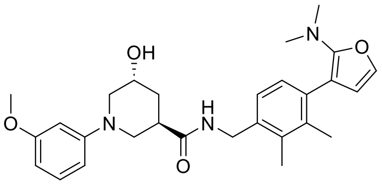	-8.9	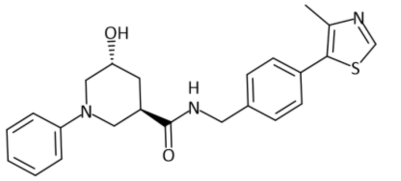
404807783	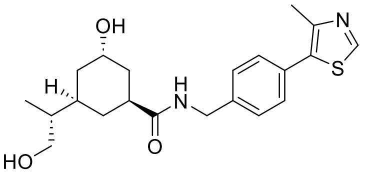	-11.6	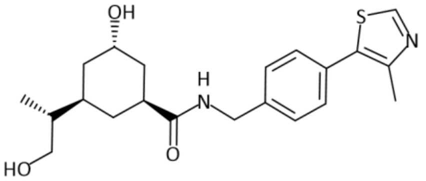
404677401	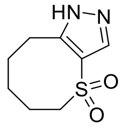	-3.2	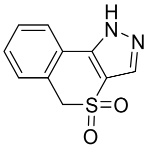
404836689	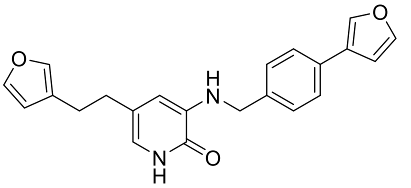	-8.1	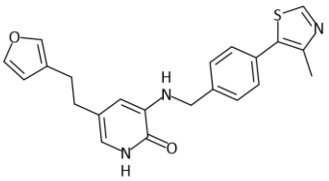
404626730	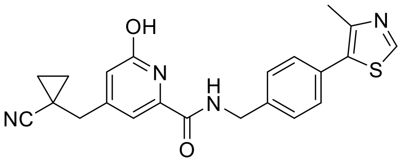	-5.5	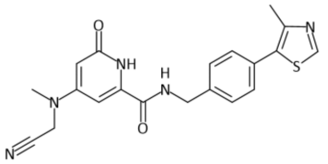
404513771	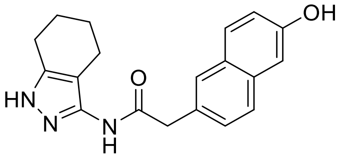	-6.6	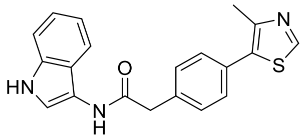
404732188	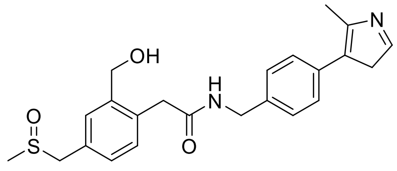	-7.3	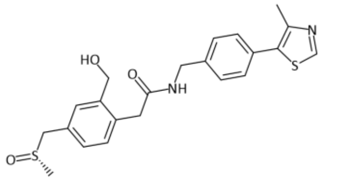
404610051	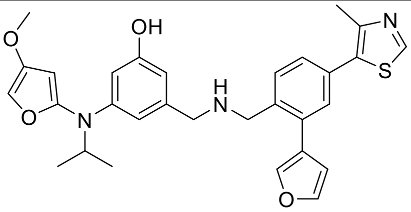	-3.8	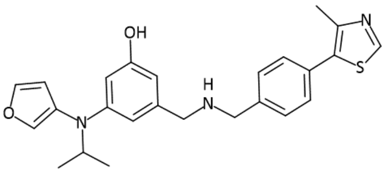
404390726	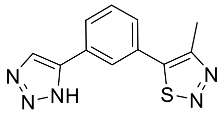	-4.1	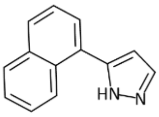

Supplementary Table 5: Synthesized compounds and calculated properties

#	Structure	Molecular weight	TPSA	HBD count	cLogP	rot. bonds
1	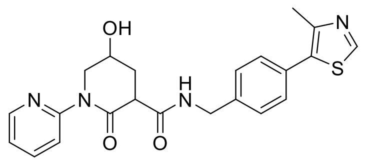	422.50	95.42	2	1.21	3
2	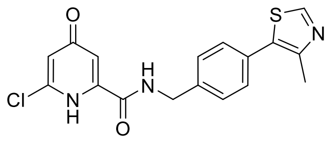	359.83	74.85	2	1.31	2
3	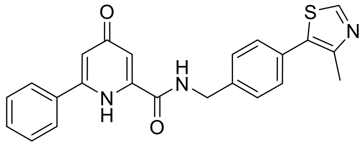	401.48	74.85	2	2.65	2
4	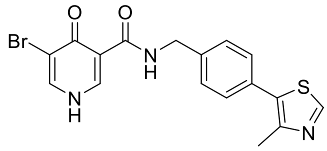	404.28	74.85	2	1.72	2
5	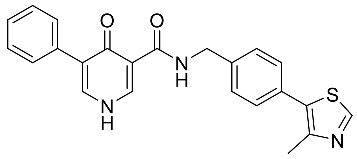	401.48	74.85	2	2.41	2
6	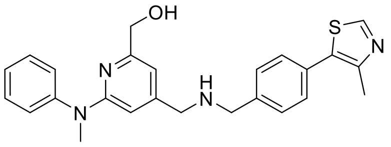	430.57	61.28	2	3.70	5
7	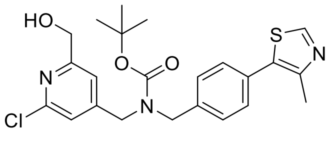	459.99	75.55	1	4.46	6

#	Structure	Molecular weight	TPSA	HBD count	clogP	rot. bonds
8	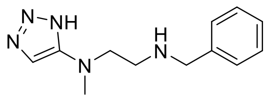	231.30	56.84	2	2.03	5
9	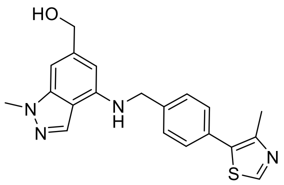	364.47	62.97	2	2.54	3
10	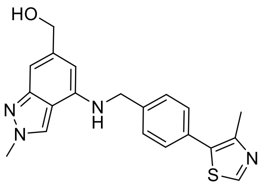	364.47	62.97	2	2.54	3
11	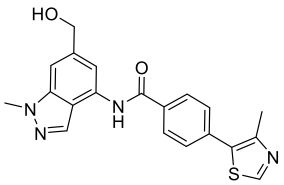	378.45	80.04	2	2.08	1
12	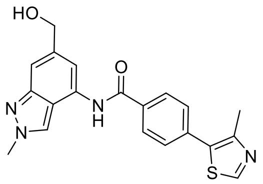	378.45	80.04	2	2.08	1
13	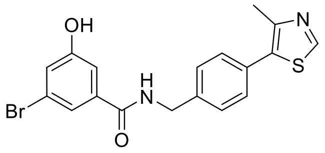	403.29	62.22	2	4.17	2
14	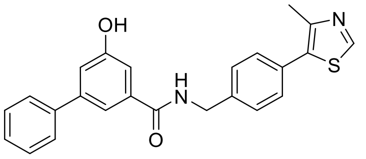	400.50	62.22	2	5.03	2

#	Structure	Molecular weight	TPSA	HBD count	clogP	rot. bonds
15	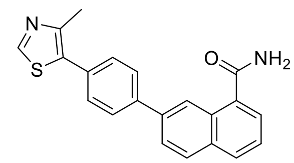	344.43	55.98	1	4.35	0
16	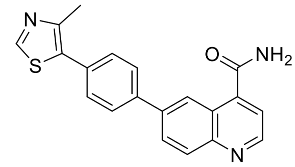	345.42	68.87	1	3.70	0
17	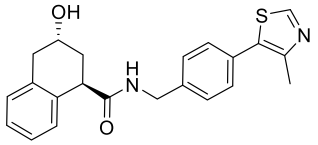	378.49	62.22	2	3.13	3
18	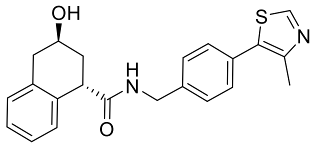	378.49	62.22	2	3.13	3
19	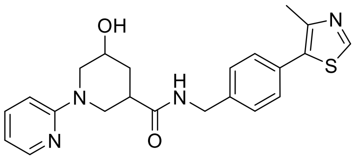	408.52	78.35	2	2.64	3
20	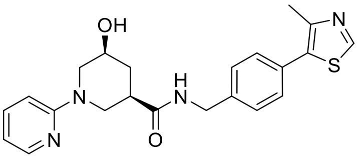	408.52	78.35	2	2.64	3
21	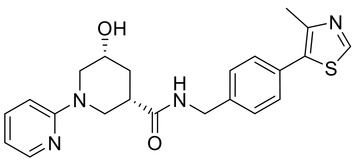	408.52	78.35	2	2.64	3

#	Structure	Molecular weight	TPSA	HBD count	clogP	rot. bonds
22	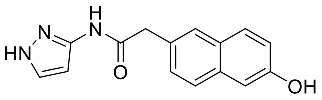	267.29	78.01	3	2.08	2
23	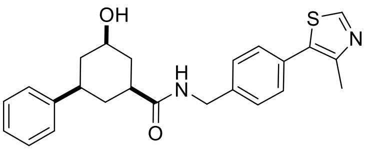	406.54	62.22	2	3.72	4
24	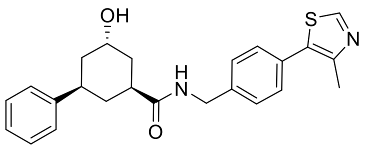	406.54	62.22	2	3.72	4
25	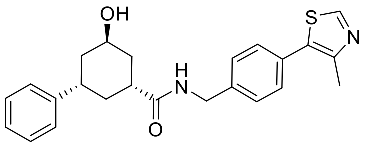	406.54	62.22	2	3.72	4
26	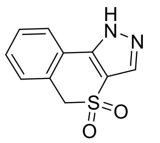	220.25	62.82	1	1.21	0
27	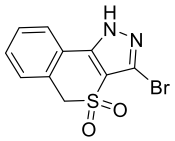	299.14	62.82	1	2.17	0
28	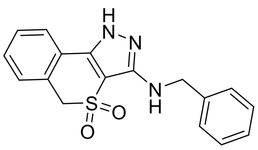	325.39	74.85	2	2.68	2

#	Structure	Molecular weight	TPSA	HBD count	clogP	rot. bonds
29	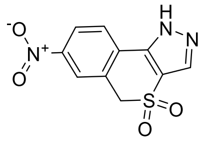	265.24	105.96	1	0.99	0
30	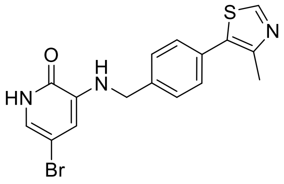	376.27	57.78	2	2.58	2
31	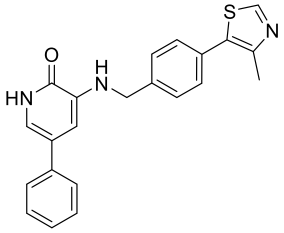	373.47	57.78	2	3.44	2
32	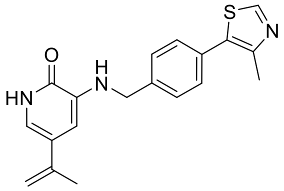	337.44	57.78	2	2.67	2
33	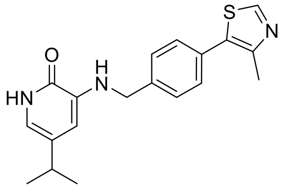	339.46	57.78	2	2.98	3
34	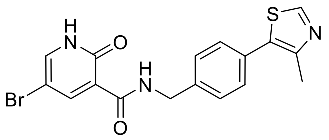	404.28	74.85	2	2.29	2
35	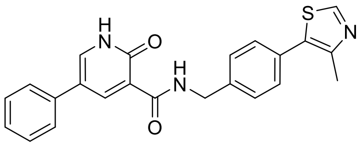	401.48	74.85	2	3.22	2

#	Structure	Molecular weight	TPSA	HBD count	clogP	rot. bonds
36	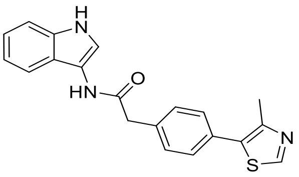	347.44	57.78	2	3.33	2
37	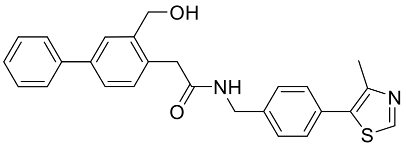	428.55	62.22	2	4.15	5
38	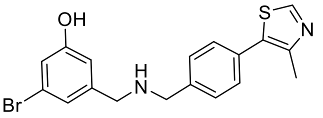	389.31	45.15	2	3.80	4
39	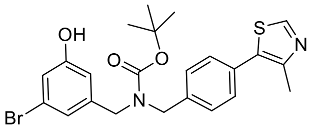	489.43	62.66	1	6.24	5
40	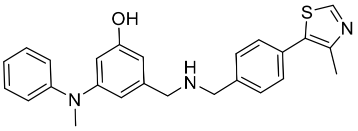	415.56	48.39	2	4.57	4
41	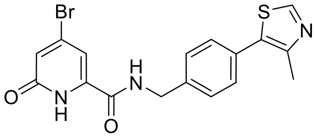	404.28	74.85	2	2.29	2
42	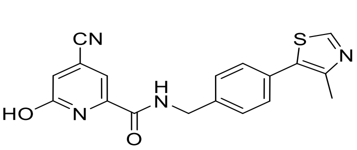	350.40	98.9	2	3.05	2
43	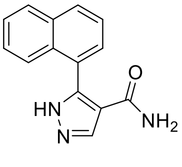	237.26	71.77	2	2.33	0

Supplementary Table 6: Affinity, solubility, and ^{19}F recovery.

#	Structure	TR-FRET IC_{50} [µM]	Solubility in NMR buffer [µM]	% ^{19}F recovery
1	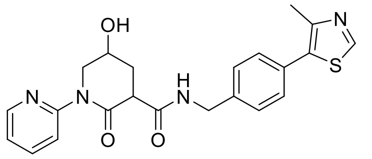	**264 ± 30**	>500.0	52%
2	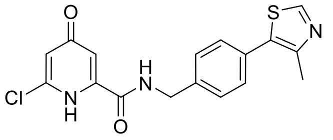	>100	20.0	0%
3	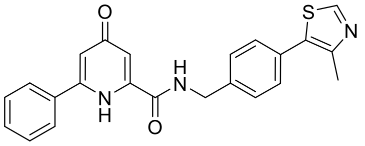	13.8 ± 1.8	280.0	23%
4	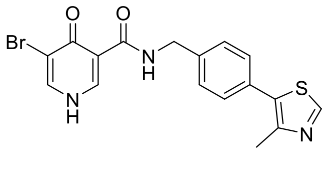	>100	40.0	n.a.
5	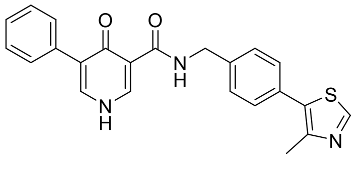	>100	20.0	n.a.
6	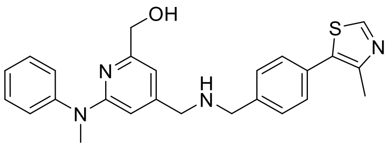	>500	70.0	n.a.
7	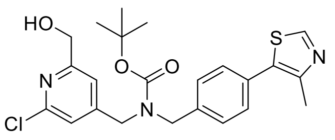	106 ± 5.2	60.0	n.a.

#	Structure	TR-FRET IC_{50} [µM]	Solubility in NMR buffer [µM]	% ^{19}F recovery
8	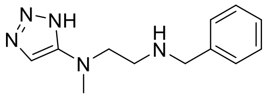	>500	430.0	0%
9	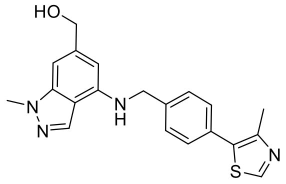	>100	110.0	4%
10	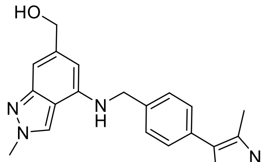	167 ± 47	240.0	6%
11	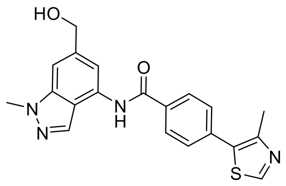	>500	180.0	28%
12	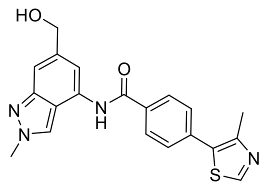	255 ± 45	70.0	n.a.
13	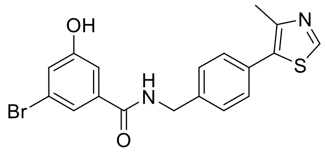	>100	20.0	7%
14	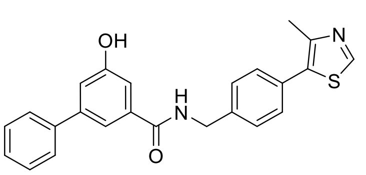	15.6 ± 1.2	<10.0	2%

#	Structure	TR-FRET IC_{50} [µM]	Solubility in NMR buffer [µM]	% ^{19}F recovery
15	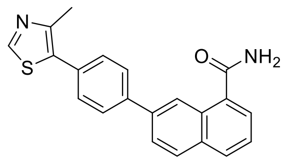	3.8 ± 1.8	<10.0	n.a.
16	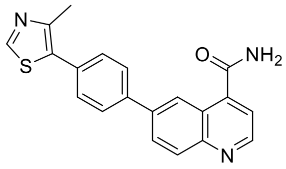	8.8 ± 0.8	<10.0	22%
17	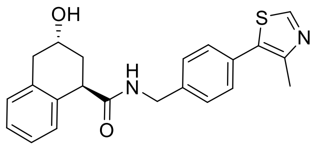	313 ± 135	400.0	18%
18	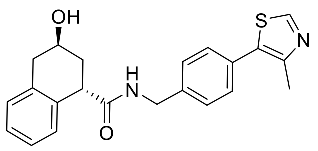	166 ± 77	>500.0	0%
19	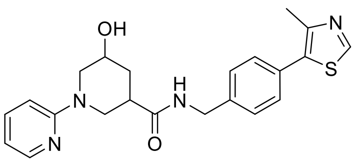	>100	230.0	6%
20	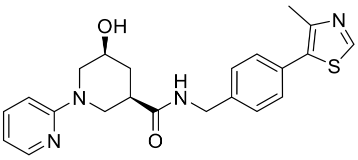	43.2 ± 5.4	140.0	50%
21	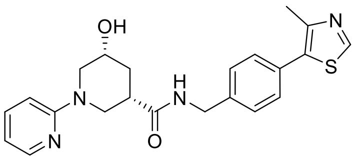	20.5 ± 1.6	90.0	42%

#	Structure	TR-FRET IC50 [µM]	Solubility in NMR buffer [µM]	% 19F recovery
22	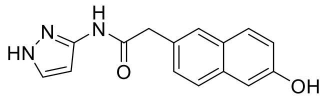	315 ± 44	350.0	13%
23	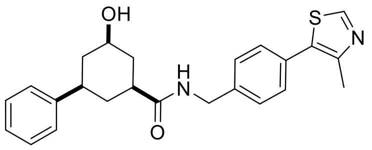	>100	110.0	15%
24	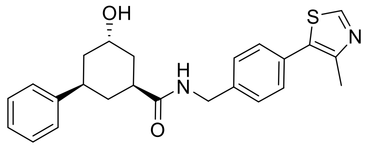	>100	<10.0	n.a.
25	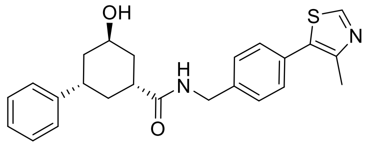	>100	<10.0	n.a.
26	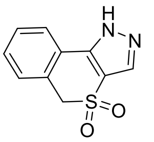	265 ± 74	>500.0	0%
27	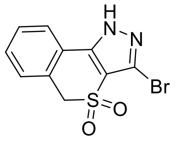	>500	>500.0	0%
28	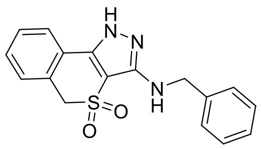	244 ± 22	360.0	5%

#	Structure	TR-FRET IC_{50} [µM]	Solubility in NMR buffer [µM]	% ^{19}F recovery
29	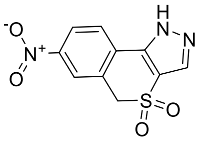	175 ± 18	n.a	n.a.
30	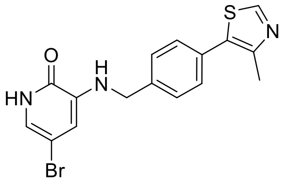	157 ± 1.8	30.0	0%
31	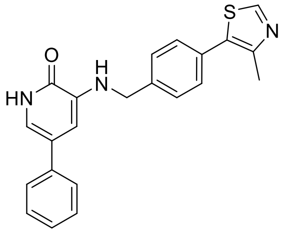	102 ± 22	20.0	0%
32	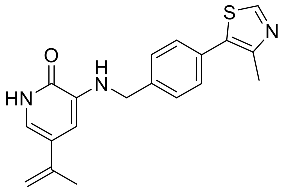	58.3 ± 43.1	20.0	n.a.
33	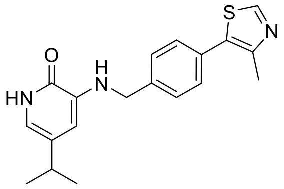	71.7 ± 49.3	50.0	n.a.
34	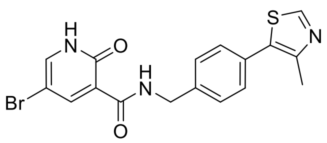	>100	20.0	6%
35	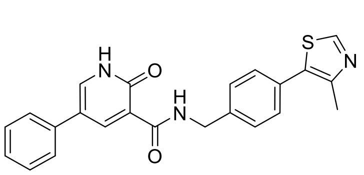	n.a.	<10.0	n.a.

#	Structure	TR-FRET IC_{50} [µM]	Solubility in NMR buffer [µM]	% ^{19}F recovery
36	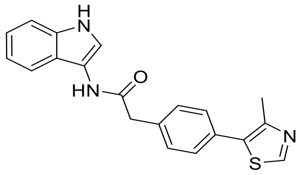	13.0 ± 4.5	<10.0	30%
37	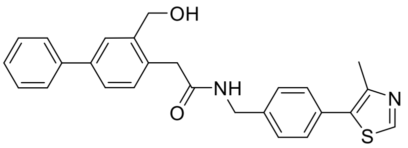	>100	<10.0	n.a.
38	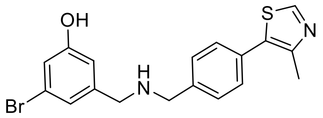	85.7 ± 16.2	80.0	8%
39	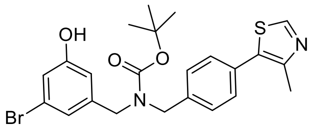	70.7 ± 0.4	<10.0	n.a.
40	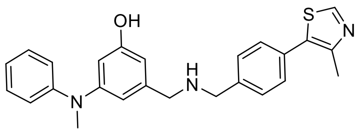	67.8 ± 5.1	<10.0	n.a.
41	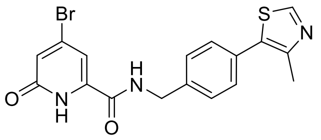	>100	<10.0	n.a.
42	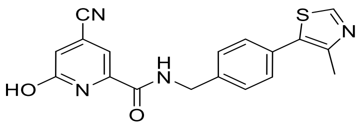	144 ± 10	>500.0	6%
43	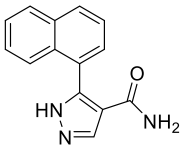	>500	>500.0	0%

Supplementary Table 7. Data collection and refinement statistics

**Data collection***	
Space group	P1
Cell dimensions	
a, *b*, *c* (Å)	47.38, 47.72, 98.76
α, β, γ (°)	81.07, 76.66, 82.80
Resolution (Å)	1.98
R_{merge}	0.07
$I / \sigma I$	8.9 / 1.7
Completeness, ellipsoidal (%)	87.3
Redundancy	3.4
**Refinement***	
Resolution (Å)	1.98
No. reflections	33542
R_{work} / R_{free}	22.88 /25.65
No. atoms	
Protein	5427
Ligand/ion	105
Water	329
B-factors	
Protein	55.49
Ligand/ion	53.49
Water	57.1
R.m.s. deviations	
Bond lengths (Å)	0.008
Bond angles (°)	0.95

*Values as output by STARANISO[2] and autoBUSTER[3]
**Values in parentheses are for highest-resolution shell.

Supplementary Table 8. Absolute binding free-energy predictions for compounds shown in Figure 4

**Ligand**	**Predicted free energy (ΔG) [kcal/mol]**
reference molecule 1	-11.8
bound diastereomer of compound 1	-6.0
player-designed compound	-6.9

Supplementary Methods

NMR spectroscopy

NMR experiments were recorded on Bruker Avance HD 400 MHz or 500 MHz spectrometer equipped with a Prodigy BBO probe or TCI cryoprobe, respectively. Samples were dissolved in 600 µL DMSO-d6. Temperature was set to 298 K. 1D ^{1}H spectra were acquired with 30° excitation pulses and an interpulse delay of 4.2 sec with 64k data points and 20 ppm sweep width. 1D ^{13}C spectra were acquired with broadband composite pulse decoupling (WALTZ16) and an interpulse delay of 3.3 sec with 64 k data points and a sweep width of 240 ppm. ^{13}C DEPT- modulated spectra were phased so that C, CH_2 are positive, and CH, CH_3 are negative.

Processing of spectra was performed with Bruker Topspin 3.6 software. Spectra were analyzed with ACD/NMR Workbook 2023. Chemical shifts are reported in ppm on the δ scale. No zero filling was performed and spectra were manually integrated after automatic baseline correction.

2D HSQC spectra were recorded on all samples to aid the interpretation of the data and to identify signals hidden underneath solvent peaks. Spectra were acquired with sweep widths obtained by automatic sweep width detection from 1D reference spectra in the direct dimension with 1k datapoints and with 210 ppm and 256 datapoints in the indirect dimension.

HPLC-MS

All samples were analyzed on an Agilent 1200 series LC system coupled with an Agilent 6140 mass spectrometer. Purity was determined via UV detection with a bandwidth of 170 nm in the range from 230-400 nm. LC parameters were as follows: Waters Xbridge C18 column, 2.5 µm particle size, 2.1 x 20 mm. Run time 2.1 minutes, flow 1 ml/min, column temperature 60°C and 5 µl injections. Solvent A (20 mM NH_4HCO_3/ NH_3 pH 9), solvent B (MS grade acetonitrile). Start 10 % B, gradient 10 % - 95 % B from 0.0 - 1.5 min, 95 % B from 1.5 - 2.0 min, gradient 95 % - 10 % B from 2.0 – 2.1 min.

HRMS

The mass calibration was performed using the Pierce LTQ Velos ESI positive ion calibration solution from Thermo Scientific (Product Nr. 88323).

MS parameters: The scan window was set to 200-1200 amu with a maximum injection time of 500 ms and 1 microscan. The resolution of the Orbitrap was 120000 with a mass accuracy $\leqslant$ 5 ppm. The ion mode set to positive with a capillary temperature 275 °C and voltage of 60 eV. The tube lens potential was set to 110 eV. 12 NanoESI voltage was 1.45 kV and the N2-gas pressure set to 0.45 psi. Total sample volume was 1 µl and the acquisition time was 0.4 sec, with 10 scans of averaging per spectrum Sample dilution: 10 mM DMSO stock solution was diluted 1:1000 in 25 % acetonitrile +0.01 % formic acid.

Synthetic Procedures

Synthesis of 1

Reaction scheme:

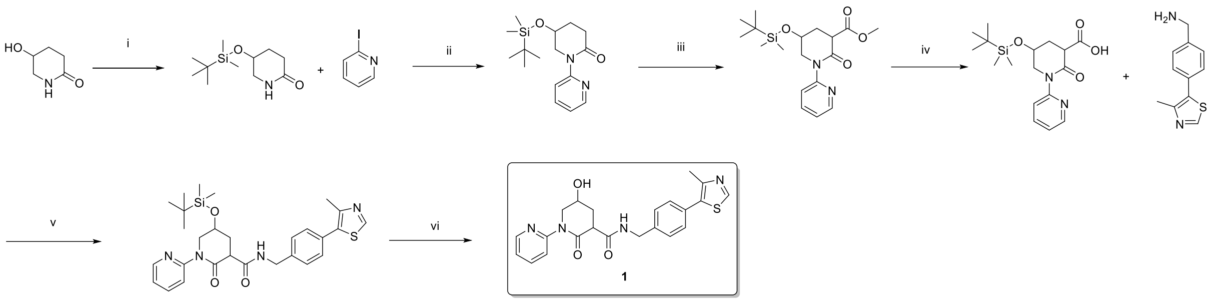

i) TBDMSCl, Imidazole, DMF, 0°C-RT; ii) $Pd_2(dba)_3$, Xanthphos, Cs_2CO_3, toluene, 110 °C; iii) 1.0M LiHMDS in THF, Dimethyl carbonate, THF, -78 °C; iv) $LiOH.H_2O$, THF:water:MeOH (1:1:1), 0°C-RT; v) T3P, pyridine, 0°C-RT; vi) HCl, DCM

5-hydroxy-N-(4-(4-methylthiazol-5-yl)benzyl)-2-oxo-1-(pyridin-2-yl)piperidine-3-carboxamide (1):

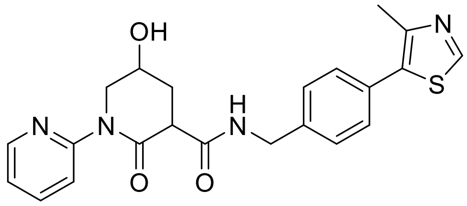

To a stirred solution of 5-hydroxypiperidin-2-one (3.50 g, 30.4 mmol) in DMF (30 mL), tert-butyldimethylsilyl chloride (5.47 g, 36.5 mmol) and imidazole (3.10 g, 45.6 mmol) were added at 0°C and the reaction mixture was stirred for 16 h at RT. Upon completion of the reaction, the mixture was quenched with ice cold water (100 mL) and stirred for 10 min. The obtained solids were filtered, rinsed with water and dried under reduced pressure to give 5-[tert-butyl(dimethyl)silyl] oxypiperidin-2-one (6.00 g, 77% yield) which was directly used in the next step without further purification.

To a stirred solution of 5-[tert-butyl(dimethyl)silyl] oxypiperidin-2-one (1.00 g, 4.36 mmol) in Toluene (20 mL), 2-iodopyridine (1.79 g, 8.72 mmol) and Caesium carbonate (2.13 g, 6.54 mmol) were added and purged with argon for 5 min. $Pd_2(dba)_3$ (0.20 g, 0.22 mmol) and Xantphos (0.15 g, 0.26 mmol) were added and the reaction continued at 110°C for 16 h. Upon completion of the reaction, the reaction mixture was cooled to RT and concentrated under reduced pressure. The crude residue was purified by column chromatography (0-70% EtOAc in petroleum ether) to give 5-[tert-butyl(dimethyl)silyl]oxy-1-(2-pyridyl)piperidin-2-one (440 mg, 31% yield).

A stirred solution of 5-[tert-butyl(dimethyl)silyl]oxy-1-(2-pyridyl)piperidin-2-one (440 mg, 1.44 mmol) in THF (10 mL), was cooled to -78°C and 1.0 M LiHMDS in THF (0.99 mL, 5.02 mmol) was added dropwise and reaction continued at the same temperature for 30 min. Dimethyl carbonate (323 mg, 3.59 mmol) was added dropwise and reaction continued at the same temperature for 30 min and at -10°C for 1 h. Upon completion of reaction, the mixture was quenched with sat. ammonium chloride solution (40 mL) and extracted with ethyl acetate (2*50 mL). The organic layer was dried over

sodium sulphate, filtered and concentrated. The obtained crude product was purified by column chromatography (10-60% EtOAc/petroleum ether) to give methyl 5-[tert-butyl(dimethyl)silyl]oxy-2-oxo-1-(2-pyridyl)piperidine-3-carboxylate (400 mg, 73% yield) which was directly used in the next step.

To a stirred solution of methyl 5-[tert-butyl(dimethyl)silyl]oxy-2-oxo-1-(2-pyridyl)piperidine-3-carboxylate (1.00 g, 2.74 mmol) in THF (5 mL), methanol (5 mL) and water (5 mL) was added LiOH.H_2O (138 mg, 2.39 mmol) at 0°C and the reaction mixture was stirred for 2 h at RT. Upon completion of the reaction, the mixture was concentrated under reduced pressure. The resultant residue was dissolved in water (20 mL), acidified with 1 N aq. HCl and extracted with DCM (2*150 mL). The organic layer was dried over sodium sulphate, filtered and concentrated to give 5-((tert-butyldimethylsilyl)oxy)-2-oxo-1-(pyridin-2-yl)piperidine-3-carboxylic acid (0.8 g, crude), which was directly used in the next step without further purification.

To a stirred solution of 5-[tert-butyl(dimethyl)silyl] oxy-2-oxo-1-(2-pyridyl)piperidine-3-carboxylic acid (0.80 g, 2.28 mmol) and [4-(4-methylthiazol-5-yl)phenyl]methanamine; hydrochloride(0.55 g, 2.28 mmol) in Pyridine (8 mL), T3P (2.0 mL, 6.85 mmol) was added at 0°C and reaction continued for 2h at RT. Upon completion of the reaction, the mixture was quenched with ice cold water (150 mL) and extracted with ethyl acetate (2*150 mL). The organic layer was dried over sodium sulphate, filtered and concentrated. Obtained crude product was purified by column chromatography (20-80% EtOAc/petroleum ether) to give 5-[tert-butyl(dimethyl)silyl]oxy-N-[[4-(4-methylthiazol-5-yl)phenyl]methyl]-2-oxo-1-(2-pyridyl)piperidine-3-carboxamide (330 mg, 25% yield) that directly used in the next step.

To a stirred solution of 5-[tert-butyl(dimethyl)silyl]oxy-N-[[4-(4-methylthiazol-5-yl)phenyl]methyl]-2-oxo-1-(2-pyridyl)piperidine-3-carboxamide (0.80 g, 1.49 mmol) in DCM (10 mL), 4.0 M HCl in 1,4-Dioxane (0.27 g, 7.45 mmol) was added at 0°C and reaction continued for 2 h at the same temperature. Upon completion of the reaction, the mixture was concentrated under reduced pressure. The obtained crude product was purified by reverse phase column chromatography (0-70% ACN/0.1% FA in water). The product-containing fractions were lyophilized to give 5-hydroxy-N-[[4-(4-methylthiazol-5-yl)phenyl]methyl]-2-oxo-1-(2-pyridyl)piperidine-3-carboxamide **1** (0.50 g, 1.18 mmol, 77% yield).

^{1}H NMR (DMSO-d$_6$, 400 MHz) δ 8.98 (s, 1H), 8.78 (m, 1H), 8.45 (br d, 1H, *J*=3.8 Hz), 7.88 (m, 1H), 7.70 (d, 1H, *J*=8.1 Hz), 7.43 (q, 4H, *J*=8.3 Hz), 7.15 (m, 1H), 5.25 (d, 1H, *J*=2.8 Hz), 4.35 (m, 3H), 3.50-4.23 (m, 3H), 2.45 (s, 3H), 2.26 (s, 1H), 2.05 (m, 1H)

^{13}C NMR (DMSO-d$_6$, 101 MHz) δ 170.1, 167.8, 154.1, 151.5, 147.8, 139.2, 137.2, 131.1, 129.8, 128.8, 127.5, 120.8, 120.3, 120.1, 61.8, 53.8, 46.6, 41.8, 31.8, 15.9

HRMS (*m/z*): $[M+H]^+$ calculated for $C_{22}H_{22}N_4O_3S$, 423.14854; found, 423.14883

R_f value (SiO_2, DCM:MeOH=14:1) = 0.3

Synthesis of 2

Reaction scheme:

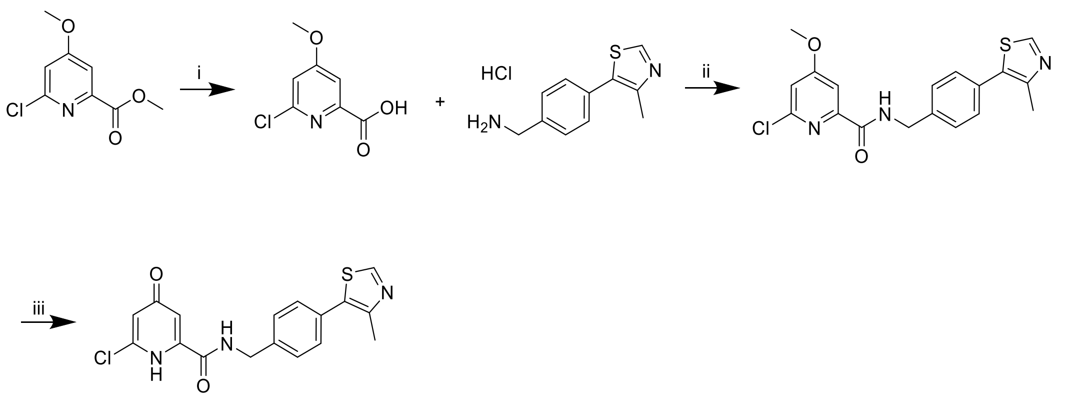

2

i) LiOH•H_2O, THF:H_2O (1:1), 0°C; ii) HATU, DIPEA, DMF, 0°C-RT; iii) pyridine•HCl, 150°C

6-chloro-N-(4-(4-methylthiazol-5-yl) benzyl)-4-oxo-1,4-dihydropyridine-2-carboxamide (2):

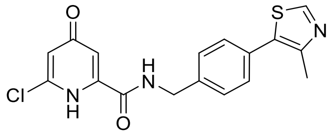

To a stirred solution of methyl 6-chloro-4-methoxy-pyridine-2-carboxylate (300 mg, 1.49 mmol) in THF (4 mL) and water (4 mL) was added LiOH.H_2O (94 mg, 2.23 mmol) at 0°C and the reaction mixture was stirred for 2h at RT. Upon completion of the reaction, the mixture was concentrated under reduced pressure. The resultant residue was dissolved in water (20 mL), acidified with 1 N aq. HCl, and stirred for 10 min. The obtained solids were filtered, rinsed with water, and dried under reduced pressure to give 6-chloro-4-methoxy-pyridine-2-carboxylic acid (220 mg, 71% yield) which was used without further purification.

To a stirred solution of 6-chloro-4-methoxy-pyridine-2-carboxylic acid (220 mg, 1.17 mmol) and [4-(4-methylthiazol-5-yl) phenyl] methanamine hydrochloride **3** (311 mg, 1.29 mmol) in DMF (3 mL) was added DIPEA (0.61 mL, 3.52 mmol) and HATU (669 mg, 1.76 mmol) at 0°C and the reaction mixture was stirred at RT for 2 h. Upon completion of the reaction, the mixture was diluted with ice cold water (50 mL) and extracted with EtOAc (2*30 mL). The organic layer was washed with brine, dried over Na_2SO_4, filtered and concentrated under reduced pressure. The crude residue was purified by column chromatography (0-70% EtOAc in petroleum ether) to give 6-chloro-4-methoxy-N-(4-(4-methylthiazol-5-yl) benzyl) picolinamide (300 mg, 63% yield), which was directly used in the next step.

A mixture of 6-chloro-4-methoxy-N-[[4-(4-methylthiazol-5-yl) phenyl] methyl] pyridine-2-carboxamide (200 mg, 0.535 mmol) and Pyridine hydrochloride (618 mg, 5.35 mmol) was stirred at 150°C for 2 h. Upon completion of the reaction, the mixture was cooled to RT, diluted with ice cold water (50 mL) and stirred for 10 min. The obtained solids were collected by filtration and dried under reduced pressure. The crude residue was purified by column chromatography (0-70 % EtOAc in petroleum ether) to give 6-chloro-N-[[4-(4-methylthiazol-5-yl) phenyl] methyl]-4-oxo-1H-pyridine-2-carboxamide **2** (75 mg, 0.20 mmol, 39% yield).

^{1}H NMR (DMSO-d_6, 400 MHz) δ 11.65 (s, 1H), 9.20 (t, 1H, *J*=6.3 Hz), 8.98 (s, 1H), 7.4-7.5 (m, 5H), 7.00 (d, 1H, *J*=2.0 Hz), 4.49 (d, 2H, *J*=6.3 Hz), 2.44 (s, 3H)

^{13}C NMR (DMSO-d$_6$, 100 MHz) δ 167.2, 162.7, 151.7, 151.5, 150.2, 147.8, 139.3, 131.1, 129.9, 128.9, 128.0, 113.1, 109.8, 42.2, 15.9

HRMS (*m/z*): $[M+H]^+$ calculated for $C_{17}H_{14}ClN_3O_2S$, 360.05680; found, 360.05716

R_f value (SiO_2, DCM:MeOH=9:1) = 0.6

Synthesis of 3

Reaction scheme:

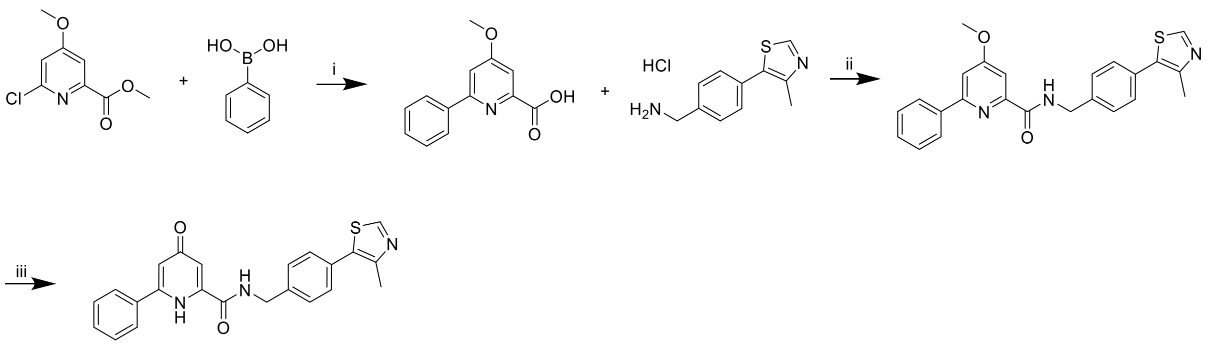

3

i) Pd(dppf)Cl_2 • CH_2Cl_2, Cs_2CO_3, dioxane, water, 100 °C; ii) HATU, DIPEA, DMF, 0°C-RT; iii) pyridine•HCl, 150°C;

N-(4-(4-methylthiazol-5-yl)benzyl)-4-oxo-6-phenyl-1,4-dihydropyridine-2-carboxamide (3):

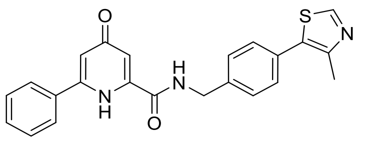

A stirred solution of 6-chloro-4-methoxy-pyridine-2-carboxylate (200 mg, 0.99 mmol), phenylboronic acid (133 mg, 1.09 mmol) and cesium carbonate (645 mg, 1.98 mmol) in 1,4-dioxane (3.5 mL) and water (1.5 mL) was purged with nitrogen for 5 min and [1,1′-Bis(diphenylphosphino)ferrocene]dichloropalladium(II), complex with dichloromethane (41 mg, 0.050 mmol) was added and the reaction continued for 16 h at 100°C. Upon completion of the reaction, the mixture was cooled to RT and concentrated under reduced pressure. The resultant residue was purified by reverse phase column chromatography to give 4-methoxy-6-phenyl-pyridine-2-carboxylic acid (160 mg, 63% yield) which was directly used in the next step.

To a stirred solution of 4-methoxy-6-phenyl-pyridine-2-carboxylic acid (150 mg, 0.65 mmol) in DMF (3 mL) was added DIPEA (0.61 mL, 3.52 mmol) and HATU (669 mg, 1.76 mmol) at 0°C and the reaction mixture was stirred for 10 min. [4-(4-methylthiazol-5-yl)phenyl]methanamine hydrochloride (173 mg, 0.72 mmol) was added and reaction continued for 2h at RT. Upon completion of the reaction, the mixture was diluted with ice cold water (45 mL) and extracted with EtOAc (50 mL). The organic layer was washed with water and brine solution, dried over Na_2SO_4, filtered and concentrated under reduced pressure. The crude residue was purified by column chromatography (0-60% EtOAc in petroleum ether) to give 4-methoxy-N-[[4-(4-methylthiazol-5-yl)phenyl]methyl]-6-phenyl-pyridine-2-carboxamide (180 mg, 57% yield) which was directly used in the next step.

A mixture of 4-methoxy-N-[[4-(4-methylthiazol-5-yl)phenyl]methyl]-6-phenyl-pyridine-2-carboxamide (130 mg, 0.31 mmol) and Pyridine hydrochloride (362 mg, 3.13 mmol) was stirred at 150°C for 2 h. Upon completion of the reaction, the mixture was cooled to RT, diluted with ice cold water (50 mL), and stirred for 10 min. The obtained solids were collected by filtration and dried under reduced pressure. The crude residue was purified by column chromatography (0-70% EtOAc in petroleum ether) to give N-[[4-(4-methylthiazol-5-yl)phenyl]methyl]-4-oxo-6-phenyl-1H-pyridine-2-carboxamide **3** (75 mg, 0.18 mmol, 57% yield).

^{1}H NMR (DMSO-d$_6$, 500 MHz) δ 11.09 (br s, 1H), 9.44 (t, 1H, *J*=6.5 Hz), 8.97 (s, 1H), 8.2-8.3 (m, 2H), 7.4-7.5 (m, 10H), 4.58 (d, 1H, *J*=6.6 Hz), 2.44 (s, 3H)

^{13}C NMR (DMSO-d$_6$, 125 MHz) δ 166.1, 164.2, 156.8, 151.5, 151.5, 147.8, 139.7, 137.8, 131.1, 129.9, 129.4, 128.9, 128.6, 127.8, 126.9, 109.6, 108.4, 42.1, 15.9

HRMS (*m/z*): $[M+H]^+$ calculated for $C_{23}H_{19}N_3O_2S$, 402.12707; found, 402.1275

R_f value (SiO_2, DCM:MeOH=14:1) = 0.2

Synthesis of 4

Reaction scheme:

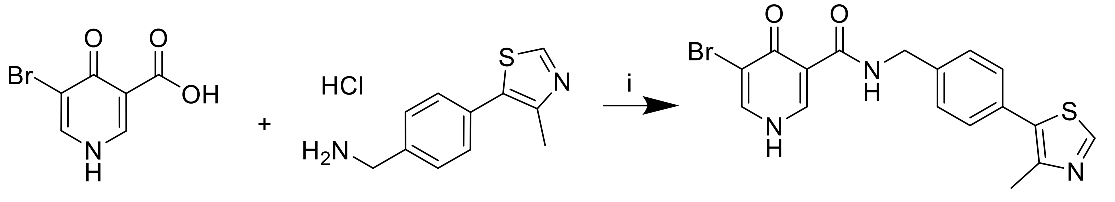

4

i) HATU, DIPEA, DMF, 0 °C-RT.

5-bromo-N-(4-(4-methylthiazol-5-yl)benzyl)-4-oxo-1,4-dihydropyridine-3-carboxamide (4):

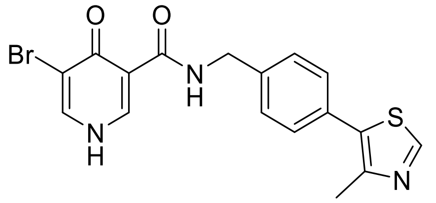

To a stirred solution of 5-bromo-4-oxo-1,4-dihydropyridine-3-carboxylic acid (1.20 g, 5.50 mmol) in DMF (12 mL) was added DIPEA (0.61 mL, 3.52 mmol) and HATU (669 mg, 1.76 mmol) at 0°C and the reaction mixture was stirred for 10 min. Then [4-(4-methylthiazol-5-yl) phenyl] methanamine hydrochloride (311 mg, 1.29 mmol) was added and reaction continued for 16 h at RT. Upon completion of the reaction, the mixture was diluted with ice cold water (100 mL) and stirred for 10 min. The obtained solids were collected by filtration, rinsed with diethyl ether, and dried under reduced pressure to give 5-bromo-N-[[4-(4-methylthiazol-5-yl)phenyl]methyl]-4-oxo-1H-pyridine-3-carboxamide **4** (1.50 g, 37.12 mmol, 62% yield).

^{1}H NMR (DMSO-d_6, 500 MHz) δ 12.45 (m, 1H), 10.53 (t, 1H, *J*=6.0 Hz), 8.99 (s, 1H), 8.48 (d, 1H, *J*=1.6 Hz), 8.38 (d, 1H, *J*=1.6 Hz), 7.4-7.5 (m, 2H), 7.4-7.4 (m, 2H), 4.57 (t, 2H, *J*=6.0 Hz), 2.45 (s, 3H)

^{13}C NMR (DMSO-d_6, 125 MHz) δ 172.0, 163.5, 151.5, 147.9, 141.4, 139.4, 139.1, 131.0, 130.1, 129.0, 127.9, 117.2, 116.2, 41.8, 15.9

HRMS (*m/z*): $[M+H]^+$ calculated for $C_{17}H_{14}N_3O_2S$, 404.00629; found, 404.00656

R_f value (SiO_2, DCM:MeOH=14:1) = 0.5

Synthesis of 5

Reaction scheme:

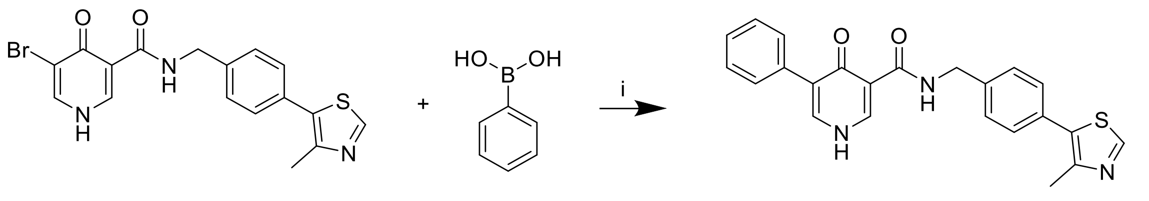

5

i) Pd(dppf)Cl_2·CH_2Cl_2, Cs_2CO_3, dioxane, water, 100°C

N-(4-(4-methylthiazol-5-yl)benzyl)-4-oxo-5-phenyl-1,4-dihydropyridine-3-carboxamide (5)

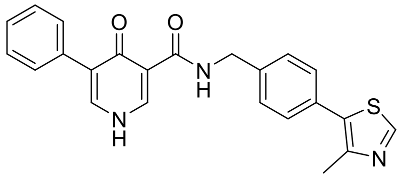

A stirred solution of 5-bromo-N-(4-(4-methylthiazol-5-yl)benzyl)-4-oxo-1,4-dihydropyridine-3-carboxamide (0.10 g, 0.25 mmol), phenylboronic acid (0.033 g, 0.27 mmol) and Caesium carbonate (0.12 g, 0.371 mmol) in 1,4-Dioxane (4 mL) and Water (1.5 mL) was purged with nitrogen for 5 min and [1,1′-Bis(diphenylphosphino)ferrocene]dichloropalladium(II), complex with dichloromethane (0.010 g, 0.012 mmol) was added and the reaction continued for 16 h at 100°C. Upon completion of the reaction, the mixture was cooled to RT, quenched with ice cold water (25 mL), and extracted with EtOAc (2*75 mL). The combined organic layer was dried over sodium sulphate, filtered and concentrated. The resultant crude compound was purified by prep-HPLC, to give N-[[4-(4-methylthiazol-5-yl)phenyl]methyl]-4-oxo-5-phenyl-1H-pyridine-3-carboxamide **5** (40 mg, 0.09 mmol, 38% yield).

^{1}H NMR (DMSO-d$_6$, 500 MHz) δ 11.12 (br t, 1H, *J*=5.7 Hz), 8.98 (s, 1H), 8.51 (d, 1H, *J*=0.9 Hz), 8.41 (br s, 1H), 7.97 (d, 1H, *J*=0.9 Hz), 7.60 (d, 2H, *J*=7.3 Hz), 7.4-7.5 (m, 2H), 7.4-7.4 (m, 2H), 7.3-7.4 (m, 2H), 7.31 (d, 1H, *J*=7.3 Hz), 4.57 (d, 2H, *J*=6.0 Hz), 2.45 (s, 3H)

^{13}C NMR (DMSO-d$_6$, 125 MHz) δ 175.1, 165.0, 151.5, 147.8, 142.5, 139.5, 138.7, 135.3, 131.1, 129.9, 129.9, 129.0, 128.8, 127.9, 127.7, 127.0, 117.9, 41.7, 15.9

HRMS (*m/z*): $[M+H]^+$ calculated for $C_{23}H_{19}N_3O_2S$, 402.12707; found, 402.12714

R_f value (SiO_2, DCM:MeOH=14:1) = 0.4

Synthesis of 6

Reaction scheme:

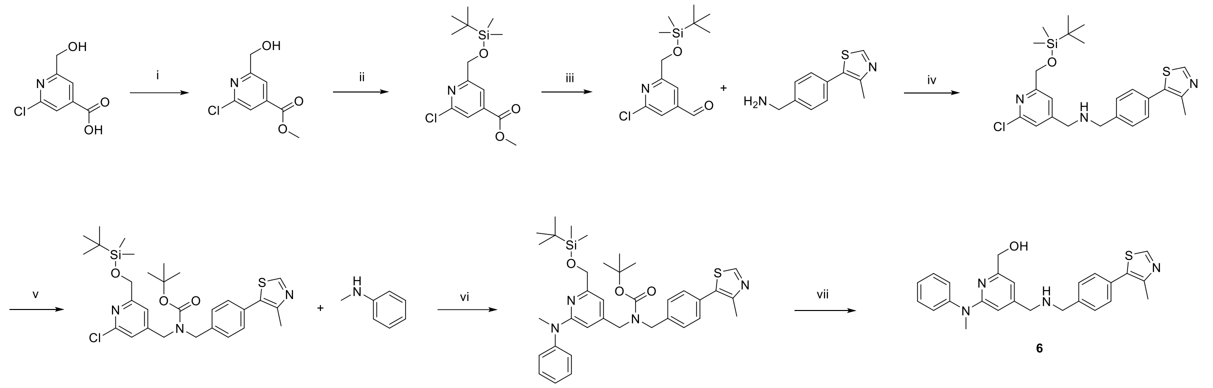

i) MeOH, H_2SO_4, reflux; ii) TBDMSCl, Imidazole, DCM; iii) DIBAL-H, THF, -78^oC; iv) STAB, AcOH, DCM; v) Boc anhydride, Et_3N, DCM; vi) $Pd_2(dba)_3$, Xantphos, Cs_2CO_3, toluene, 110^oC; vii) 4.0 M HCl, DCM

(6-(methyl(phenyl)amino)-4-(((4-(4-methylthiazol-5-yl)benzyl)amino)methyl)pyridin-2-yl)methanol (6):

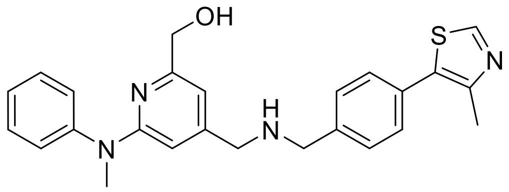

To a stirred solution of 2-chloro-6-(hydroxymethyl)pyridine-4-carboxylic acid (1.00 g, 5.33 mmol) in Methanol (15 mL), Sulphuric acid (0.57 mL, 10.7 mmol) was added at 0°C and the reaction mixture was stirred for 7 h at 70°C. Upon completion of the reaction, it was cooled to RT and concentrated under reduced pressure. The obtained residue was quenched with ice cold water (30 mL) and stirred for 10 min. The obtained solids were filtered, rinsed with water and dried under reduced pressure to give methyl 2-chloro-6-(hydroxymethyl)pyridine-4-carboxylate (1.00 g, 73% yield) which was directly used in the next step without purification.

To a stirred solution of methyl 2-chloro-6-(hydroxymethyl)pyridine-4-carboxylate (1.00 g, 4.96 mmol) in DCM (10 mL), tert-butyldimethylsilyl chloride (0.8223 g, 5.46 mmol) and Imidazole (0.21 mL, 4.96 mmol) were added at 0°C and the reaction mixture was stirred for 16 h at RT. Upon completion of the reaction, it was quenched with ice cold water (100 mL) and extracted with DCM (150 mL). The organic layer was washed with water (100 mL), dried over sodium sulphate, filtered, and concentrated under reduced pressure. The resultant residue was purified by column chromatography (0-30% EtOAc/petroleum ether), to give methyl 2-chloro-6-[(1-methyl-1-trimethylsilyl-ethoxy)methyl]pyridine-4-carboxylate (1.30 g, 78% yield) which was directly used in the next step.

To a stirred solution of methyl 2-[[tert-butyl(dimethyl)silyl]oxymethyl]-6-chloro-pyridine-4-carboxylate (1.30 g, 4.12 mmol) in DCM (15 mL), 1M DIBAL-H in toluene (0.64 g, 4.53 mmol) was added dropwise at -78°C and the reaction was continued at the same temperature for 2 h. Upon completion of the reaction, the mixture was quenched with saturated ammonium chloride solution (50 mL) and extracted with DCM (2*125 mL). The organic layer was dried over sodium sulphate, filtered, and concentrated. The resultant residue was purified by column chromatography (0-20% EtOAc/petroleum ether) to give 2-[[tert-butyl(dimethyl)silyl]oxymethyl]-6-chloro-pyridine-4-carbaldehyde (1.10 g, 72% yield) which was directly used in the next step.

To a stirred solution of 2-[[tert-butyl(dimethyl)silyl]oxymethyl]-6-chloro-pyridine-4-carbaldehyde (1.10 g, 3.85 mmol) and [4-(4-methylthiazol-5-yl)phenyl]methanamine hydrochloride (0.74 g, 3.08 mmol) in DCM (15 mL), Acetic Acid (0.069 g, 1.15 mmol) was added dropwise at RT and the reaction continued at the same temperature for 2 h. Then sodium triacetoxyborohydride (2.45 g, 11.5 mmol) was added at 0°C and the reaction continued at RT for 16 h. Upon completion of the reaction, it was quenched with ice cold water (100 mL) and extracted with DCM (2*100 mL). The combined organic layer was washed with water, dried over sodium sulphate, filtered, and concentrated. The resultant residue was purified by column chromatography (30-80% EtOAC/petroleum ether) to give N-[[2-[[tert-butyl(dimethyl)silyl]oxymethyl]-6-chloro-4-pyridyl]methyl]-1-[4-(4-methylthiazol-5-yl)phenyl]methanamine (0.68 g, 35% yield) which was directly used in the next step.

To a stirred solution of N-[[2-[[tert-butyl(dimethyl)silyl]oxymethyl]-6-chloro-4-pyridyl]methyl]-1-[4-(4-methylthiazol-5-yl)phenyl]methanamine (680 mg, 1.43 mmol) in DCM (10 mL), Triethylamine (0.59 mL,

4.30 mmol) and Boc anhydride (0.36 mL, 1.58 mmol) were added at 0°C and the reaction was continued at RT for 4 h. Upon completion of reaction, it was quenched with ice cold water (50 mL) and extracted with DCM (2*75 mL). The organic layer was dried over sodium sulphate, filtered, and concentrated. The obtained crude was purified by column chromatography (0-40% EtOAc/petroleum ether) to give tert-butyl N-[[2-[[tert-butyl(dimethyl)silyl]oxymethyl]-6-chloro-4-pyridyl]methyl]-N-[[4-(4-methylthiazol-5-yl)phenyl]methyl]carbamate (600 mg, 70% yield) which was directly used in the next step.

To a stirred solution of tert-butyl N-[[2-[[tert-butyl(dimethyl)silyl]oxymethyl]-6-chloro-4-pyridyl]methyl]-N-[[4-(4-methylthiazol-5-yl)phenyl]methyl]carbamate (100 mg, 0.17 mmol) and N-methylaniline (28 mg, 0.26 mmol) in Toluene (4 mL), Caesium carbonate (113 mg, 0.35 mmol) was added and the mixture was purged with nitrogen for 5 min. $Pd_2(dba)_3$ (8 mg, 0.0087 mmol) and Xanthphos (6 mg, 0.010 mmol) were added and the reaction continued at 110°C for 16 h. Upon completion of the reaction, the mixture was concentrated under reduced pressure. The resultant residue was purified by column chromatography to give tert-butyl N-[[2-[[tert-butyl(dimethyl)silyl]oxymethyl]-6-(N-methylanilino)-4-pyridyl]methyl]-N-[[4-(4-methylthiazol-5-yl)phenyl]methyl]carbamate (90 mg, 64% yield) which was directly used in the next.

To a stirred solution of tert-butyl N-[[2-[[tert-butyl(dimethyl)silyl]oxymethyl]-6-(N-methylanilino)-4-pyridyl]methyl]-N-[[4-(4-methylthiazol-5-yl)phenyl]methyl]carbamate (90 mg, 0.14 mmol) in DCM (2 mL), 4.0M HCl in 1,4-dioxane (50 mg, 1.40 mmol) was added dropwise at 0°C and the reaction continued at the same temperature for 3 h. Upon completion of the reaction, the mixture was concentrated under reduced pressure. The resultant residue was purified by prep-HPLC to give [6-(N-methylanilino)-4-[[[4-(4-methylthiazol-5-yl)phenyl]methylamino]methyl]-2-pyridyl]methanol **6** (35 mg, 0.08 mmol, 57% yield).

^{1}H NMR (DMSO-d$_6$, 400 MHz) δ 8.98 (s, 1H), 7.4-7.4 (m, 4H), 7.3-7.4 (m, 2H), 7.28 (d, 2H, *J*=7.4 Hz), 7.20 (t, 1H, *J*=7.2 Hz), 6.80 (s, 1H), 6.45 (s, 1H), 5.19 (br t, 1H, *J*=5.6 Hz), 4.43 (d, 2H, *J*=4.8 Hz), 3.62 (s, 2H), 3.54 (s, 2H), 3.4-3.4 (m, 3H), 2.4-2.5 (m, 3H)

^{13}C NMR (DMSO-d$_6$, 100 MHz) δ 159.8, 157.7, 151.4, 150.8, 147.7, 146.6, 140.4, 131.2, 129.7, 129.5, 128.6, 128.5, 125.5, 124.8, 109.4, 105.7, 64.3, 51.5, 51.3, 38.0, 16.0

HRMS (*m/z*): $[M+H]^+$ calculated for $C_{25}H_{26}N_4OS$, 431.19001; found, 431.18991.

R_f value (SiO_2, DCM:MeOH=9:1) = 0.4

Synthesis of 7

Reaction scheme:

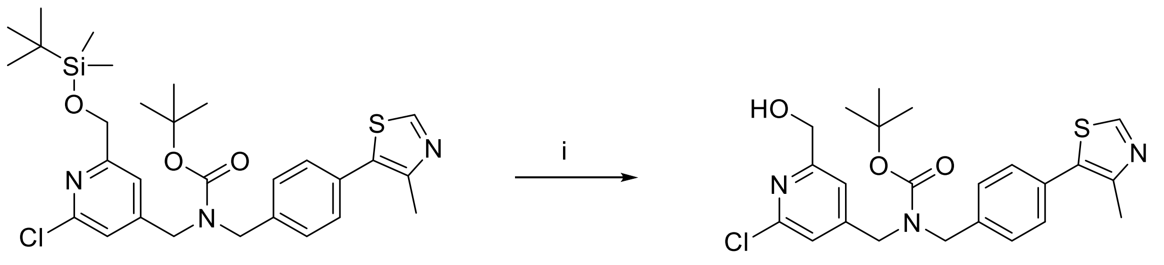

7

i) 1.0 M TBAF in THF, THF, 0 °C

tert-butyl ((2-chloro-6-(hydroxymethyl)pyridin-4-yl)methyl)(4-(4-methylthiazol-5-yl)benzyl)carbamate (7):

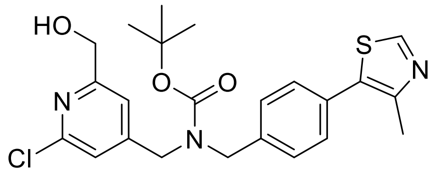

To a stirred solution of tert-butyl N-[[2-[[tert-butyl(dimethyl)silyl]oxymethyl]-6-chloro-4-pyridyl]methyl]-N-[[4-(4-methylthiazol-5-yl)phenyl]methyl]carbamate (0.24 g, 0.42 mmol) in THF (5 mL), 1.0 M TBAF in THF (0.33 g, 1.04 mmol) was added dropwise at 0°C and the reaction continued for 4 h at the same temperature. Upon completion of the reaction, it was quenched with ice cold water (25 mL) and extracted with EtOAc (2*50 mL). The organic layer was washed with water (50 mL), dried over sodium sulphate, filtered, and concentrated. The resultant crude compound was purified by column chromatography (40-100% EtOAc/petroleum ether) to give tert-butyl N-[[2-chloro-6-(hydroxymethyl)-4-pyridyl]methyl]-N-[[4-(4-methylthiazol-5-yl)phenyl]methyl]carbamate **7** (0.15 g, 0.32 mmol, 77% yield).

^{1}H NMR (DMSO-d$_6$, 400 MHz) 8.99 (s, 1H), 7.46 (d, 2H, *J*=8.1 Hz), 7.34 (br d, 3H, *J*=10.9 Hz), 7.06 (br s, 1H), 5.55 (t, 1H, *J*=5.8 Hz), 4.49 (br d, 6H, *J*=5.8 Hz), 2.4-2.5 (m, 3H), 1.39 (br d, 9H, *J*=22.8 Hz)

^{13}C NMR (DMSO-d$_6$, 100 MHz) 163.3, 154.9, 151.6, 149.2, 147.9, 137.9, 130.9, 130.4, 129.0, 128.1, 120.6, 120.2, 117.4, 79.8, 63.4, 50.1, 49.1, 27.9, 15.9

HRMS (*m/z*): [M+H]$^+$ calculated for $C_{23}H_{26}ClN_3O_3S$, 460.14562; found, 460.1459.

R_f value (SiO_2, DCM:MeOH=14:1) = 0.6

Synthesis of 8

Reaction scheme:

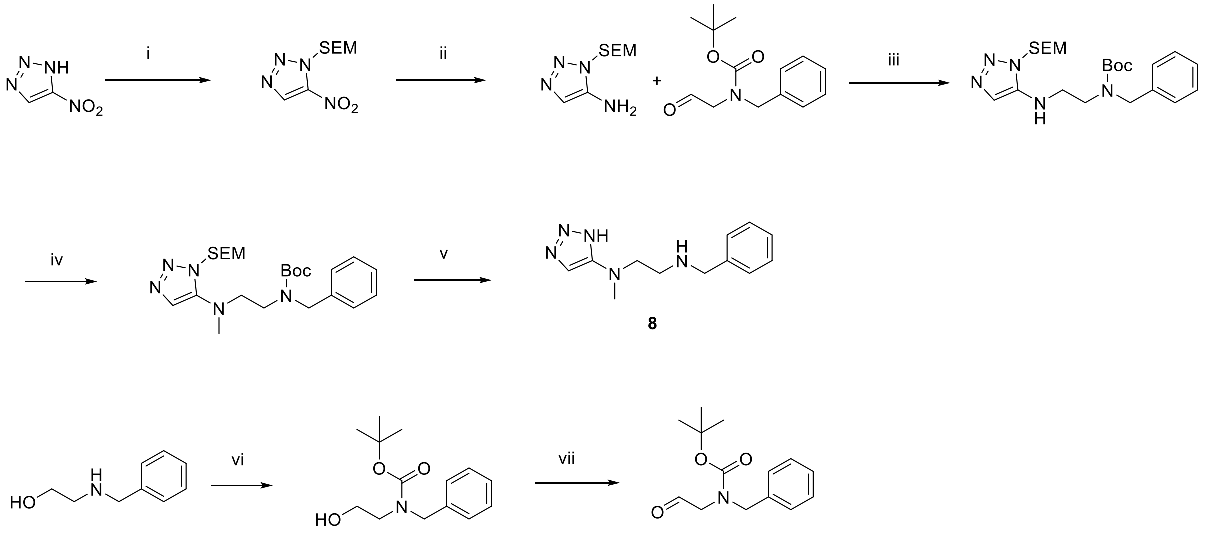

i) SEM-Cl, NaH THF, 0 °C-RT; ii) Fe, NH_4Cl, EtOH, Water, 90 °C; iii) STAB, DCM, 60 °C; iv) MeI, Cs2CO3, DMF; v) TFA, DCM; vi) Boc anhydride, THF; vii) Diacetoxyiodobenzene, TEMPO, DCM, RT

N1-benzyl-N2-methyl-N2-(1H-1,2,3-triazol-5-yl)ethane-1,2-diamine (8):

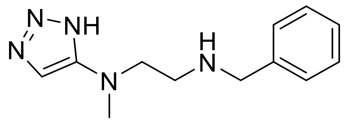

To a stirred solution of 5-nitro-1H-triazole (1.00 g, 8.77 mmol) in THF (15 mL), sodium hydride (0.32 g, 13.2 mmol) was added portion wise at 0°C and stirred for 10 min. Then 2-(chloromethoxy)ethyl-trimethyl-silane (1.61 g, 9.64 mmol) was added dropwise at 0°C and the reaction continued at RT for 5 h. Upon completion of the reaction, it was quenched with ice cold water (100 mL) and extracted with EtOAc (100 mL). The organic layer was dried over sodium sulphate, filtered, and concentrated. The resultant crude product was purified by column chromatography (0-50% EtOAc/petroleum ether) to give trimethyl-[2-[(5-nitrotriazol-1-yl)methoxy]ethyl]silane (1.40 g, 62% yield), which was directly used in the next step.

To a stirred solution of trimethyl-[2-[(5-nitrotriazol-1-yl)methoxy]ethyl]silane (0.80 g, 3.27 mmol) in Ethanol (15 mL) and Water (5 mL), Ammonium chloride (0.88 g, 16.4 mmol) and Iron powder (0.91 g, 16.4 mmol) were added at RT and the reaction was continued at 90°C for 2 h. Upon completion of the reaction, it was cooled to RT, diluted with EtOAc (100 mL), filtered, and concentrated. The resultant crude solid was washed with n-pentane and dried under reduced pressure to give 3-(2-trimethylsilylethoxymethyl)triazol-4-amine (0.60 g, 80% yield), which was directly used in the reductive amination step.

tert-butyl benzyl(2-oxoethyl)carbamate:

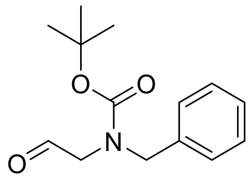

To a stirred solution of 2-(benzylamino)ethanol (10.0 g, 66.1 mmol) in THF (200 mL), Boc anhydride (15.9 g, 72.7 mmol) was added dropwise at 0° C and the reaction continued at RT for 5 h. Upon completion of the reaction, it was concentrated under reduced pressure. The resultant residue was purified by column chromatography (0-40% EtOAc/petroleum ether) to give tert-butyl N-benzyl-N-(2-hydroxyethyl)carbamate (14.0 g, 59% yield), which was directly used in the next step.

To a stirred solution of tert-butyl N-benzyl-N-(2-hydroxyethyl)carbamate (5.0 g, 19.9 mmol) in DCM (50 mL), TEMPO (0.62 g, 3.98 mmol) and iodobenzene diacetate (7.37 g, 22.9 mmol) were added at RT and the reaction continued for 6 h. Upon completion of the reaction, the mixture was concentrated under reduced pressure. The resultant residue was purified by column chromatography (0-40% EtOAc/petroleum ether) to give tert-butyl N-benzyl-N-(2-oxoethyl)carbamate (4.0 g, 72% yield), which was directly used in the reductive amination step.

A stirred solution of 3-(2-trimethylsilylethoxymethyl)triazol-4-amine (600 mg, 2.80 mmol) and tert-butyl N-benzyl-N-(2-oxoethyl)carbamate (698 mg, 2.80 mmol) in DCM (10 mL) was heated to 50°C and sodium triacetoxyborohydride (1.48 g, 7.00 mmol) was added. The reaction was continued for 1 h at the same temperature. Upon completion of the reaction, it was cooled to RT, quenched with ice cold water (50 mL) and extracted with DCM (50 mL). The organic layer was dried over sodium sulphate, filtered, and concentrated. The obtained crude was purified by column chromatography (0-50% EtOAc/

petroleum ether) to give tert-butyl N-benzyl-N-[2-[[3-(2-trimethylsilylethoxymethyl)triazol-4-yl]amino]ethyl]carbamate (500 mg, 35% yield) which was directly used in the next step.

To a stirred solution of tert-butyl N-benzyl-N-[2-[[3-(2-trimethylsilylethoxymethyl)triazol-4-yl]amino]ethyl]carbamate (500 mg, 1.12 mmol) and Caesium carbonate (910 mg, 2.79 mmol) in DMF (6 mL), iodomethane (476 mg, 3.35 mmol) was added dropwise at RT and the reaction continued for 5 h at the same temperature. Upon completion of the reaction, it was quenched with ice cold water (50 mL) and extracted with EtOAc (75 mL). The organic layer was washed with water (50 mL), dried over sodium sulphate, filtered, and concentrated. The resultant residue was purified by column chromatography (0-40% EtOAc/petroleum ether) to give tert-butyl N-benzyl-N-[2-[methyl-[3-(2-trimethylsilylethoxymethyl)triazol-4-yl]amino]ethyl]carbamate (300 mg, 52% yield) which was directly used in the next step.

To a stirred solution of tert-butyl N-benzyl-N-[2-[methyl-[3-(2-trimethylsilylethoxymethyl)triazol-4-yl]amino]ethyl]carbamate (300 mg, 0.65 mmol) in DCM (5 mL), Trifluoroacetic acid (0.49 mL, 6.50 mmol) was added dropwise at 0° C and the reaction continued for 2h at RT. Upon completion of the reaction, the mixture was concentrated under reduced pressure. The resultant crude product was purified by prep-HPLC to give N-benzyl-N'-methyl-N'-(1H-triazol-5-yl)ethane-1,2-diamine **8** (80 mg, 0.34 mmol, 43% yield).

^{1}H NMR (DMSO-d_6, 400 MHz) δ 13.92 (br s, 1H), 8.89 (br s, 2H), 7.45 (m, 5H), 7.24 (br s, 1H), 4.20 (br s, 2H), 3.4-3.5 (m, 2H), 3.16 (br d, 2H, *J*=1.5 Hz), 2.7-2.9 (m, 3H)

^{13}C NMR (DMSO-d_6, 100 MHz) 155.6, 131.9, 130.0, 129.0, 128.7, 118.9, 50.2, 48.6, 43.8, 38.2

HRMS (*m/z*): $[M+H]^+$ calculated for $C_{12}H_{17}N_5$, 232.15567; found, 232.15605.

R_f value (SiO_2, DCM:MeOH=14:1) = 0.4

Synthesis of 9 and 10

Reaction scheme:

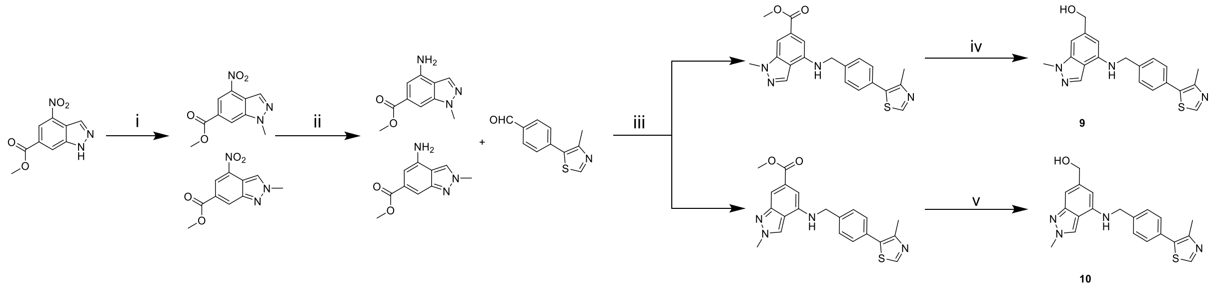

i) K_2CO_3, MeI, DMF, RT; ii) Fe, NH_4Cl, EtOH:H_2O:THF (7:2:1), 70°C; iii) STAB, AcOH, DCM, RT; iv, v) 2.0 M LAH in THF, THF -78°C-RT.

To a stirred solution of methyl 4-nitro-1H-indazole-6-carboxylate (500 mg, 2.26 mmol) in DMF (5 mL) was added K_2CO_3 (480 mg, 4.52 mmol) and Methyl iodide (0.17 mL, 2.71 mmol) at RT and the reaction was continued for 4 h. Upon completion of the reaction, the mixture was quenched with water (30 mL) and extracted with EtOAc (2*40 mL). The combined organic layers were washed with water (10 mL)

and brine solution (5 mL), dried over anhydrous Na_2SO_4, and concentrated under reduced pressure to give crude methyl 1-methyl-4-nitro-indazole-6-carboxylate (450 mg, 44% yield) and methyl 2-methyl-4-nitro-indazole-6-carboxylate (450 mg, 36% yield) as a mixture of positional isomers that was used in the next step without further purification.

To a stirred solution of a mixture of Methyl 4-amino-1-methyl-1H-indazole-6-carboxylate and methyl 4-amino-2-methyl-2H-indazole-6-carboxylate (250 mg, 1.06 mmol) in EtOH:THF:H_2O (7:2:1), was added Fe (600 mg, 10.6 mmol) and NH_4Cl (170 mg, 3.19 mmol) at RT and the reaction mixture was stirred at 70°C for 4 h. Upon completion of the reaction, it was quenched with water (20 mL) and passed through a Celite bed. The filtrate was diluted with water and extracted with EtOAc (2*30 mL). The combined organic layers were washed with brine (5 mL), dried over anhydrous Na_2SO_4, and concentrated under reduced pressure to afford methyl 4-amino-1-methyl-indazole-6-carboxylate (130 mg, 13% yield) and methyl 4-amino-2-methyl-indazole-6-carboxylate (130 mg, 15% yield) as a mixture of positional isomers that was used in the next step.

To a mixture of methyl 1-methyl-4-((4-(4-methylthiazol-5-yl) benzyl) amino)-1H-indazole-6-carboxylate and methyl 2-methyl-4-((4-(4-methylthiazol-5-yl) benzoyl) amino) indazole-6-carboxylate (130 mg, 0.63 mmol) in DCM (10 mL) were added 4-(4-methylthiazol-5-yl) benzaldehyde (140 mg, 0.70 mmol) and AcOH (0.0036 mL, 0.063 mmol). The mixture was stirred at RT for 2 h. Then Sodium triacetoxyborohydride (270 mg, 1.27 mmol) was added and stirring was continued at RT for 16 h. Upon completion of the reaction, the mixture was quenched with water (20 mL) and extracted with EtOAc (2*30 mL). The combined organic layer was washed with brine (10 mL), dried over anhydrous Na_2SO_4, and concentrated under reduced pressure. The crude residue was purified by column chromatography (30-60 % EtOAc in petroleum ether) to afford methyl 1-methyl-4-[[4-(4-methylthiazol-5-yl) benzoyl] amino] indazole-6-carboxylate (30 mg, 10% yield) and methyl 2-methyl-4-[[4-(4-methylthiazol-5-yl) benzoyl] amino] indazole-6-carboxylate (32 mg, 10% yield) that was used in the next step.

(1-methyl-4-((4-(4-methylthiazol-5-yl)benzyl)amino)-1H-indazol-6-yl)methanol (9):

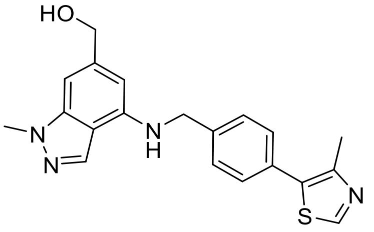

2M LAH in THF (0.38 mL, 0.76 mmol) was added dropwise to a stirred solution of methyl 1-methyl-4-[[4-(4-methylthiazol-5-yl) phenyl] methylamino] indazole-6-carboxylate (100 mg, 0.26 mmol) in THF (10 mL) at -78°C. After 10 min the temperature was slowly raised to RT and stirred for 4 h. Upon completion of the reaction, the mixture was quenched with sat. NH_4Cl solution (10 mL) and extracted with EtOAc (2*50 mL). The combined organic layer was washed with brine (5 mL), dried over anhydrous Na_2SO_4, and concentrated under reduced pressure. The crude residue was purified by column chromatography (2-4% MeOH in DCM) to afford [1-methyl-4-[[4-(4-methylthiazol-5-yl) phenyl] methyl amino] indazol-6-yl] methanol **9** (70 mg, 0.19 mmol, 70% yield).

^{1}H NMR (DMSO-d_6, 500 MHz) δ 8.98 (s, 1H), 8.11 (s, 1H), 7.5-7.5 (m, 2H), 7.4-7.5 (m, 2H), 6.90 (t, 1H, *J*=6.0 Hz), 6.68 (s, 1H), 6.04 (s, 1H), 5.07 (t, 1H, *J*=5.8 Hz), 4.47 (d, 2H, *J*=6.0 Hz), 4.45 (d, 2H, *J*=5.7 Hz), 3.90 (s, 3H), 2.45 (s, 3H)

^{13}C NMR (DMSO-d_6, 125 MHz) δ 150.9 (HSQC), 147.7, 142.9, 141.5, 141.1, 140.1, 131.1, 130.5, 129.8, 128.8, 127.7, 113.2, 96.9, 94.4, 63.7, 45.8, 35.2, 16.0

HRMS (*m/z*): $[M+H]^+$ calculated for $C_{20}H_{20}N_4OS$, 365.14306; found, 365.14334.

R_f value (SiO_2, DCM:MeOH=14:1) = 0.3

(2-methyl-4-((4-(4-methylthiazol-5-yl) benzyl) amino)-2H-indazol-6-yl) methanol (10):

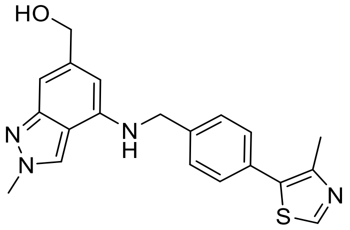

2M LAH in THF (0.38 mL, 0.76 mmol) was added dropwise to a stirred solution of methyl 2-methyl-4-[[4-(4-methylthiazol-5-yl) phenyl] methyl amino] indazole-6-carboxylate (150 mg, 0.38 mmol) in THF (5 mL) at -78 °C. After 10 min the temperature was slowly raised to RT and stirred for 4 h. Upon completion of the reaction, the mixture was quenched with sat. NH_4Cl solution (10 mL) and extracted with EtOAc (2*50 mL). The combined organic layer was washed with brine, dried over anhydrous Na_2SO_4, and concentrated under reduced pressure. The crude residue was purified by column chromatography (2-4% MeOH in DCM) to afford [2-methyl-4-[[4-(4-methylthiazol-5-yl) phenyl] methyl amino] indazol-6-yl] methanol **10** (90 mg, 0.24 mmol, 58% yield).

^1H NMR (DMSO-d_6, 400 MHz) δ 8.97 (s, 1H), 8.28 (s, 1H), 7.47 (d, 4H, *J*=6.1 Hz), 6.70 (s, 1H), 6.6-6.7 (m, 1H), 5.85 (s, 1H), 4.95 (t, 1H, *J*=5.8 Hz), 4.44 (d, 2H, *J*=5.8 Hz), 4.36 (d, 2H, *J*=5.8 Hz), 4.07 (s, 3H), 2.4-2.5 (m, 3H).

^{13}C NMR (DMSO-d_6, 100 MHz) δ 151.4, 149.5, 147.7, 141.5, 140.9, 140.1, 131.1, 129.8, 128.8, 127.7, 122.8, 113.8, 101.3, 95.6, 64.0, 45.9, 16.0, 1C under DMSO

HRMS (*m/z*): $[M+H]^+$ calculated for $C_{20}H_{20}N_4OS$, 365.14306; found, 365.14304.

R_f value (SiO_2, DCM:MeOH=9:1) = 0.5

Synthesis of 11 and 12
Reaction scheme:

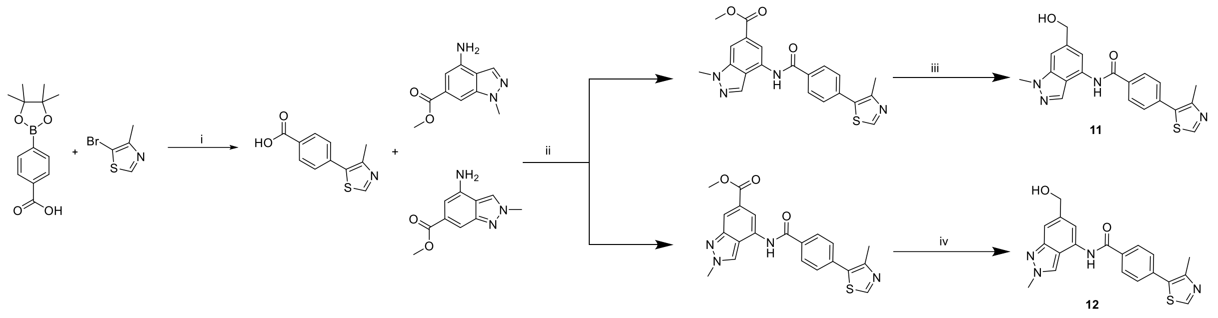

i) $Pd(PPh_3)_4$, K_3PO_4, dioxane:H_2O (8:2), 100 °C; ii) HATU, HOAt, DIPEA, DMF, RT; iii, iv) 2.0 M LAH in THF, THF, -78°C-RT

To a suspension of 4-(4,4,5,5-tetramethyl-1,3,2-dioxaborolan-2-yl) benzoic acid (1.0 g, 4.03 mmol) and 5-bromo-4-methyl-thiazole (1.08 g, 6.05 mmol) in dioxane:water (7 mL:3 mL), was added Caesium carbonate (3.94 g, 12.1 mmol) and Pd(dppf)Cl_2·DCM (160 mg, 0.20 mmol) at RT under inert conditions. The reaction mixture was stirred at 100°C for 16 h. Upon completion of the reaction, the mixture was diluted with water (20 mL), acidified with 2M HCl (13 mL), and extracted with EtOAc (2*60 mL). The combined organic layer was washed with brine, dried over anhydrous Na_2SO_4, and concentrated under reduced pressure. The crude residue was purified by column chromatography (2-6% MeOH in DCM) to afford 4-(4-methylthiazol-5-yl) benzoic acid (510 mg, 50% yield) that was used directly in the next step. To a stirred solution of 4-(4-methylthiazol-5-yl) benzoic acid (450 mg, 2.05 mmol) in DMF (10 mL) was added DIPEA (1.1 mL, 6.16 mmol), HATU (1.17 g, 3.08 mmol) and HOAt (420 mg, 3.08 mmol) at RT. After 5 minutes, a mixture of methyl 4-amino-2-methyl-indazole-6-carboxylate (250 mg, 1.23 mmol) and methyl 4-amino-1-methyl-indazole-6-carboxylate (250 mg, 1.23 mmol) was added at RT. The reaction mixture was stirred for 16 h. Upon completion of the reaction, the mixture was quenched with water (20 mL) and extracted with EtOAc (2*60 mL). The combined organic layer was washed with brine, dried over anhydrous Na_2SO_4, and concentrated under reduced pressure to get the crude mixture of products. The crude residue was purified by column chromatography (30-60% EtOAc in petroleum ether) to afford methyl 1-methyl-4-[[4-(4-methylthiazol-5-yl) benzoyl] amino] indazole-6-carboxylate (120 mg, 13% yield) and methyl 2-methyl-4-[[4-(4-methylthiazol-5-yl) benzoyl] amino] indazole-6-carboxylate (181 mg, 21% yield); the batches of which were directly subjected to the reduction step.

N-(6-(hydroxymethyl)-1-methyl-1H-indazol-4-yl)-4-(4-methylthiazol-5-yl) benzamide (11):

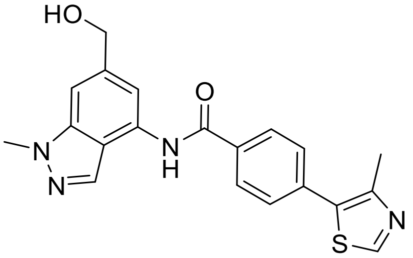

To a stirred solution of methyl 1-methyl-4-[[4-(4-methylthiazol-5-yl) benzoyl] amino] indazole-6-carboxylate (70 mg, 0.172 mmol) in THF (5 mL) was added Lithium aluminium hydride 2.0 M in THF (0.26 mL, 0.52 mmol) at 0°C and the reaction mixture was stirred at RT for 4 h. Upon completion of the reaction, the reaction mixture was quenched with sat. NH_4Cl solution (8 mL) and extracted with EtOAc (2*50 mL). The combined organic layer was washed with brine, dried over anhydrous Na_2SO_4, and concentrated under reduced pressure. The crude residue was purified by column chromatography (2-4% MeOH in DCM) to afford N-[6-(hydroxymethyl)-1-methyl-indazol-4-yl]-4-(4-methylthiazol-5-yl) benzamide **11** (25 mg, 0.06 mmol, 37% yield).

^{1}H NMR (DMSO-d_6, 500 MHz) δ 10.49 (s, 1H), 9.08 (s, 1H), 8.15 (s, 1H), 8.10 (d, 2H, *J*=8.2 Hz), 7.68 (d, 2H, *J*=8.2 Hz), 7.55 (s, 1H), 7.35 (s, 1H), 5.36 (br t, 1H, *J*=5.4 Hz), 4.65 (br d, 2H, *J*=4.7 Hz), 4.02 (s, 3H), 2.53 (s, 3H)

^{13}C NMR (DMSO-d_6, 125 MHz) δ 165.2, 152.4, 148.8, 141.7, 140.8, 134.7, 134.0, 131.6, 130.8, 130.4, 128.8, 128.6, 116.7, 111.7, 102.9, 63.2, 35.3, 16.1

HRMS (m/z): $[M+H]^+$ calculated for $C_{20}H_{18}N_4O_2S$, 379.12232; found, 379.12256.

R_f value (SiO_2, DCM:MeOH=14:1) = 0.2

N-(6-(hydroxymethyl)-2-methyl-2H-indazol-4-yl)-4-(4-methylthiazol-5-yl) benzamide (12):

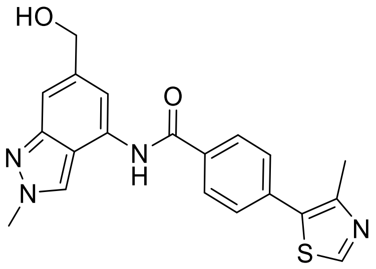

To a stirred solution of methyl 2-methyl-4-[[4-(4-methylthiazol-5-yl) benzoyl] amino] indazole-6-carboxylate (100 mg, 0.246 mmol) in THF (5mL) was added Lithium aluminium hydride 2.0 M in THF (0.37 mL, 0.74 mmol) at 0°C and the reaction mixture was stirred at RT for 4 h. Upon completion of the reaction, the mixture was quenched with sat. NH_4Cl solution (8 mL) and extracted with EtOAc (2*50 mL). The combined organic layer was washed with brine, dried over anhydrous Na_2SO_4, and concentrated under reduced pressure. The crude residue was purified by column chromatography (2-4% MeOH in DCM) to afford N-[6-(hydroxymethyl)-2-methyl-indazol-4-yl]-4-(4-methylthiazol-5-yl) benzamide **12** (46 mg, 0.12 mmol, 49% yield).

^{1}H NMR (DMSO-d$_6$, 400 MHz) δ 10.38 (s, 1H), 9.08 (s, 1H), 8.36 (s, 1H), 8.09 (d, 2H, J=8.4 Hz), 7.68 (d, 2H, J=8.4 Hz), 7.38 (s, 1H), 7.32 (s, 1H), 5.24 (t, 1H, J=5.7 Hz), 4.56 (d, 2H, J=5.6 Hz), 4.14 (s, 3H), 2.5-2.5 (m, 3H)

^{13}C NMR (DMSO-d$_6$, 100 MHz) δ 164.9, 152.4, 149.1, 148.8, 140.2, 134.6, 134.0, 130.4, 130.4, 128.7, 128.5, 124.4, 116.0, 112.2, 109.8, 63.4, 16.1, 1C under DMSO

HRMS (m/z): $[M+H]^+$ calculated for $C_{20}H_{18}N_4O_2S$, 379.12232; found, 379.12234.

R_f value (SiO_2, DCM:MeOH=14:1) = 0.4

Synthesis of 13
Reaction scheme:

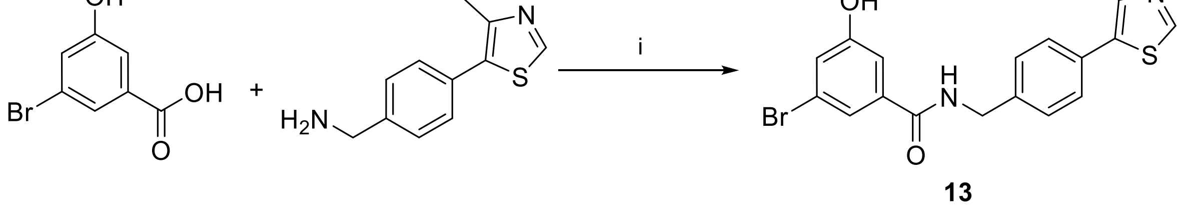

13

i) HATU, HOAt, DIPEA, DMF, RT

3-bromo-5-hydroxy-N-(4-(4-methylthiazol-5-yl) benzyl) benzamide (13):

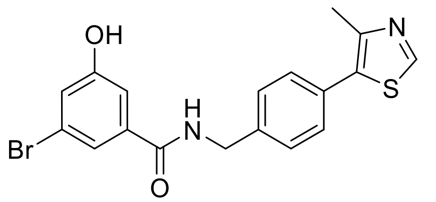

To a stirred solution of 3-bromo-5-hydroxy-benzoic acid (1.00 g, 4.61 mmol) in DMF (10 mL) were added DIPEA (2.4 mL, 13.8 mmol), HATU (2.63 g, 6.91 mmol), HOAt (940 mg, 6.91 mmol) and [4-(4-methylthiazol-5-yl) phenyl] methanamine (1.04 g, 5.07 mmol) and the reaction mixture was stirred at RT for 16 h. Upon completion of the reaction, the mixture was quenched with water (50 mL) and extracted with EtOAc (2*75 mL). The combined organic layer was washed with brine, dried over anhydrous Na_2SO_4, and concentrated under reduced pressure. The crude residue was purified by column chromatography (4-6% MeOH in DCM) to afford 3-bromo-5-hydroxy-N-[[4-(4-methylthiazol-5-yl) phenyl] methyl] benzamide **13** (640 mg, 1.58 mmol, 34% yield).

^{1}H NMR (DMSO-d$_6$, 400 MHz) δ 10.18 (s, 1H), 9.11 (s, 1H), 8.98 (s, 1H), 7.51 (s, 1H), 7.4-7.5 (m, 2H), 7.41 (s, 2H), 7.3-7.3 (m, 1H), 7.10 (t, 1H, *J*=1.9 Hz), 4.48 (d, 2H, *J*=5.8 Hz), 2.45 (m, 3H)

^{13}C NMR (DMSO-d$_6$, 100 MHz) δ 164.7, 158.5, 151.5, 147.8, 139.3, 137.3, 131.0, 129.9, 128.9, 127.8, 121.8, 120.7, 120.4, 113.8, 42.4, 15.9

HRMS (*m/z*): $[M+H]^+$ calculated for $C_{18}H_{15}BrN_2O_2S$, 403.01104; found, 403.01144.

R_f value (SiO_2, DCM:MeOH=14:1) = 0.3

Synthesis of 14
Reaction scheme:

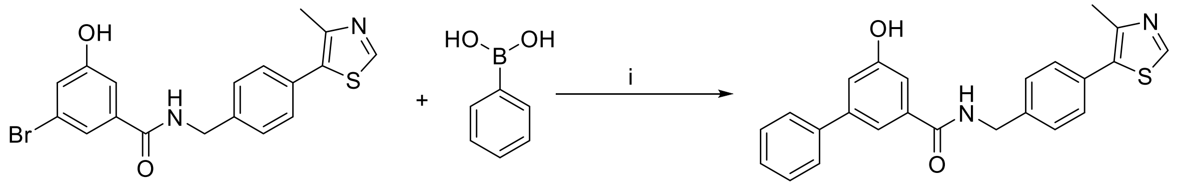

i) $Pd(PPh_3)_4$, K_3PO_4, dioxane:H_2O (8:2), 100°C

5-hydroxy-N-(4-(4-methylthiazol-5-yl)benzyl)-[1,1'-biphenyl]-3-carboxamide (14):

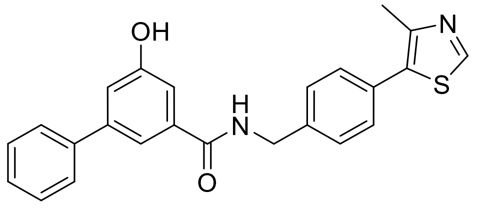

To a suspension of 3-bromo-5-hydroxy-N-[[4-(4-methylthiazol-5-yl) phenyl] methyl] benzamide (0.10 g, 0.25 mmol) and phenylboronic acid (33 mg, 0.27 mmol) in dioxane/water (10 mL), K_3PO_4 (160 mg, 0.74 mmol) and tetrakis(triphenylphosphine)palladium (2.9 mg, 0.0025 mmol) were added at RT under inert conditions. The reaction mixture was stirred at 100°C for 16 h. Upon completion of the reaction, the reaction mixture was quenched with water (30 mL) and extracted with EtOAc (2*50 mL). The

combined organic layer was washed with brine, dried over anhydrous Na_2SO_4, and concentrated under reduced pressure. The crude residue was purified by column chromatography (2-4% MeOH in DCM) to afford 3-hydroxy-N-[[4-(4-methylthiazol-5-yl) phenyl] methyl]-5-phenyl-benzamide **14** (40 mg, 0.09 mmol, 40% yield).

^{1}H NMR (DMSO-d$_6$, 400 MHz) δ 9.88 (br s, 1H), 9.13 (s, 1H), 8.99 (s, 1H), 7.6-7.7 (m, 3H), 7.47 (t, 7H, *J*=8.0 Hz), 7.32 (s, 1H), 7.19 (s, 1H), 4.53 (d, 2H, *J*=5.8 Hz), 2.4-2.5 (m, 3H)

^{13}C NMR (DMSO-d$_6$, 100 MHz) δ 166.1, 157.9, 151.5, 147.8, 141.6, 139.7, 139.6, 136.2, 131.1, 129.9, 128.9, 127.8, 127.7, 126.7, 120.6, 116.2, 113.6, 42.3, 15.9

HRMS (*m/z*): $[M+H]^+$ calculated for $C_{24}H_{20}N_2O_2S$, 401.13183; found, 401.13184.

R_f value (SiO_2, DCM:MeOH=14:1) = 0.6

Synthesis of 15

Reaction scheme

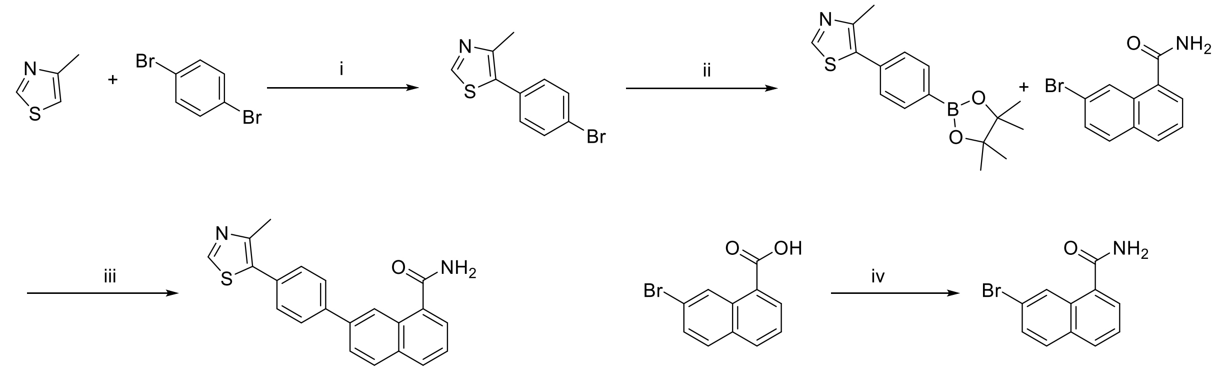

15

i) $Pd(OAc)_2$, KOAc, DMA, 100° C; ii) Bis(pinacolato)diboron, KOAc, $PdCl_2$(dppf)DCM, dioxane, 90°C; iii) Na_2CO_3, dioxane/H_2O (7:3), $PdCl_2$(dppf), 80° C; iv) $SOCl_2$, THF, NH_4OH, 80°C.

7-(4-(4-methylthiazol-5-yl)phenyl)-1-naphthamide (15)

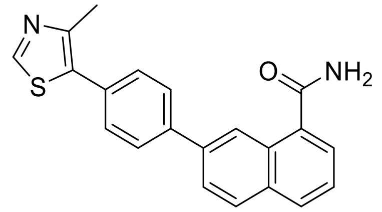

To a stirred solution of 4-methylthiazole (500 mg, 5.04 mmol) in DMA (10 mL) were added 1,4-dibromobenzene (1.18 g, 5.04 mmol), Potassium acetate (1.60 g, 10.08 mmol) and palladium (II) acetate (0.121 g, 0.54 mmol) at RT under inert conditions. The reaction mixture was stirred at 100°C for 4 h. Upon completion of the reaction, the reaction mixture was diluted with water (50 mL) and extracted with EtOAc (2*30 mL). The combined organic layer was washed with brine, dried over anhydrous Na_2SO_4, and concentrated under reduced pressure. The crude residue was purified by column chromatography (15-30% EtOAc in petroleum ether) to give 5-(4-bromophenyl)-4-methyl-thiazole (252 mg, 20% yield) which was directly used in the next step.

To a stirred solution of 5-(4-bromophenyl)-4-methyl-thiazole (150 mg, 0.59 mmol) in 1,4-Dioxane (5 mL) was added 4,4,5,5-tetramethyl-2-(4,4,5,5-tetramethyl-1,3,2-dioxaborolan-2-yl)-1,3,2-dioxaborolane (179 mg, 0.71 mmol) and Potassium acetate (115 mg, 1.18 mmol) and $PdCl_2$(dppf).DCM (8.0 mg, 0.0098 mmol) were added at RT under inert conditions. The combined reaction mixture was stirred at 100°C for 8 h. Upon completion of the reaction, the solvent was evaporated under reduced pressure. The crude residue was purified by column chromatography (15-30% EtOAc in petroleum ether) to afford 4-methyl-5-[4-(4,4,5,5-tetramethyl-1,3,2-dioxaborolan-2-yl)phenyl]thiazole (46 mg, 25% yield) which was directly used in the next step.
To a stirred solution of 7-bromonaphthalene-1-carboxylic acid (100 mg, 0.4 mmol) was added thionyl chloride (0.17 mL, 2.41 mmol) at room temperature. The mixture was stirred at 80°C for 4 h. Upon completion of the chlorination, it was concentrated under reduced pressure to remove the excess of thionyl chloride. The crude residue was dissolved in THF (5 mL) and aqueous ammonia (3 mL) was added dropwise at 0°C. The reaction was allowed to stir at RT for 12 h. Upon completion of the reaction, the solvent was evaporated. It was diluted with water (10 mL) and extracted with DCM (2*10 mL). The combined organic layer was dried over anhydrous Na_2SO_4 and concentrated under pressure to afford 7-bromonaphthalene-1-carboxamide (50 mg, 51% yield) and were used without further purification.
To a stirred solution of 7-bromonaphthalene-1-carboxamide **6** (100 mg, 0.40 mmol) in 1,4-Dioxane (7 mL) and water (3 mL), was added 4-methyl-5-[4-(4,4,5,5-tetramethyl-1,3,2-dioxaborolan-2-yl) phenyl] thiazole **3** (133 mg, 0.44 mmol) and Sodium carbonate (85 mg, 0.81 mmol) at room temperature and Pd(dppf)Cl_2 (15 mg, 0.020 mmol) was added at RT under inert conditions. The reaction mixture was stirred at 100°C for 6 h. Upon completion of the reaction, the organic solvent was evaporated under reduced pressure, the residue was diluted with water (5 mL) and extracted with DCM (2*10 mL). The combined organic layer was dried over anhydrous Na_2SO_4, concentrated under reduced pressure, and purified by prep. HPLC to give **15** (98 mg, 0.28 mmol, 71% yield).

^{1}H NMR (DMSO-d$_6$, 500 MHz) δ 9.05 (s, 1H), 8.63 (s, 1H), 8.0-8.2 (m, 3H), 7.94 (br d, 1H, *J*=8.5 Hz), 7.86 (br d, 3H, *J*=7.9 Hz), 7.4-7.7 (m, 7H)

^{13}C NMR (DMSO-d$_6$, 100 MHz) δ 170.4, 148.2, 139.6, 137.3, 134.7, 132.5, 130.8, 130.8, 129.9, 129.6, 129.6, 129.1, 127.5, 125.9, 125.3, 125.1, 123.2, 16.1

HRMS (*m/z*): $[M+H]^+$ calculated for $C_{21}H_{16}N_2O_2S$, 345.10561; found, 345.1055.

R_f value (SiO_2, DCM:MeOH=14:1) = 0.2

Synthesis of 16
Reaction scheme

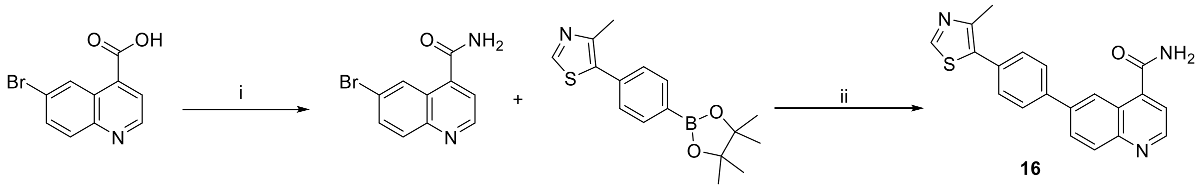

16

i) $SOCl_2$, THF, NH_4OH, 80°C; ii) Bis(pinacolato)diboron, KOAc, PdCl2(dppf).DCM, Dioxane, 90°C; iii) Na_2CO_3, dioxane/H_2O (7:3), $PdCl_2$(dppf), 80°C; iv) $SOCl_2$, THF, NH_4OH, 80°C.

6-(4-(4-methylthiazol-5-yl)phenyl)quinoline-4-carboxamide (16)

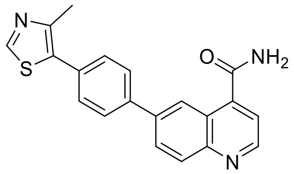

To a stirred solution of 6-bromoquinoline-4-carboxylic acid (60 mg, 0.18 mmol) was added thionyl chloride (0.14 mL, 1.98 mmol) at room temperature. The mixture was stirred at 80°C for 4 h. Upon completion of the chlorination, it was concentrated under reduced pressure to remove the excess of thionyl chloride. The crude residue was dissolved in THF (7 mL) and aqueous ammonia (3 mL) was added dropwise at 0°C. The reaction was allowed to stir at RT for 12 h. Upon completion of the reaction, the solvent was evaporated. It was diluted with water (10 mL) and extracted with DCM (2*10 mL). The combined organic layer was dried over anhydrous Na_2SO_4 and concentrated under reduced pressure to afford 6-bromoquinoline-4-carboxamide (60 mg, 46% yield) which was directly used in the next step without further purification.

To a stirred solution of 6-bromoquinoline-4-carboxamide (100 mg, 0.39 mmol) in 1,4-Dioxane (5 mL) and water (1 mL), was added 4-methyl-5-[4-(4,4,5,5-tetramethyl-1,3,2-dioxaborolan-2-yl) phenyl] thiazole (140 mg, 0.47 mmol) and Sodium carbonate (65 mg 0.71 mmol) and Pd(dppf)Cl_2 (14 mg, 0.019 mmol) was added at RT under inert conditions. The reaction mixture was stirred at 100°C for 6 h. Upon completion of the reaction, the organic solvent was evaporated under reduced pressure, and the residue was diluted with water (5 mL) and extracted with DCM (2*10 mL). The combined organic layer was dried over anhydrous Na_2SO_4, concentrated under reduced pressure, and purified by prep. HPLC to give **16** (87 mg, 0.25 mmol, 65% yield).

^{1}H NMR (DMSO-d$_6$, 400 MHz) δ 9.05 (s, 1H), 8.98 (d, 1H, *J*=4.1 Hz), 8.52 (s, 1H), 8.32 (br s, 1H), 8.19 (s, 2H), 7.95 (m, 1H), 7.87 (s, 2H), 7.68 (d, 2H, *J*=8.1 Hz), 7.65 (m, 1H), 2.54 (s, 3H)

^{13}C NMR (DMSO-d$_6$, 101 MHz) δ 168.5, 151.9, 150.4, 148.3, 147.5, 142.1, 138.8, 137.9, 131.2, 130.7, 130.1, 129.7, 128.7, 127.6, 124.3, 123.0, 119.6, 16.1

HRMS (*m/z*): $[M+H]^+$ calculated for $C_{20}H_{15}N_3OS$, 346.10086; found, 346.10077.

R_f value (SiO_2, DCM:MeOH=14:1) = 0.3

Synthesis of 17 and 18
Reaction scheme

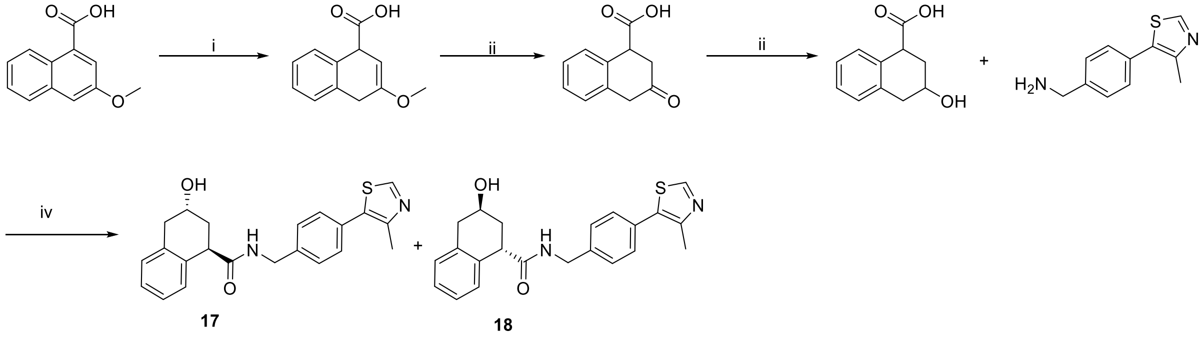

i) Na, liq. ammonia, Ethanol, -78°C; ii) Water, 2M HCl, RT; iii) $NaBH_4$, MeOH, 0°C-RT; iv) HATU, DIPEA, DMF, 0°C-RT.

(1R,3R)-3-hydroxy-N-(4-(4-methylthiazol-5-yl)benzyl)-1,2,3,4-tetrahydronaphthalene-1-carboxamide (17) and (1S,3S)-3-hydroxy-N-(4-(4-methylthiazol-5-yl)benzyl)-1,2,3,4-tetrahydronaphthalene-1-carboxamide (18)

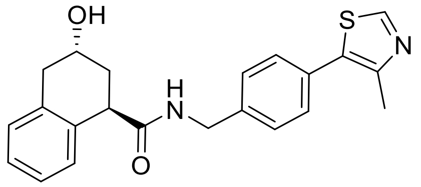

17

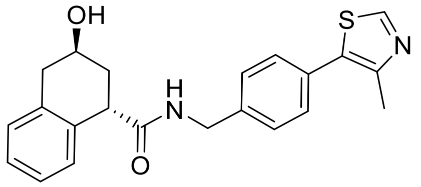

18

To a stirred solution of 3-methoxynaphthalene-1-carboxylic acid (1.50 g, 7.42 mmol) in Ethanol (15 mL) at -78°C was added liquid ammonia (52 mL, 148 mmol) and sodium metal (687 mg, 29.92 mmol) was added portion wise at -78°C over 30 minutes. Upon completion of the reaction, the mixture was allowed to reach RT and the liquid ammonia evaporated. The reaction mixture was dissolved in water (30 mL) and washed with DCM (10 mL). The aqueous layer was used for the next step without further purification (1.50 g crude).

To a stirred solution of 3-methoxy-1,4-dihydronaphthalene-1-carboxylic acid (1.50 g, 7.35 mmol) in water (20 mL) was added aq. 2M HCl (7.35 mL, 14.7 mmol) at room temperature and the mixture was stirred for 12 h. Upon completion of the reaction, it was diluted with water (20 mL) and extracted with DCM (2*50 mL). The organic layer was washed with brine, dried over anhydrous Na_2SO_4, and concentrated under reduced pressure. The residue was triturated with diethyl ether to afford 3-oxotetralin-1-carboxylic acid (407 mg, 52% yield) that was used without further purification in the next step.

To a stirred solution of 3-oxotetralin-1-carboxylic acid (110 mg, 0.58 mmol) in Methanol (5 mL) was added Sodium borohydride (43 mg, 1.15 mmol) at 0°C, the mixture was allowed to warm to room temperature and was stirred for 2 h. Upon completion of the reaction, it was quenched with excess methanol and the solvent was evaporated under reduced pressure. The crude residue was diluted with EtOAc (2 mL) and extracted with water (10 mL). The desired compound (75 mg) was obtained using a lyophilisation process of the aqueous layer and was used for the next step without further purification.

To a stirred solution of 3-hydroxytetralin-1-carboxylic acid (75 mg, 0.39 mmol) in DMF (5 mL) was added DIPEA (0.20 mL, 1.17 mmol), HATU (20 mg, 0.526 mmol), and [4-(4-methylthiazol-5-yl) phenyl] methanamine (88 mg, 0.43 mmol) at 0°C. The mixture was allowed to reach RT and was stirred for 12 h. Upon completion of the reaction, it was quenched with water (20 mL) and extracted with EtOAc (2*30 mL). The combined organic layer was washed with brine, dried over anhydrous Na_2SO_4, and concentrated under reduced pressure. The crude compound was purified by prep. HPLC to obtain a mixture of trans-isomers, which were separated by prep. SFC to give **17** (16 mg, 0.04 mmol, 11% yield) and **18** (15 mg, 0.04 mmol, 10% yield).

For **17**

^{1}H NMR (DMSO-d$_6$, 400 MHz) δ 8.99 (s, 1H), 8.68 (br t, 1H, *J*=5.7 Hz), 7.46 (s, 2H), 7.37 (d, 2H, *J*=7.9 Hz), 7.09 (br d, 4H, *J*=4.3 Hz), 4.77 (d, 1H, *J*=3.3 Hz), 4.36 (s, 2H), 4.2-4.3 (m, 1H), 3.89 (br t, 1H, *J*=6.3 Hz), 2.96 (br dd, 1H, *J*=4.1, 16.5 Hz), 2.6-2.7 (m, 1H), 2.4-2.5 (m, 3H), 2.0-2.1 (m, 1H), 1.8-1.9 (m, 1H)

^{13}C NMR (DMSO-d$_6$, 100 MHz) δ 174.2, 151.5, 147.8, 139.6, 135.4, 134.6, 131.0, 129.9, 129.4, 128.9, 127.7, 127.7, 126.2, 125.5, 62.5, 43.3, 41.9, 37.6, 34.5, 15.9

HRMS (*m/z*): $[M+H]^+$ calculated for $C_{22}H_{22}N_2O_2S$, 379.14748; found, 379.14764.

R_f value (SiO_2, DCM:MeOH=9:1) = 0.5

For **18**

^1H NMR (DMSO-d_6, 400 MHz) δ 8.99 (s, 1H), 8.68 (br t, 1H, *J*=5.8 Hz), 7.4-7.5 (m, 2H), 7.37 (d, 2H, *J*=8.1 Hz), 7.1-7.1 (m, 4H), 4.77 (d, 1H, *J*=3.5 Hz), 4.36 (t, 2H, *J*=5.6 Hz), 4.2-4.3 (m, 1H), 3.89 (t, 1H, *J*=6.6 Hz), 2.96 (dd, 1H, *J*=4.4, 16.6 Hz), 2.60 (br dd, 1H, *J*=6.2, 16.6 Hz), 2.4-2.5 (m, 3H), 2.0-2.1 (m, 1H), 1.85 (td, 1H, *J*=6.7, 13.2 Hz)

^{13}C NMR (DMSO-d_6, 101 MHz) δ 174.2, 151.5, 147.8, 139.6, 135.4, 134.6, 131.0, 129.9, 129.4, 128.9, 127.7, 127.7, 126.2, 125.5, 62.5, 43.3, 41.9, 37.6, 34.5, 15.9

HRMS (*m/z*): $[M+H]^+$ calculated for $C_{22}H_{22}N_2O_2S$, 379.14748; found, 379.14761.

R_f value (SiO_2, DCM:MeOH=9:1) = 0.4

Synthesis of 19, 20 and 21

Reaction scheme:

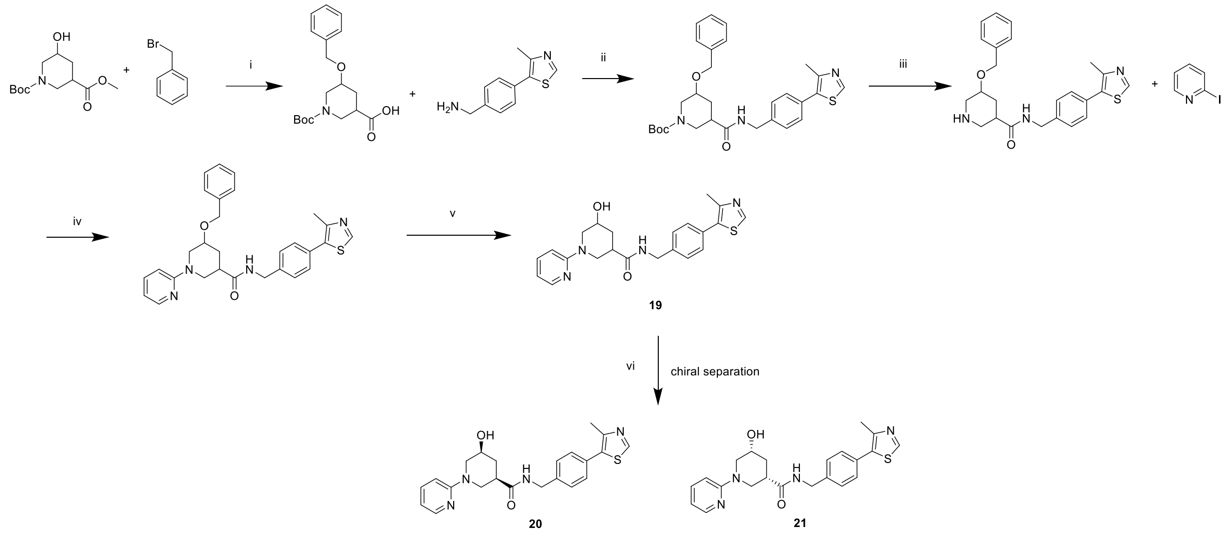

i) NaH (60%), DMF, 0°C-RT; ii) HATU, DIPEA, DMF, RT; iii) 4M HCl in dioxane, DCM; iv) Ruphos-G3, Cs_2CO_3, DME, 110°C, µw; v) BBr_3 (1.0 M in DCM), DCM, 0°C; vi) Chiral separation

5-hydroxy-N-(4-(4-methylthiazol-5-yl) benzyl)-1-(pyridin-2-yl) piperidine-3-carboxamide (19):

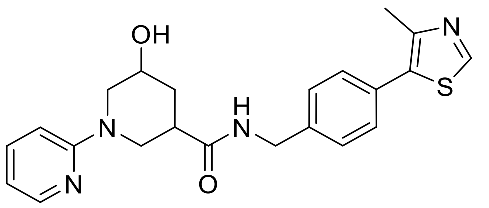

To a stirred solution of 1-tert-butyl O3-methyl 5-hydroxypiperidine-1,3-dicarboxylate (1.00 g, 3.86 mmol) in DMF (30 mL) was added NaH (0.23 g, 9.61 mmol) slowly portion wise at 0°C and the reaction mixture was stirred well for 30 min at the same temperature. Then bromomethyl benzene (1.32 g, 7.71 mmol) was added at 0°C and the reaction mixture was stirred for 2h at RT. Upon completion of the reaction, the mixture was quenched with ice cold water and extracted with EtOAc (2*30 mL). The aqueous layer was acidified to pH 5-6 with 1N HCl and extracted with EtOAc (2*15 mL). The organic layer was washed with brine, dried over Na_2SO_4, filtered, and concentrated under reduced pressure. The obtained crude (0.53 g, 30% yield) was taken to the next step without any further purification.

To a stirred solution of 5-benzyloxy-1-tert-butoxycarbonyl-piperidine-3-carboxylic acid (0.050 g, 0.15 mmol) in DMF (1 mL) was added HATU (0.085 g, 0.22 mmol) and DIPEA (0.065 mL, 0.37 mmol) at 0°C. The reaction mixture was stirred at RT for 10 min followed by addition of [4-(4-methylthiazol-5-yl) phenyl] methoxamine (0.034 g, 0.16 mmol), and the reaction was stirred for 4h at RT. Upon completion of the reaction, the mixture was quenched with ice cold water and extracted with EtOAc (2*5 mL). The organic layer was washed with brine, dried over Na_2SO_4, filtered, and concentrated under reduced pressure. The resultant residue was purified by reverse phase column chromatography (50-60% of 0.1% formic acid in water/acetonitrile) to give tert-butyl 3-(benzyloxy)-5-((4-(4-methylthiazol-5-yl) benzyl) carbamoyl) piperidine-1-carboxylate (0.050 g, 58% yield) that was directly used in the next step.
To a stirred solution of tert-butyl 3-benzyloxy-5-[[4-(4-methylthiazol-5-yl) phenyl] methyl carbamoyl] piperidine-1-carboxylate (0.050 g, 0.096 mmol) in DCM (0.5 mL) was added 4M HCl in 1,4-Dioxane (0.0070 g, 0.19 mmol) at 0°C and the reaction mixture was stirred at RT for 4 h. Upon completion of the reaction, the mixture was concentrated under reduced pressure to give crude 5-(benzyloxy)-N-(4-(4-methylthiazol-5-yl) benzyl) piperidine-3-carboxamide (0.040 g, 95% yield), which was taken to the next step without further purification.
In a 5 mL microwave vial 5-benzyloxy-N-[[4-(4-methylthiazol-5-yl) phenyl] methyl] piperidine-3-carboxamide (0.25 g, 0.59 mmol), 2-bromopyridine (0.19 g, 1.19 mmol), and caesium carbonate (0.48 g, 1.48 mmol) were taken up in DME (1 mL), and the mixture was degassed with argon for 2 min. Then RuPhos-Pd-G3 (0.050 g, 0.059 mmol) was added, and the reaction mixture was irradiated for 1 h at 110°C. The solvent was removed under reduced pressure and the crude residue was purified by reverse phase column chromatography (30-50% of 0.1% formic acid in water/acetonitrile to give 5-(benzyloxy)-N-(4-(4-methylthiazol-5-yl) benzyl)-1-(pyridin-2-yl) piperidine-3-carboxamide (0.015 g, 5% yield).
To a stirred solution of 5-benzyloxy-N-[[4-(4-methylthiazol-5-yl) phenyl] methyl]-1-(2-pyridyl) piperidine-3-carboxamide (0.13 g, 0.26 mmol) in DCM (1 mL) was added BBr_3 (1M in DCM) (0.078 g, 0.31 mmol) at -78°C and the reaction mixture was stirred for 30 min at -78°C. Upon completion of the reaction, it was quenched with sodium bicarbonate solution and extracted with 10% MeOH/DCM

(2*2 mL). The organic layer was dried over Na_2SO_4, filtered, and concentrated under reduced pressure. The resultant residue was purified by reverse phase column chromatography (20-30% of 0.1%formic acid in water/acetonitrile) to give **19** (0.025 g, 0.06 mmol, 21% yield).

^{1}H NMR (DMSO-d$_6$, 400 MHz) δ 8.99 (s, 1H), 8.50 (br t, 1H, *J*=6.0 Hz), 8.08 (dd, 1H, *J*=1.4, 4.4 Hz), 7.5-7.5 (m, 1H), 7.45 (s, 2H), 7.36 (s, 2H), 6.84 (d, 1H, *J*=8.6 Hz), 6.59 (dd, 1H, *J*=4.9, 7.0 Hz), 5.03 (d, 1H, *J*=4.8 Hz), 4.2-4.4 (m, 5H), 3.45 (br d, 1H, *J*=4.6 Hz), 2.77 (m, 1H), 2.46 (s, 3H), 2.09 (br d, 1H, *J*=12.2 Hz), 1.53 (q, 1H, *J*=12.1 Hz), 1H under DMSO

^{13}C NMR (DMSO-d$_6$, 100 MHz) δ 172.3, 158.3, 151.5, 147.8, 147.6, 139.4, 137.6, 131.0, 129.9, 128.9, 127.6, 112.5, 107.0, 64.3, 51.8, 46.8, 41.6, 40.6, 37.4, 15.9

HRMS (*m/z*): $[M+H]^+$ calculated for $C_{22}H_{24}N_4O_2S$, 409.16927; found, 409.16968.

R_f value (SiO_2, DCM:MeOH=14:1) = 0.2, 0.5

5-hydroxy-N-(4-(4-methylthiazol-5-yl) benzyl)-1-(pyridin-2-yl) piperidine-3-carboxamide (20 and 21):

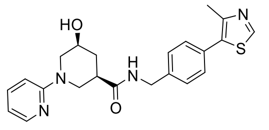

20

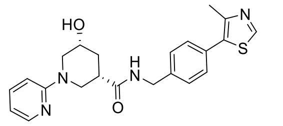

21

The mixture of isomers of 5-hydroxy-N-[[4-(4-methylthiazol-5-yl) phenyl] methyl]-1-(2-pyridyl) piperidine-3-carboxamide (0.12 g, 0.29 mmol) was subjected to chiral SFC purification to obtain two cis enantiomers.

20 analytical data:

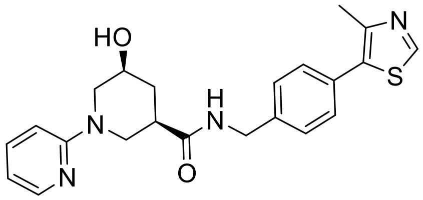

20

(3R,5S)-5-hydroxy-N-(4-(4-methylthiazol-5-yl)benzyl)-1-(pyridin-2-yl)piperidine-3-carboxamide **20** (0.017 g, 0.04 mmol, 12% yield).

^{1}H NMR (DMSO-d$_6$, 400 MHz) δ 8.99 (s, 1H), 8.50 (br t, 1H, *J*=5.8 Hz), 8.08 (br d, 1H, *J*=3.5 Hz), 7.46 (br d, 3H, *J*=8.1 Hz), 7.35 (d, 2H, *J*=8.1 Hz), 6.84 (d, 1H, *J*=8.6 Hz), 6.59 (dd, 1H, *J*=5.1, 6.8 Hz), 5.05 (br s, 1H), 4.2-4.4 (m, 5H), 3.44 (br d, 1H, *J*=4.6 Hz), 2.80 (t, 1H, *J*=12.2 Hz), 2.46 (s, 3H), 2.09 (br d, 1H, *J*=11.9 Hz), 1.53 (q, 1H, *J*=11.9 Hz), 1H under DMSO

^{13}C NMR (DMSO-d$_6$, 100 MHz) δ 172.4, 158.3, 151.5, 147.8, 147.6, 139.4, 137.6, 131.0, 129.9, 128.9, 127.7, 112.5, 107.0, 64.3, 51.8, 46.8, 41.6, 40.6, 37.4, 15.9

HRMS (*m/z*): $[M+H]^+$ calculated for $C_{22}H_{24}N_4O_2S$, 409.16927; found, 409.16962.

R_f value (SiO_2, DCM:MeOH=14:1) = 0.2

21 analytical data:

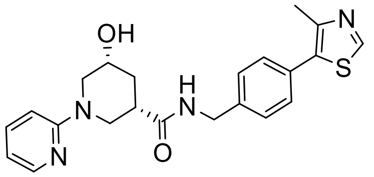

21

(3S,5R)-5-hydroxy-N-(4-(4-methylthiazol-5-yl) benzyl)-1-(pyridin-2-yl) piperidine-3-carboxamide **21** (0.018 g, 0.04 mmol, 14% yield).

^{1}H NMR (DMSO-d_6, 400 MHz) δ 8.99 (s, 1H), 8.50 (t, 1H, *J*=5.8 Hz), 8.08 (dd, 1H, *J*=1.8, 4.8 Hz), 7.5-7.5 (m, 1H), 7.46 (d, 2H, *J*=8.4 Hz), 7.35 (d, 2H, *J*=8.1 Hz), 6.84 (d, 1H, *J*=8.6 Hz), 6.59 (dd, 1H, *J*=4.9, 7.0 Hz), 5.04 (br s, 1H), 4.2-4.4 (m, 5H), 3.4-3.5 (m, 1H), 2.8-2.8 (m, 1H), 2.4-2.5 (m, 3H), 2.09 (br d, 1H, *J*=12.2 Hz), 1.5-1.6 (m, 1H)

^{13}C NMR (DMSO-d_6, 100 MHz) δ 172.4, 158.3, 151.5, 147.8, 147.6, 139.4, 137.6, 131.0, 129.9, 128.9, 127.7, 112.5, 107.0, 64.3, 51.8, 46.8, 41.6, 40.6, 37.4, 15.9

HRMS (*m/z*): $[M+H]^+$ calculated for $C_{22}H_{24}N_4O_2S$, 409.16927; found, 409.16934.

R_f value (SiO_2, DCM:MeOH=14:1) = 0.5

Synthesis of 22

Reaction scheme:

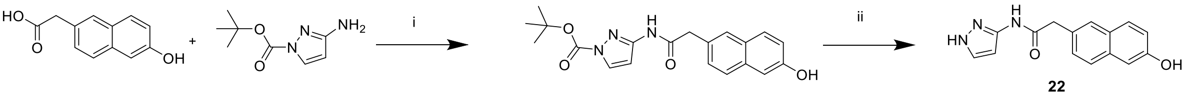

22

i) HATU, HOAt, DIPEA, DMF, rt; ii) LiOH, THF, Methanol, Water.

tert-butyl 3-(2-(6-hydroxynaphthalen-2-yl) acetamido)-1H-pyrazole-1-carboxylate (22)

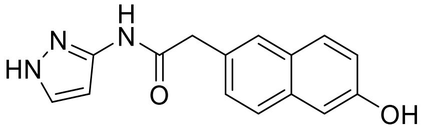

To a stirred solution of 2-(6-hydroxy-2-naphthyl) acetic acid (0.010 g, 0.050 mmol) in DMF (0.2 mL) was added HATU (0.028 g, 0.074 mmol) and DIPEA (0.026 mL, 0.15 mmol) at 0°C. The reaction mixture was stirred at RT for 10 min. Then tert-butyl 3-aminopyrazole-1-carboxylate (0.011 g, 0.059 mmol) was added, and the mixture was stirred for 4h at RT. Upon completion of the reaction, it was quenched with ice cold water and extracted with EtOAc (2*5 mL). The organic layer was washed with brine, dried over Na_2SO_4, filtered, and concentrated under reduced pressure to give crude tert-butyl 3-(2-(6-hydroxynaphthalen-2-yl) acetamido)-1H-pyrazole-1-carboxylate (0.02 g) that was directly used in the next step.

To a stirred solution of crude tert-butyl 3-(2-(6-hydroxynaphthalen-2-yl) acetamido)-1H-pyrazole-1-carboxylate (0.150 g) in THF (2 mL), Water (0.5 mL), and Methanol (1 mL) was added LiOH (0.014 g, 0.33 mmol) and the reaction mixture was stirred at RT for 1 h. Upon completion of the reaction, the

mixture was diluted with water and extracted with EtOAc (2*10 mL). The organic layer was washed with brine, dried over Na_2SO_4, filtered, and concentrated under reduced pressure. The obtained crude residue was purified by reverse phase chromatography (20-30% of 0.1% formic acid in water/acetonitrile) to give **22** (0.04 g, 0.17 mmol, 31% yield).

^{1}H NMR (DMSO-d$_6$, 500 MHz) δ 12.38 (br s, 1H), 10.62 (br s, 1H), 9.9-10.4 (m, 1H), 7.69 (d, 1H, *J*=8.5 Hz), 7.66 (s, 1H), 7.60 (d, 1H, *J*=8.5 Hz), 7.55 (br s, 1H), 7.35 (dd, 1H, *J*=1.6, 8.2 Hz), 7.1-7.1 (m, 2H), 6.45 (br s, 1H), 3.69 (s, 2H)

^{13}C NMR (DMSO-d$_6$, 125 MHz) δ 168.3, 155.3, 147.3, 133.4, 130.2, 128.8, 128.5, 127.8, 127.5, 127.2, 125.9, 118.8, 108.5, 95.8, 42.4

HRMS (*m/z*): $[M+H]^+$ calculated for $C_{15}H_{13}N_3O_2$, 231.12920; found, 231.12988.

R_f value (SiO_2, DCM:MeOH=14:1) = 0.3

Synthesis of 23, 24 and 25
Reaction scheme:

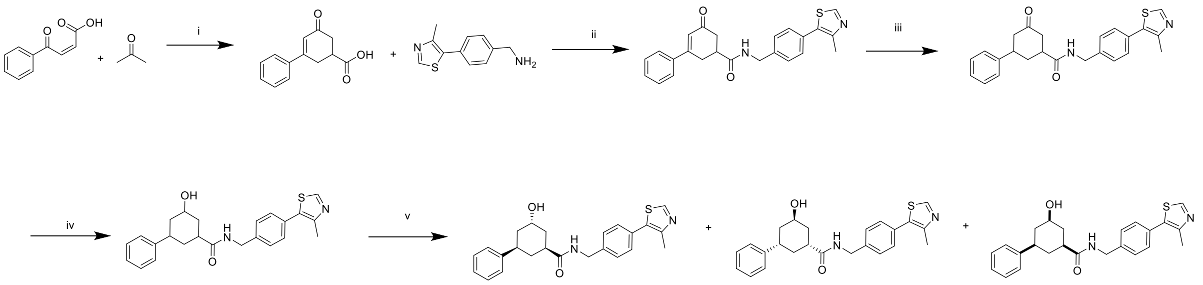

24 25 2

i) pyrrolidine, Methanol, 40°C; ii) HATU, HOAt, DIPEA, DMF, RT; iii) 10% Pd/C, Methanol, H_2 gas (70 PSI), rt; iv) L-Selectride, THF, 0°C; v) Chiral separation.

N-(4-(4-methylthiazol-5-yl) benzyl)-5-oxo-2,3,4,5-tetrahydro-[1,1'-biphenyl]-3-carboxamide:

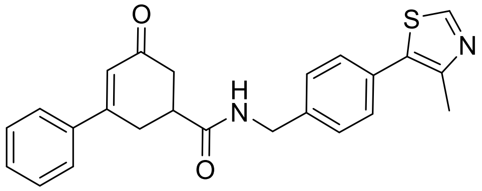

To a stirred solution of acetone (13.19 g, 227 mmol) in methanol (4 mL) was added (E)-4-oxo-4-phenyl-but-2-enoic acid (2.00 g, 11.4 mmol) and pyrrolidine (0.40 g, 5.68 mmol). The solution was heated to 40°C for 24 h. Upon completion of reaction, the solvents were removed under reduced pressure and water (50 mL) was added. It was extracted with EtOAc three times. The combined organic layer was dried over anhydrous Na_2SO_4, filtered, and concentrated under reduced pressure. The resultant residue was purified by flash chromatography (50-70% EtOAc/petroleum ether) to give 5-oxo-2,3,4,5-tetrahydro-[1,1'-biphenyl]-3-carboxylic acid (0.5 g, 17% yield) which was directly used in the next step.

To a stirred solution of 5-oxo-3-phenyl-cyclohex-3-ene-1-carboxylic acid (0.050 g, 0.23 mmol) in DMF (0.5 mL) was added HATU (0.085 g, 0.22 mmol) and DIPEA (0.065 mL, 0.373 mmol) at 0°C and the reaction mixture was stirred at RT for 10 min. Then [4-(4-methylthiazol-5-yl) phenyl] methoxamine (0.057 g, 0.28 mmol) was added and stirring was continued for 16 h at RT. Upon completion of the reaction, it was quenched with ice cold water, extracted with EtOAc (2*5 mL), dried over Na_2SO_4, filtered, and concentrated under reduced pressure. The resultant residue was purified by reverse phase column chromatography (40-50% of 0.1% formic acid in water/acetonitrile) to give N-(4-(4-methylthiazol-5-yl) benzyl)-5-oxo-2,3,4,5-tetrahydro-[1,1'-biphenyl]-3-carboxamide (0.050 g, 51% yield) which was directly used in the next step.

(cis)-3-hydroxy-N-(4-(4-methylthiazol-5-yl) benzyl)-5-phenylcyclohexane-1-carboxamide (23):

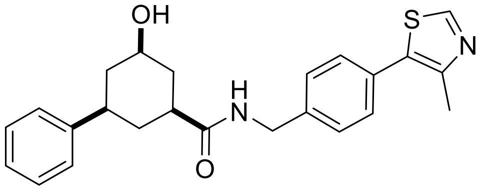

23

To a stirred solution of N-(4-(4-methylthiazol-5-yl) benzyl)-5-oxo-2,3,4,5-tetrahydro-[1,1'-biphenyl]-3-carboxamide (0.20 g, 0.50 mmol) in methanol (4 mL) was added 10% Pd/C (0.047 g, 0.45 mmol) slowly and the reaction mixture was stirred for 20 h at RT under hydrogen atmosphere (bladder). Upon completion of the reaction, the catalyst was filtered off through a Celite bed, rinsed with DCM (2*10 mL), and the filtrate was concentrated under reduced pressure to give crude N-(4-(4-methylthiazol-5-yl) benzyl)-3-oxo-5-phenylcyclohexane-1-carboxamide (0.018 g, 52% yield) that was directly used in the next step.
To a stirred solution of N-[[4-(4-methylthiazol-5-yl) phenyl] methyl]-3-oxo-5-phenyl-cyclohexane-1-carboxamide (0.10 g, 0.25 mmol) in THF (4 mL) was added L-Selectride (0.11 g, 0.371 mmol) at 0°C and the reaction was stirred for 1h at 0°C. Upon completion of the reaction, it was quenched with sat. ammonium chloride solution, extracted with EtOAc (2*5 mL), dried over sodium sulphate, and concentrated under reduced pressure. The resultant residue was purified by reverse phase column chromatography (50-60% of 0.1% formic acid in water/acetonitrile) to give **23** (0.01 g, 9% yield) as a racemic mixture and the trans-OH isomer (0.03 g, 0.07 mmol, 29% yield) as a racemic mixture.

^{1}H NMR (DMSO-d_6, 400 MHz) δ 8.98 (s, 1H), 8.36 (t, 1H, *J*=6.0 Hz), 7.44 (d, 2H, *J*=8.1 Hz), 7.1-7.4 (m, 7H), 4.74 (d, 1H, *J*=4.6 Hz), 4.30 (d, 2H, *J*=5.8 Hz), 3.60 (br d, 1H, *J*=4.3 Hz), 2.5-2.7 (m, 1H), 2.4-2.5 (m, 4H), 1.7-2.1 (m, 3H), 1.2-1.6 (m, 3H)

^{13}C NMR (DMSO-d_6, 100 MHz) δ 174.1, 151.4, 147.8, 145.9, 139.7, 131.0, 129.8, 128.8, 128.4, 127.5, 126.7, 126.0, 68.4, 42.6, 42.3, 41.5, 41.0, 38.2, 36.2, 15.9

HRMS (*m/z*): $[M+H]^+$ calculated for $C_{24}H_{26}N_2O_2S$, 407.17878; found, 407.17868.

R_f value (SiO_2, DCM:MeOH=14:1) = 0.3

3-hydroxy-N-(4-(4-methylthiazol-5-yl) benzyl)-5-phenylcyclohexane-1-carboxamide (24 and 25):

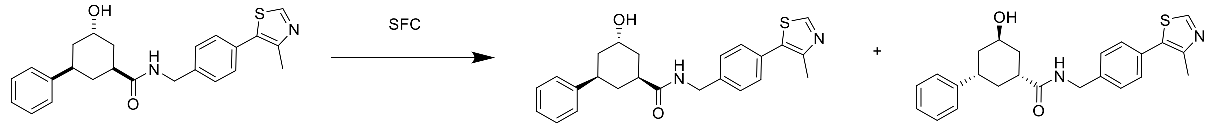

The racemic mixture of trans isomers of 3-hydroxy-N-[[4-(4-methylthiazol-5-yl) phenyl] methyl]-5-phenyl-cyclohexanecarboxamide (0.10 g, 0.25 mmol) was purified by SFC. **24** (0.035 g, 0.08 mmol 34% yield) and **25** (0.035 g, 0.08 mmol 34% yield) were obtained.

(1S,3S,5R)-3-hydroxy-N-(4-(4-methylthiazol-5-yl) benzyl)-5-phenylcyclohexane-1-carboxamide (24):

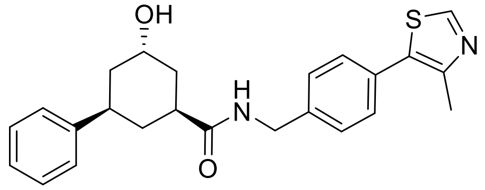

24

^{1}H NMR (DMSO-d$_6$, 500 MHz) δ 8.97 (s, 1H), 8.37 (t, 1H, *J*=6.0 Hz), 7.43 (d, 2H, *J*=8.2 Hz), 7.29 (dd, 4H, *J*=7.9, 11.3 Hz), 7.2-7.2 (m, 2H), 7.1-7.2 (m, 1H), 4.6-4.6 (m, 1H), 4.3-4.3 (m, 2H), 4.11 (br s, 1H), 3.0-3.1 (m, 1H), 2.8-2.9 (m, 1H), 2.43 (s, 3H), 1.7-1.9 (m, 3H), 1.5-1.6 (m, 3H)

^{13}C NMR (DMSO-d$_6$, 125 MHz) δ 175.1, 151.4, 147.7, 146.7, 139.8, 131.1, 129.7, 128.8, 128.3, 127.4, 126.8, 125.9, 64.4, 41.4, 38.5, 36.9, 36.5, 35.0, 15.9, 1C under DMSO

HRMS (*m/z*): [M+H]$^+$ calculated for $C_{24}H_{26}N_2O_2S$, 407.17878; found, 407.17877.

R_f value (SiO_2, DCM:MeOH=14:1) = 0.2

(1R,3R,5S)-3-hydroxy-N-(4-(4-methylthiazol-5-yl) benzyl)-5-phenylcyclohexane-1-carboxamide (25):

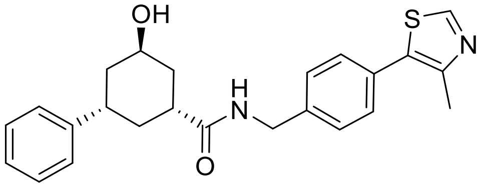

25

^{1}H NMR (DMSO-d$_6$, 400 MHz) δ 8.97 (s, 1H), 8.37 (t, 1H, *J*=5.8 Hz), 7.43 (d, 2H, *J*=7.9 Hz), 7.3-7.3 (m, 4H), 7.2-7.2 (m, 2H), 7.18 (d, 1H, *J*=7.4 Hz), 4.60 (d, 1H, *J*=2.3 Hz), 4.28 (d, 2H, *J*=6.1 Hz), 4.12 (br s, 1H), 3.02 (br t, 1H, *J*=12.5 Hz), 2.7-2.9 (m, 1H), 2.43 (s, 3H), 1.76 (br s, 3H), 1.5-1.7 (m, 3H)

^{13}C NMR (DMSO-d$_6$, 100 MHz) δ 175.1, 151.4, 147.8, 146.7, 139.8, 131.1, 129.7, 128.8, 128.3, 127.4, 126.8, 125.9, 64.4, 41.5, 38.5, 36.9, 36.5, 35.0, 15.9

HRMS (*m/z*): [M+H]$^+$ calculated for $C_{24}H_{26}N_2O_2S$, 407.17878; found, 407.17856.

R_f value (SiO_2, DCM:MeOH=14:1) = 0.2

Synthesis of 26
Reaction scheme:

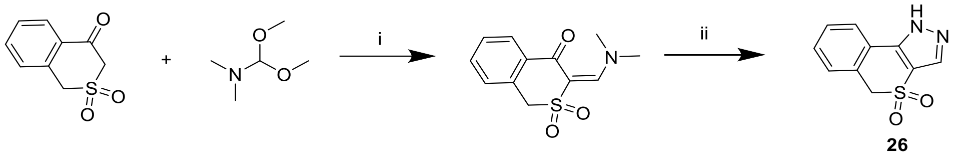

26

i) DMF-DMA, toluene, 60°C; ii) $NH_2NH_2.H_2O$, IPA, 0°C-RT.

1,5-dihydroisothiochromeno[4,3-c]pyrazole 4,4-dioxide (26):

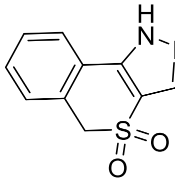

To a stirred solution of 2,2-dioxo-1H-isothiochromen-4-one (0.50 g, 2.55 mmol) in Toluene (6 mL), 1,1-dimethoxy-N, N-dimethyl-methanamine (3.4 mL, 10.2 mmol) was added slowly and the reaction mixture was stirred for 16 h at 60°C. Upon completion of the reaction, it was concentrated under reduced pressure. The resultant residue was triturated with diethyl ether to give (3E)-3-(dimethylaminomethylene)-2,2-dioxo-1H-isothiochromen-4-one (0.6 g, 74% yield) which was used in the next step without further purification.
To a stirred solution of (3E)-3-(dimethylaminomethylene)-2,2-dioxo-1H-isothiochromen-4-one (0.12 g, 0.48 mmol) in IPA (2 mL) was added 35% Hydrazine hydrate (0.023 g, 0.72 mmol) at 0°C and the reaction mixture was stirred for 16 h at RT. Upon completion of the reaction, it was concentrated under reduced pressure. The resultant residue was triturated with diethyl ether to give 1,5-dihydroisothiochromeno[4,3-c] pyrazole 4,4-dioxide **26** (0.04 g, 0.18 mmol, 37% yield).

^{1}H NMR (DMSO-d_6, 400 MHz) δ 8.25 (s, 1H), 7.84 (d, 1H, *J*=7.9 Hz), 7.39 (s, 1H), 7.3-7.4 (m, 2H), 4.60 (s, 2H), NH – missing

^{13}C NMR (DMSO-d_6, 100 MHz) δ 146.6, 131.3, 130.4, 130.4, 130.3, 130.1, 126.3, 126.2, 117.9, 57.7

HRMS (*m/z*): $[M+H]^+$ calculated for $C_{10}H_8N_2O_2S$, 221.03793; found, 221.03845.

R_f value (SiO_2, DCM:MeOH=14:1) = 0.5

Synthesis of 27
Reaction scheme:

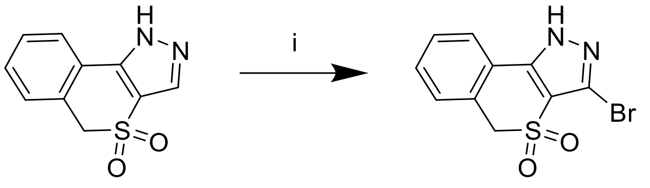

26 **27**

i) NBS, DMF, 50°C, 90 min, MW

3-bromo-1,5-dihydroisothiochromeno[4,3-c]pyrazole 4,4-dioxide (27):

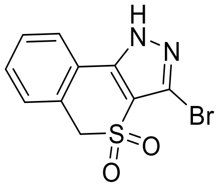

In a 5 mL microwave vial 1,5-dihydroisothiochromeno[4,3-c]pyrazole-4,4-dioxide **26** (0.15 g, 0.68 mmol) was dissolved in DMF (1.5 mL) and N-bromosuccinimide (0.30 g, 1.70 mmol) was added. The reaction mixture was irradiated at 50°C for 90 min. Upon completion of the reaction, it was diluted with ice cold water (5 mL), extracted with EtOAc (3*15 mL), washed with brine solution (2*10 mL), dried over Na_2SO_4, filtered and concentrated under reduced pressure. The crude residue was purified by reverse phase column chromatography (60-70% of 0.1% formic acid in water/acetonitrile) to give 3-bromo-1,5-dihydroisothiochromeno[4,3-c]pyrazole 4,4-dioxide **27** (0.090 g, 0.30 mmol, 44% yield).

^{1}H NMR (DMSO-d$_6$, 400 MHz) δ 12.6-15.7 (m, 1H), 7.91 (d, 1H, *J*=7.9 Hz), 7.4-7.6 (m, 3H), 4.88 (s, 2H)

^{13}C NMR (DMSO-d$_6$, 100 MHz) δ 144.3, 130.3, 130.1, 130.0, 129.0, 123.9, 123.2, 120.6, 115.4, 56.9

HRMS (*m/z*): $[M+H]^+$ calculated for $C_{10}H_7BrN_2O_2S$, 298.94844; found, 298.94843.

R_f value (SiO_2, DCM:MeOH=14:1) = 0.7

Synthesis of 28
Reaction scheme:

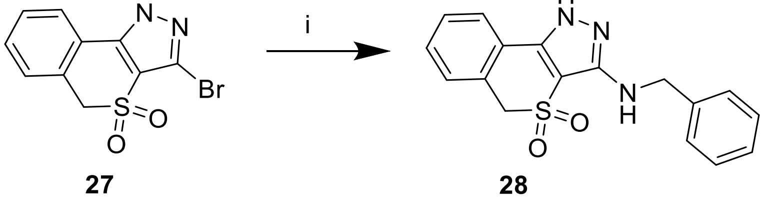

27 **28**

i) benzyl amine, NMP, 180°C, 180 min, µw

N-benzyl-4,4-dioxo-1,5-dihydroisothiochromeno[4,3-c] pyrazol-3-amine (28):

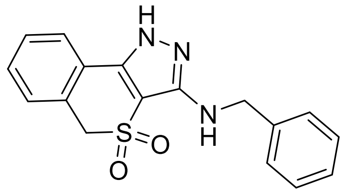

In a 30 mL microwave vial 3-bromo-1,5-dihydroisothiochromeno[4,3-c] pyrazole 4,4-dioxide **27** (0.20 g, 0.67 mmol) was dissolved in NMP (1 mL) and benzyl amine (0.14 g, 1.34 mmol) was added. The reaction mixture was irradiated at 180°C for 180 min. Upon completion of the reaction, it was diluted with water (3 mL) and extracted with EtOAc (3*5 mL), washed with brine solution (2*10 mL), dried over Na_2SO_4, and concentrated under reduced pressure. The crude residue was purified by Prep-HPLC (0.1% formic acid in water/acetonitrile) to give N-benzyl-4,4-dioxo-1,5-dihydroisothiochromeno[4,3-c]pyrazol-3-amine **28** (0.019 g, 0.05 mmol, 8% yield).

^1H NMR (DMSO-d_6, 400 MHz) δ 7.76 (s, 1H), 7.35 (m, 3H), 7.15 (m, 6H), 4.61 (s, 2H), 4.36 (s, 2H)

^{13}C NMR (DMSO-d_6, 100 MHz) δ 148.9, 145.4, 139.8, 131.2, 130.8, 130.6, 129.8, 129.2, 128.2, 128.0, 125.3, 125.1, 99.5, 58.1, 47.4

HRMS (*m/z*): $[M+H]^+$ calculated for $C_{17}H_{15}N_3O_2S$, 326.09577; found, 326.09604.

R_f value (SiO_2, DCM:MeOH=14:1) = 0.4

Synthesis of 29
Reaction scheme:

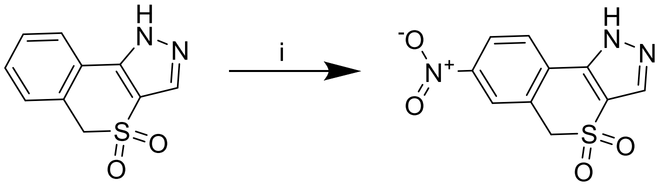

26 **29**

i) nitric acid (fuming), sulphuric acid, rt

7-nitro-1,5-dihydroisothiochromeno[4,3-c]pyrazole 4,4-dioxide (29):

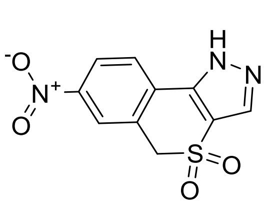

To a stirred solution of 1,5-dihydroisothiochromeno[4,3-c]pyrazole 4,4-dioxide **26** (0.10 g, 0.45 mmol) in sulfuric acid (0.80 mL), nitric acid (fuming, 98%) (0.034 g, 0.55 mmol) was added, and the reaction mixture was stirred for 1h at RT. Upon completion of the reaction, the mixture was diluted with ice water (5 mL) and basified with sodium bicarbonate to pH ~6. It was extracted with EtOAc (2*5 mL). The organic layer was washed with brine solution, dried over Na_2SO_4, filtered, and concentrated under reduced pressure. The resultant crude residue was purified by reverse phase

column chromatography (40-55% of 0.1% formic acid in water/acetonitrile) to give 7-nitro-1,5-dihydroisothiochromeno[4,3-c]pyrazole 4,4-dioxide **29** (0.008 g, 0.02 mmol, 7% yield).

^{1}H NMR (DMSO-d$_6$, 400 MHz) δ 9.0-9.2 (m, 1H), 8.98 (s, 1H), 8.46 (s, 1H), 7.69 (t, 1H, *J*=7.7 Hz), 7.44 (d, 4H, *J*=8.9 Hz), 7.28 (d, 1H, *J*=7.1 Hz), 6.75 (br d, 1H, *J*=8.6 Hz), 4.51 (d, 2H, *J*=6.1 Hz), 2.45(m, 3H)

^{13}C NMR (DMSO-d$_6$, 100 MHz) δ 165.8, 162.9, 151.5, 147.8, 145.0, 140.2, 139.1, 131.0, 130.0, 128.9, 128.0, 116.5, 110.7, 42.2, 15.9

HRMS (*m/z*): $[M+H]^+$ calculated for $C_{17}H_{15}N_3O_2S$, 326.09577; found, 326.09616.

R_f value (SiO_2, DCM:MeOH=14:1) = 0.3

Synthesis of 30
Reaction scheme:

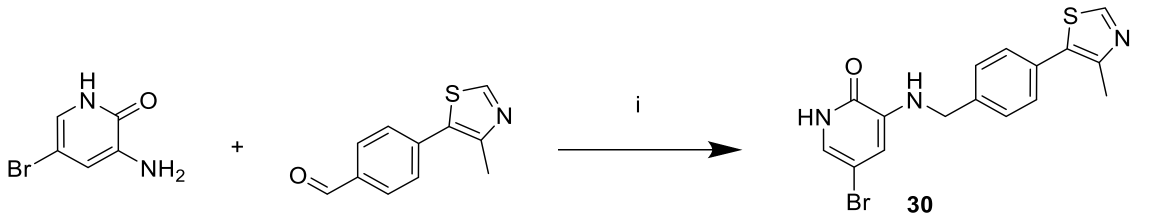

i) $NaCNBH_3$, Acetic acid, Methanol, DCM, rt

5-bromo-3-((4-(4-methylthiazol-5-yl)benzyl)amino)pyridin-2(1H)-one (30):

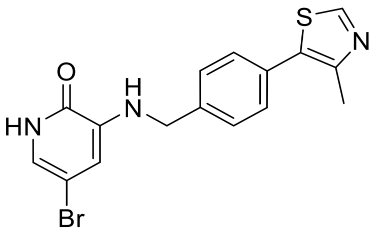

A solution of 3-amino-5-bromo-1H-pyridin-2-one (2.00 g, 10.6 mmol) and 4-(4-methylthiazol-5-yl)benzaldehyde (2.58 g, 12.7 mmol) in DCM (20 mL) and Methanol (20 mL) was stirred for 1h at RT for imine formation. Then sodium cyano borohydride (1.66 g, 26.5 mmol) was added slowly and the reaction mixture was stirred for 1h at RT. Upon completion of the reaction, it was diluted with ice water (10 mL) and extracted with 10% MeOH in DCM (2*30 mL). The organic layer was washed with ice water, dried over Na_2SO_4, filtered, and concentrated under reduced pressure. The resultant crude residue was purified by reverse phase column chromatography (90-95% of 0.1% formic acid in water/acetonitrile) to give 5-bromo-3-((4-(4-methylthiazol-5-yl) benzyl) amino) pyridin-2(1H)-one **30** (0.8 g, 2.12 mmol, 18% yield).

^{1}H NMR (DMSO-d$_6$, 500 MHz) δ 11.60 (br s, 1H), 8.98 (s, 1H), 7.4-7.5 (m, 2H), 7.4-7.4 (m, 2H), 6.76 (d, 1H, *J*=2.2 Hz), 6.4-6.5 (m, 1H), 6.12 (d, 1H, *J*=2.2 Hz), 4.35 (d, 2H, *J*=6.3 Hz), 2.4-2.5 (m, 3H)

^{13}C NMR (DMSO-d$_6$, 125 MHz) δ 156.2, 151.5, 147.8, 139.2, 138.8, 131.1, 130.0, 128.9, 127.5, 118.6, 108.7, 98.7, 45.0, 16.0

HRMS (*m/z*): $[M+H]^+$ calculated for $C_{16}H_{14}BrN_3OS$, 376.01137; found, 376.01172

R_f value (SiO_2, DCM:MeOH=14:1) = 0.1

Synthesis of 31

Reaction scheme:

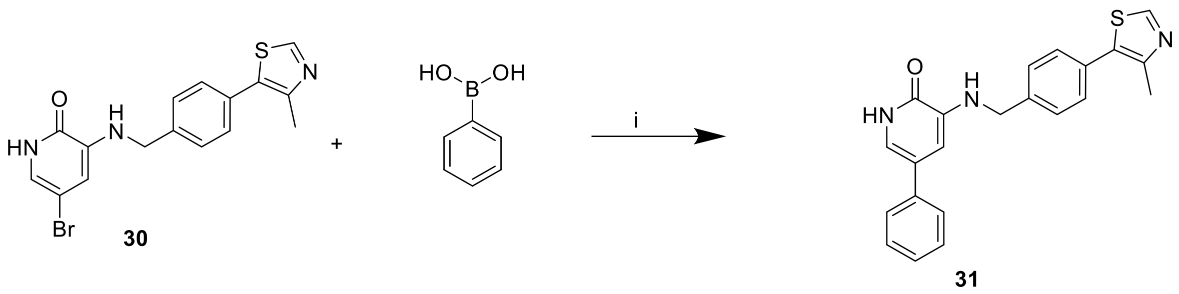

i) $Pd(PPh_3)_4$, Phenyl boronic acid, K_2CO_3, Dioxane, Water, μw, 100°C

3-((4-(4-methylthiazol-5-yl)benzyl)amino)-5-phenylpyridin-2(1H)-one (31):

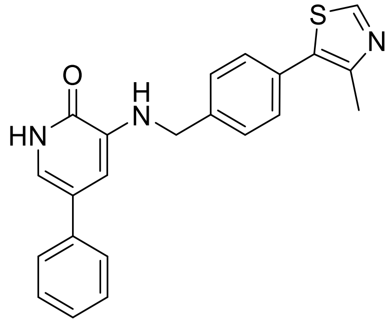

In a 5 mL microwave vial 5-bromo-3-[[4-(4-methylthiazol-5-yl) phenyl] methylamino]-1H-pyridin-2-one **30** (0.15 g, 0.40 mmol) was taken up in 1,4-Dioxane (2.5 mL) and H_2O (0.5 mL). Potassium carbonate (0.14 g, 1.00 mmol) was added, and the reaction mixture was degassed with argon for 2 min. Then phenylboronic acid (0.073 g, 0.60 mmol) and Palladium-tetrakis(triphenylphosphine) (0.046 g, 0.040 mmol) were added and the reaction mixture was irradiated at 100°C for 1 h. Upon completion of the reaction, it was diluted with water (2 mL) and extracted with EtOAc (2*10 mL). The organic layer was washed with brine, dried over Na_2SO_4, filtered, and concentrated under reduced pressure. The resultant crude residue was purified by reverse phase column chromatography (50-70% of 0.1% formic acid in water/acetonitrile) to give 3-((4-(4-methylthiazol-5-yl) benzyl amino)-5-phenylpyridin-2(1H)-one **31** (0.060 g, 0.16 mmol, 37% yield).

^{1}H NMR (DMSO-d_6, 500 MHz) δ 11.61 (br s, 1H), 8.97 (s, 1H), 7.46 (s, 4H), 7.4-7.4 (m, 2H), 7.34 (t, 2H, *J*=7.7 Hz), 7.2-7.3 (m, 1H), 6.92 (br s, 1H), 6.46 (d, 1H, *J*=2.2 Hz), 6.21 (t, 1H, *J*=6.5 Hz), 4.45 (d, 2H, *J*=6.3 Hz), 2.4-2.5 (m, 3H)

^{13}C NMR (DMSO-d_6, 125 MHz) δ 157.0, 151.6 (HSQC), 147.8, 139.4, 138.2, 137.6, 131.1, 129.9, 128.9, 128.7, 127.7, 126.4, 125.3, 119.1, 116.5, 106.1, 45.3, 16.0

HRMS (*m/z*): $[M+H]^+$ calculated for $C_{22}H_{19}N_3OS$, 374.13216; found, 374.13223.

R_f value (SiO_2, DCM:MeOH=14:1) = 0.3

Synthesis of 32 and 33

Reaction scheme:

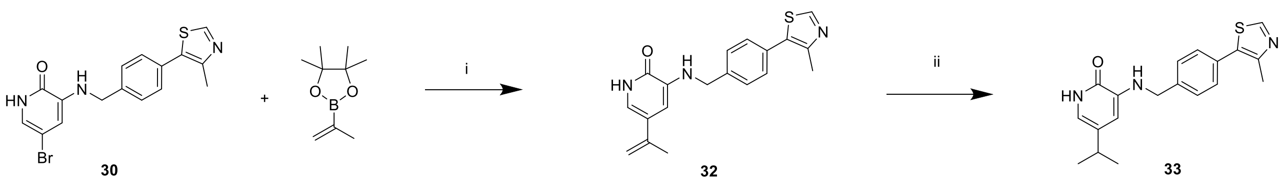

i) $PdCl_2$(dppf)DCM, K_2CO_3, Dioxane, Water, 110°C, µw; ii) 10% Pd/C, Methanol, H_2 (30 psi)

3-((4-(4-methylthiazol-5-yl) benzyl) amino)-5-(prop-1-en-2-yl) pyridin-2(1H)-one (32):

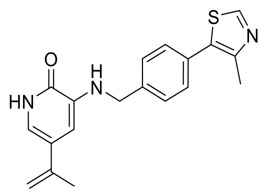

In a 5 mL microwave vial 5-bromo-3-[[4-(4-methylthiazol-5-yl) phenyl] methylamine]-1H-pyridin-2-one **30** (0.10 g, 0.27 mmol) was dissolved in 1,4-dioxane (1.2 mL) and water (0.4 mL) and potassium carbonate (0.092 g, 0.66 mmol) was added. The reaction mixture was degassed with argon for 2 min, then 4,4,5,5-tetramethyl-2-(prop-1-en-2-yl)-1,3,2-dioxaborolane (0.18 g, 1.06 mmol) was added, followed by addition of Pd(dppf)Cl_2 (0.019 g, 0.027 mmol). The reaction mixture was irradiated at 100°C for 1 h. Upon completion of the reaction, it was diluted with water (1 mL) and extracted with 10% MeOH in DCM (2*8 mL). The organic layer was washed with brine solution, dried over Na_2SO_4, filtered, and concentrated under reduced pressure. The resultant crude residue was purified by reverse phase column chromatography (50-60% of 0.1% formic acid in water/acetonitrile) to give 5-isopropenyl-3-[[4-(4-methylthiazol-5-yl) phenyl] methylamine]-1H-pyridin-2-one **32** (0.030 g, 0.08 mmol, 28% yield).

^{1}H NMR (DMSO-d$_6$, 400 MHz) δ 11.43 (br s, 1H), 8.97 (s, 1H), 7.45 (s, 4H), 6.64 (br s, 1H), 6.37 (d, 1H, *J*=2.3 Hz), 6.10 (t, 1H, *J*=6.3 Hz), 5.08 (s, 1H), 4.82 (s, 1H), 4.38 (d, 2H, *J*=6.3 Hz), 2.4-2.5 (m, 3H), 1.90 (s, 3H)

^{13}C NMR (DMSO-d$_6$, 100 MHz) δ 157.2, 151.4, 147.8, 139.5, 139.0, 137.4, 131.1, 129.9, 128.9, 127.7, 118.9, 116.6, 109.4, 104.4, 45.4, 20.6, 16.0

HRMS (*m/z*): $[M+H]^+$ calculated for $C_{19}H_{19}N_3OS$, 338.13216; found, 338.13217.

R_f value (SiO_2, DCM:MeOH=14:1) = 0.5

5-isopropyl-3-((4-(4-methylthiazol-5-yl) benzyl) amino) pyridin-2(1H)-one (33):

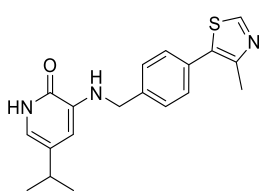

To a stirred solution of 5-isopropenyl-3-[[4-(4-methylthiazol-5-yl) phenyl] methylamine]-1H-pyridin-2-one **32** (0.050 g, 0.15 mmol) in methanol (1 mL) was added 10% Pd/C (0.047 g, 0.45 mmol) and the

reaction mixture was stirred for 20 h at RT under 1 atm of hydrogen. Upon completion of the reaction, the mixture was filtered through Celite bed, rinsed with DCM (2*10 mL) and the filtrate was concentrated under reduced pressure. The resultant crude was purified by reverse phase column chromatography (50-60% of 0.1% formic acid in water/acetonitrile) to give 5-isopropyl-3-[[4-(4-methylthiazol-5-yl) phenyl] methylamine]-1H-pyridin-2-one **33** (0.013 g, 0.03 mmol, 25% yield).

^{1}H NMR (DMSO-d$_6$, 400 MHz) δ 11.15 (br s, 1H), 8.97 (s, 1H), 7.44 (s, 4H), 6.35 (br s, 1H), 6.09 (d, 1H, *J*=1.5 Hz), 5.95 (br t, 1H, *J*=6.2 Hz), 4.34 (br d, 2H, *J*=6.3 Hz), 2.4-2.5 (m, 3H), 1.01 (d, 6H, *J*=6.8 Hz), 1H under DMSO

^{13}C NMR (DMSO-d$_6$, 100 MHz) δ 156.9, 151.4, 147.8, 139.5, 137.8, 131.1, 129.8, 128.8, 127.8, 125.4, 114.4, 107.5, 45.5, 30.1, 23.0, 15.9

HRMS (*m/z*): [M+H]$^+$ calculated for $C_{19}H_{21}N_3OS$, 340.14781; found: 340.14758.

R_f value (SiO_2, DCM:MeOH=9:1) = 0.4

Synthesis of 34

Reaction scheme:

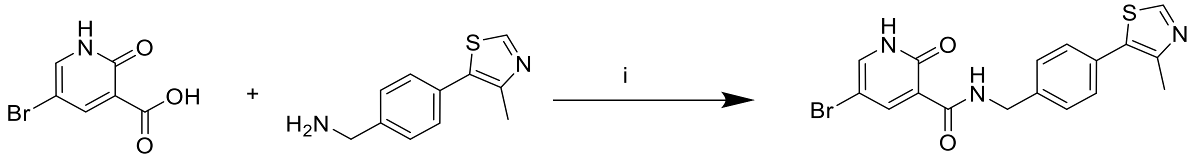

34

i) HATU, HOAt, DIPEA, DMF, RT

5-bromo-N-(4-(4-methylthiazol-5-yl) benzyl)-2-oxo-1,2-dihydropyridine-3-carboxamide (34):

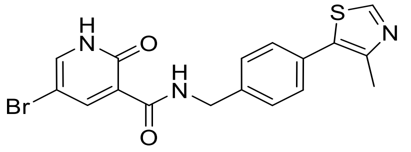

To a solution of 5-bromo-2-oxo-1H-pyridine-3-carboxylic acid (0.10 g, 0.46 mmol) in DMF (1 mL) was added HATU (0.26 g, 0.69 mmol) and HOAt (0.094 g, 0.69 mmol) and the mixture was stirred for 10 min followed by addition of [4-(4-methylthiazol-5-yl) phenyl] methanamine (0.11 g, 0.55 mmol). Stirring was continued for 15 h at RT. Upon completion of the reaction, it was diluted with ice water (5 mL), extracted with 10% MeOH in DCM (2*15 mL), washed with ice water, and dried over Na_2SO_4, filtered, and concentrated under reduced pressure. The resultant crude residue was purified by reverse phase column chromatography (90-95% of 0.1%formic acid in water/acetonitrile to give 5-bromo-N-(4-(4-methylthiazol-5-yl)benzyl)-2-oxo-1,2-dihydropyridine-3-carboxamide **34** (0.08 g, 0.19 mmol, 42% yield).

^{1}H NMR (DMSO-d$_6$, 400 MHz) δ 12.92 (br s, 1H), 10.09 (br t, 1H, *J*=5.8 Hz), 8.99 (s, 1H), 8.33 (d, 1H, *J*=3.0 Hz), 8.03 (d, 1H, *J*=3.0 Hz), 7.4-7.5 (m, 2H), 7.4-7.4 (m, 2H), 4.58 (d, 2H, *J*=5.8 Hz), 2.46 (s, 3H)

^{13}C NMR (DMSO-d_6, 100 MHz) δ 162.2, 161.0, 151.5, 147.8, 145.8, 139.9, 138.9, 131.0, 130.1, 129.0, 127.9, 121.5, 120.6, 42.0, 15.9

HRMS (*m/z*): $[M+H]^+$ calculated for $C_{17}H_{14}BrN_3O_2S$, 404.00629; found, 404.00668.

R_f value (SiO_2, DCM:MeOH=14:1) = 0.3

Synthesis of 35
Reaction scheme:

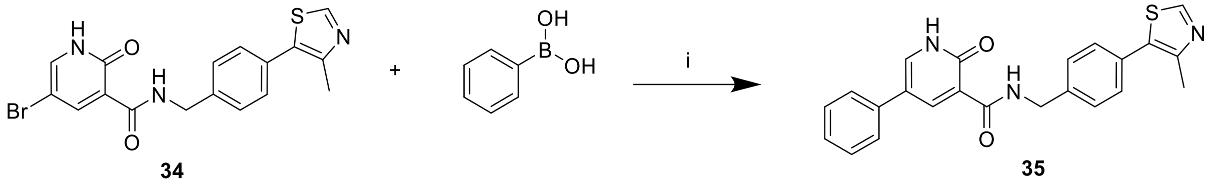

i) $Pd(PPh_3)_4$, Phenyl boronic acid, K_2CO_3, Dioxane, Water, 100°C

N-(4-(4-methylthiazol-5-yl) benzyl)-2-oxo-5-phenyl-1,2-dihydropyridine-3-carboxamide (35):

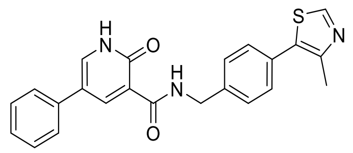

To a stirred solution of 5-bromo-N-[[4-(4-methylthiazol-5-yl)phenyl]methyl]-2-oxo-1H-pyridine-3-carboxamide **34** (0.15 g, 0.37 mmol) in 1,4-Dioxane (1.2 mL) and H_2O (0.3 mL), Potassium carbonate (0.13 g, 0.93 mmol) was added and the reaction mixture was degassed for 2 min with argon. Then phenyl boronic acid (0.054 g, 0.45 mmol) was added and it was again degassed with argon for 5 min, followed by addition of Palladium-tetrakis(triphenylphosphine) (0.043 g, 0.037 mmol). The reaction mixture was stirred for 1 h at 100°C. Upon completion of the reaction, it was diluted with water (1 mL) and extracted with 10% MeOH in DCM (2*8 mL). The organic layer was washed with brine, dried over Na_2SO_4, filtered, and concentrated under reduced pressure. The resultant crude residue was purified by reverse phase column chromatography (50-60% of 0.1% formic acid in water/acetonitrile) to give N-[[4-(4-methylthiazol-5-yl) phenyl] methyl]-2-oxo-5-phenyl-1H-pyridine-3-carboxamide **35** (0.027 g, 0.06 mmol, 17% yield).

^1H NMR (DMSO-d_6, 500 MHz) δ 11.9-13.6 (m, 1H), 10.31 (br t, 1H, *J*=5.8 Hz), 8.99 (s, 1H), 8.65 (d, 1H, *J*=3.2 Hz), 8.07 (d, 1H, *J*=2.8 Hz), 7.62 (d, 2H, *J*=7.6 Hz), 7.4-7.5 (m, 6H), 7.3-7.4 (m, 1H), 4.61 (d, 2H, *J*=6.0 Hz), 2.45 (s, 3H)

^{13}C NMR (DMSO-d_6, 125 MHz) δ 163.4, 161.9, 151.5, 147.9, 142.1, 139.2, 137.0, 135.4, 131.0, 130.1, 129.1, 129.0, 127.9, 127.2, 125.6, 119.8, 119.0, 42.0, 15.9

HRMS (*m/z*): $[M+H]^+$ calculated for $C_{23}H_{19}N_3O_2S$, 402.12707; found, 402.12717.

R_f value (SiO_2, DCM:MeOH=14:1) = 0.5

Synthesis of 36
Reaction scheme:

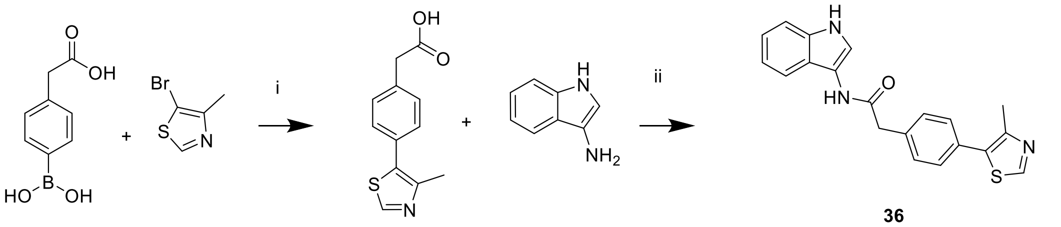

36

i) $PdCl_2$dppf, CS_2CO_3,1,4-Dioxane, 100°C, 16 h; ii) HATU, HOAT, DIPEA, DMF, RT, 16 h.

N-(1H-indol-3-yl)-2-(4-(4-methylthiazol-5-yl) phenyl) acetamide (36):

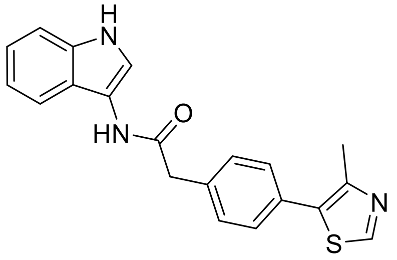

To a stirred solution of 2-(4-boronophenyl)acetic acid (0.20 g, 1.11 mmol) in 1,4-dioxane (1.4 mL) was added 5-bromo-4-methyl-thiazole (0.30 g, 1.67 mmol) followed by caesium carbonate (0.91 g, 2.78 mmol) and water (0.6 mL). The reaction mixture was degassed for 15 min with nitrogen, then Pd(dppf)Cl_2 (0.081 g, 0.11 mmol) was added, followed by purging with nitrogen for 5 min. The reaction mixture was stirred for 6 h at 100°C. Upon completion of the reaction, the solvents were evaporated under reduced pressure to give crude 2-[4-(4-methylthiazol-5-yl)phenyl]acetic acid (0.15 g, 18% yield), which was used in the following step without further purification.

To a stirred solution of 2-[4-(4-methylthiazol-5-yl)phenyl]acetic acid (0.50 g, 2.14 mmol) in DMF (5 mL) was added DIPEA (1.1 mL, 6.43 mmol) and HATU (0.90 g, 2.36 mmol). The mixture was stirred for 5 min at RT. Then 1H-indol-3-amine (0.31 g, 2.36 mmol) was added at 0°C and stirring was continued for 16 h at RT. Upon completion of the reaction, ice cold water was added and the precipitated solid was collected by filtration and purified by reverse phase column chromatography (50-60% of 0.1% formic acid in water/acetonitrile) to give of N-(1H-indol-3-yl)-2-[4-(4-methylthiazol-5-yl) phenyl] acetamide **36** (0.13 g, 0.03 mmol, 17% yield).

^{1}H NMR (DMSO-d_6, 500 MHz) δ 10.77 (br s, 1H), 10.09 (s, 1H), 8.99 (s, 1H), 7.79 (d, 1H, *J*=7.9 Hz), 7.70 (d, 1H, *J*=2.2 Hz), 7.47 (s, 4H), 7.33 (d, 1H, *J*=7.9 Hz), 7.1-7.1 (m, 1H), 7.0-7.0 (m, 1H), 3.77 (s, 2H), 2.4-2.5 (m, 3H)

^{13}C NMR (DMSO-d_6, 125 MHz) δ 167.5, 151.5, 147.7, 136.5, 133.4, 131.1, 129.7, 129.6, 128.8, 121.4, 120.3, 118.1, 117.8, 115.3, 115.0, 111.4, 41.9, 15.9

HRMS (*m/z*): $[M+H]^+$ calculated for $C_{20}H_{17}N_3OS$, 348.11651; found, 348.11636.

R_f value (SiO_2, DCM:MeOH=14:1) = 0.5

Synthesis of 37
Reaction scheme:

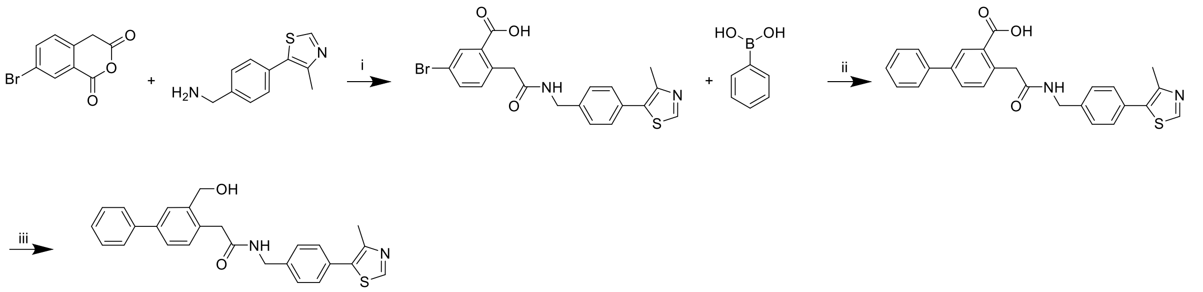

37

i) Et_3N, Toluene, 100 °C, 3h; ii) $Pd(dppf)Cl_2$.DCM, Dioxane, 100°C, 6 h; iii) 2M LAH, THF, 0°C-rt, 6 h;

2-(3-(hydroxymethyl)-[1,1'-biphenyl]-4-yl)-N-(4-(4-methylthiazol-5-yl) benzyl) acetamide (37):

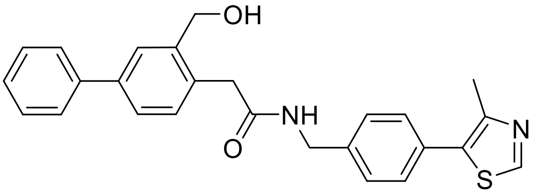

To a stirred solution of 7-bromoisochromane-1,3-dione (1.00 g, 4.15 mmol) in toluene (20 mL) was added Et_3N (1.7 mL, 12.4 mmol) and [4-(4-methylthiazol-5-yl) phenyl] methoxamine hydrochloride (1.10 g, 4.56 mmol). The reaction mixture was stirred for 3h at 100°C. Upon completion of the reaction, it was concentrated under reduced pressure. The crude was triturated with a mixture of acetonitrile and water to obtain a solid, which was dried under reduced pressure to get 5-bromo-2-[2-[[4-(4-methylthiazol-5-yl) phenyl] methyl amino]-2-oxo-ethyl] benzoic acid (0.60 g, 24% yield), which was used in the next step without further purification.

To a stirred solution of 5-bromo-2-[2-[[4-(4-methylthiazol-5-yl)phenyl]methylamino]-2-oxo-ethyl]benzoic acid (0.20 g, 0.45 mmol) in 1,4-dioxane (0.9 mL) was added phenyl boronic acid (0.055 g, 0.45 mmol) followed by caesium carbonate (0.37 g, 1.12 mmol) and water (0.5 mL). The reaction mixture was degassed for 15 min with nitrogen, then $Pd(dppf)Cl_2$ (0.033 g, 0.045 mmol) was added, followed by purging with nitrogen for 5 min. The reaction mixture was stirred for 6 h at 100°C. Upon completion of the reaction, the solvents were evaporated under reduced pressure. The resultant crude residue was purified by silica gel column chromatography (70-80% of EtOAc/petroleum ether) to give 4-(2-((4-(4-methylthiazol-5-yl) benzyl) amino)-2-oxoethyl)-[1,1'-biphenyl]-3-carboxylic acid (0.12 g, 47% yield) that was directly used in the next step.

To a stirred solution of 2-[2-[[4-(4-methylthiazol-5-yl) phenyl] methyl amino]-2-oxo-ethyl]-5-phenyl-benzoic acid (0.15 g, 0.34 mmol) in THF (5 mL) was added 2.4M LAH in THF (0.019 g, 0.508 mmol) at 0°C and the reaction mixture was stirred for 6h at RT. Upon completion of the reaction, it was quenched with 1N aq. HCl (pH 2) and extracted with 10% methanol in DCM (2*5 mL). The organic layer was washed with brine solution, dried over Na_2SO_4, filtered, and concentrated under reduced pressure. The resultant crude residue was purified by reverse phase column chromatography (40-55% of 0.1% formic acid in water/acetonitrile) to get 2-[2-(hydroxymethyl)-4-phenyl-phenyl]-N-[[4-(4-methylthiazol-5-yl) phenyl] methyl] acetamide **37** (0.040 g, 0.09 mmol, 27% yield).

^{1}H NMR (DMSO-d$_6$, 500 MHz) δ 8.99 (s, 1H), 8.66 (t, 1H, *J*=5.8 Hz), 7.69 (d, 1H, *J*=1.9 Hz), 7.65 (dd, 2H, *J*=0.9, 8.2 Hz), 7.4-7.5 (m, 5H), 7.3-7.4 (m, 4H), 5.27 (br t, 1H, *J*=5.2 Hz), 4.63 (d, 2H, *J*=4.4 Hz), 4.33 (d, 2H, *J*=6.0 Hz), 3.62 (s, 2H), 2.45 (m, 3H)

^{13}C NMR (DMSO-d$_6$, 125 MHz) δ 170.3, 151.3 (HSQC) 147.8, 141.2, 140.2, 139.3, 138.4, 133.2, 130.6, 129.9, 128.9, 128.9, 127.8, 127.2, 126.5, 125.7, 124.9, 61.1, 42.0, 38.7, 15.9, 1C missing

HRMS (*m/z*): $[M+H]^+$ calculated for $C_{26}H_{24}N_2O_2S$, 429.16313; found, 429.16315.

R_f value (SiO_2, DCM:MeOH=14:1) = 0.2

Synthesis of 38, 39 and 40
Reaction scheme:

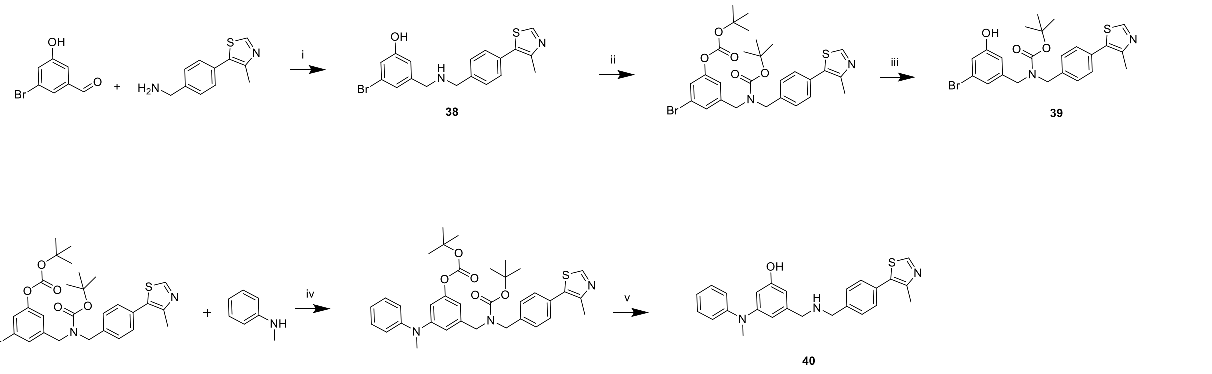

i) $NaCNBH_3$, DIPEA, acetic acid, DCM, rt, 16 h; ii) $(Boc)_2O$, DCM, 0-rt, 6 h; iii) piperidine, DCM, 0°C-rt, 16 h; iv) Caesium carbonate, RuPhos Pd G3, DME; v) 4M HCl in 1,4-dioxane, DCM

3-bromo-5-(((4-(4-methylthiazol-5-yl) benzyl) amino) methyl) phenol (38):

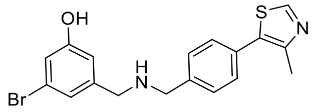

To a stirred solution of [4-(4-methylthiazol-5-yl) phenyl] methoxamine hydrochloride (0.12 g, 0.50 mmol) in DCM (4 mL) and Methanol (4 mL), DIPEA (0.13 mL, 0.75 mmol) was added at 0°C and it was stirred for 5 min. Acetic acid (0.057 mL, 1.00 mmol) was added at 0°C (pH 6 to 6.5) and the reaction mixture was stirred for 5 min. Then 3-bromo-5-hydroxy-benzaldehyde (0.10 g, 0.50 mmol) was added and stirring was continued for 2 h at RT, before sodium cyanoborohydride (0.13 g, 1.99 mmol) was added. The mixture was stirred for 16 h at RT. Upon completion of the reaction, it was diluted with ice cold water (5 mL) and concentrated under reduced pressure. The resultant crude residue was purified by reverse phase column chromatography (40-55% of 0.1% formic acid in water/acetonitrile) to get 3-bromo-5-[[[4-(4-methylthiazol-5-yl) phenyl] methylamine] methyl] phenol **38** (0.050 g, 0.12 mmol, 25% yield).

^{1}H NMR (DMSO-d$_6$, 400 MHz) δ 9.81 (s, 1H), 8.99 (s, 1H), 7.45 (s, 4H), 6.99 (s, 1H), 6.7-6.9 (m, 2H), 3.72 (s, 2H), 3.65 (s, 2H), 2.5-2.5 (m, 3H), NH – missing

^{13}C NMR (DMSO-d$_6$, 100 MHz) δ 158.4, 151.4, 147.7, 144.3, 140.3, 131.2, 129.8, 128.7, 128.5, 121.6, 121.1, 116.3, 114.1, 51.5, 51.4, 16.0

HRMS (*m/z*): $[M+H]^+$ calculated for $C_{18}H_{17}BrN_2OS$, 389.03177; found, 389.03232.

R_f value (SiO_2, DCM:MeOH=14:1) = 0.2

tert-butyl (3-bromo-5-hydroxybenzyl) (4-(4-methylthiazol-5-yl) benzyl) carbamate (39):

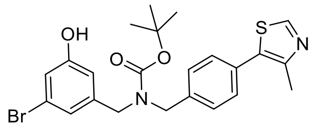

To a stirred solution of 3-bromo-5-[[[4-(4-methylthiazol-5-yl) phenyl] methylamine] methyl] phenol (0.050 g, 0.073 mmol) in DCM (5 mL), Triethylamine (0.020 mL, 0.15 mmol) and Boc anhydride (0.020 mL, 0.088 mmol) were added at 0°C and the reaction was allowed to reach RT and stirred for 14 h. Upon completion of the reaction, it was quenched with ice cold water (10 mL) and extracted with DCM (2*30 mL). The combined organic layer was dried over sodium sulphate and concentrated under reduced pressure. The crude product was purified by flash silica column chromatography to afford [3-bromo-5-[[tert-butoxycarbonyl-[[4-(4-methylthiazol-5-yl)phenyl]methyl]amino]methyl] phenyl] tert-butyl carbonate (0.055 g, 92% yield) that was directly used in the next step.

To a stirred solution of [3-bromo-5-[[tert-butoxycarbonyl-[[4-(4-methylthiazol-5-yl)phenyl]methyl]amino]methyl]phenyl] tert-butyl carbonate (0.30 g, 0.51 mmol) in DCM (5 mL) was added, piperidine (0.15 mL, 1.53 mmol) at 0°C and the reaction was stirred at RT for 16 h. Upon completion of the reaction, it was quenched with ice cold water (20 mL) and extracted with DCM (2*20 mL). The organic layer was washed with brine solution, dried over sodium sulphate and concentrated under reduced pressure. The resultant residue was purified by normal phase column chromatography (40% EtOAc in petroleum ether) to get tert-butyl N-[(3-bromo-5-hydroxy-phenyl)methyl]-N-[[4-(4-methylthiazol-5-yl)phenyl]methyl]carbamate **39** (59 mg, 0.12 mmol, 23% yield).

^{1}H NMR (DMSO-d$_6$, 500 MHz) δ 9.90 (s, 1H), 8.99 (s, 1H), 7.46 (d, 2H, *J*=8.2 Hz), 7.32 (br d, 2H, *J*=7.6 Hz), 6.7-6.9 (m, 2H), 6.64 (br s, 1H), 4.2-4.6 (m, 4H), 2.4-2.5 (m, 3H), 1.40 (s, 9H)

^{13}C NMR (DMSO-d$_6$, 100 MHz) δ 158.6, 155.0, 151.5, 147.9, 141.9, 138.0, 131.0, 130.3, 129.0, 127.9, 121.7, 120.6, 116.7, 113.4, 79.5, 49.4, 48.9, 27.9, 15.9

HRMS (*m/z*): $[M+H]^+$ calculated for $C_{23}H_{25}BrN_2O_3S$, 489.08420; found, 489.08432.

R_f value (SiO_2, DCM:MeOH=14:1) = 0.4

3-(methyl(phenyl)amino)-5-(((4-(4-methylthiazol-5-yl) benzyl) amino) methyl) phenol (40)

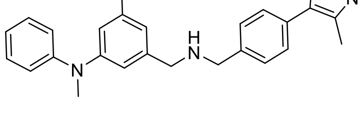

In a 5 mL microwave vial [3-bromo-5-[[tert-butoxycarbonyl-[[4-(4-methylthiazol-5-yl)phenyl]methyl]amino]methyl]phenyl] tert-butyl carbonate (0.10 g, 0.17 mmol), N-methyl aniline

(0.018 g, 0.17 mmol) and Caesium carbonate (0.14 g, 0.42 mmol) were taken up in DME (2 mL) and degassed for 2 min. Then RuPhos-Pd-G3 (0.014 g, 0.017 mmol) was added, and the reaction mixture was irradiated for 1 h at 110°C. Upon completion of the reaction, it was diluted with ice cold water (5 mL) and extracted with EtOAc (2*10 mL). The organic layer was washed with brine solution, dried over Na_2SO_4 and concentrated under reduced pressure. The resultant crude residue was purified by reverse phase column chromatography (50-60% of 0.1% formic acid in water/acetonitrile) to get [3-[[tert-butoxycarbonyl-[[4-(4-methylthiazol-5-yl) phenyl] methyl] amino] methyl]-5-(N-methylanilino) phenyl] tert-butyl carbonate (0.030 g, 11% yield) which was directly used in the next step.

To a stirred solution of [3-[[tert-butoxycarbonyl-[[4-(4-methylthiazol-5-yl)phenyl]methyl]amino]methyl]-5-(N-methylanilino)phenyl]tert-butyl carbonate (0.15 g, 0.24 mmol) in DCM (5 mL), 4M HCl in 1,4-dioxane (0.11 g, 1.22 mmol) was added at 0°C and the reaction was stirred at RT for 16 h. Upon completion of the reaction, it was concentrated under reduced pressure. The resultant crude residue was purified by reverse phase column chromatography (40-55% of 0.1% formic acid in water/acetonitrile) to get 3-(N-methylanilino)-5-[[[4-(4-methylthiazol-5-yl)phenyl]methylamino]methyl]phenol **40** (0.037 g, 0.08 mmol, 33% yield)

^1H NMR (DMSO-d_6, 400 MHz) δ 9.0-9.5 (m, 1H), 8.99 (s, 1H), 7.44 (s, 4H), 7.2-7.3 (m, 2H), 7.01 (d, 2H, *J*=7.9 Hz), 6.9-7.0 (m, 1H), 6.46 (s, 1H), 6.40 (s, 1H), 6.26 (t, 1H, *J*=2.2 Hz), 3.75 (s, 2H), 3.61 (s, 2H), 3.21 (s, 3H), 2.4-2.5 (m, 3H), NH – missing

^{13}C NMR (DMSO-d_6, 100 MHz) δ 158.0, 151.4, 149.6, 148.7, 147.8, 141.5, 139.6, 131.1, 129.9, 129.1, 128.7, 128.7, 121.2, 120.6, 110.5, 108.2, 105.4, 52.0, 51.3, 16.0, 1C under DMSO

HRMS (*m/z*): $[M+H]^+$ calculated for $C_{25}H_{25}N_3OS$, 416.17911; found, 416.17877.

R_f value (SiO_2, DCM:MeOH=14:1) = 0.3

Synthesis of 41
Reaction scheme:

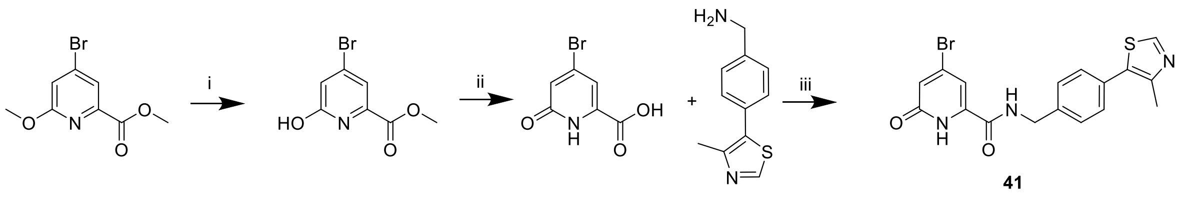

i) BBr_3(1M in DCM), -70°C, DCM, 5h; ii) LiOH, THF, water, 0°C-rt, 6 h; iii) HATU, DIPEA, DMF, rt, 16 h

4-bromo-N-(4-(4-methylthiazol-5-yl) benzyl)-6-oxo-1,6-dihydropyridine-2-carboxamide (41):

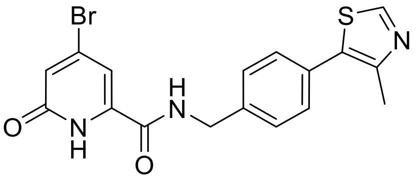

To a stirred solution of methyl 4-bromo-6-methoxy-pyridine-2-carboxylate (0.10 g, 0.41 mmol) in DCM (5 mL) at -70°C, 1M boron tribromide solution in DCM (0.28 mL, 2.03 mmol) was added and the

reaction mixture was stirred at -10°C for 4 h. Upon completion of the reaction, it was cooled to -70°C and quenched with methanol (5 mL). After reaching RT, it was concentrated under reduced pressure. The resulting residue was purified by flash silica column chromatography (40% EtOAc in petroleum ether) to get methyl 4-bromo-6-hydroxy-pyridine-2-carboxylate (0.030 g, 12% yield) that was directly used in the next step.

To a stirred solution of methyl 4-bromo-6-hydroxy-pyridine-2-carboxylate (0.070 g, 0.30 mmol) in THF (5 mL) and water (2 mL), LiOH (0.038 g, 0.91 mmol) was added, and the reaction mixture was stirred for 6h at RT. Upon completion of the reaction, it was concentrated under reduced pressure and the crude residue was purified by reverse phase column chromatography (40-55% of 0.1% formic acid in water/acetonitrile) to get 4-bromo-6-hydroxy-pyridine-2-carboxylic acid (0.07 g, 82% yield) which was directly used in the next step.

To a stirred solution of 4-bromo-6-oxo-1H-pyridine-2-carboxylic acid (0.030 g, 0.14 mmol) in DMF (5 mL) were added DIPEA (0.12 mL, 0.69 mmol) and HATU (0.078 g, 0.21 mmol). After 10 min [4-(4-methylthiazol-5-yl)phenyl]methoxamine hydrochloride (0.036 g, 0.15 mmol) was added at 0°C and the reaction mixture was stirred for 2h at RT. Upon completion of the reaction, it was concentrated under reduced pressure and the resultant residue was purified by reverse phase column chromatography (60-65% of 0.1% formic acid in water/acetonitrile) to get 4-bromo-N-[[4-(4-methylthiazol-5-yl)phenyl]methyl]-6-oxo-1H-pyridine-2-carboxamide **41** (0.021 g, 0.05 mmol, 36% yield).

^{1}H NMR (DMSO-d$_6$, 400 MHz) δ 10.9-12.1 (m, 2H), 9.0-9.2 (m, 1H), 8.99 (s, 1H), 7.3-7.5 (m, 5H), 6.99 (s, 1H), 4.50 (br d, 1H, *J*=5.8 Hz), 2.4-2.5 (m, 3H)

^{13}C NMR (DMSO-d$_6$, 100 MHz) δ 162.7, 161.5, 151.5, 147.9, 138.7, 134.7, 131.0, 130.1, 128.9, 128.0, 119.3, 113.1, 42.3, 15.9, 1C - missing

HRMS (*m/z*): $[M+H]^+$ calculated for $C_{17}H_{14}BrN_3O_2S$, 404.00629; found, 404.00638.

R_f value (SiO_2, DCM:MeOH=14:1) = 0.2

Synthesis of 42
Reaction scheme:

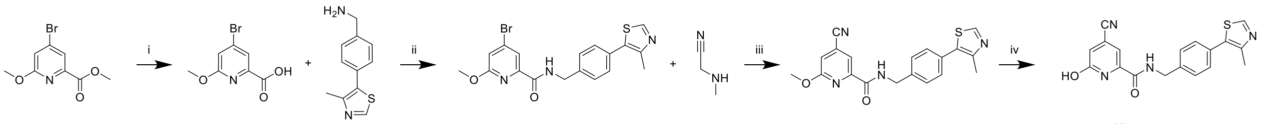

42

i) BBr_3 (1M in DCM), -70°C, DCM,5h; ii) LiOH, THF, water, 0-rt, 6 h; iii) HATU, DIPEA, DMF, RT, 16h; iv) pyridine, HCl, 150°C, 2h

4-cyano-6-hydroxy-N-(4-(4-methylthiazol-5-yl) benzyl) picolinamide (42):

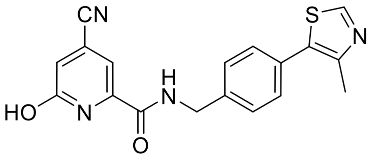

To a stirred solution of methyl 4-bromo-6-methoxy-pyridine-2-carboxylate (0.20 g, 0.81 mmol) in THF (5 mL) and water (2 mL), LiOH (0.10 g, 2.44 mmol) was added, and the reaction mixture was stirred for 6h at RT. Upon completion of the reaction, it was concentrated under reduced pressure and the crude residue was purified by reverse phase column chromatography (50-60% of 0.1% formic acid in water/acetonitrile) to get 4-bromo-6-methoxy-pyridine-2-carboxylic acid (0.20 g, quant. yield) which was directly used in the next step.

To a stirred solution of 4-bromo-6-methoxy-pyridine-2-carboxylic acid (0.20 g, 0.66 mmol) in DMF (5 mL), DIPEA (0.58 mL, 3.32 mmol) and HATU (0.38 g, 1.00 mmol) were added. After 10 min [4-(4-methylthiazol-5-yl) phenyl] methanamine hydrochloride (0.18 g, 0.73 mmol) was added at 0°C and the reaction mixture was stirred for 2 h at RT. Upon completion of the reaction, it was quenched with ice cold water and extracted with EtOAc (2*5 mL). The organic layer was washed with water and brine solution, dried over sodium sulphate, and concentrated under reduced pressure. The resultant residue was purified by normal phase column chromatography (80-100% EtOAc in hexane) to get 4-bromo-6-methoxy-N-[[4-(4-methylthiazol-5-yl) phenyl] methyl] pyridine-2-carboxamide (0.080 g, 28% yield) which was directly used in the next step.

In a 5 mL microwave vial 4-bromo-6-methoxy-N-[[4-(4-methylthiazol-5-yl)phenyl]methyl]pyridine-2-carboxamide (0.030 g, 0.072 mmol), 2-(methylamine)acetonitrile (0.025 g, 0.36 mmol), and Caesium carbonate (0.058 g, 0.18 mmol) were taken up in DME (2 mL) and degassed for 5 min, then RuPhos Pd G3 (0.006 g, 0.0072 mmol) was added and the reaction mixture was irradiated for 1 h at 110°C. Upon completion of the reaction, it was diluted with ice cold water (5 mL) and extracted with EtOAc (2*10 ml). The organic layer was washed with brine solution, dried over Na_2SO_4, and concentrated under reduced pressure to obtain 4-cyano-6-methoxy-N-(4-(4-methylthiazol-5-yl) benzyl) picolinamide, which was forwarded to the next step without any further purification.

To 4-cyano-6-methoxy-N-(4-(4-methylthiazol-5-yl) benzyl) picolinamide (0.050 g, 0.12 mmol) was added Pyridine HCl (0.14 g, 1.23 mmol) and the reaction mixture was heated to 150°C for 2 h. Upon completion of the reaction, it was cooled to RT and diluted with acetonitrile (2 mL) and water (3 mL). It was purified by reverse phase column chromatography (30-40% of 0.1% formic acid in water/acetonitrile) to give 4-cyano-6-hydroxy-N-(4-(4-methylthiazol-5-yl) benzyl) picolinamide **42** (0.012 g, 0.03 mmol, 25% yield).

^{1}H NMR (DMSO-d$_6$, 400 MHz) δ 9.05 (br t, 1H, *J*=6.1 Hz), 8.98 (s, 1H), 7.45 (m, 5H), 7.15 (s, 1H), 4.51 (d, 2H, *J*=6.1 Hz), 2.45 (m, 3H)

^{13}C NMR (DMSO-d$_6$, 100 MHz) δ 163.8, 162.3, 151.5, 148.2, 147.8, 138.8, 131.0, 130.1, 128.9, 128.0, 122.5, 119.1, 116.6, 111.1, 42.2, 15.9

HRMS (*m/z*): $[M+H]^+$ calculated for $C_{18}H_{14}N_4O_2S$, 351.09102; found, 351.09146.

R_f value (SiO_2, DCM:MeOH=14:1) = 0.3

Synthesis of 43
Reaction scheme

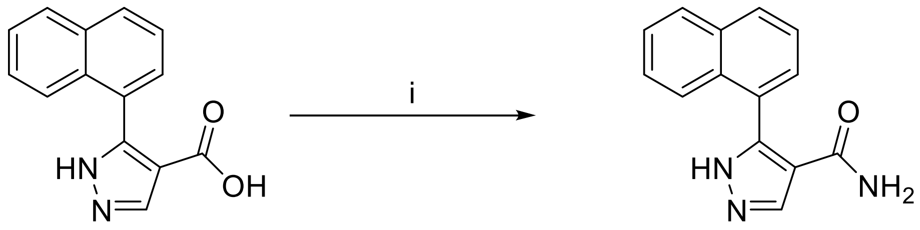

43

i) $SOCl_2$, aqueous ammonia, THF, 80°C

5-(naphthalen-1-yl)-1H-pyrazole-4-carboxamide (43)

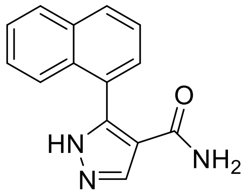

To 5-(1-naphthyl)-1H-pyrazole-4-carboxylic acid (200 mg, 0.84 mmol) was added thionyl chloride (4.3 mL, 58.8 mmol) at room temperature. The reaction was stirred at 80°C for 4 h. Upon completion of the reaction, it was concentrated under reduced pressure to remove the excess of thionyl chloride. The crude residue was dissolved in THF (5 mL) and aqueous ammonia (3 mL) was added dropwise at 0°C. The reaction was stirred at RT for 12 h. Upon completion of the reaction, the solvent was evaporated under reduced pressure and the residue was diluted with water (5 mL) and extracted with dichloromethane (3*20 mL). The combined organic layer was washed with brine, dried over anhydrous Na_2SO_4, and concentrated under reduced pressure. The crude residue was purified by column chromatography (2-4% MeOH in DCM) to afford 5-(naphthalen-1-yl)-1H-pyrazole-4-carboxamide **43** (56 mg, 0.23 mmol, 27% yield).

^{1}H NMR (DMSO-d$_6$, 400 MHz) δ 13.31 (br d, 1H, *J*=19.8 Hz), 8.23 (br d, 1H, *J*=107.0 Hz), 7.8-8.1 (m, 2H), 7.56 (m, 5H), 6.97 (m, 2H)

^{13}C NMR (DMSO-d$_6$, 100 MHz) δ 163.9, 140.3, 132.9, 132.0, 131.2, 129.0, 128.1, 127.9, 127.6, 126.5, 125.8, 125.4, 125.1, 116.3

HRMS (*m/z*): $[M+H]^+$ calculated for $C_{14}H_{11}N_3O$, 238.09749; found, 238.09769.

R_f value (SiO_2, DCM:MeOH=14:1) = 0.3

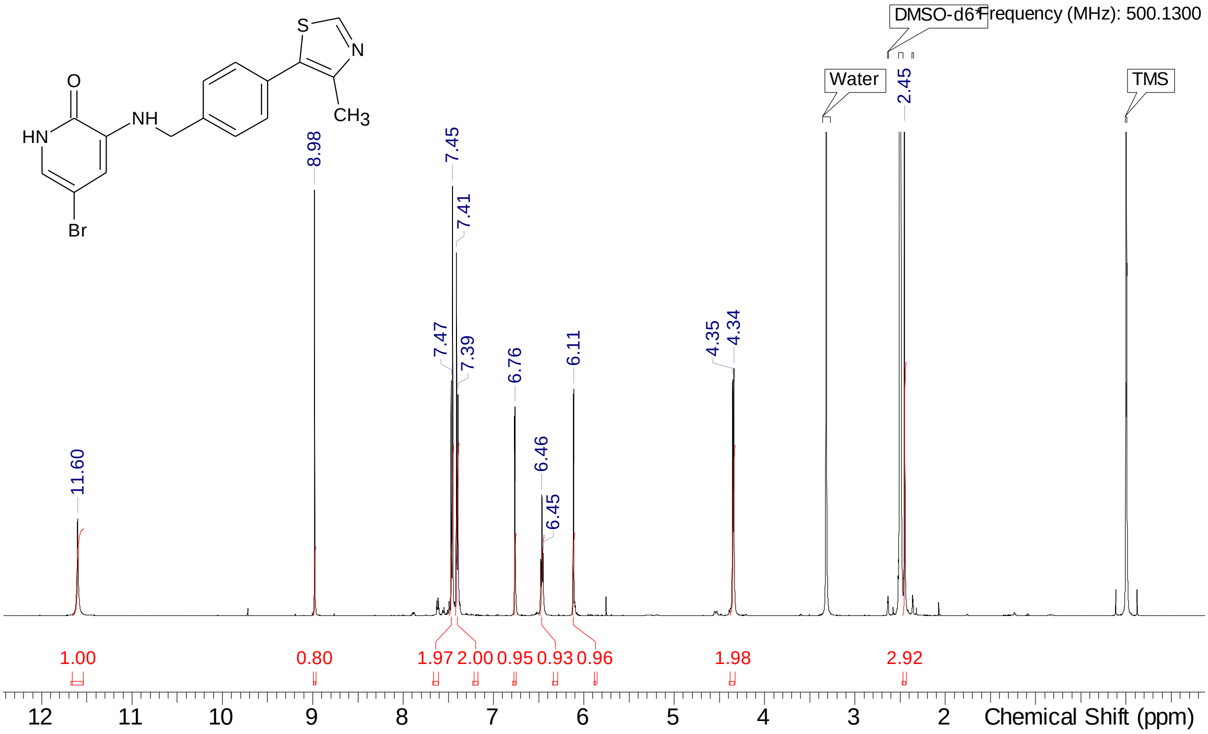
HN
O
NH
Br
S
N
CH3
DMSO-d6*
Frequency (MHz): 500.1300
Water
TMS
2.45
8.98
7.45
7.41
7.47
7.39
6.76
6.11
6.46
6.45
4.35
4.34
11.60
1.00
0.80
1.97
2.00
0.95
0.93
0.96
1.98
2.92
12 11 10 9 8 7 6 5 4 3 2
Chemical Shift (ppm)

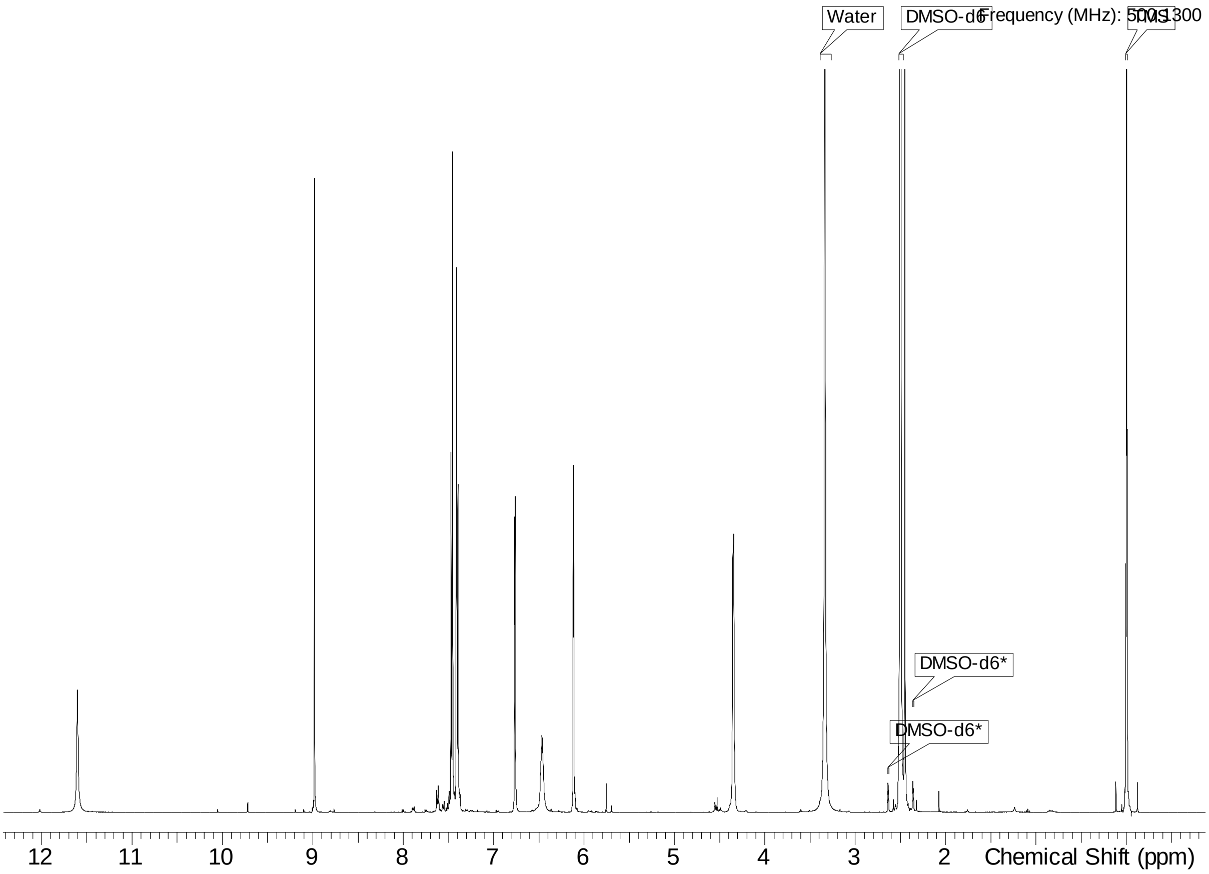
Water
DMSO-d6
Frequency (MHz): 500.1300
TMS
DMSO-d6*
DMSO-d6*
12 11 10 9 8 7 6 5 4 3 2
Chemical Shift (ppm)

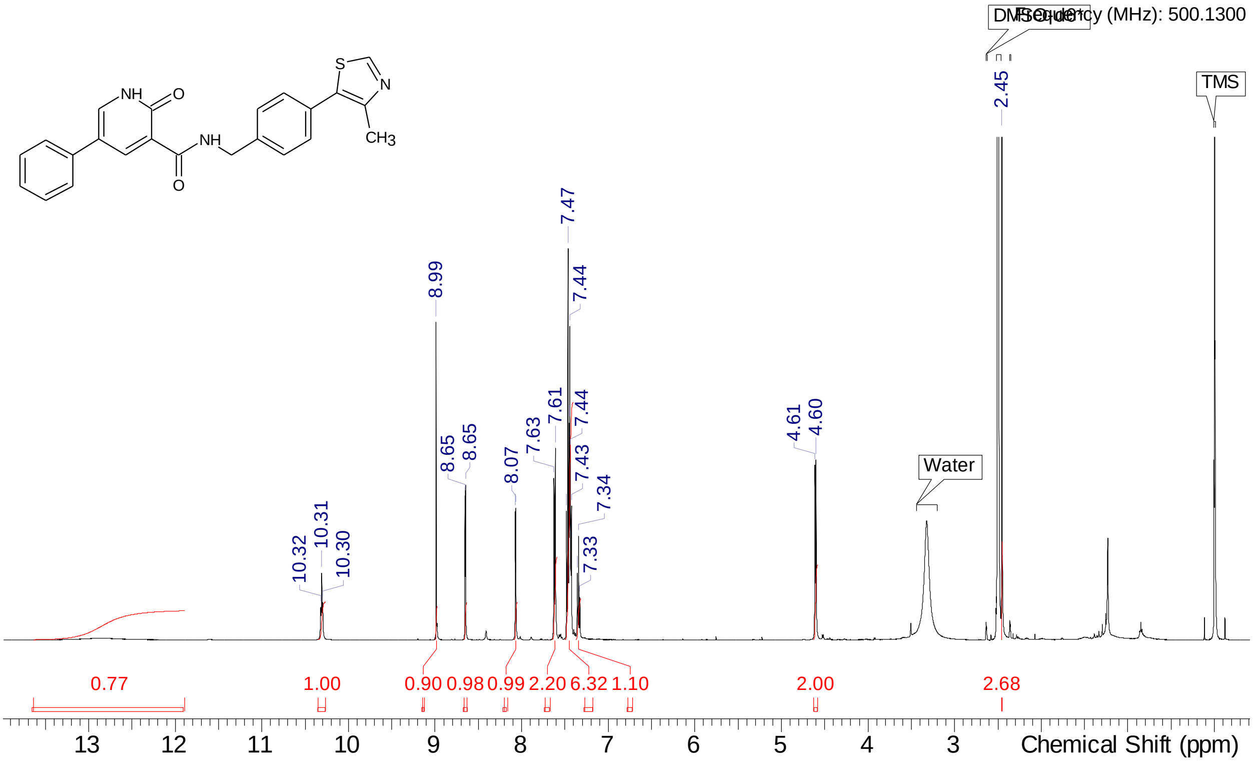
Frequency (MHz): 500.1300
DMSO-d6
2.45
TMS
Water
10.32
10.31
10.30
8.99
8.65
8.65
8.07
7.63
7.61
7.47
7.44
7.44
7.43
7.34
7.33
4.61
4.60
0.77
1.00
0.90
0.98
0.99
2.20
6.32
1.10
2.00
2.68
Chemical Shift (ppm)
NH
O
NH
O
S
N
CH3

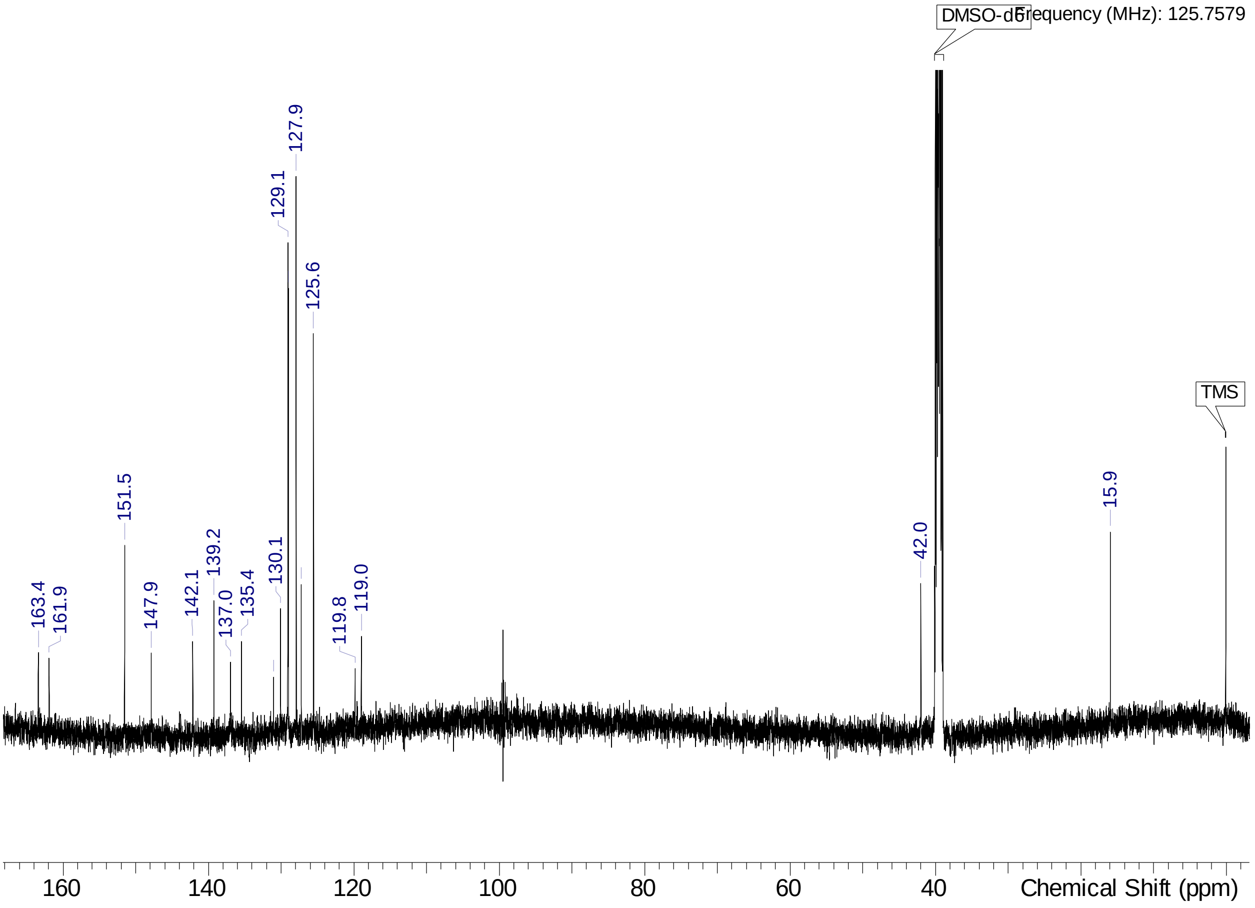
DMSO-d6
Frequency (MHz): 125.7579
TMS
163.4
161.9
151.5
147.9
142.1
139.2
137.0
135.4
130.1
129.1
127.9
125.6
119.8
119.0
42.0
15.9
Chemical Shift (ppm)

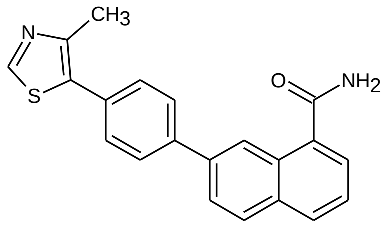
CH3
N
S
O
NH2

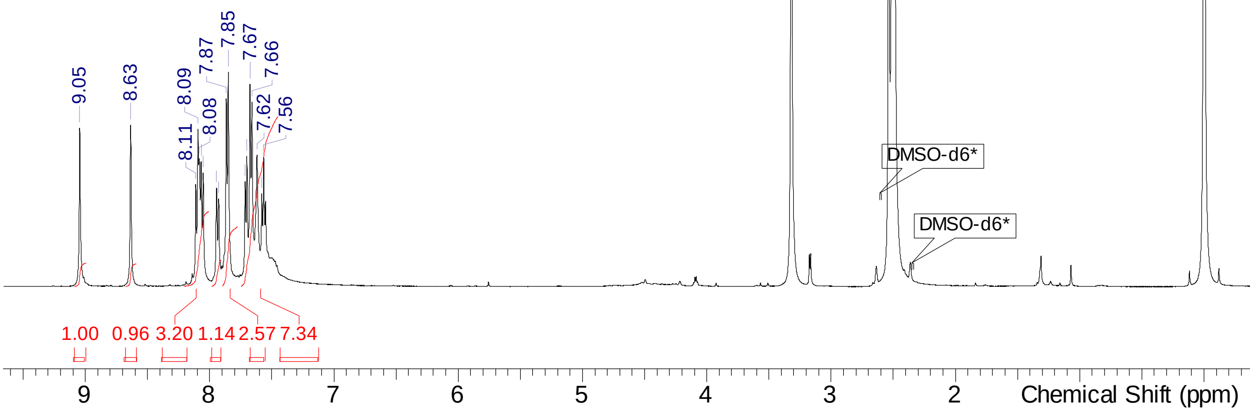
Water
DMSO-d6
Frequency (MHz): 500.1330
TMS
DMSO-d6*
DMSO-d6*
9.05
8.63
8.11
8.09
8.08
7.87
7.85
7.67
7.66
7.62
7.56
1.00
0.96
3.20
1.14
2.57
7.34
9
8
7
6
5
4
3
2
Chemical Shift (ppm)

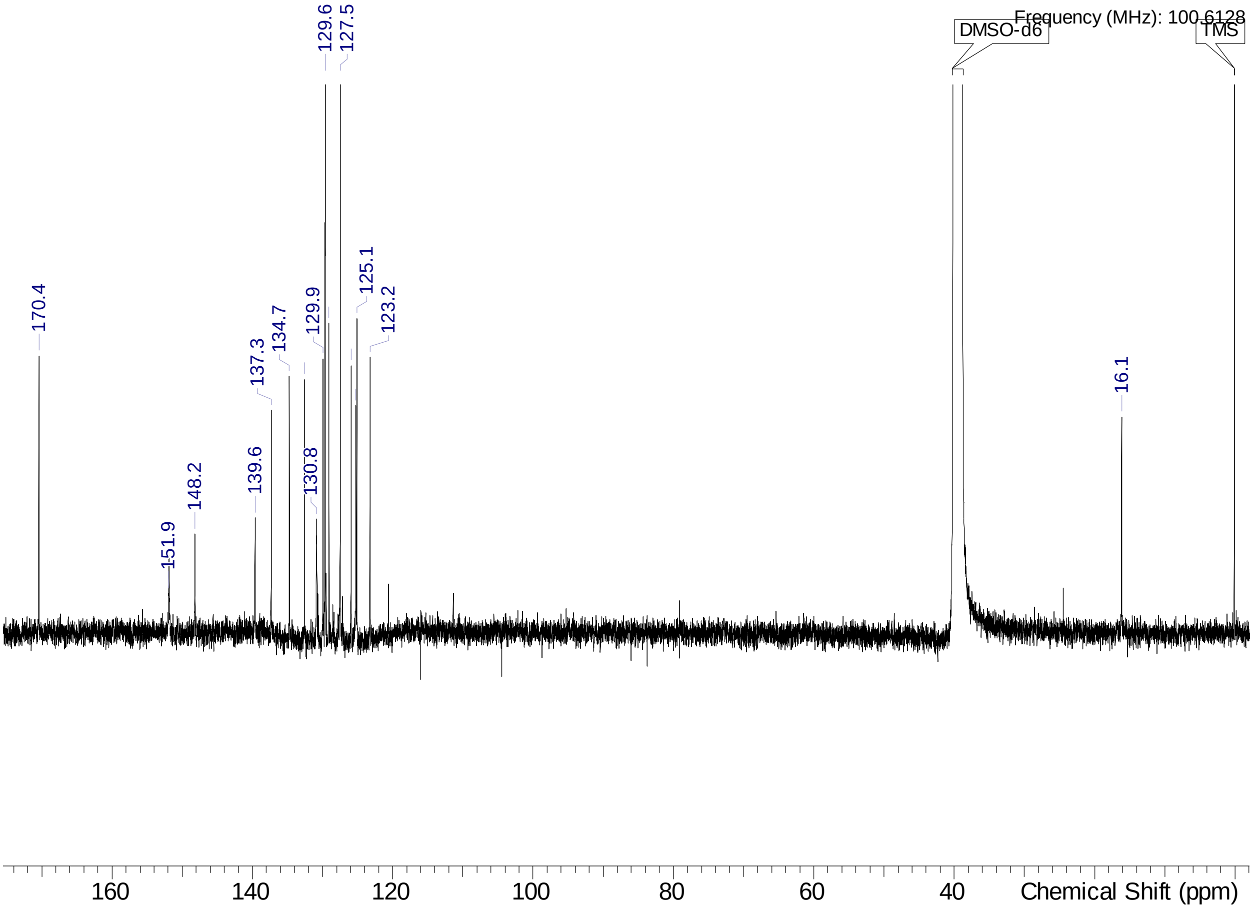
Frequency (MHz): 100.6128
DMSO-d6
TMS
170.4
151.9
148.2
139.6
137.3
134.7
130.8
129.9
129.6
127.5
125.1
123.2
16.1
160
140
120
100
80
60
40
Chemical Shift (ppm)

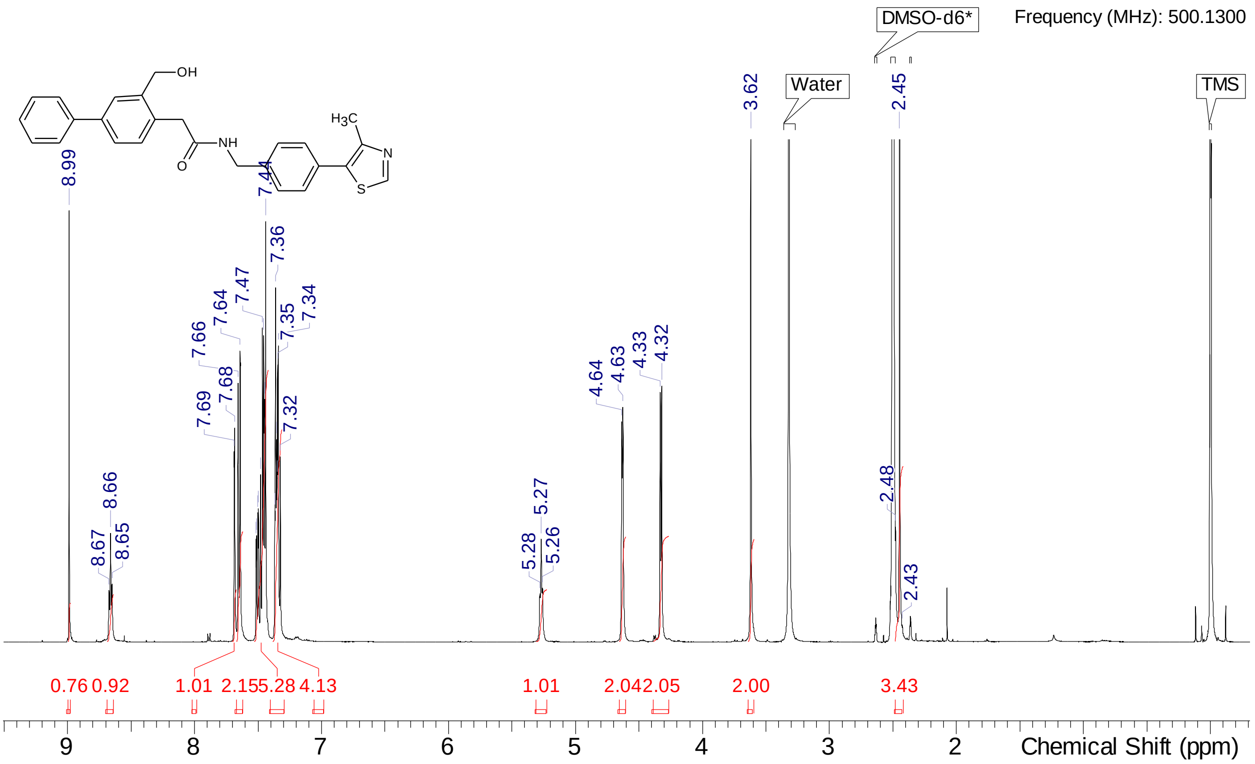
DMSO-d6*
Frequency (MHz): 500.1300
OH
NH
O
H3C
N
S
Water
TMS
8.99
8.67
8.66
8.65
7.69
7.68
7.66
7.64
7.47
7.44
7.36
7.35
7.34
7.32
5.28
5.27
5.26
4.64
4.63
4.33
4.32
3.62
2.48
2.45
2.43
0.76
0.92
1.01
2.15
5.28
4.13
1.01
2.04
2.05
2.00
3.43
9
8
7
6
5
4
3
2
Chemical Shift (ppm)

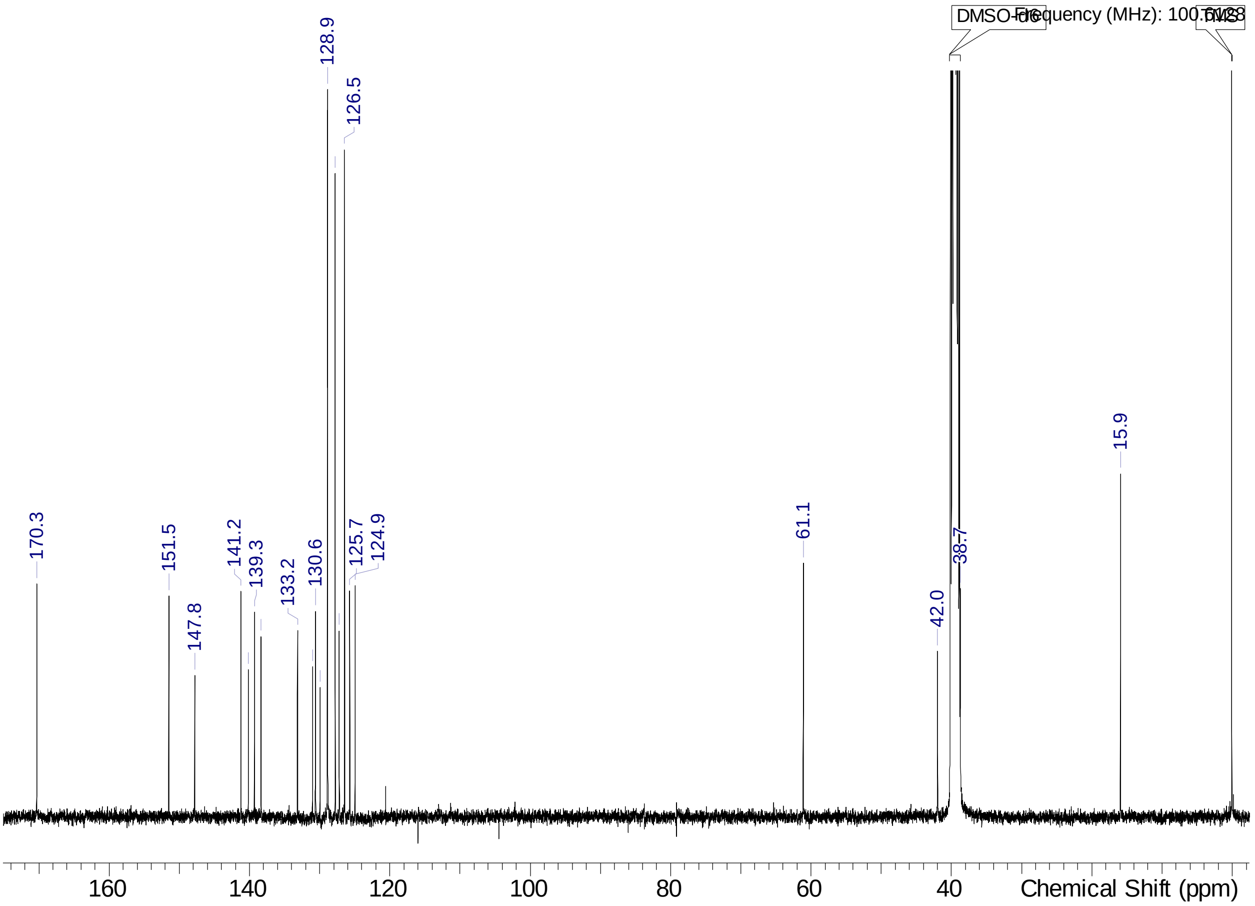
DMSO-d6
Frequency (MHz): 100.6128
TMS
170.3
151.5
147.8
141.2
139.3
133.2
130.6
128.9
126.5
125.7
124.9
61.1
42.0
38.7
15.9
160
140
120
100
80
60
40
Chemical Shift (ppm)

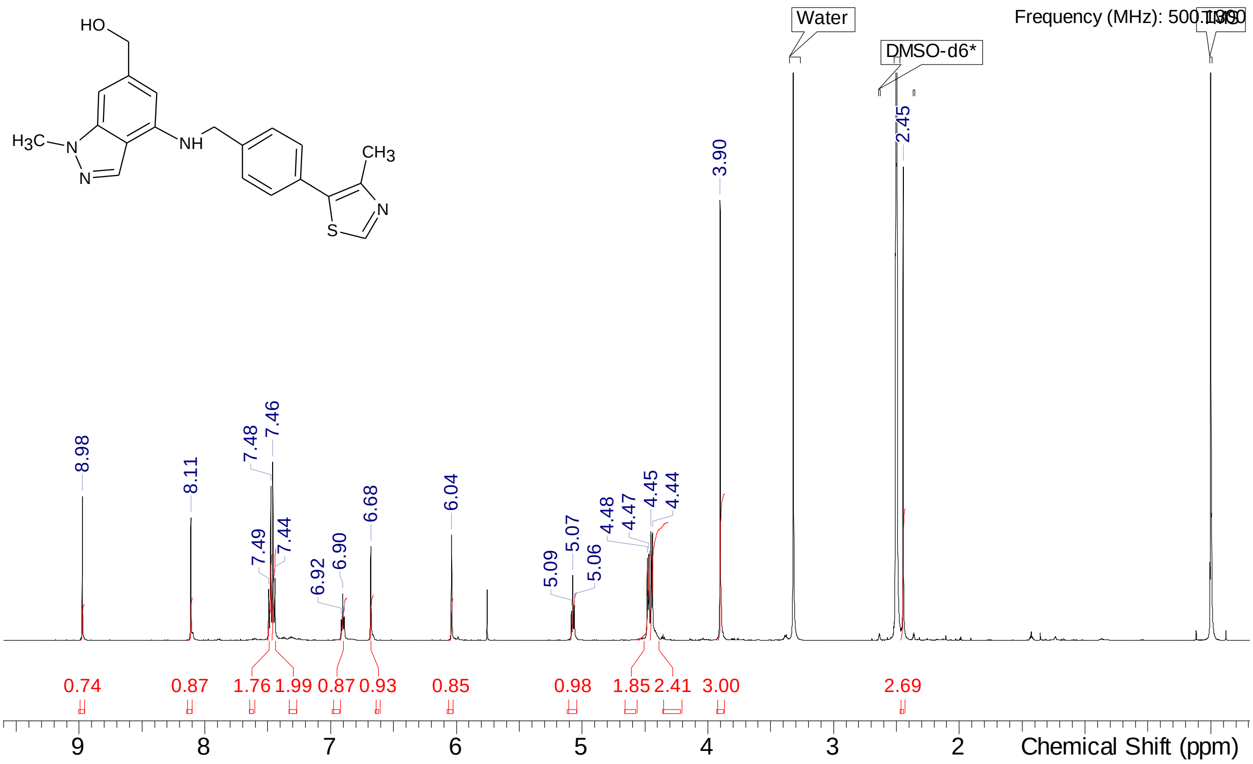
HO
H3C
N
N
NH
CH3
N
S
Water
DMSO-d6*
TMS
Frequency (MHz): 500.1300
8.98
8.11
7.49
7.48
7.46
7.44
6.92
6.90
6.68
6.04
5.09
5.07
5.06
4.48
4.47
4.45
4.44
3.90
2.45
0.74
0.87
1.76
1.99
0.87
0.93
0.85
0.98
1.85
2.41
3.00
2.69
9
8
7
6
5
4
3
2
Chemical Shift (ppm)

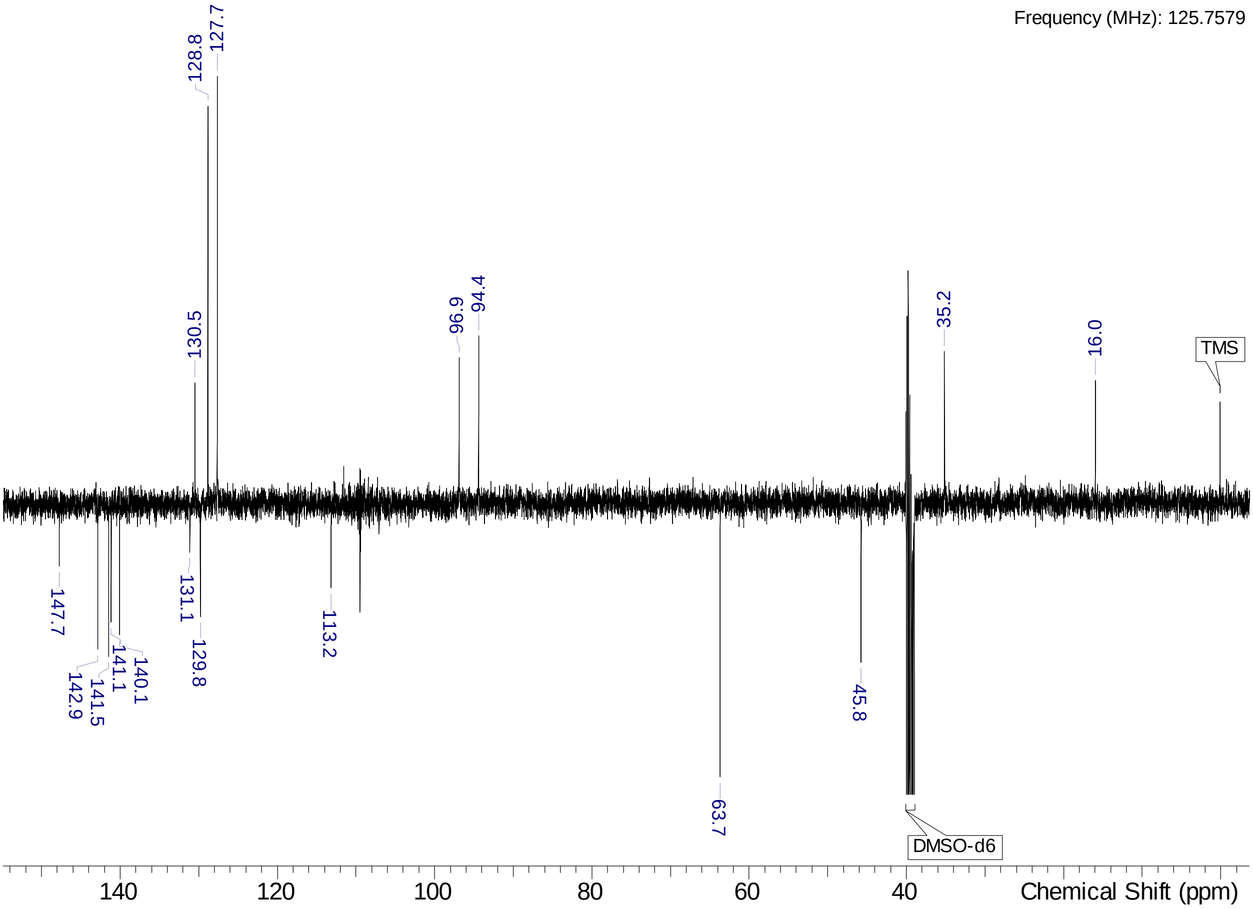
Frequency (MHz): 125.7579
128.8
127.7
130.5
96.9
94.4
35.2
16.0
TMS
147.7
142.9
141.5
141.1
140.1
131.1
129.8
113.2
63.7
45.8
DMSO-d6
140
120
100
80
60
40
Chemical Shift (ppm)

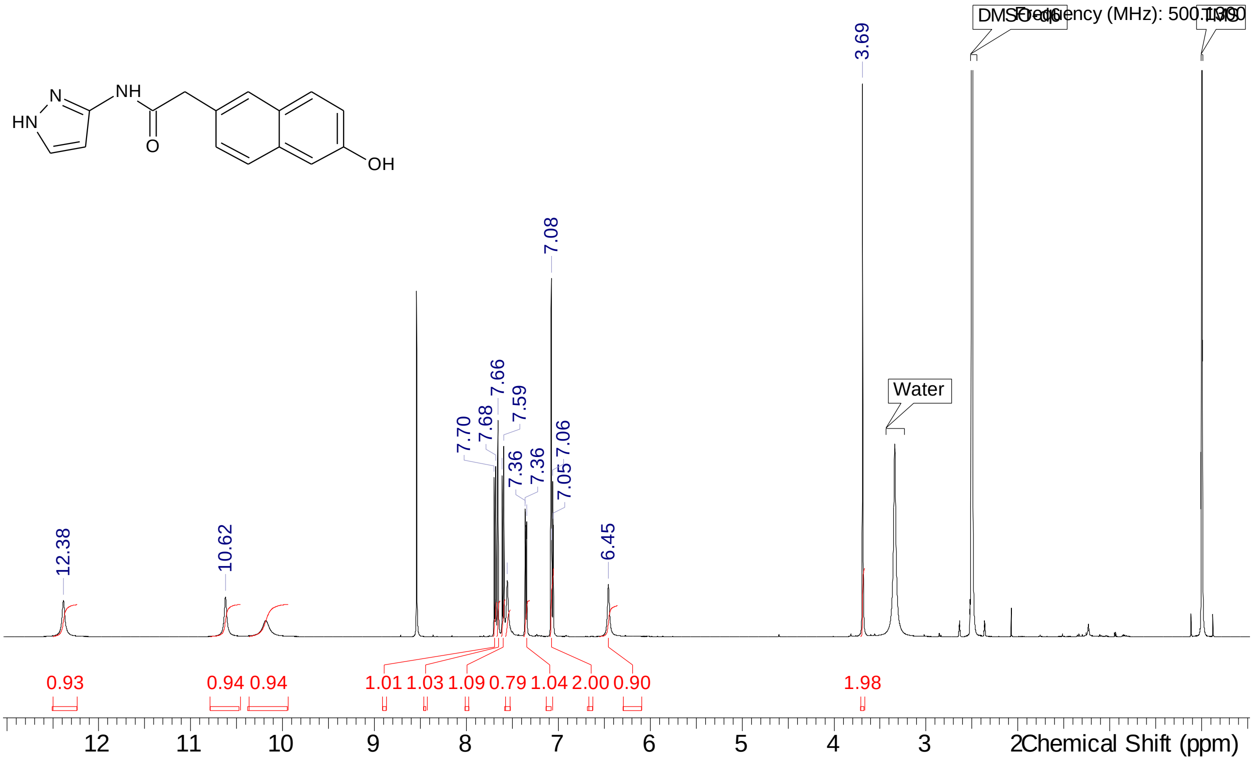

NH
HN
N
O
OH
Frequency (MHz): 500.1330
DMSO-d6
TMS
Water
12.38
10.62
7.70
7.68
7.66
7.59
7.36
7.36
7.08
7.06
7.05
6.45
3.69
0.93
0.94
0.94
1.01
1.03
1.09
0.79
1.04
2.00
0.90
1.98
12
11
10
9
8
7
6
5
4
3
2
Chemical Shift (ppm)

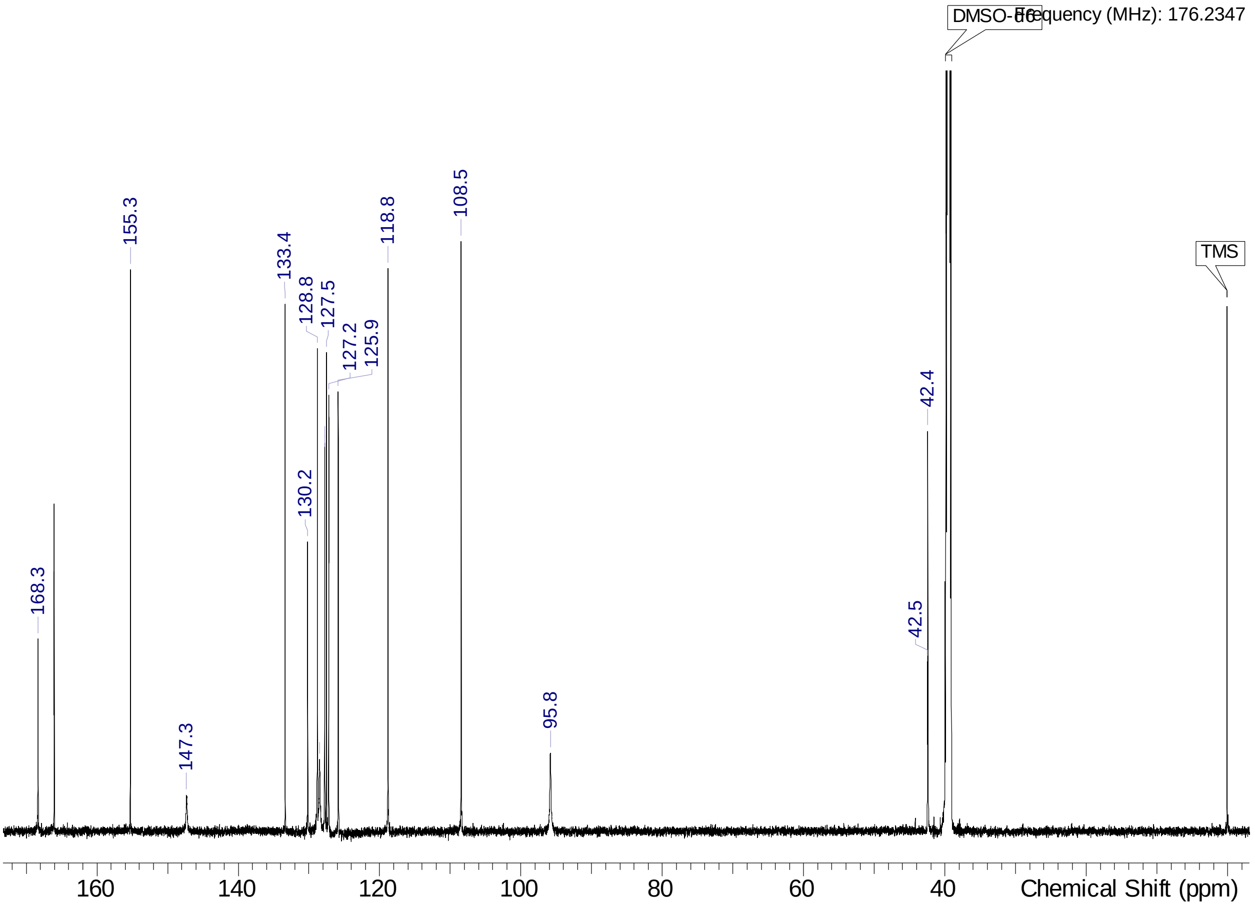

Frequency (MHz): 176.2347
DMSO-d6
TMS
168.3
155.3
147.3
133.4
130.2
128.8
127.5
127.2
125.9
118.8
108.5
95.8
42.5
42.4
160
140
120
100
80
60
40
Chemical Shift (ppm)

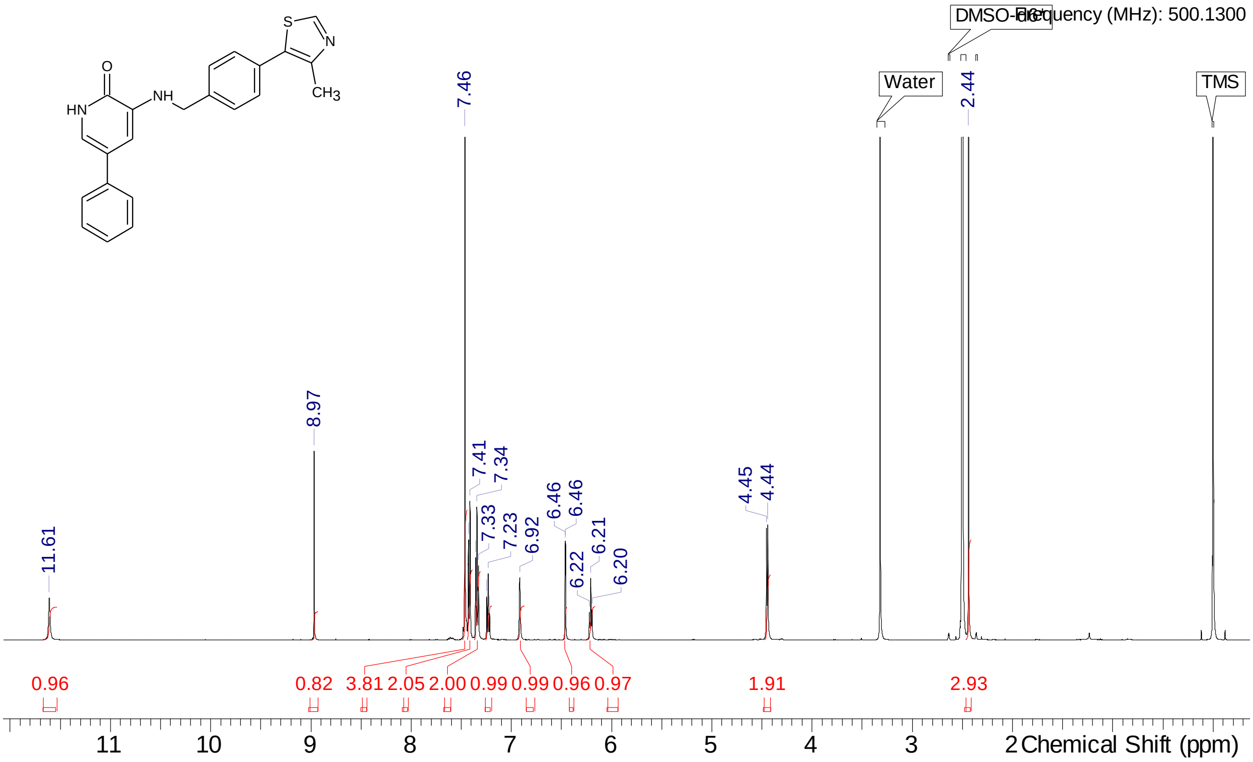
Frequency (MHz): 500.1300
DMSO-d6
Water
TMS
O
HN
NH
S
N
CH3
11.61
8.97
7.46
7.41
7.34
7.33
7.23
6.92
6.46
6.46
6.22
6.21
6.20
4.45
4.44
2.44
0.96
0.82
3.81
2.05
2.00
0.99
0.99
0.96
0.97
1.91
2.93
11
10
9
8
7
6
5
4
3
2
Chemical Shift (ppm)

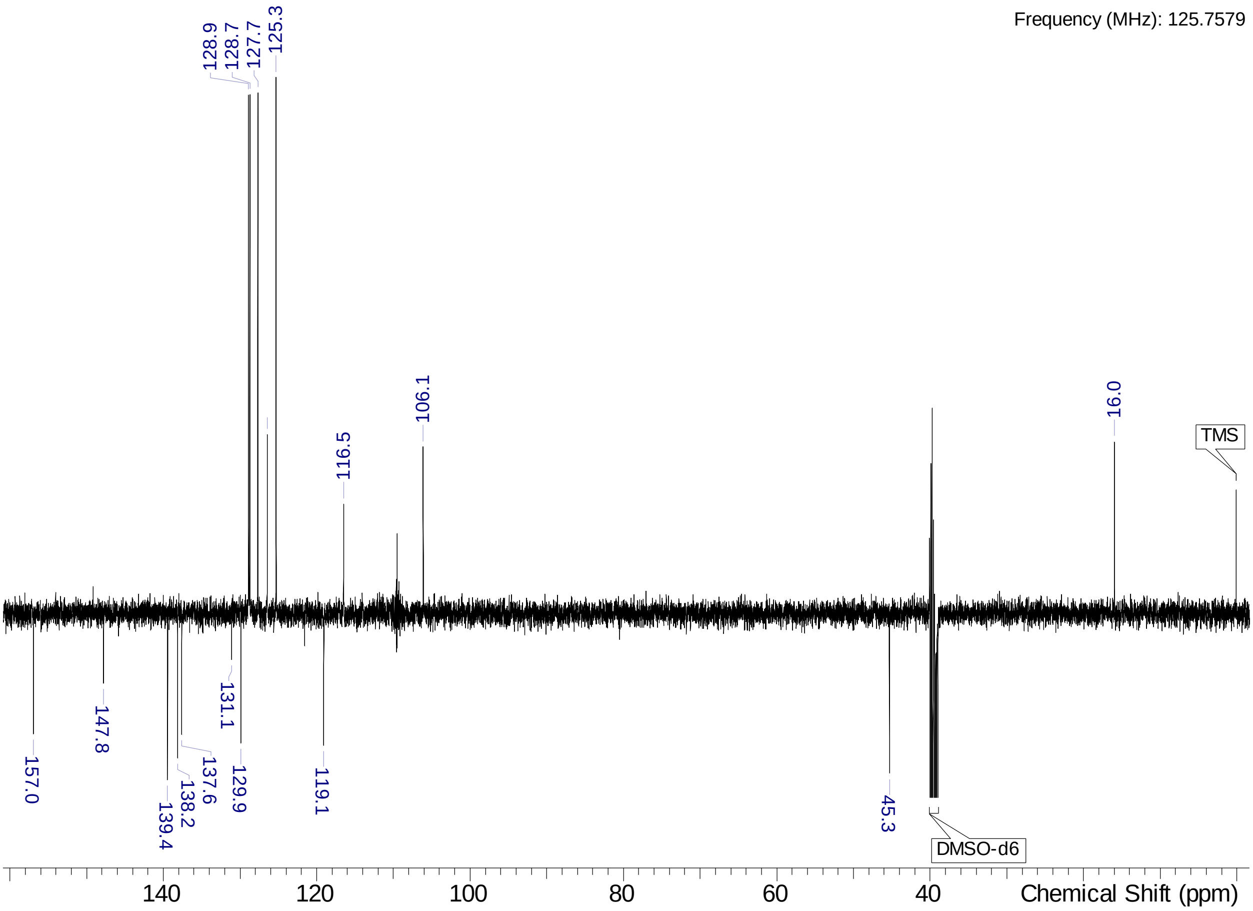
Frequency (MHz): 125.7579
128.9
128.7
127.7
125.3
116.5
106.1
16.0
TMS
157.0
147.8
139.4
138.2
137.6
131.1
129.9
119.1
45.3
DMSO-d6
140
120
100
80
60
40
Chemical Shift (ppm)

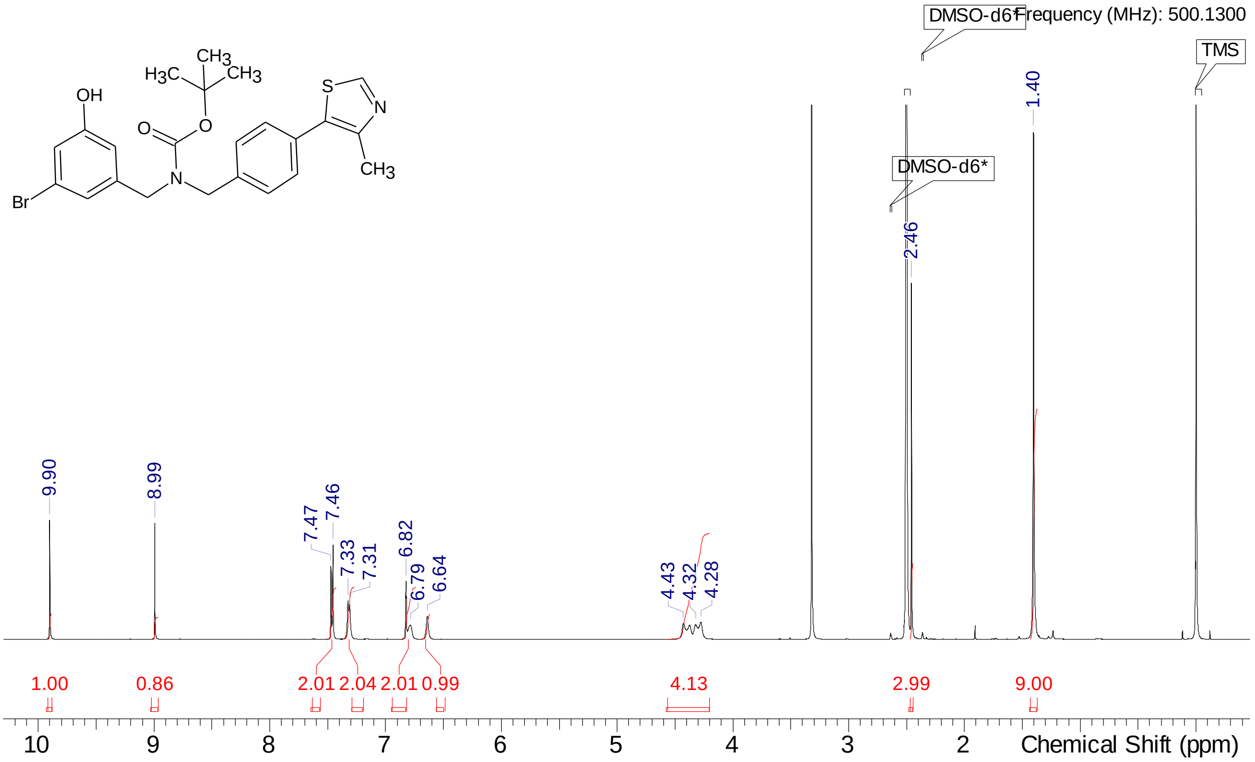
DMSO-d6*
Frequency (MHz): 500.1300
TMS
DMSO-d6*
OH
Br
H3C
CH3
CH3
O
O
N
S
N
CH3
9.90
8.99
7.47
7.46
7.33
7.31
6.82
6.79
6.64
4.43
4.32
4.28
2.46
1.40
1.00
0.86
2.01
2.04
2.01
0.99
4.13
2.99
9.00
10
9
8
7
6
5
4
3
2
Chemical Shift (ppm)

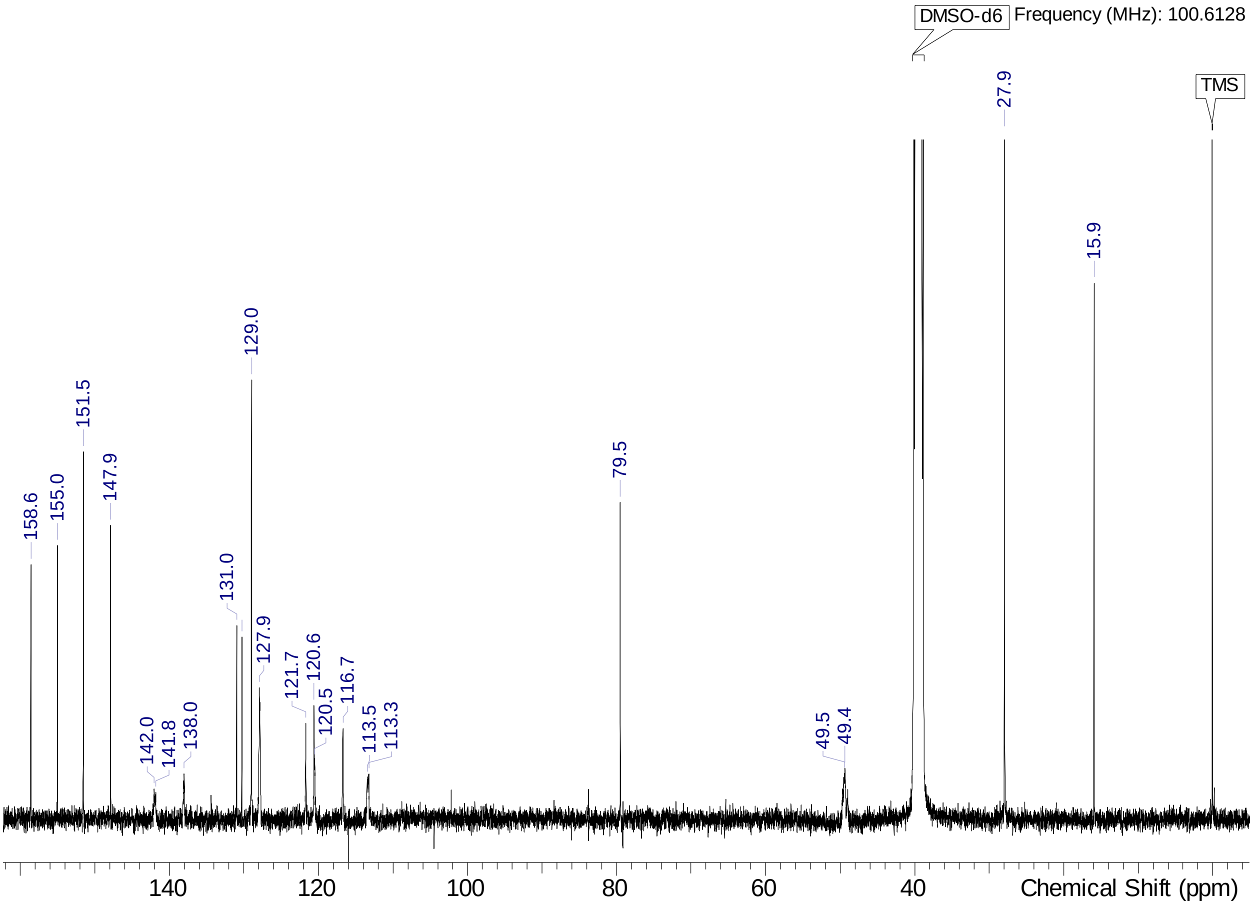
DMSO-d6
Frequency (MHz): 100.6128
TMS
158.6
155.0
151.5
147.9
142.0
141.8
138.0
131.0
129.0
127.9
121.7
120.6
120.5
116.7
113.5
113.3
79.5
49.5
49.4
27.9
15.9
140
120
100
80
60
40
Chemical Shift (ppm)

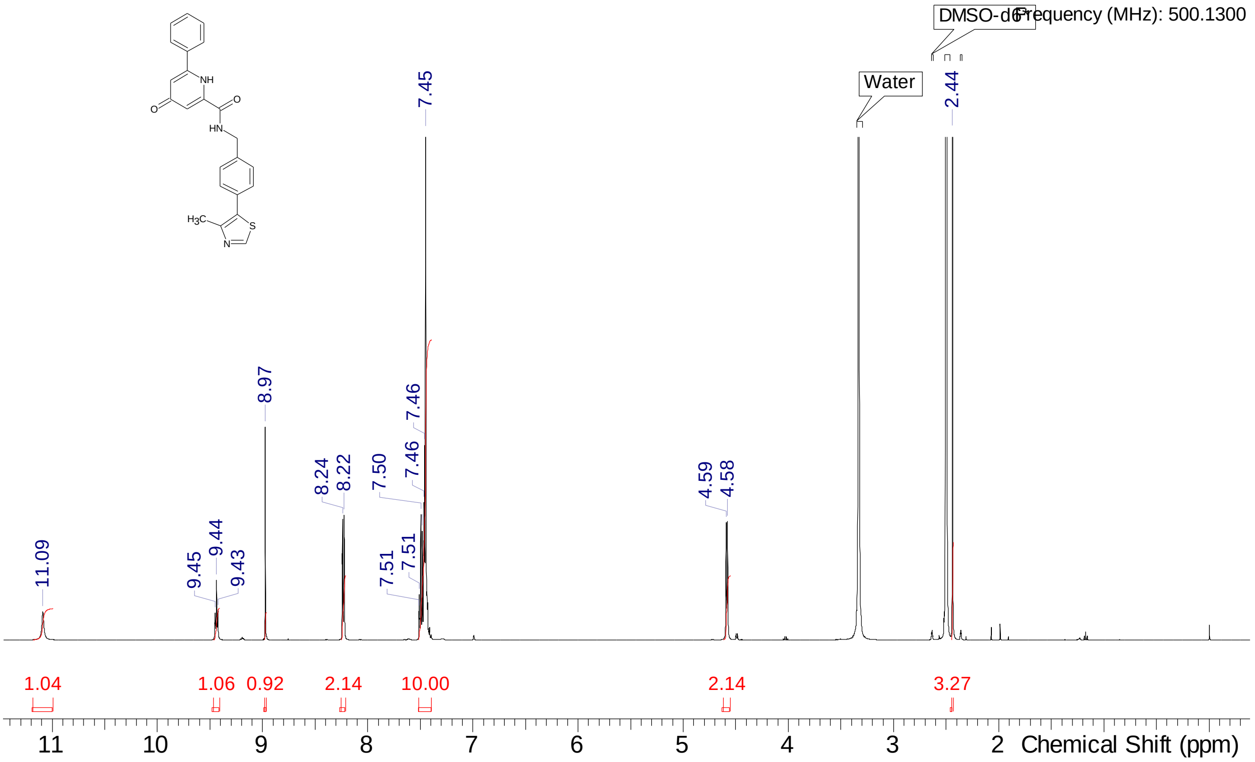
DMSO-d6
Frequency (MHz): 500.1300
Water
NH
O
HN
H3C
S
N
11.09
9.45
9.44
9.43
8.97
8.24
8.22
7.51
7.51
7.50
7.46
7.46
7.45
4.59
4.58
2.44
1.04
1.06
0.92
2.14
10.00
2.14
3.27
11
10
9
8
7
6
5
4
3
2
Chemical Shift (ppm)

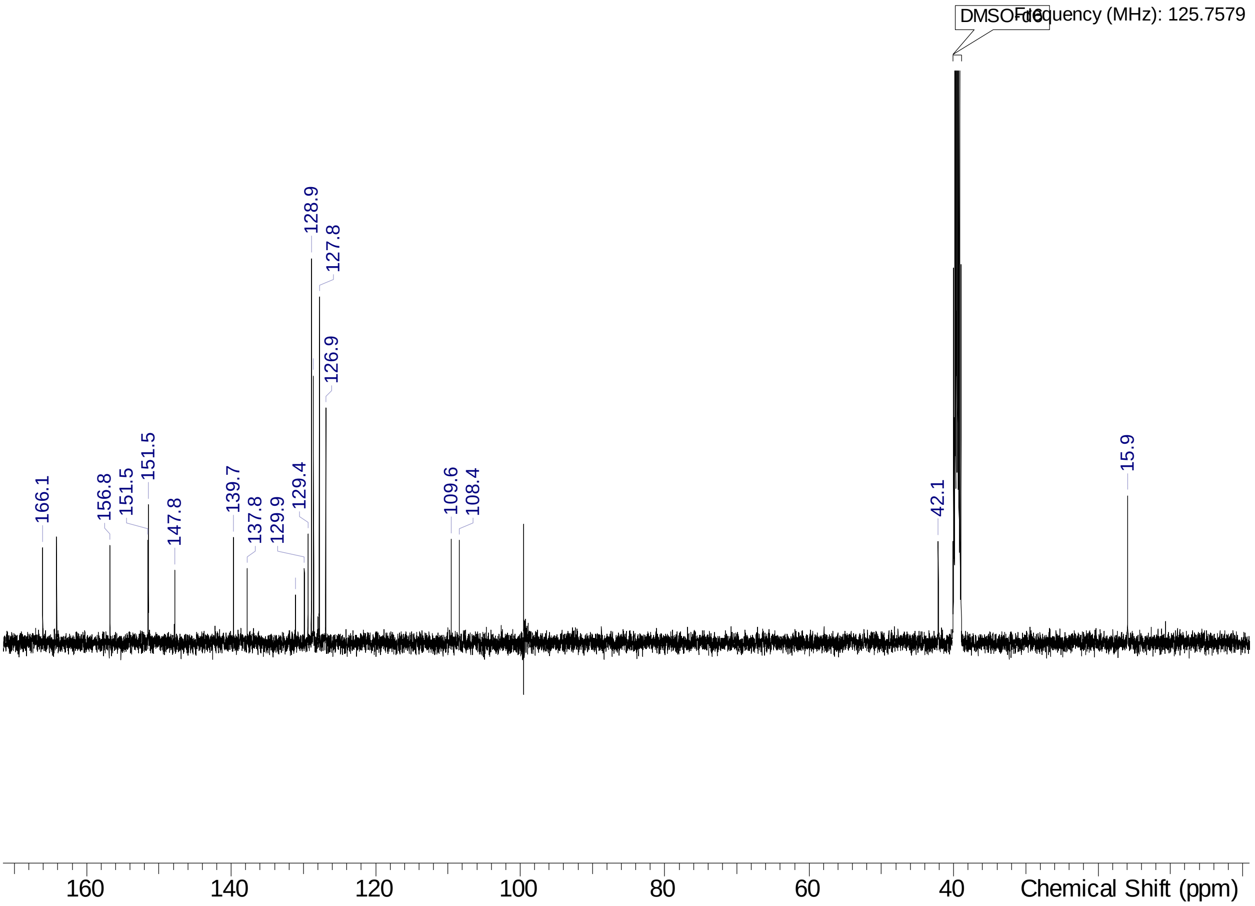
DMSO-d6
Frequency (MHz): 125.7579
166.1
156.8
151.5
151.5
147.8
139.7
137.8
129.9
129.4
128.9
127.8
126.9
109.6
108.4
42.1
15.9
160
140
120
100
80
60
40
Chemical Shift (ppm)

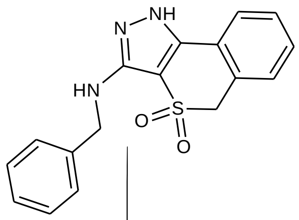
N
NH
HN
O
S
O

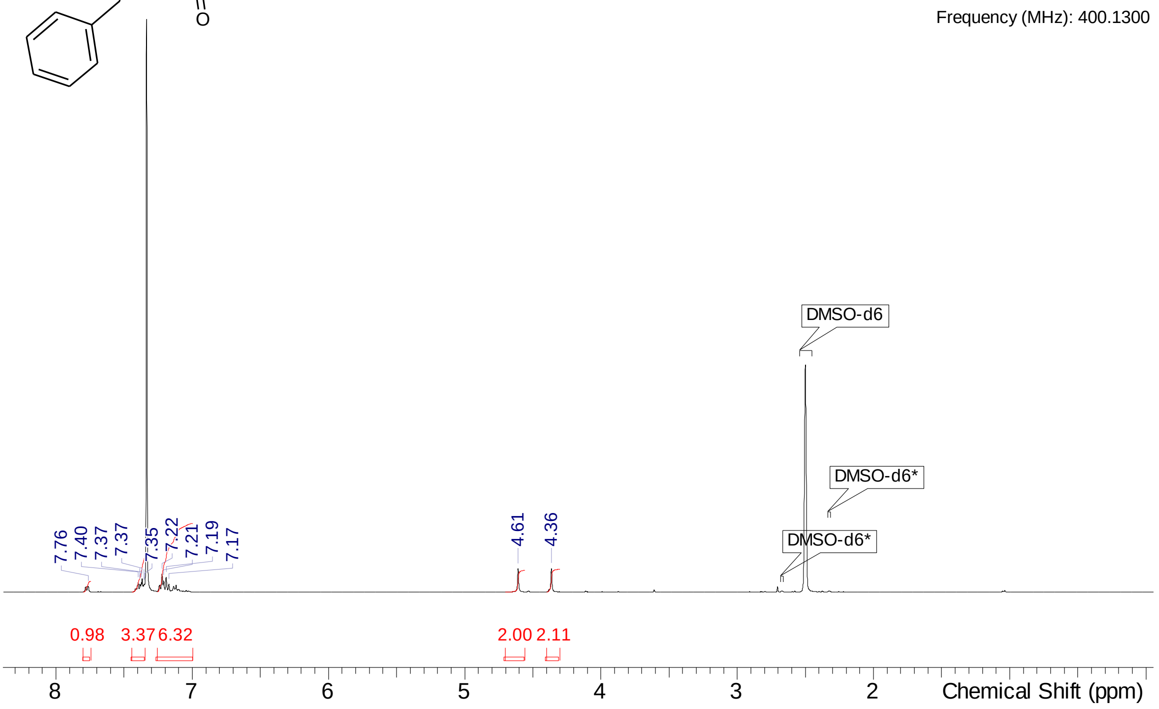
Frequency (MHz): 400.1300
7.76
7.40
7.37
7.37
7.35
7.22
7.21
7.19
7.17
4.61
4.36
DMSO-d6
DMSO-d6*
DMSO-d6*
0.98
3.37
6.32
2.00
2.11
8
7
6
5
4
3
2
Chemical Shift (ppm)

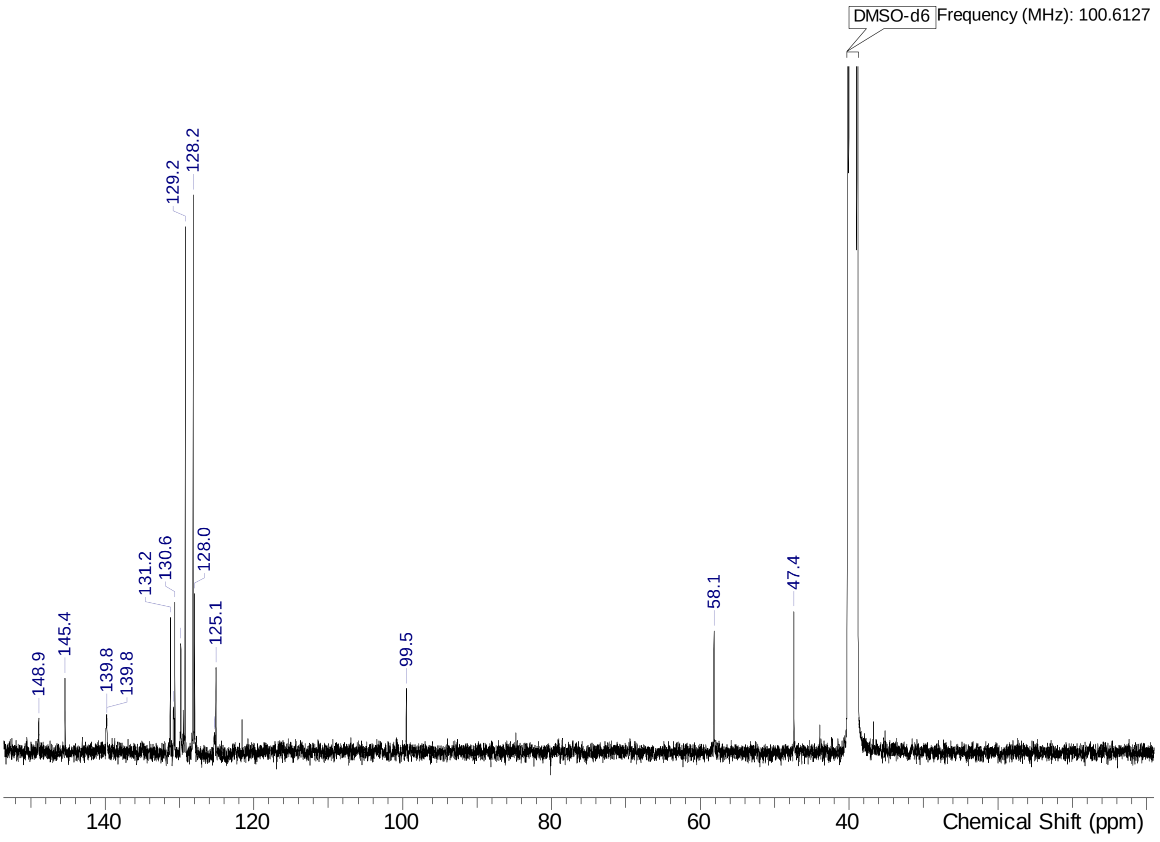
DMSO-d6
Frequency (MHz): 100.6127
129.2
128.2
148.9
145.4
139.8
139.8
131.2
130.6
128.0
125.1
99.5
58.1
47.4
140
120
100
80
60
40
Chemical Shift (ppm)

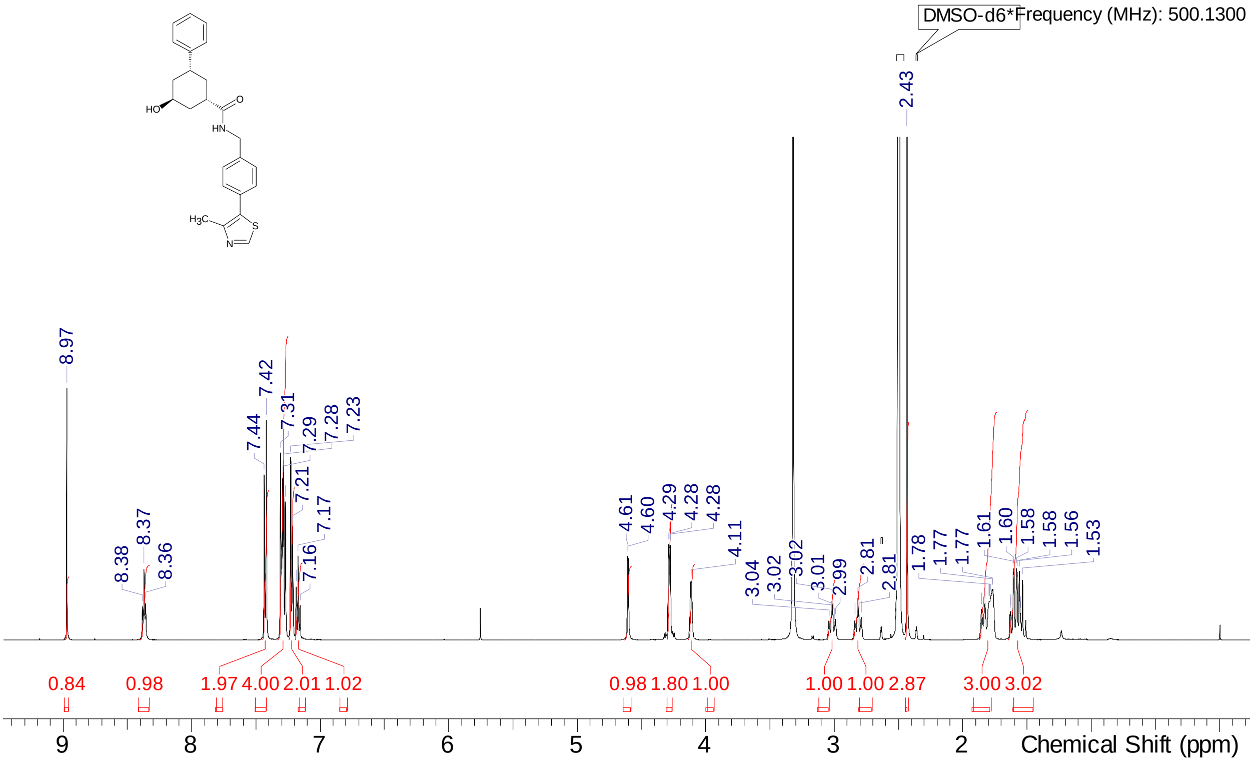
DMSO-d6*Frequency (MHz): 500.1300
HO
HN
O
H_3C
S
N
8.97
8.38 8.37 8.36
7.44 7.42 7.31 7.29 7.28 7.23 7.21 7.17 7.16
4.61 4.60 4.29 4.28 4.28 4.11
3.04 3.02 3.02 3.01 2.99 2.81 2.81
2.43
1.78 1.77 1.77 1.61 1.60 1.58 1.58 1.56 1.53
0.84 0.98 1.97 4.00 2.01 1.02 0.98 1.80 1.00 1.00 1.00 2.87 3.00 3.02
9 8 7 6 5 4 3 2
Chemical Shift (ppm)

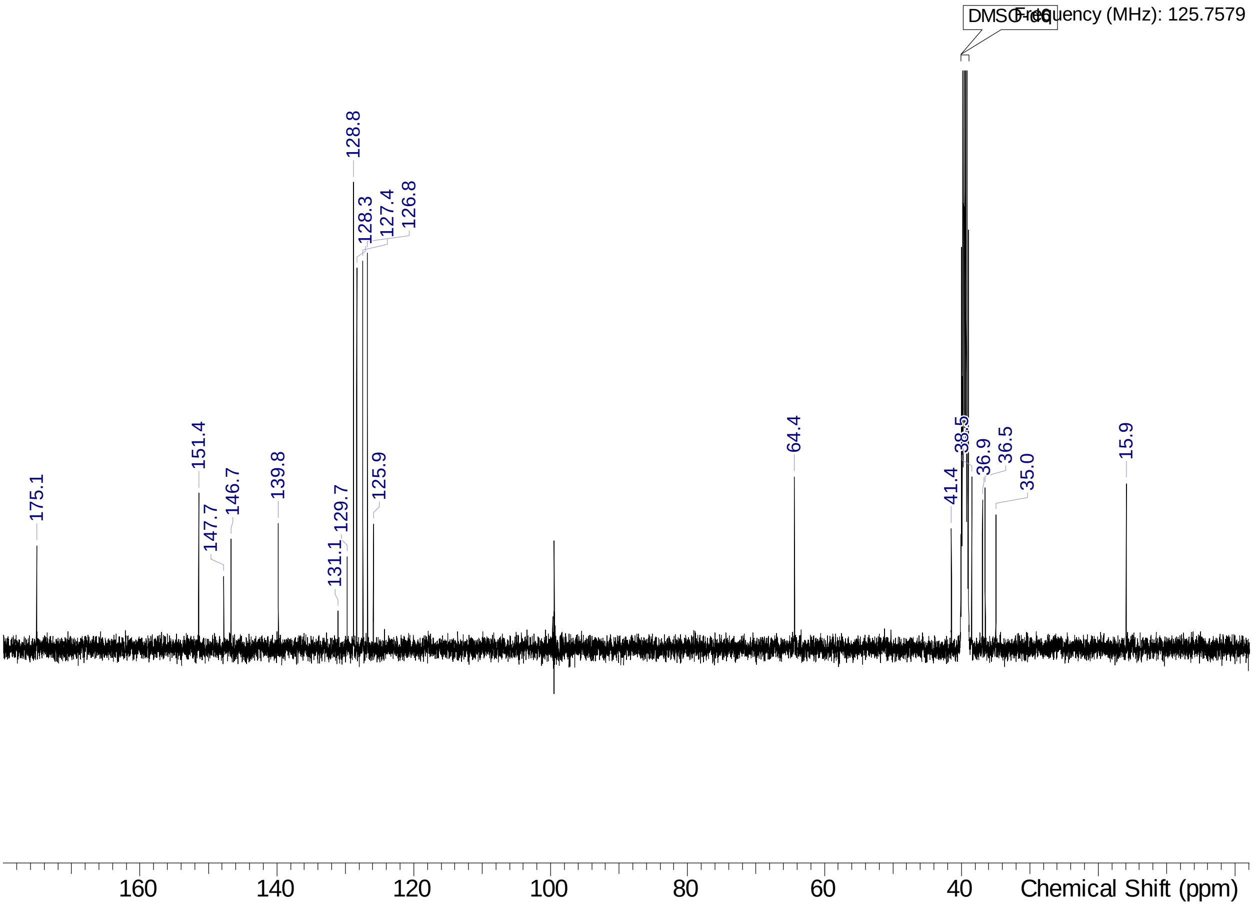
DMSO-d6 Frequency (MHz): 125.7579
175.1
151.4 147.7 146.7
139.8
131.1 129.7 128.8 128.3 127.4 126.8 125.9
64.4
41.4 38.5 36.9 36.5 35.0
15.9
160 140 120 100 80 60 40
Chemical Shift (ppm)

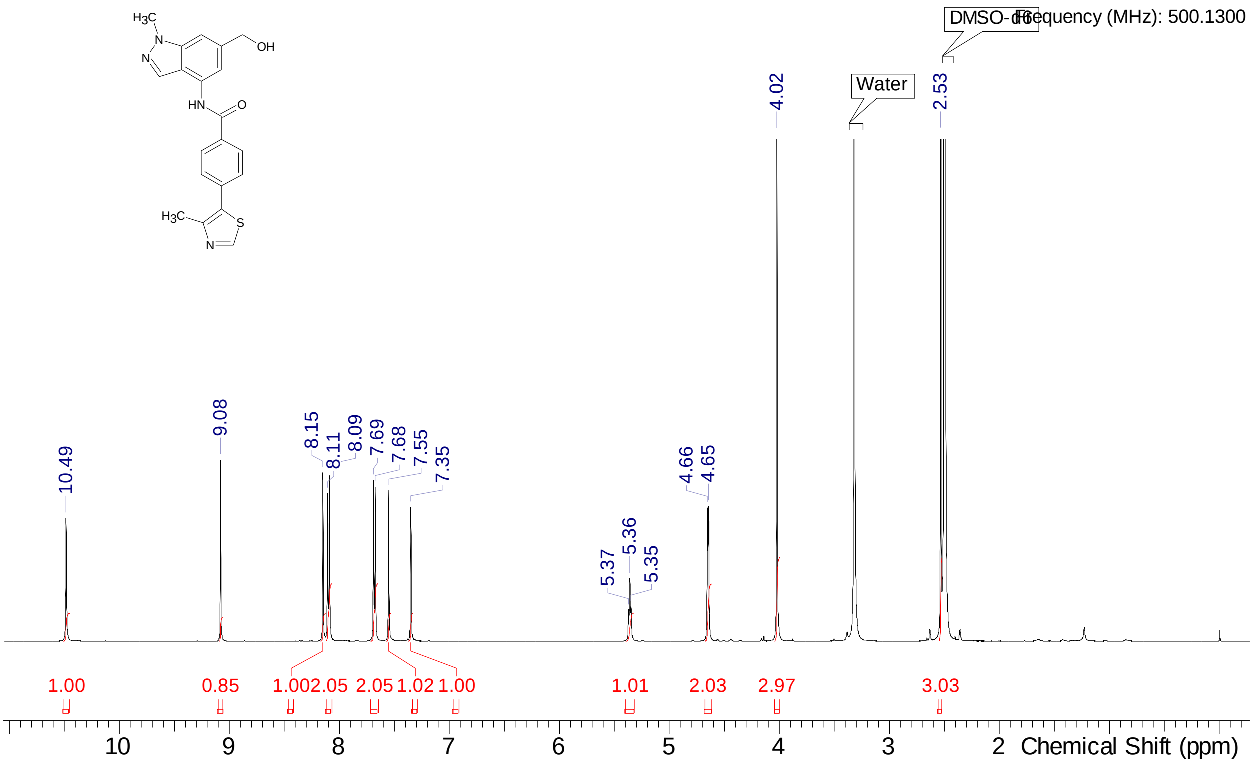
DMSO-d6
Frequency (MHz): 500.1300
Water
10.49
9.08
8.15
8.11
8.09
7.69
7.68
7.55
7.35
5.37
5.36
5.35
4.66
4.65
4.02
2.53
1.00
0.85
1.00
2.05
2.05
1.02
1.00
1.01
2.03
2.97
3.03
10
9
8
7
6
5
4
3
2
Chemical Shift (ppm)

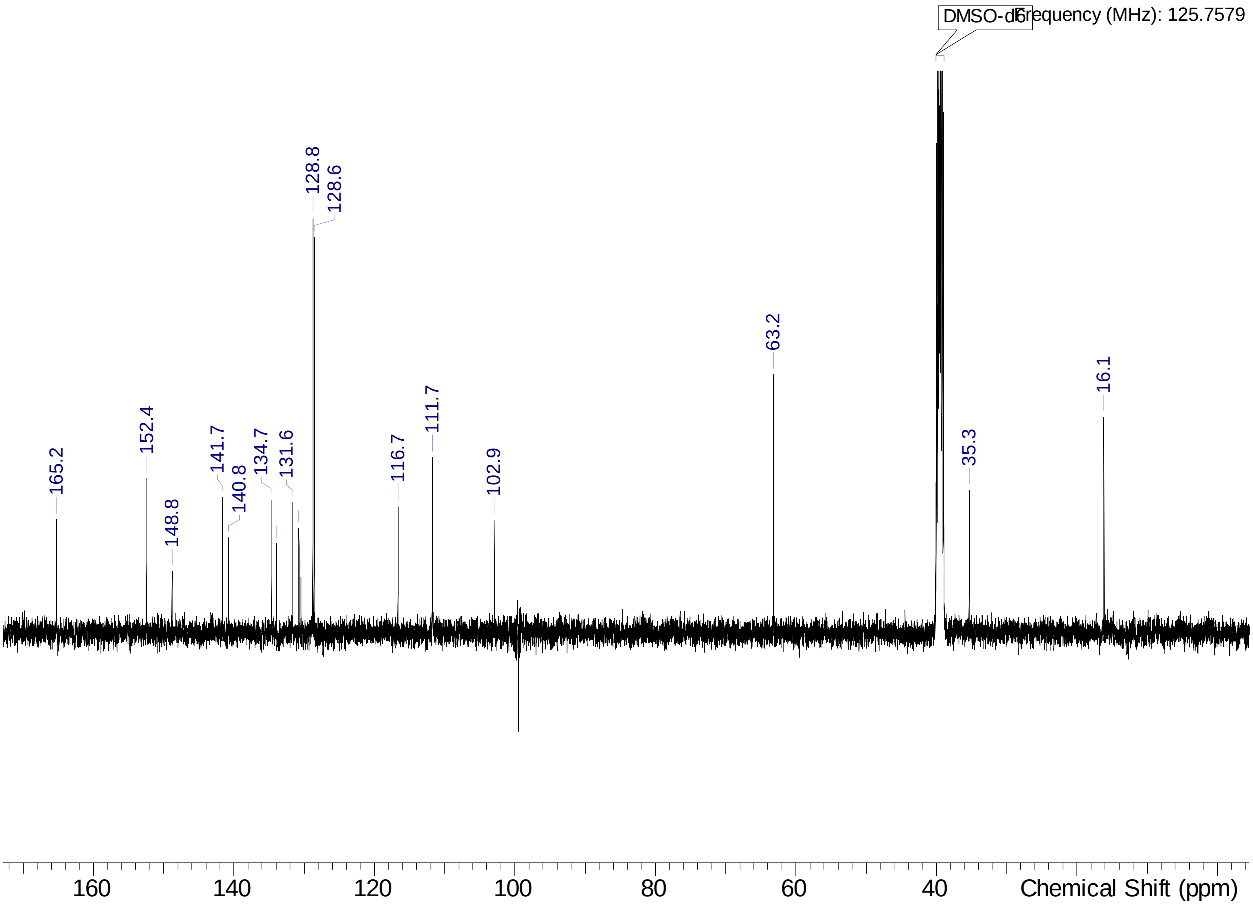
DMSO-d6
Frequency (MHz): 125.7579
165.2
152.4
148.8
141.7
140.8
134.7
131.6
128.8
128.6
116.7
111.7
102.9
63.2
35.3
16.1
160
140
120
100
80
60
40
Chemical Shift (ppm)

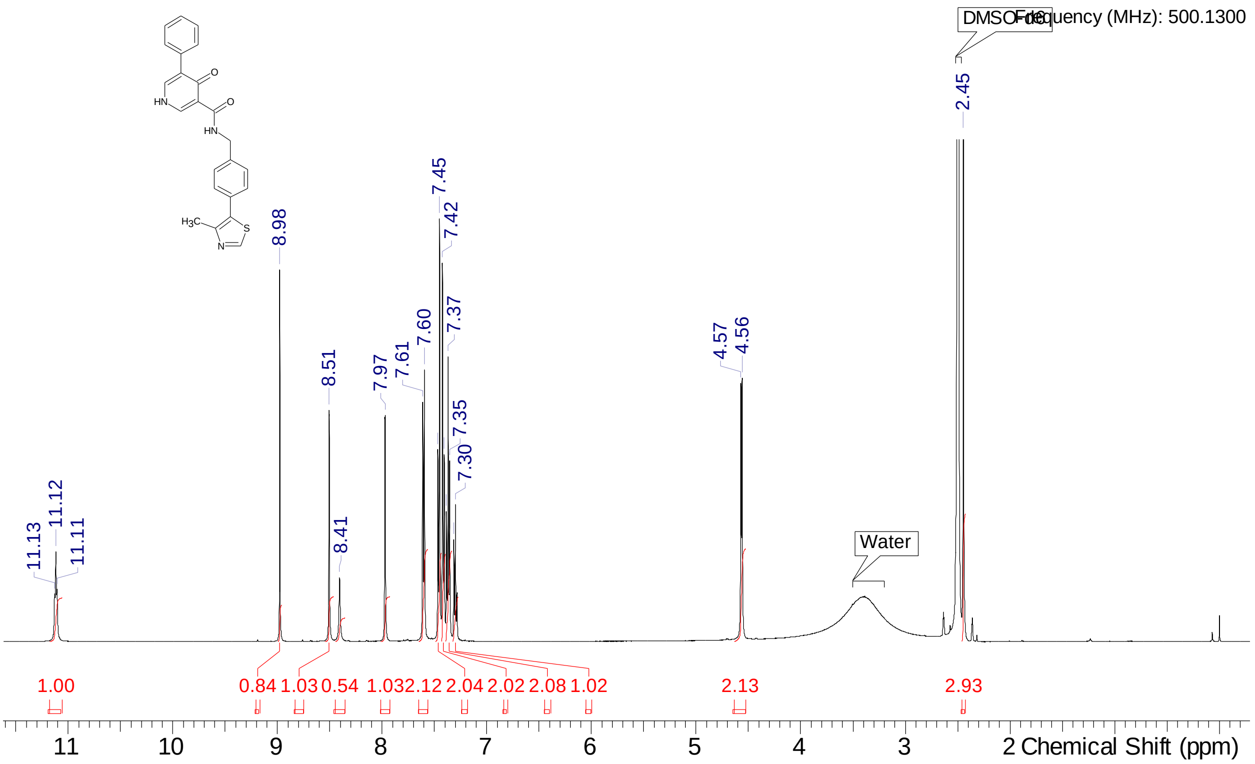
DMSO-d6
Frequency (MHz): 500.1300
2.45
11.13
11.12
11.11
8.98
8.51
8.41
7.97
7.61
7.60
7.45
7.42
7.37
7.35
7.30
4.57
4.56
Water
1.00
0.84
1.03
0.54
1.03
2.12
2.04
2.02
2.08
1.02
2.13
2.93
11
10
9
8
7
6
5
4
3
2
Chemical Shift (ppm)
HN
O
O
HN
H3C
S
N

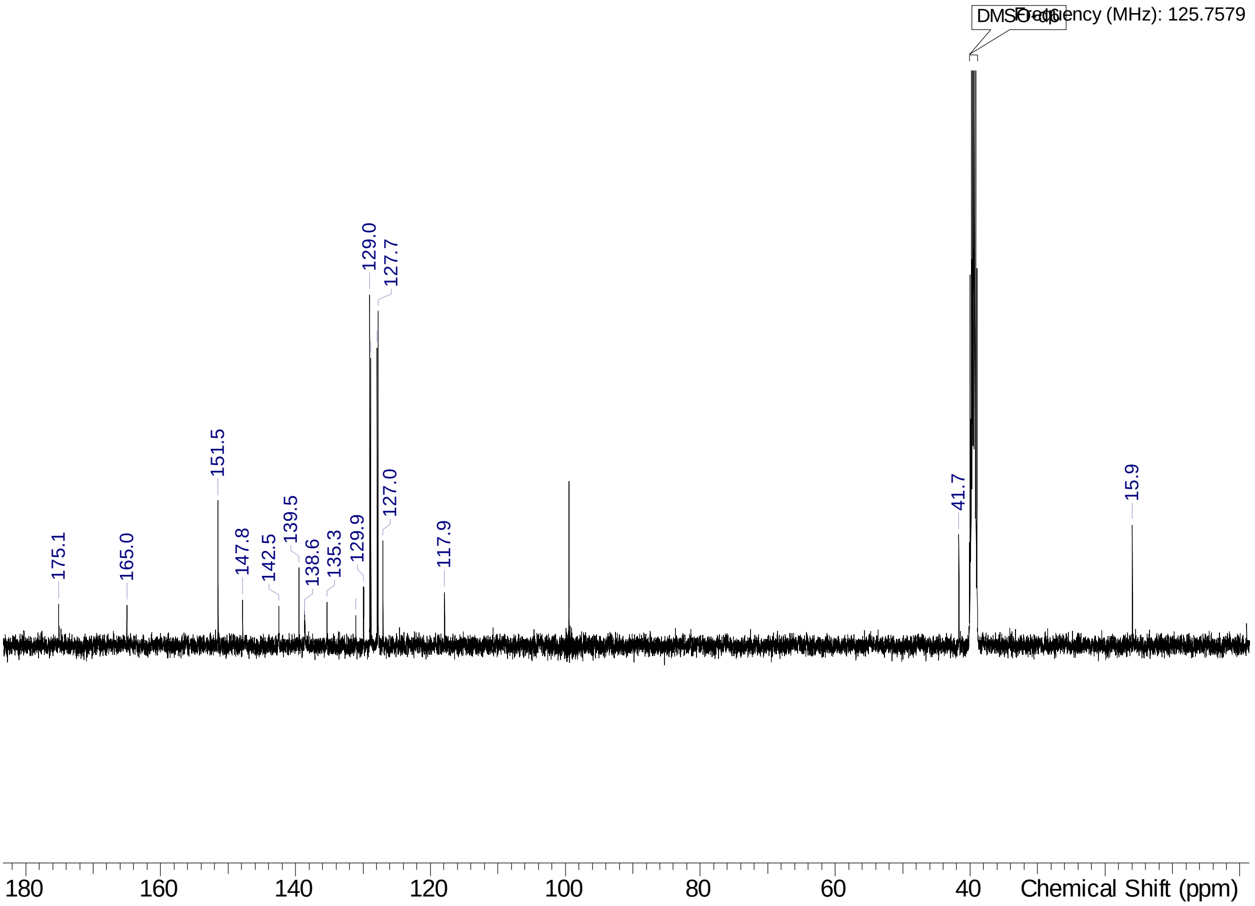
DMSO-d6
Frequency (MHz): 125.7579
175.1
165.0
151.5
147.8
142.5
139.5
138.6
135.3
129.9
129.0
127.7
127.0
117.9
41.7
15.9
180
160
140
120
100
80
60
40
Chemical Shift (ppm)

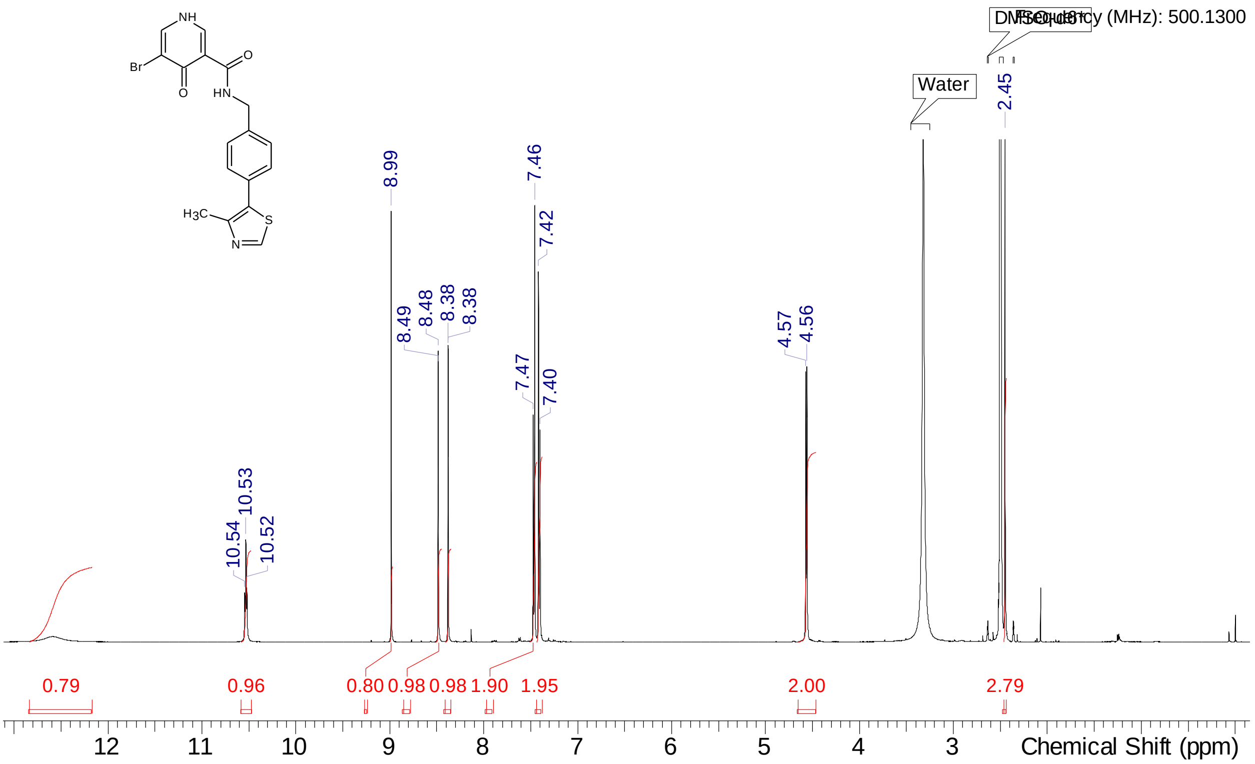
NH
Br
O
O
HN
H3C
S
N
Frequency (MHz): 500.1300
DMSO-d6
Water
2.45
8.99
7.46
7.42
8.49
8.48
8.38
8.38
7.47
7.40
4.57
4.56
10.54
10.53
10.52
0.79
0.96
0.80
0.98
0.98
1.90
1.95
2.00
2.79
12
11
10
9
8
7
6
5
4
3
Chemical Shift (ppm)

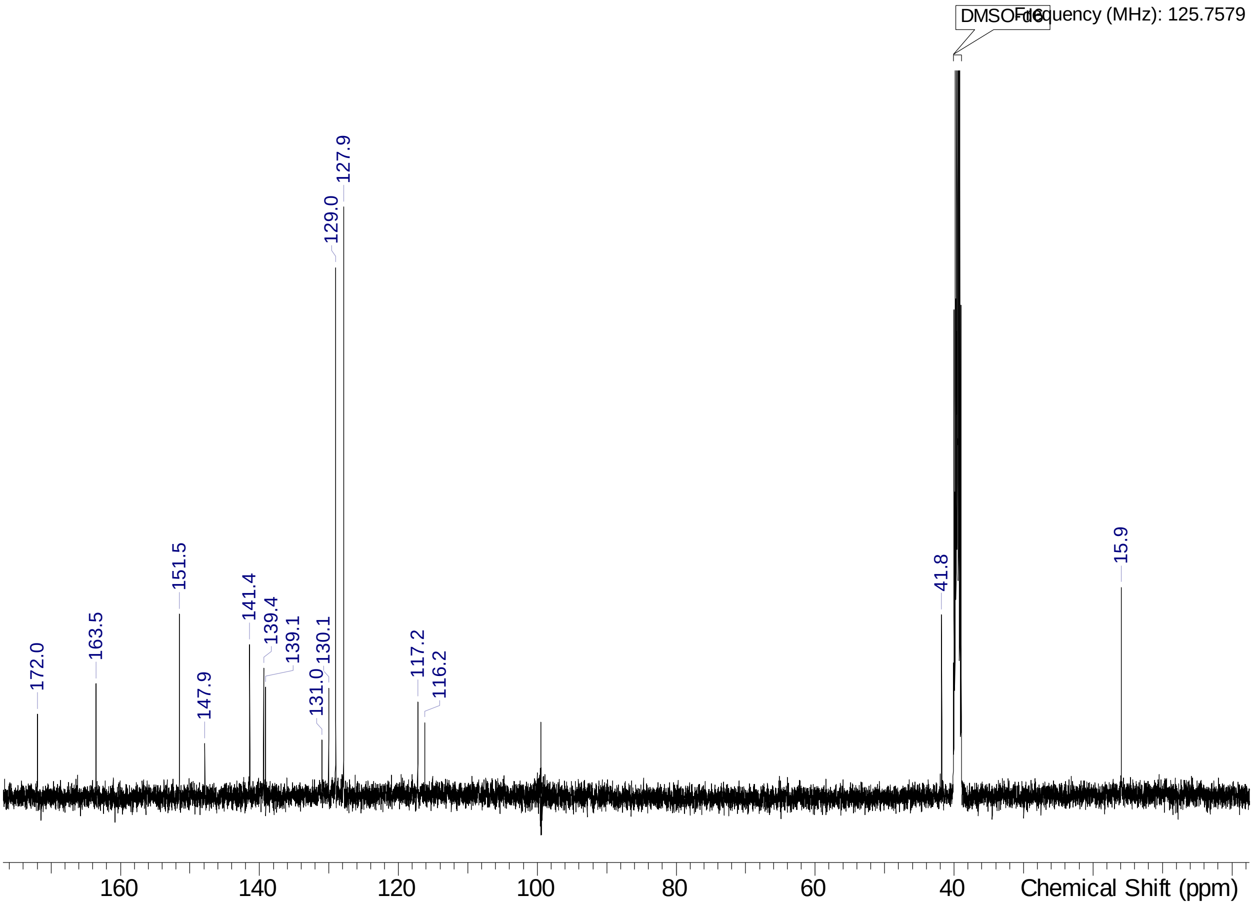
Frequency (MHz): 125.7579
DMSO-d6
127.9
129.0
151.5
141.4
139.4
139.1
130.1
131.0
172.0
163.5
147.9
117.2
116.2
41.8
15.9
160
140
120
100
80
60
40
Chemical Shift (ppm)

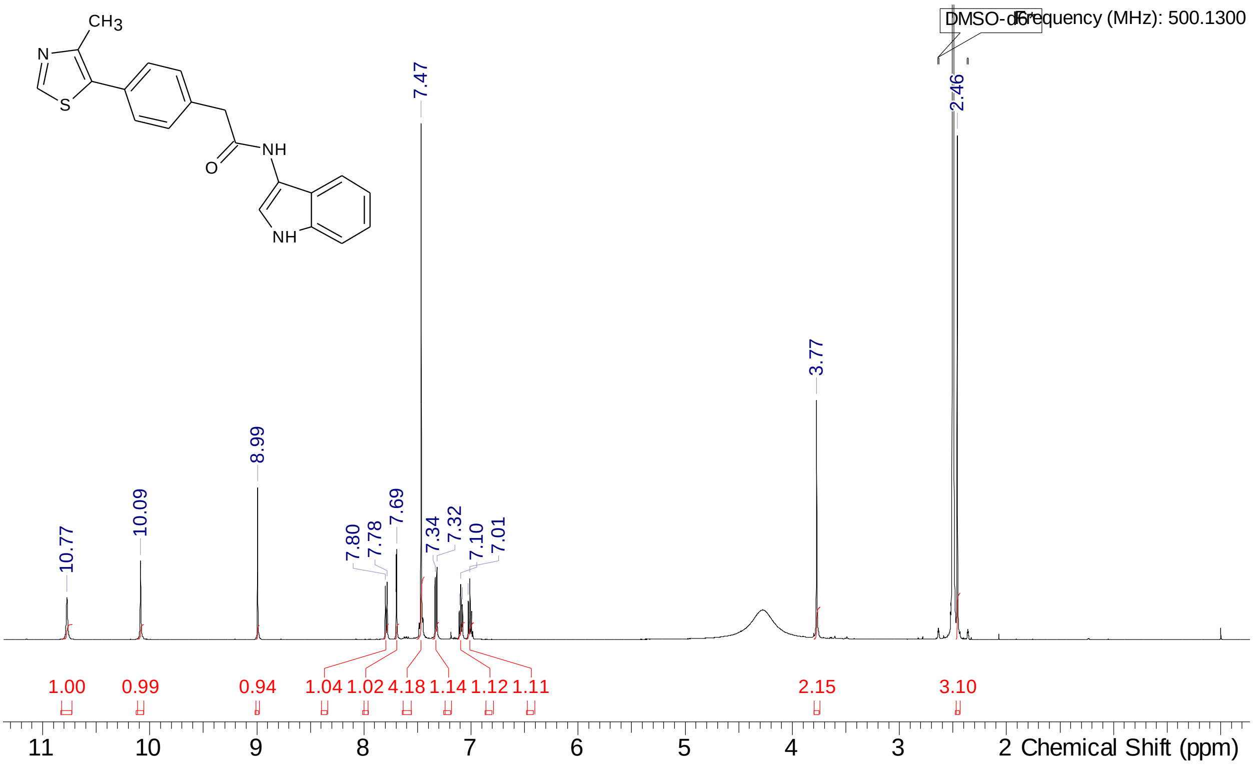
CH3
N
S
NH
O
NH
DMSO-d6
Frequency (MHz): 500.1300
10.77
10.09
8.99
7.80
7.78
7.69
7.47
7.34
7.32
7.10
7.01
3.77
2.46
1.00
0.99
0.94
1.04
1.02
4.18
1.14
1.12
1.11
2.15
3.10
11
10
9
8
7
6
5
4
3
2 Chemical Shift (ppm)

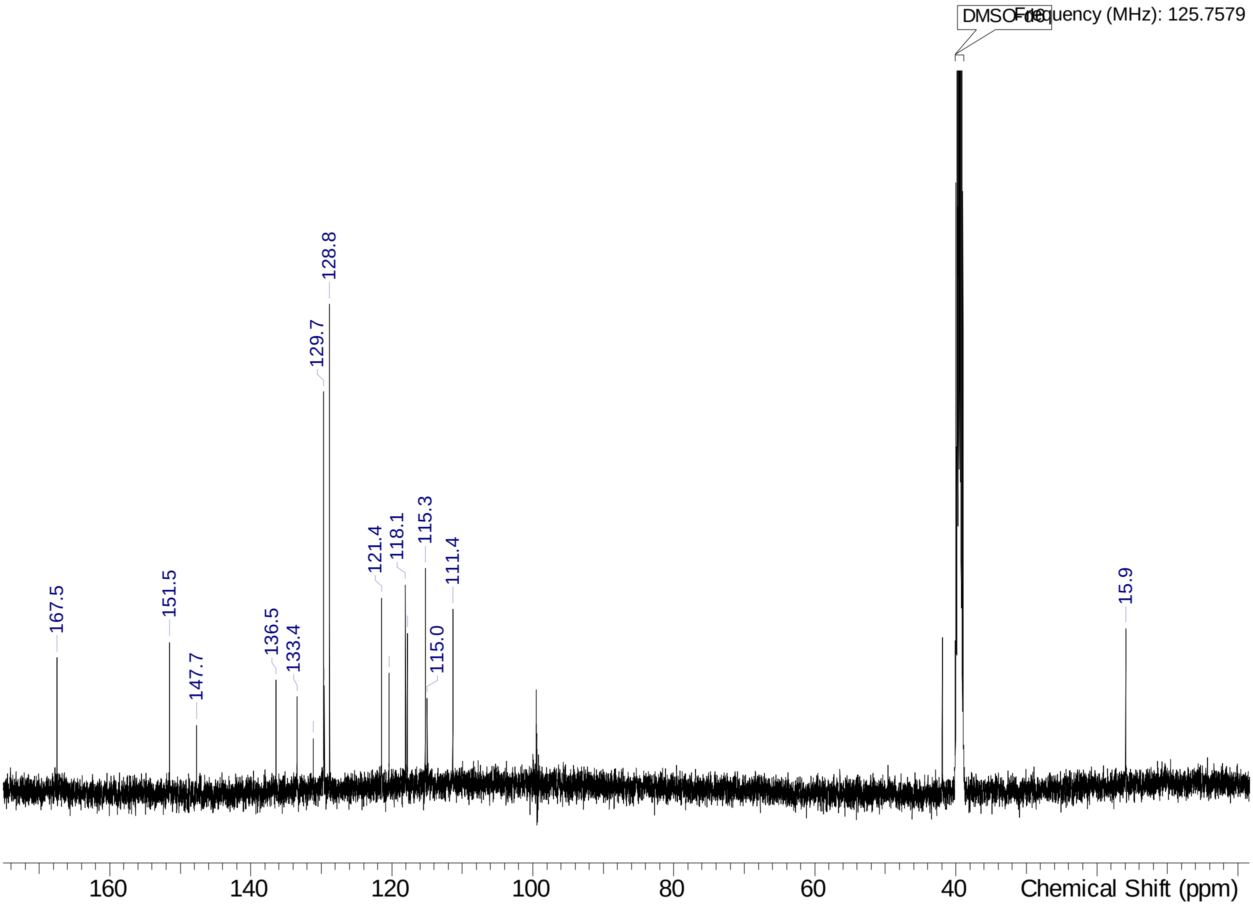
DMSO-d6
Frequency (MHz): 125.7579
167.5
151.5
147.7
136.5
133.4
129.7
128.8
121.4
118.1
115.3
115.0
111.4
15.9
160
140
120
100
80
60
40
Chemical Shift (ppm)

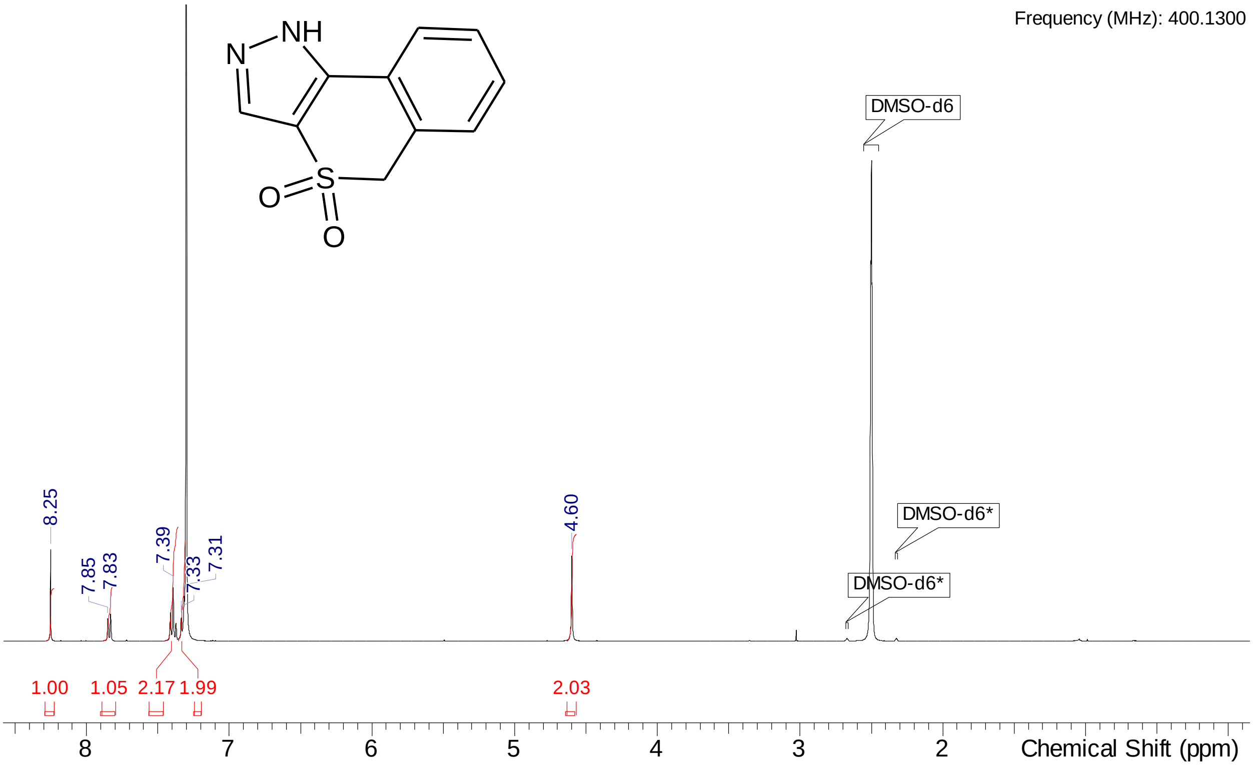
Frequency (MHz): 400.1300
N
NH
O
S
O
DMSO-d6
DMSO-d6*
DMSO-d6*
8.25
7.85
7.83
7.39
7.33
7.31
4.60
1.00
1.05
2.17
1.99
2.03
8
7
6
5
4
3
2
Chemical Shift (ppm)

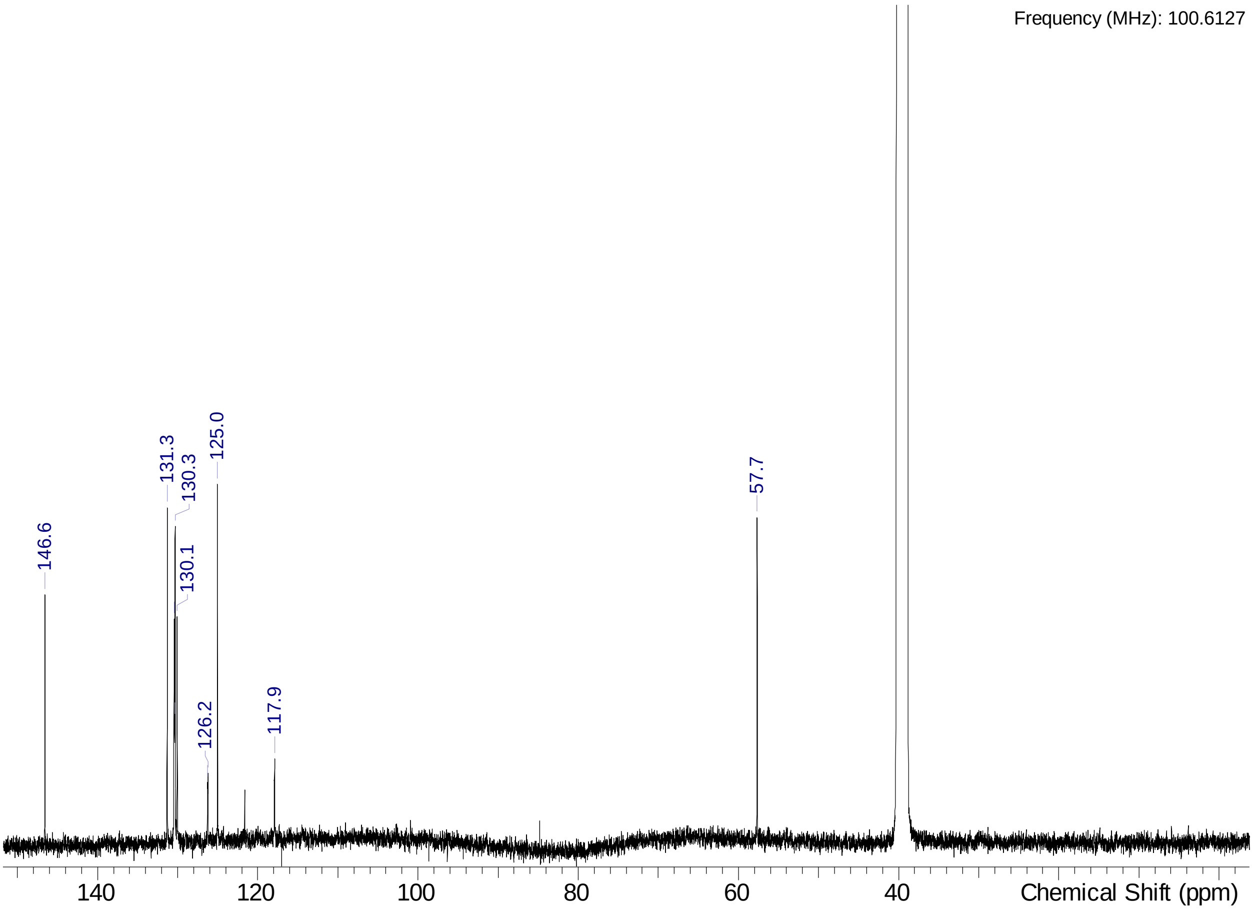
Frequency (MHz): 100.6127
146.6
131.3
130.3
130.1
126.2
125.0
117.9
57.7
140
120
100
80
60
40
Chemical Shift (ppm)

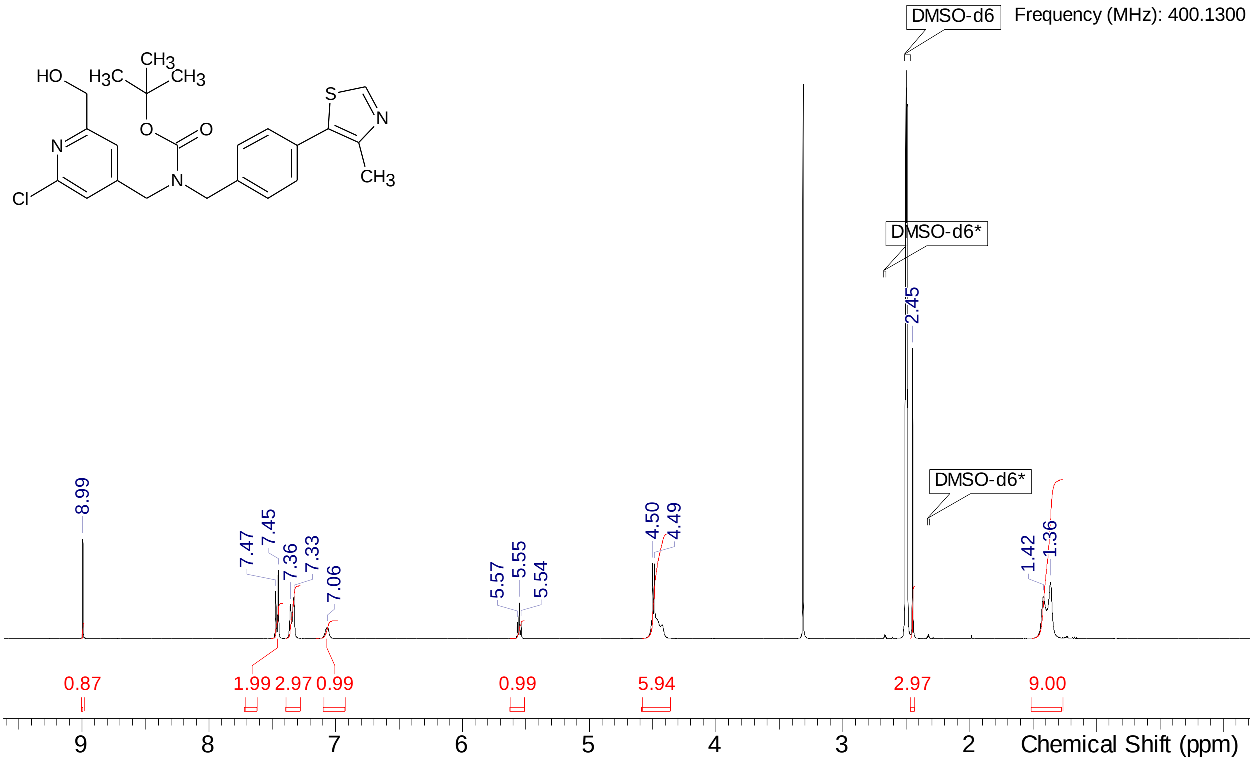
HO
H3C
CH3
CH3
O
O
N
N
Cl
S
N
CH3
DMSO-d6
Frequency (MHz): 400.1300
DMSO-d6*
DMSO-d6*
8.99
7.47
7.45
7.36
7.33
7.06
5.57
5.55
5.54
4.50
4.49
2.45
1.42
1.36
0.87
1.99
2.97
0.99
0.99
5.94
2.97
9.00
9
8
7
6
5
4
3
2
Chemical Shift (ppm)

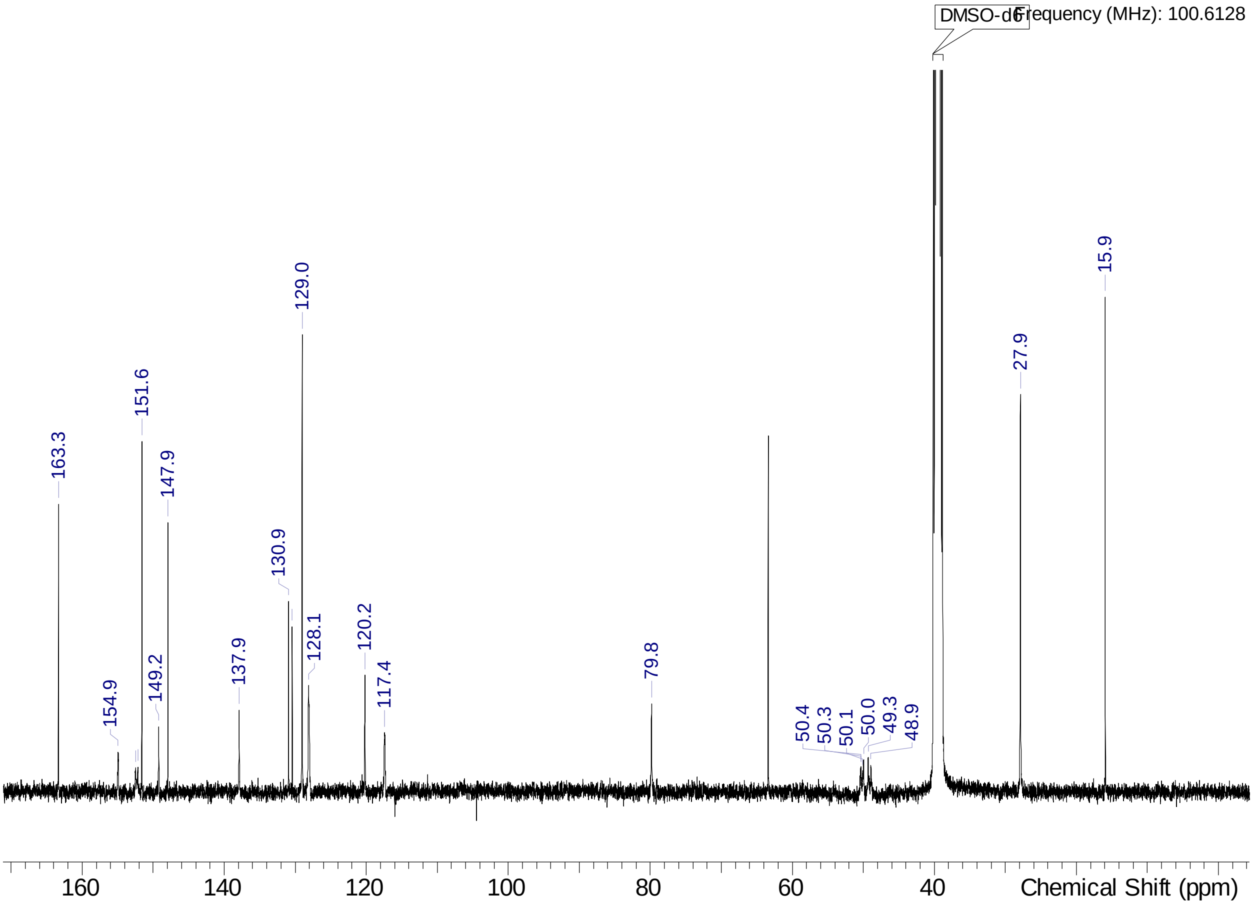
DMSO-d6
Frequency (MHz): 100.6128
163.3
154.9
151.6
149.2
147.9
137.9
130.9
129.0
128.1
120.2
117.4
79.8
50.4
50.3
50.1
50.0
49.3
48.9
27.9
15.9
160
140
120
100
80
60
40
Chemical Shift (ppm)

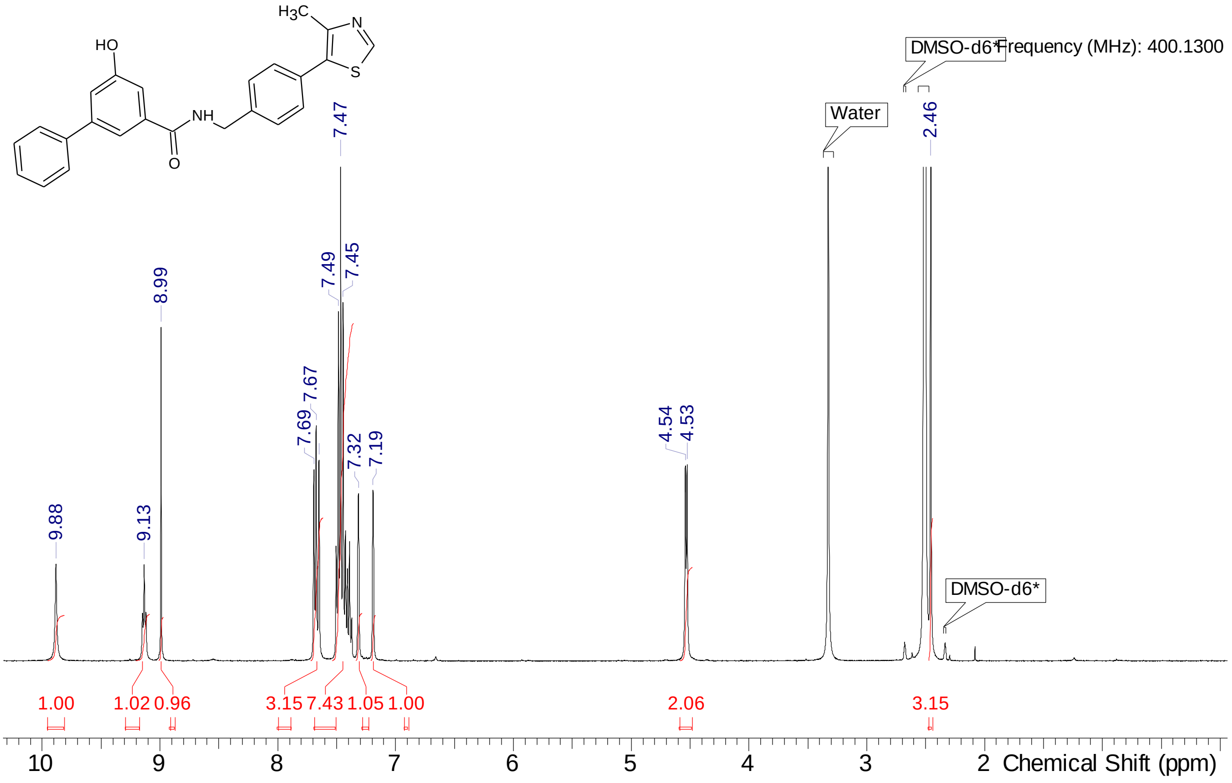
HO
NH
O
H3C
N
S
DMSO-d6*
Frequency (MHz): 400.1300
Water
DMSO-d6*
9.88
9.13
8.99
7.69
7.67
7.49
7.47
7.45
7.32
7.19
4.54
4.53
2.46
1.00
1.02
0.96
3.15
7.43
1.05
1.00
2.06
3.15
10
9
8
7
6
5
4
3
2
Chemical Shift (ppm)

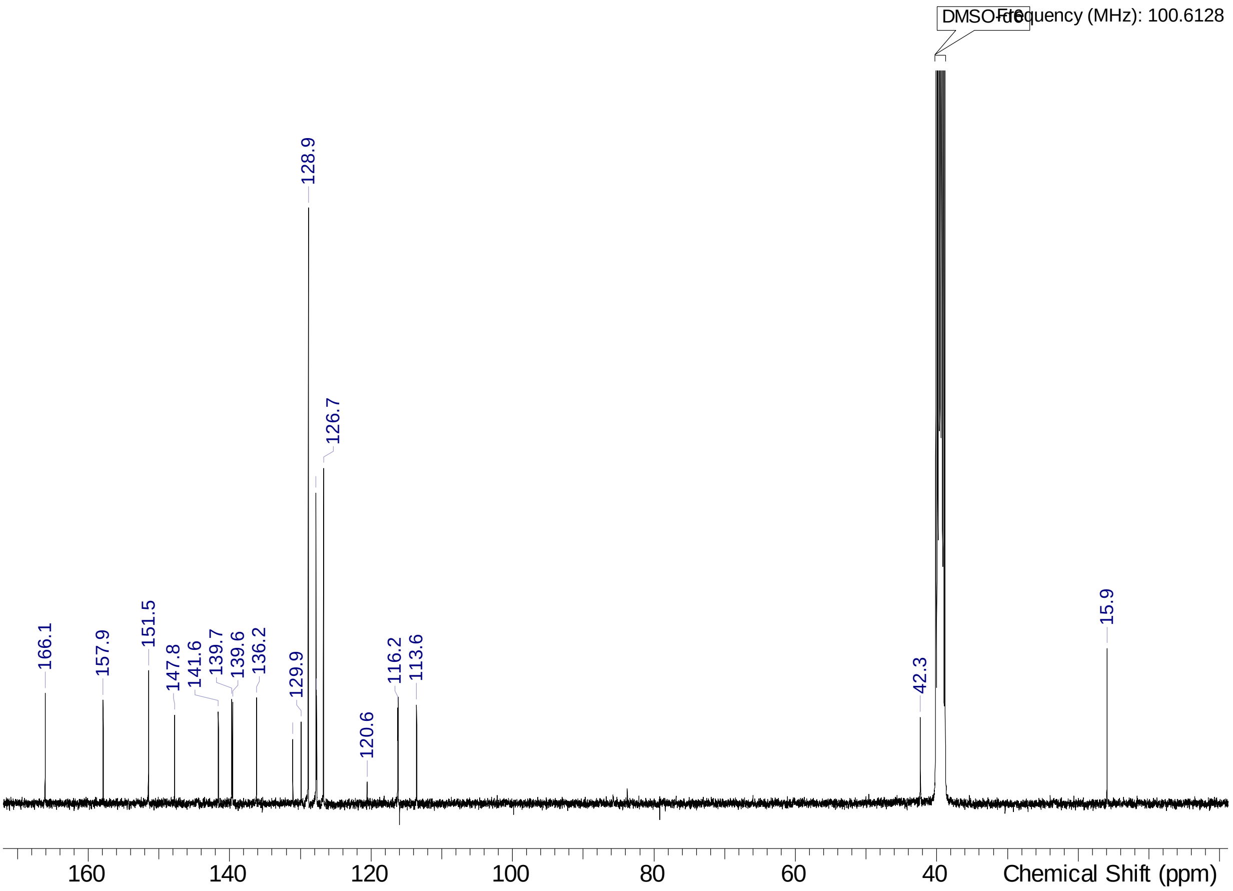
DMSO-d6
Frequency (MHz): 100.6128
166.1
157.9
151.5
147.8
141.6
139.7
139.6
136.2
129.9
128.9
126.7
120.6
116.2
113.6
42.3
15.9
160
140
120
100
80
60
40
Chemical Shift (ppm)

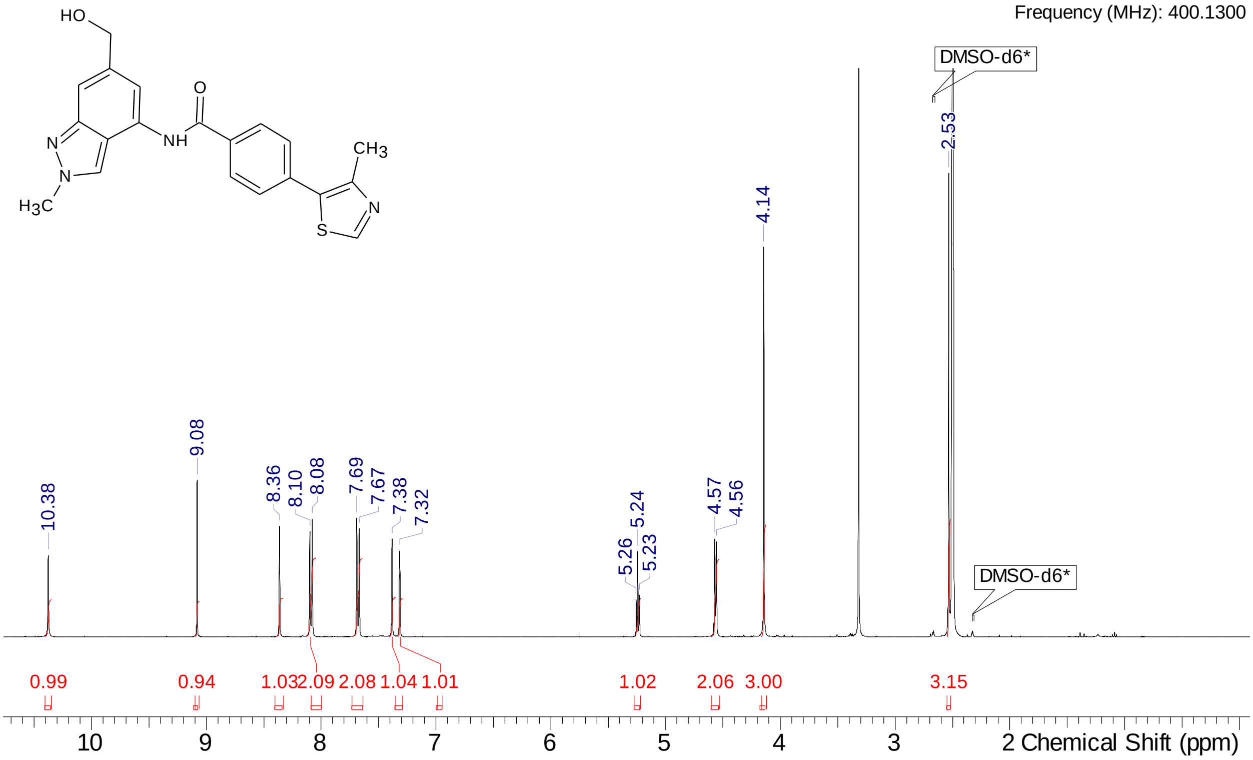
HO
O
NH
N
N
H3C
CH3
N
S
Frequency (MHz): 400.1300
DMSO-d6*
10.38
9.08
8.36
8.10
8.08
7.69
7.67
7.38
7.32
5.26
5.24
5.23
4.57
4.56
4.14
2.53
0.99
0.94
1.03
2.09
2.08
1.04
1.01
1.02
2.06
3.00
3.15
10
9
8
7
6
5
4
3
2 Chemical Shift (ppm)

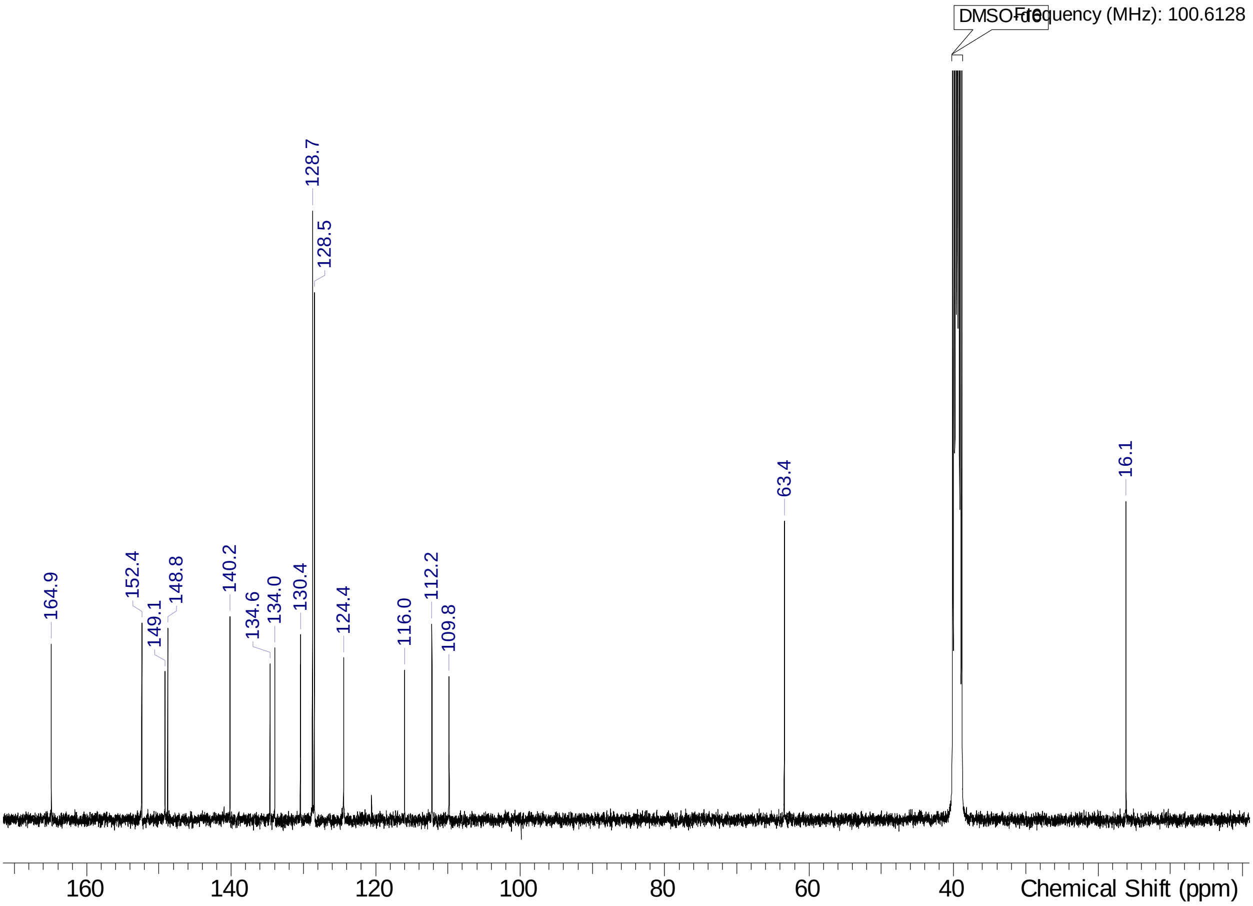
DMSO-d6
Frequency (MHz): 100.6128
164.9
152.4
149.1
148.8
140.2
134.6
134.0
130.4
128.7
128.5
124.4
116.0
112.2
109.8
63.4
16.1
160
140
120
100
80
60
40
Chemical Shift (ppm)

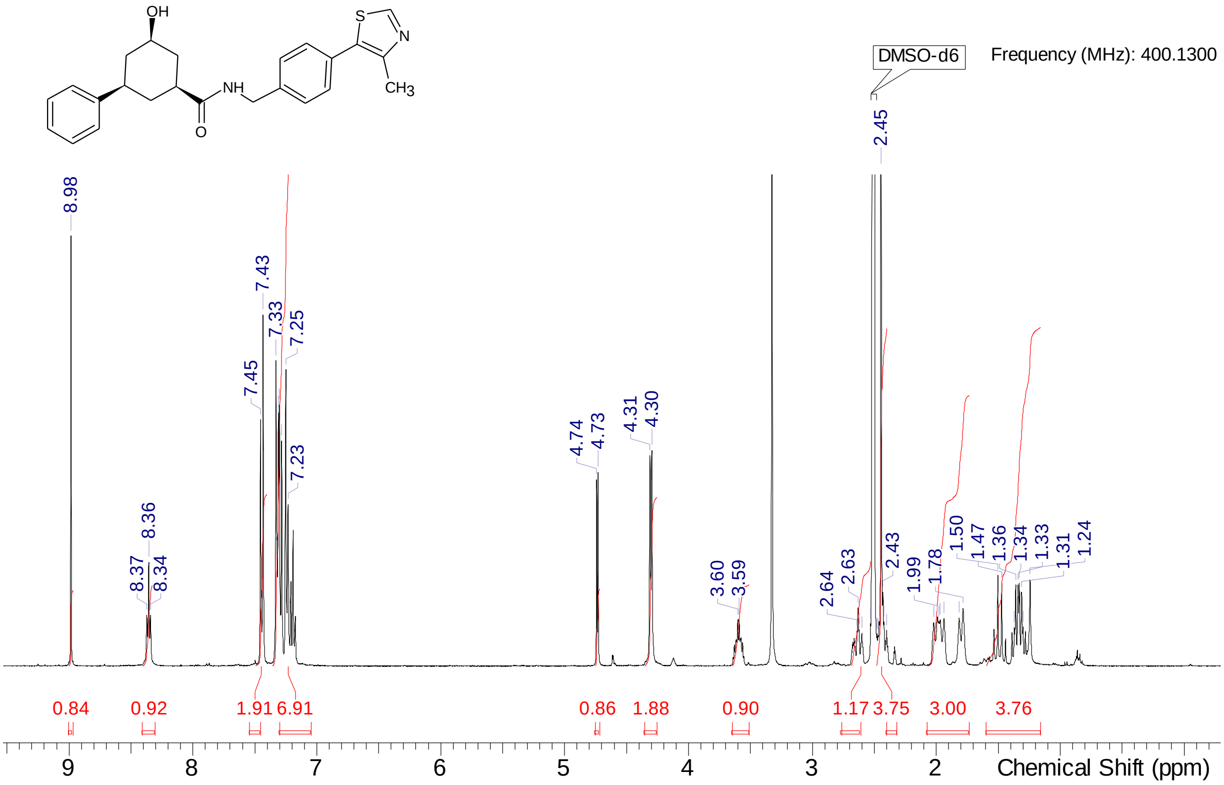
OH
NH
O
S
N
CH3
DMSO-d6
Frequency (MHz): 400.1300
8.98
8.37
8.36
8.34
7.45
7.43
7.33
7.25
7.23
4.74
4.73
4.31
4.30
3.60
3.59
2.64
2.63
2.45
2.43
1.99
1.78
1.50
1.47
1.36
1.34
1.33
1.31
1.24
0.84
0.92
1.91
6.91
0.86
1.88
0.90
1.17
3.75
3.00
3.76
9
8
7
6
5
4
3
2
Chemical Shift (ppm)

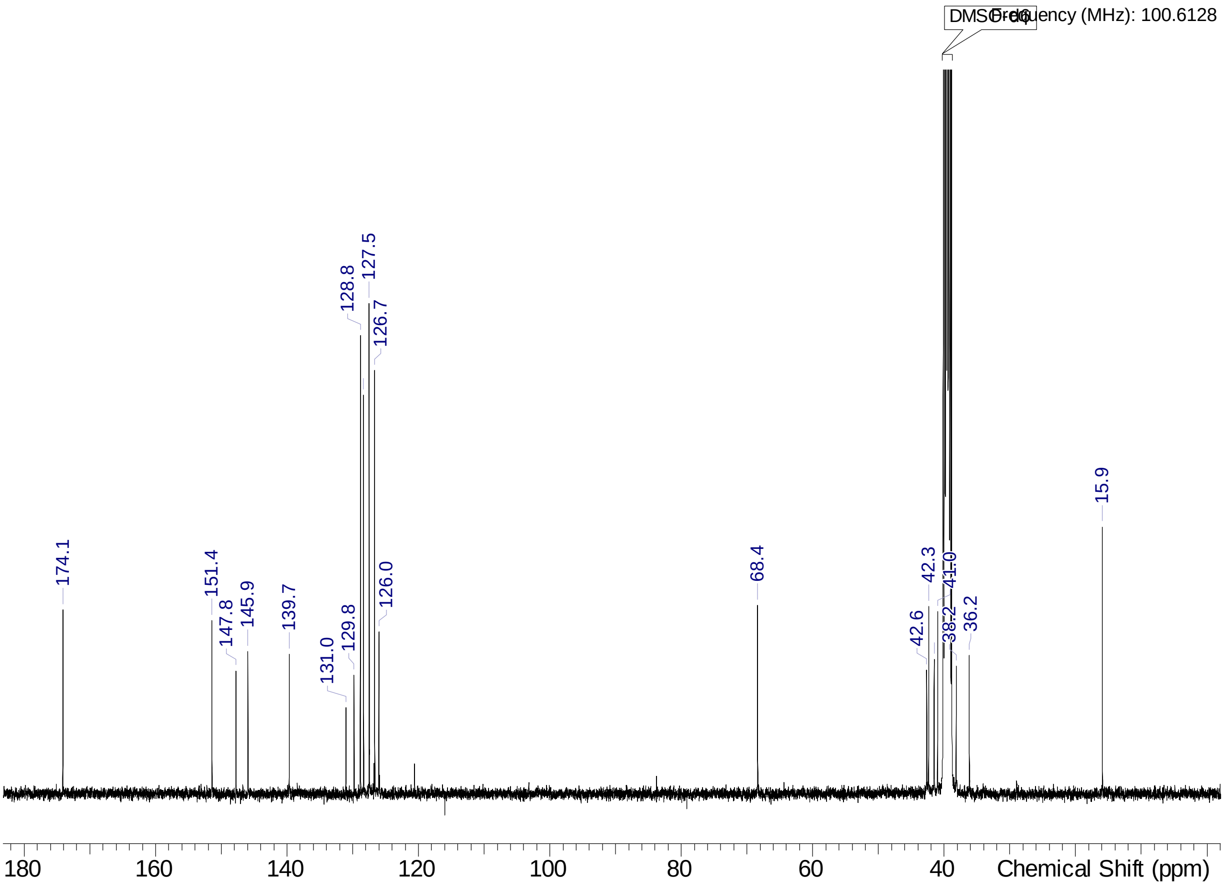
DMSO-d6
Frequency (MHz): 100.6128
174.1
151.4
147.8
145.9
139.7
131.0
129.8
128.8
127.5
126.7
126.0
68.4
42.6
42.3
41.0
38.2
36.2
15.9
180
160
140
120
100
80
60
40
Chemical Shift (ppm)

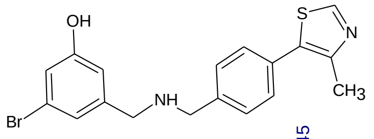
OH
Br
NH
S
N
CH3

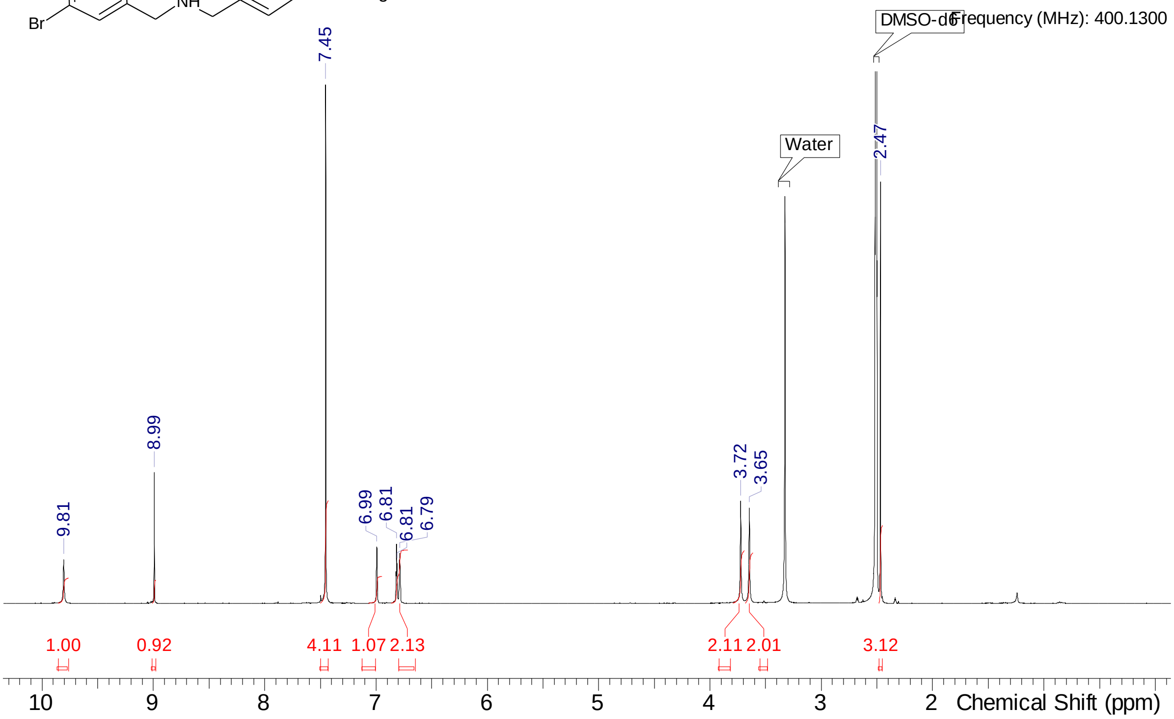
DMSO-d6
Frequency (MHz): 400.1300
Water
9.81
8.99
7.45
6.99
6.81
6.81
6.79
3.72
3.65
2.47
1.00
0.92
4.11
1.07
2.13
2.11
2.01
3.12
10 9 8 7 6 5 4 3 2
Chemical Shift (ppm)

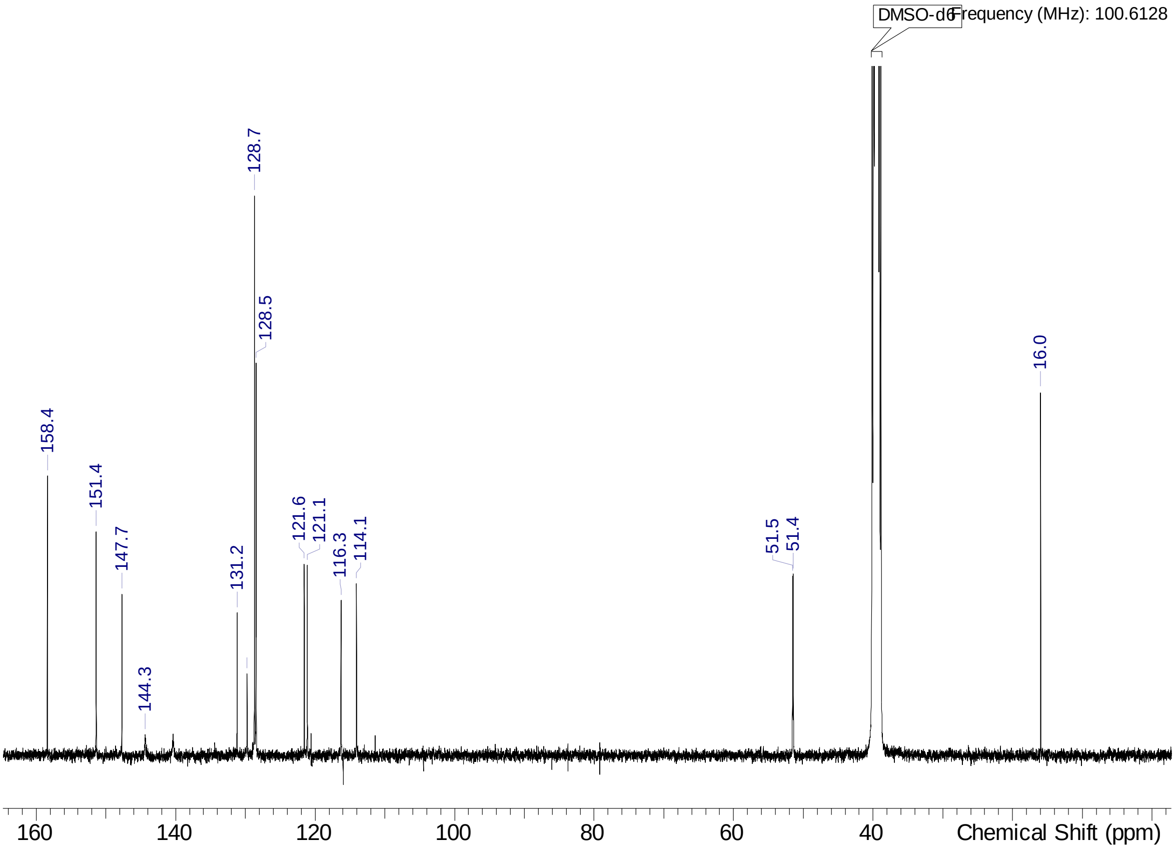
DMSO-d6
Frequency (MHz): 100.6128
158.4
151.4
147.7
144.3
131.2
128.7
128.5
121.6
121.1
116.3
114.1
51.5
51.4
16.0
160 140 120 100 80 60 40
Chemical Shift (ppm)

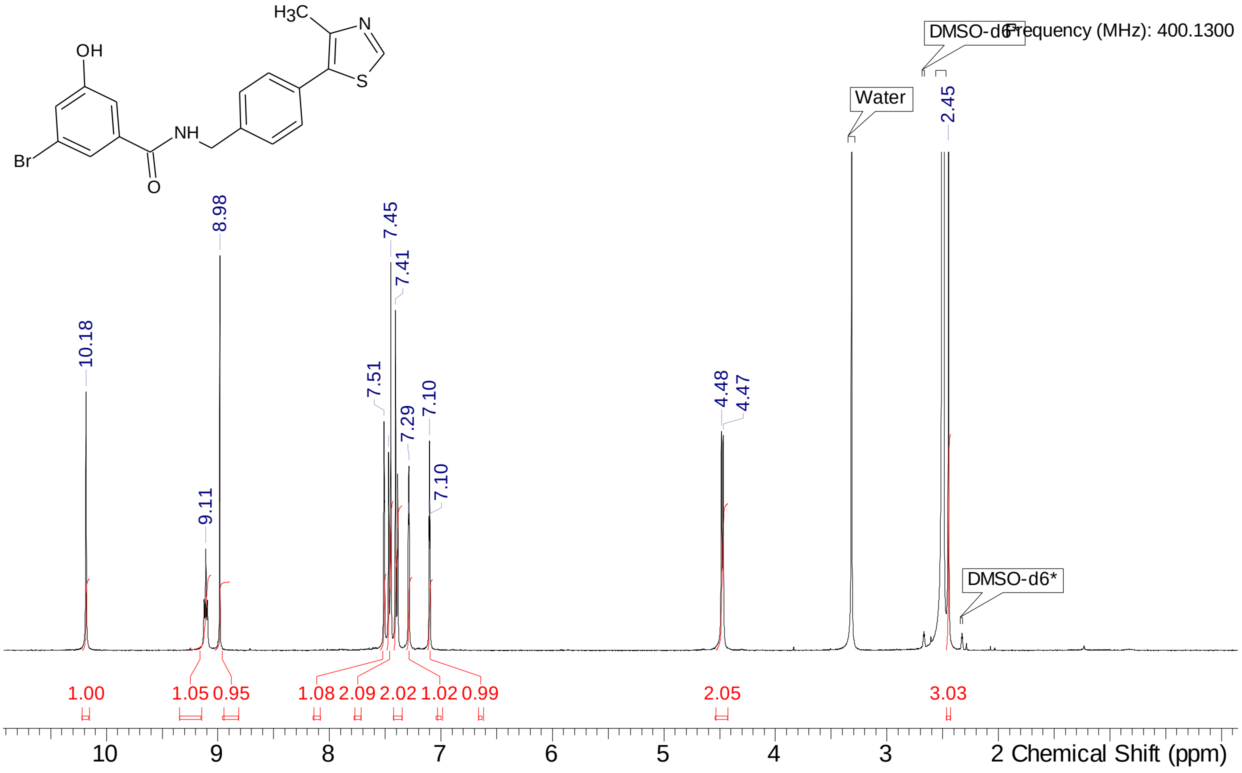
H3C
N
S
OH
NH
Br
O
DMSO-d6
Frequency (MHz): 400.1300
Water
2.45
DMSO-d6*
10.18
9.11
8.98
7.51
7.45
7.41
7.29
7.10
7.10
4.48
4.47
1.00
1.05
0.95
1.08
2.09
2.02
1.02
0.99
2.05
3.03
10
9
8
7
6
5
4
3
2 Chemical Shift (ppm)

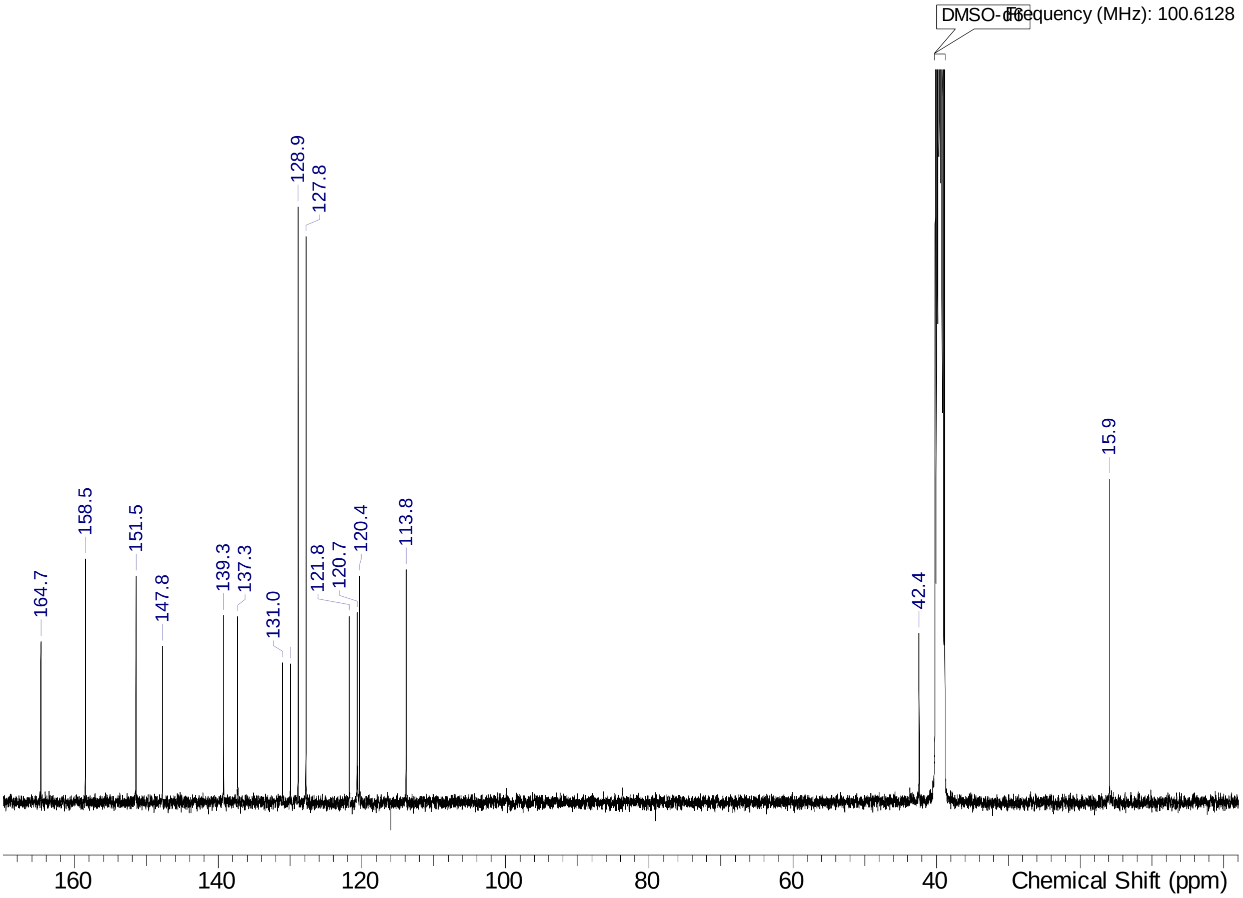
DMSO-d6
Frequency (MHz): 100.6128
164.7
158.5
151.5
147.8
139.3
137.3
131.0
128.9
127.8
121.8
120.7
120.4
113.8
42.4
15.9
160
140
120
100
80
60
40
Chemical Shift (ppm)

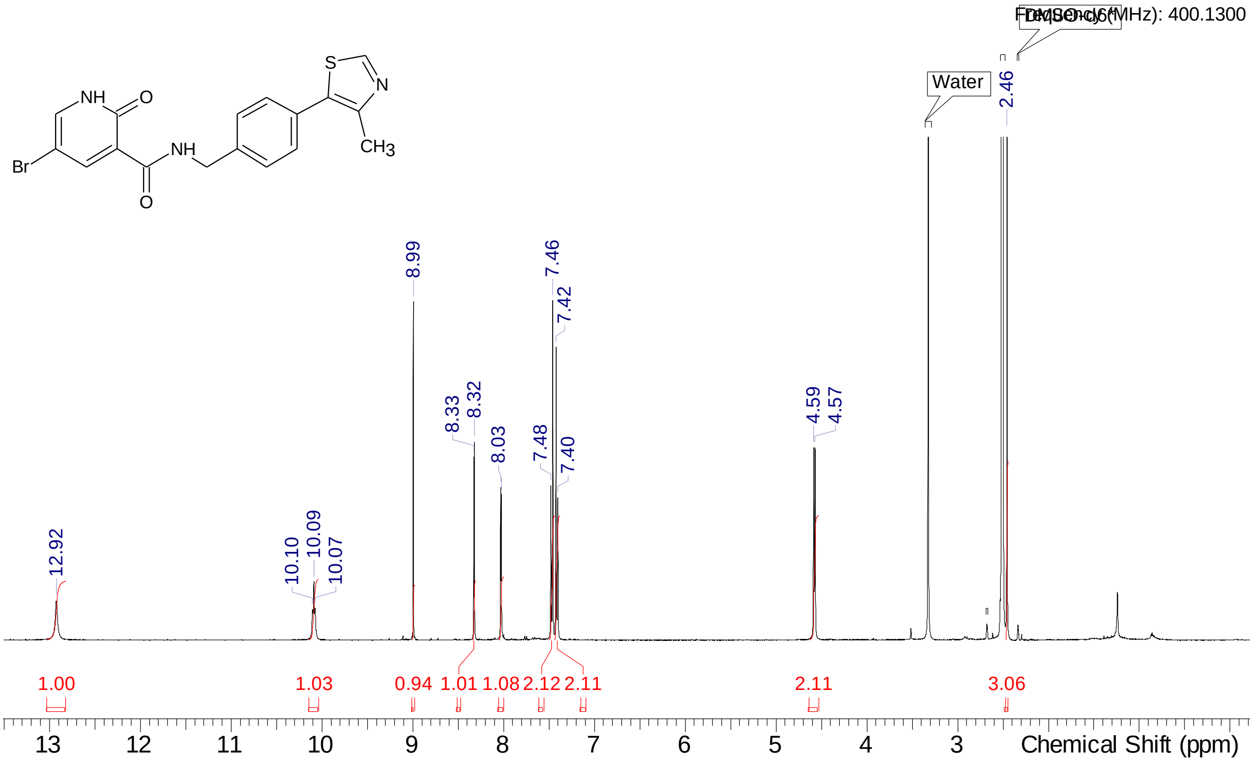
Frequency (MHz): 400.1300
DMSO-d6
Water
Br
NH
O
NH
O
S
N
CH3
12.92
10.10
10.09
10.07
8.99
8.33
8.32
8.03
7.48
7.46
7.42
7.40
4.59
4.57
2.46
1.00
1.03
0.94
1.01
1.08
2.12
2.11
2.11
3.06
13
12
11
10
9
8
7
6
5
4
3
Chemical Shift (ppm)

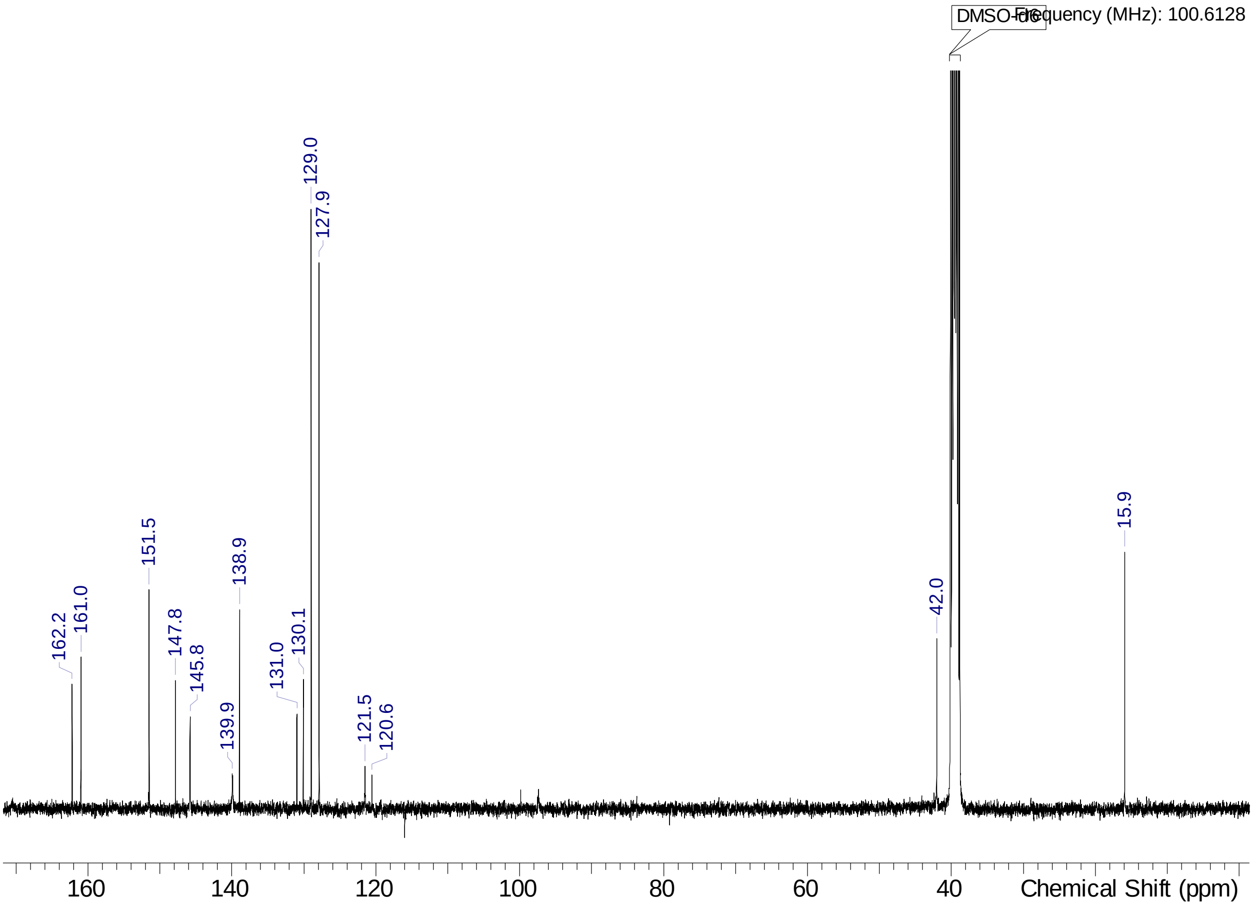
Frequency (MHz): 100.6128
DMSO-d6
162.2
161.0
151.5
147.8
145.8
139.9
138.9
131.0
130.1
129.0
127.9
121.5
120.6
42.0
15.9
160
140
120
100
80
60
40
Chemical Shift (ppm)

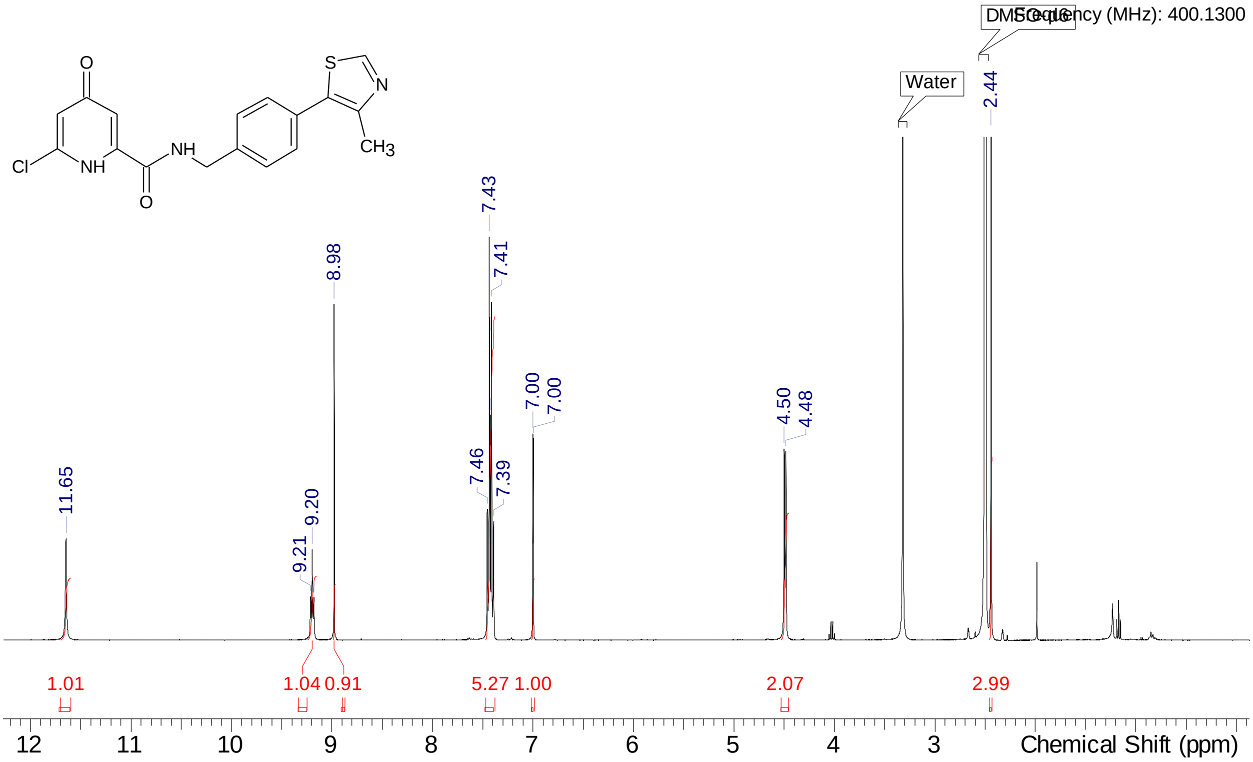
O
S
N
NH
Cl
NH
O
CH3
Frequency (MHz): 400.1300
DMSO-d6
Water
2.44
11.65
9.21
9.20
8.98
7.46
7.43
7.41
7.39
7.00
7.00
4.50
4.48
1.01
1.04
0.91
5.27
1.00
2.07
2.99
12
11
10
9
8
7
6
5
4
3
Chemical Shift (ppm)

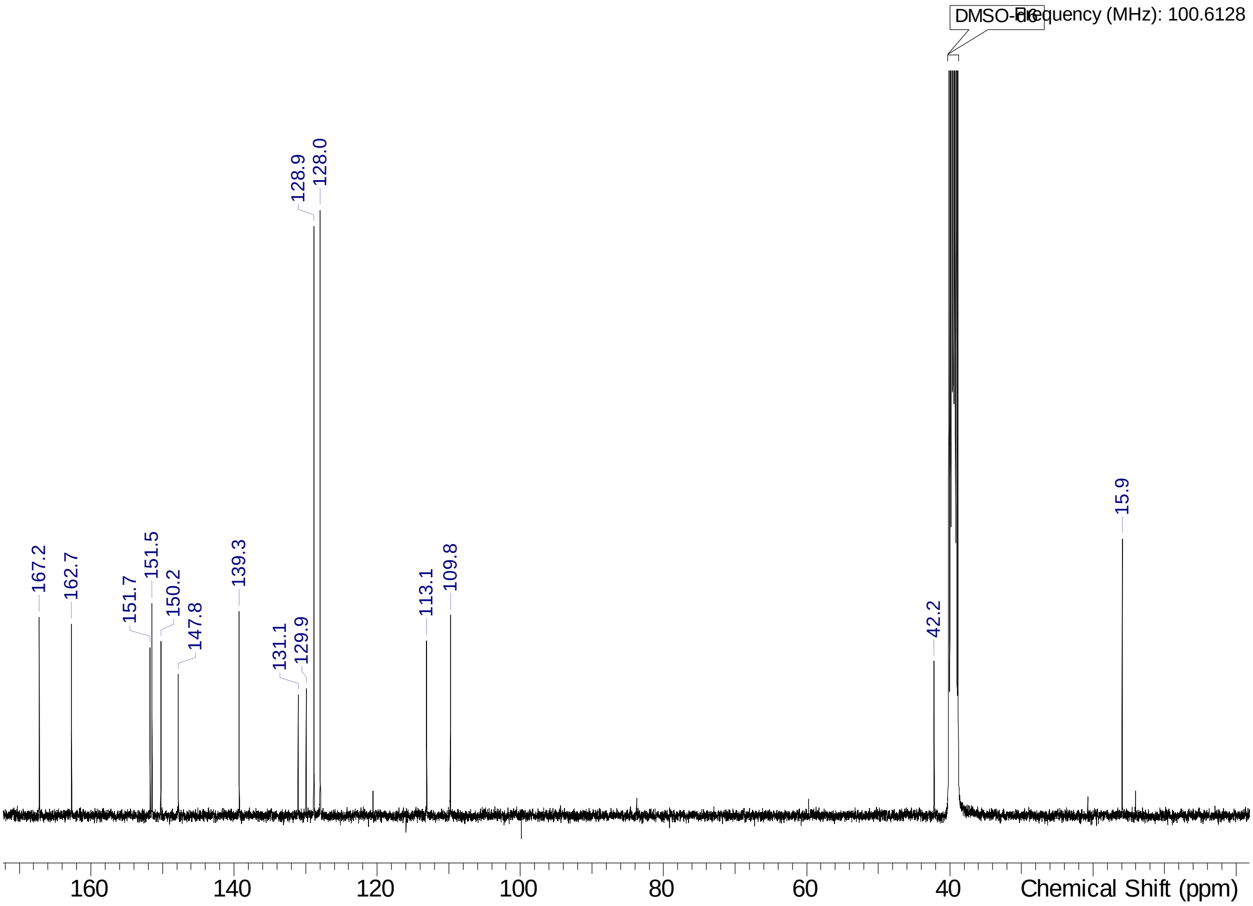
Frequency (MHz): 100.6128
DMSO-d6
167.2
162.7
151.7
151.5
150.2
147.8
139.3
131.1
129.9
128.9
128.0
113.1
109.8
42.2
15.9
160
140
120
100
80
60
40
Chemical Shift (ppm)

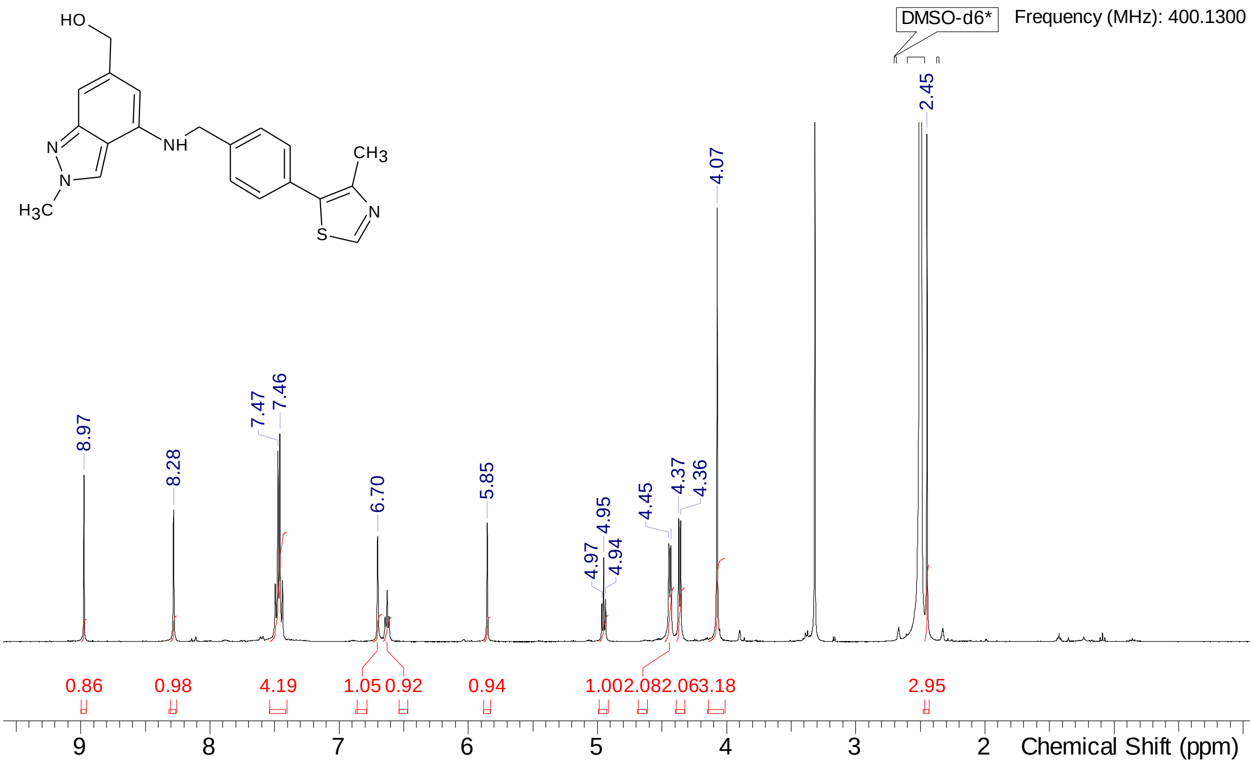
HO
N
N
H3C
NH
CH3
N
S
DMSO-d6*
Frequency (MHz): 400.1300
8.97
8.28
7.47
7.46
6.70
5.85
4.97
4.95
4.94
4.45
4.37
4.36
4.07
2.45
0.86
0.98
4.19
1.05
0.92
0.94
1.00
2.08
2.06
3.18
2.95
9
8
7
6
5
4
3
2
Chemical Shift (ppm)

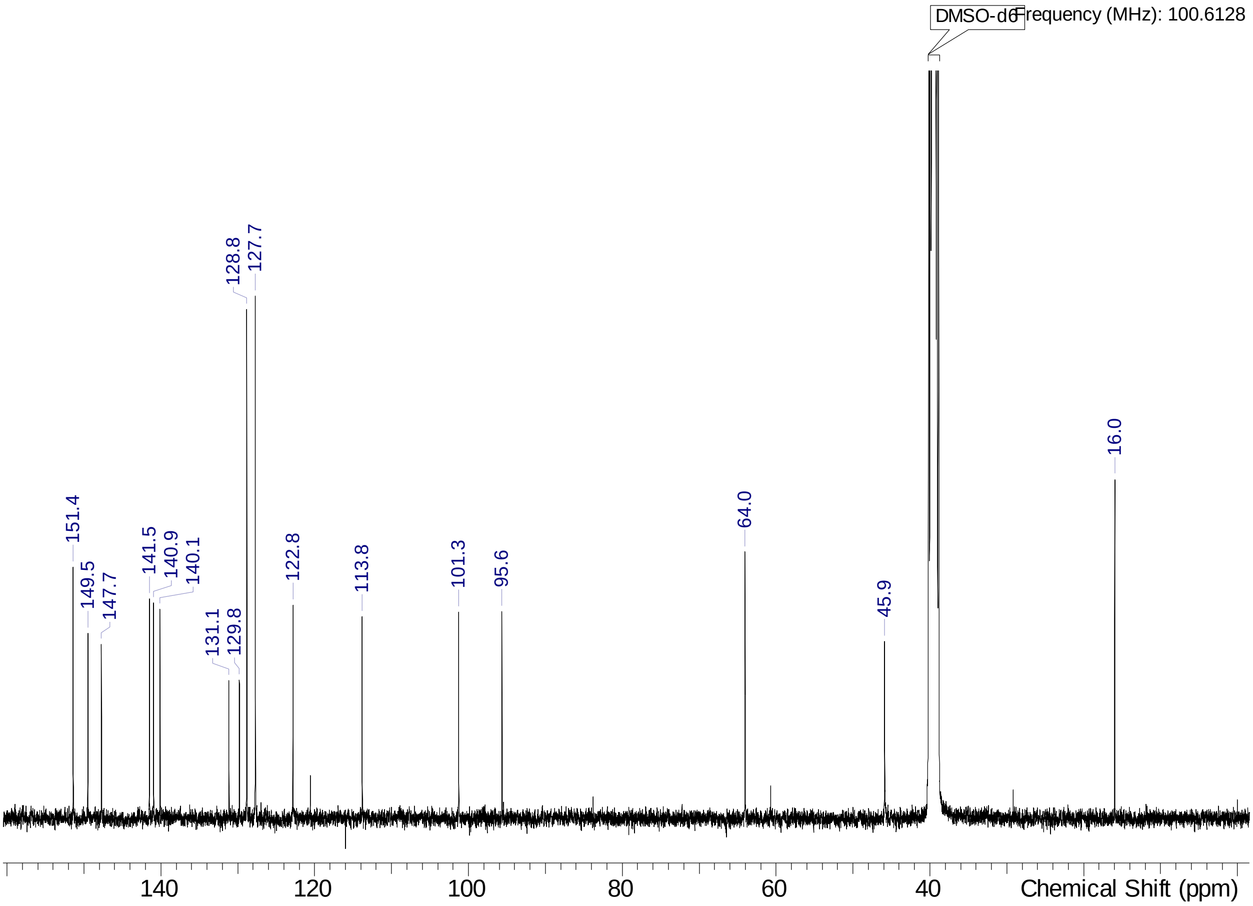
DMSO-d6
Frequency (MHz): 100.6128
151.4
149.5
147.7
141.5
140.9
140.1
131.1
129.8
128.8
127.7
122.8
113.8
101.3
95.6
64.0
45.9
16.0
140
120
100
80
60
40
Chemical Shift (ppm)

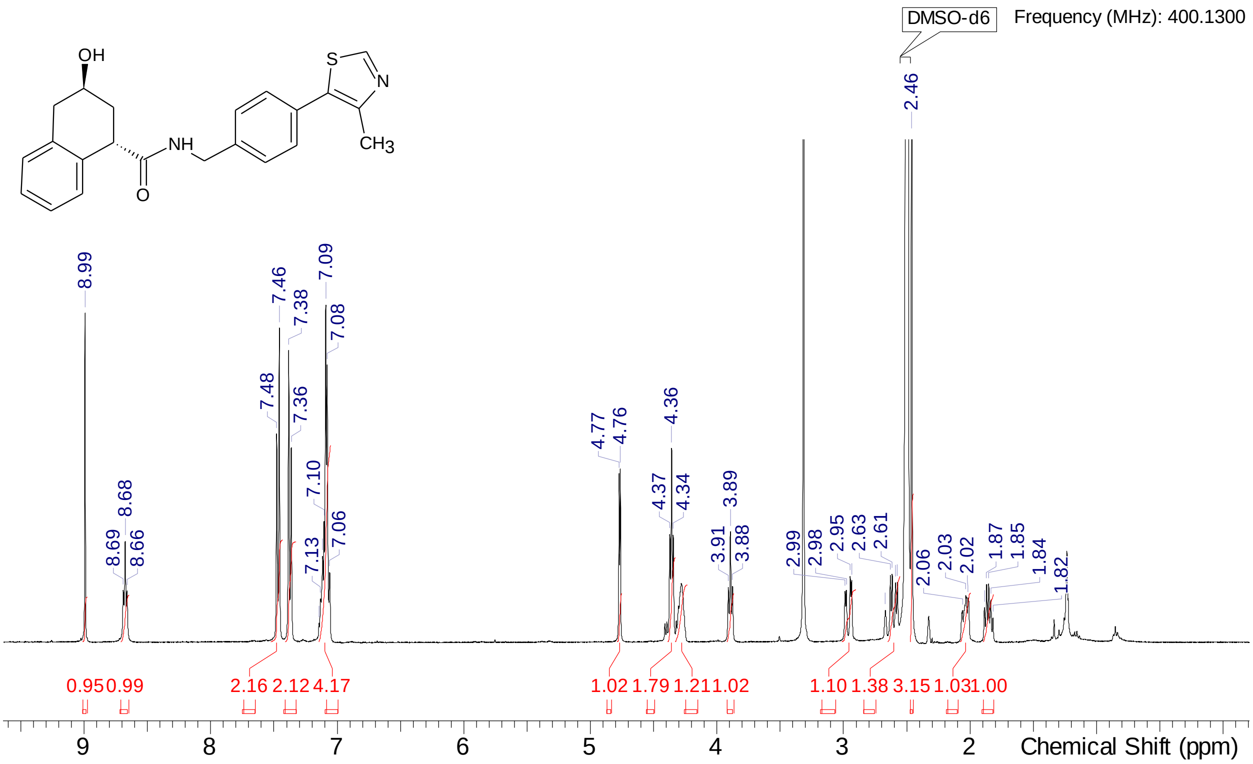
OH
NH
O
S
N
CH3
DMSO-d6
Frequency (MHz): 400.1300
2.46
8.99
8.69 8.68 8.66
7.48 7.46 7.38 7.36 7.13 7.10 7.09 7.08 7.06
4.77 4.76
4.37 4.36 4.34
3.91 3.89 3.88
2.99 2.98 2.95 2.63 2.61
2.06 2.03 2.02 1.87 1.85 1.84 1.82
0.95 0.99 2.16 2.12 4.17 1.02 1.79 1.21 1.02 1.10 1.38 3.15 1.03 1.00
9 8 7 6 5 4 3 2
Chemical Shift (ppm)

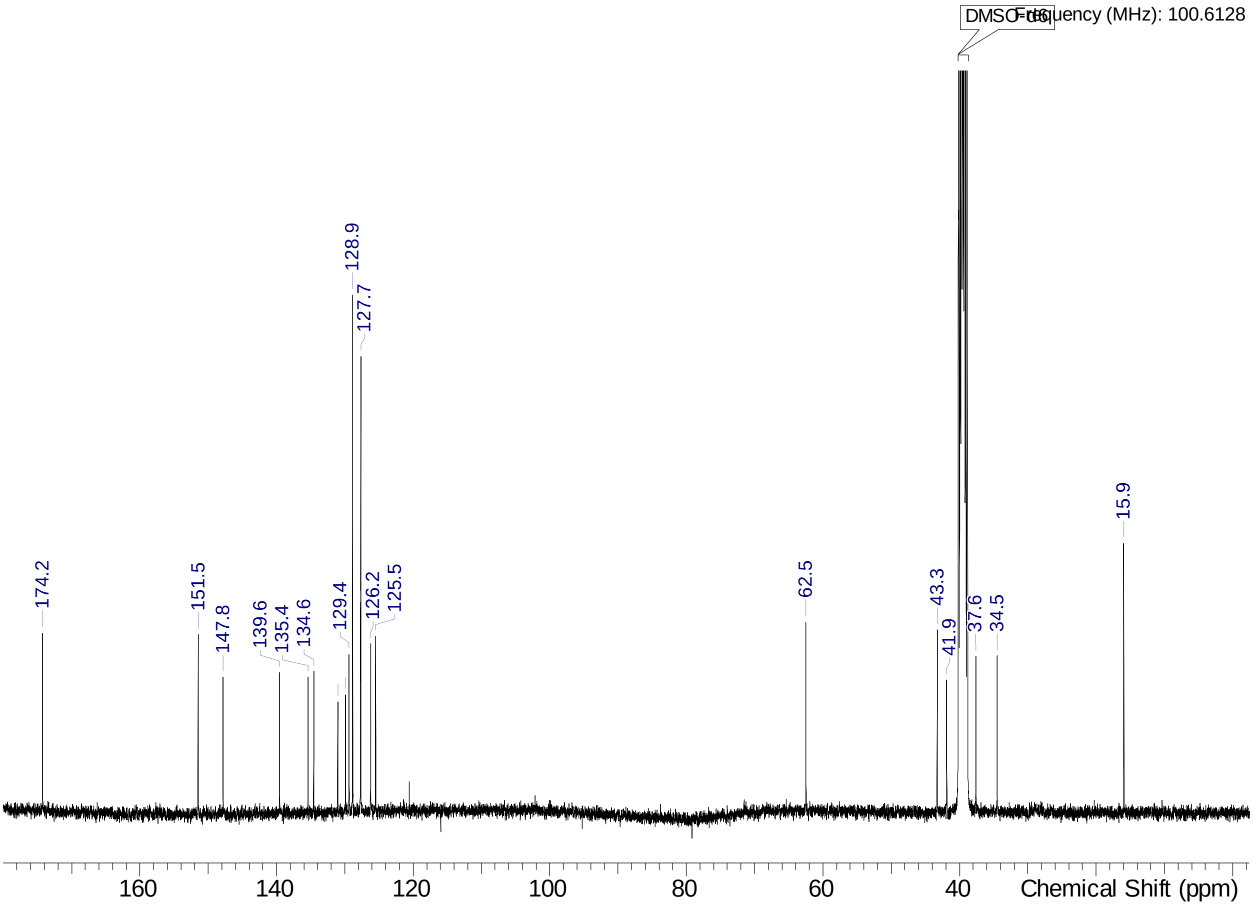
DMSO-d6
Frequency (MHz): 100.6128
174.2
151.5 147.8
139.6 135.4 134.6
129.4 128.9 127.7 126.2 125.5
62.5
43.3 41.9 37.6 34.5
15.9
160 140 120 100 80 60 40
Chemical Shift (ppm)

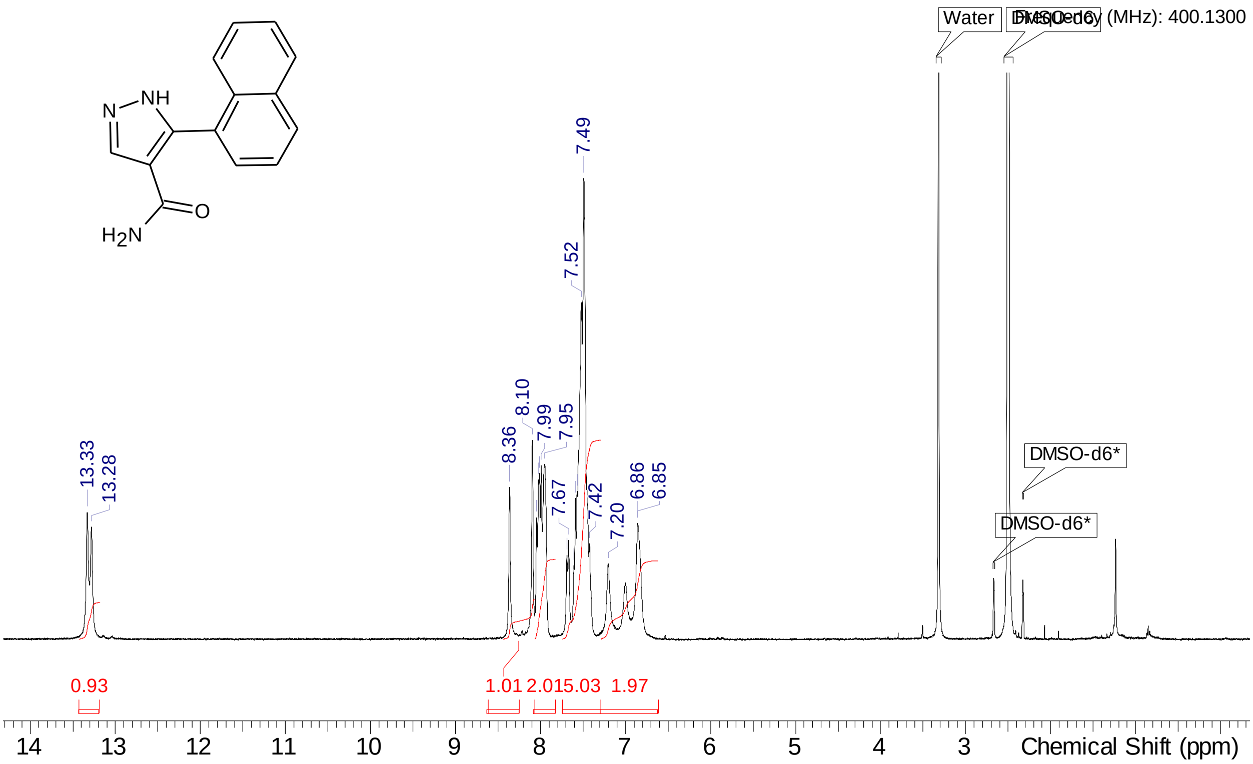
N
NH
H2N
O
Water
DMSO-d6
Frequency (MHz): 400.1300
13.33
13.28
8.36
8.10
7.99
7.95
7.67
7.52
7.49
7.42
7.20
6.86
6.85
DMSO-d6*
DMSO-d6*
0.93
1.01
2.01
5.03
1.97
14
13
12
11
10
9
8
7
6
5
4
3
Chemical Shift (ppm)

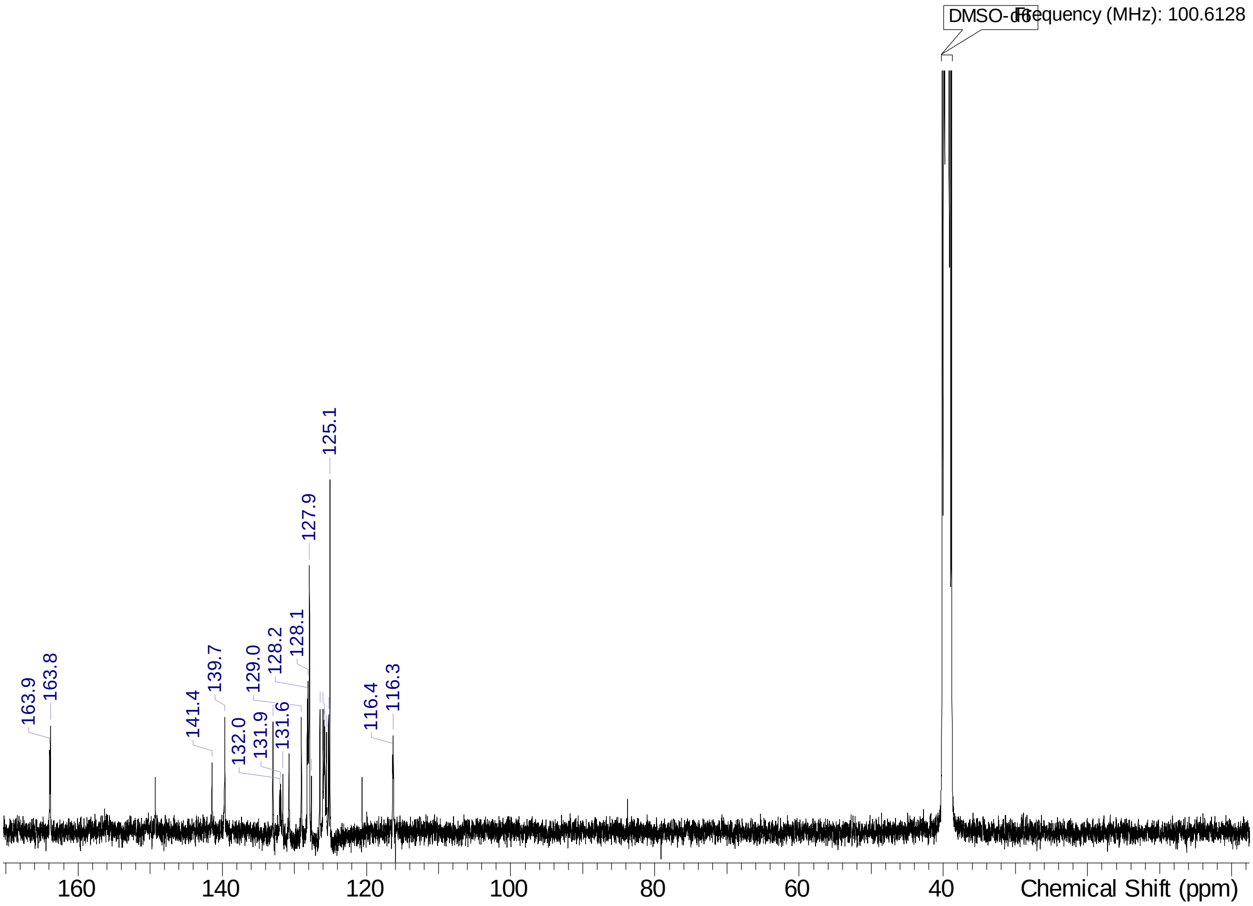
DMSO-d6
Frequency (MHz): 100.6128
163.9
163.8
141.4
139.7
132.0
131.9
131.6
129.0
128.2
128.1
127.9
125.1
116.4
116.3
160
140
120
100
80
60
40
Chemical Shift (ppm)

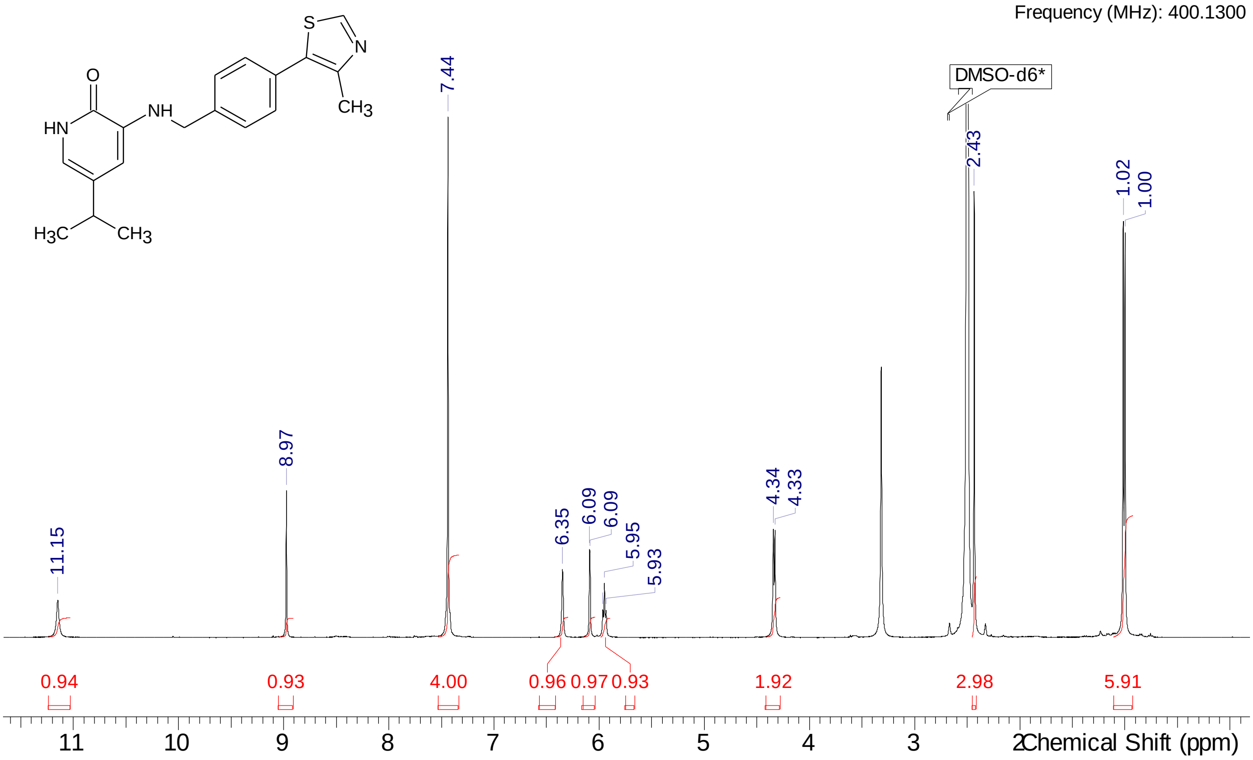
Frequency (MHz): 400.1300
DMSO-d6*
11.15
8.97
7.44
6.35
6.09
6.09
5.95
5.93
4.34
4.33
2.43
1.02
1.00
0.94
0.93
4.00
0.96
0.97
0.93
1.92
2.98
5.91
Chemical Shift (ppm)

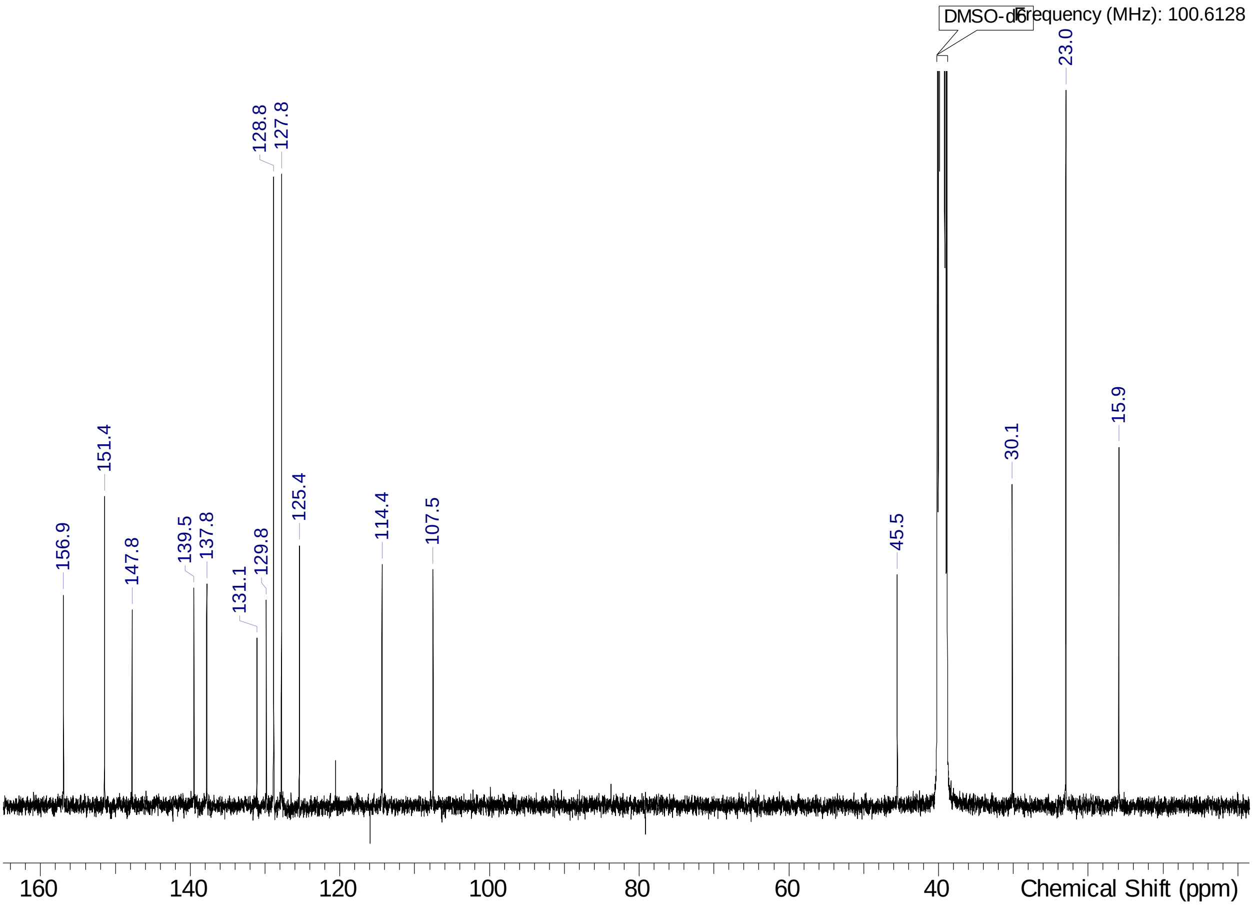
Frequency (MHz): 100.6128
DMSO-d6
156.9
151.4
147.8
139.5
137.8
131.1
129.8
128.8
127.8
125.4
114.4
107.5
45.5
30.1
23.0
15.9
Chemical Shift (ppm)

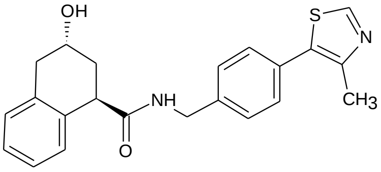
OH
S
N
NH
O
CH3

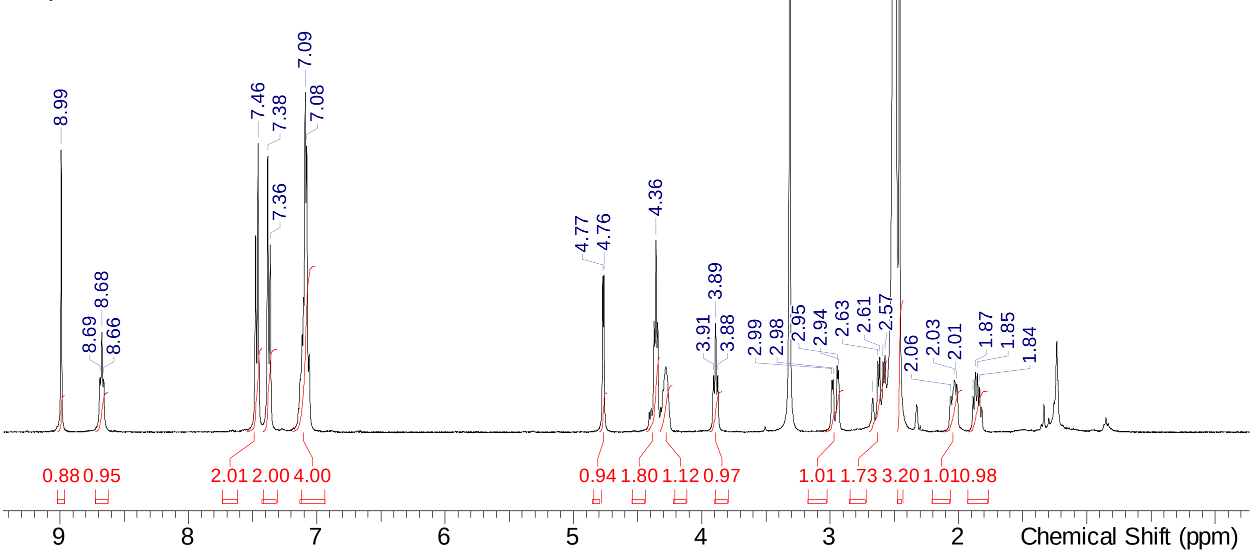
DMSO-d6
Frequency (MHz): 400.1300
8.99
8.69
8.68
8.66
7.46
7.38
7.36
7.09
7.08
4.77
4.76
4.36
3.91
3.89
3.88
2.99
2.98
2.95
2.94
2.63
2.61
2.57
2.46
2.06
2.03
2.01
1.87
1.85
1.84
0.88 0.95 2.01 2.00 4.00 0.94 1.80 1.12 0.97 1.01 1.73 3.20 1.01 0.98
9 8 7 6 5 4 3 2
Chemical Shift (ppm)

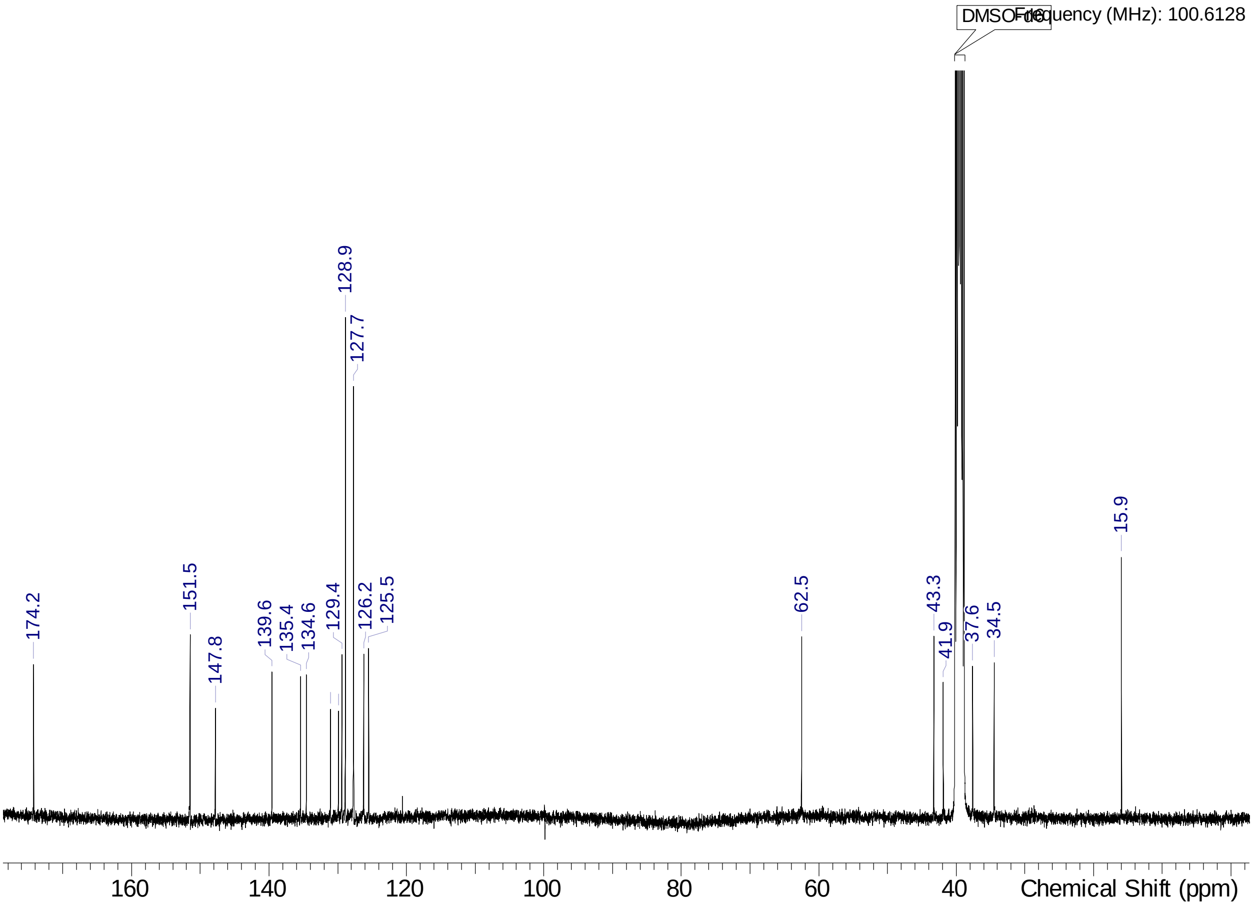
DMSO-d6
Frequency (MHz): 100.6128
174.2
151.5
147.8
139.6
135.4
134.6
129.4
128.9
127.7
126.2
125.5
62.5
43.3
41.9
37.6
34.5
15.9
160 140 120 100 80 60 40
Chemical Shift (ppm)

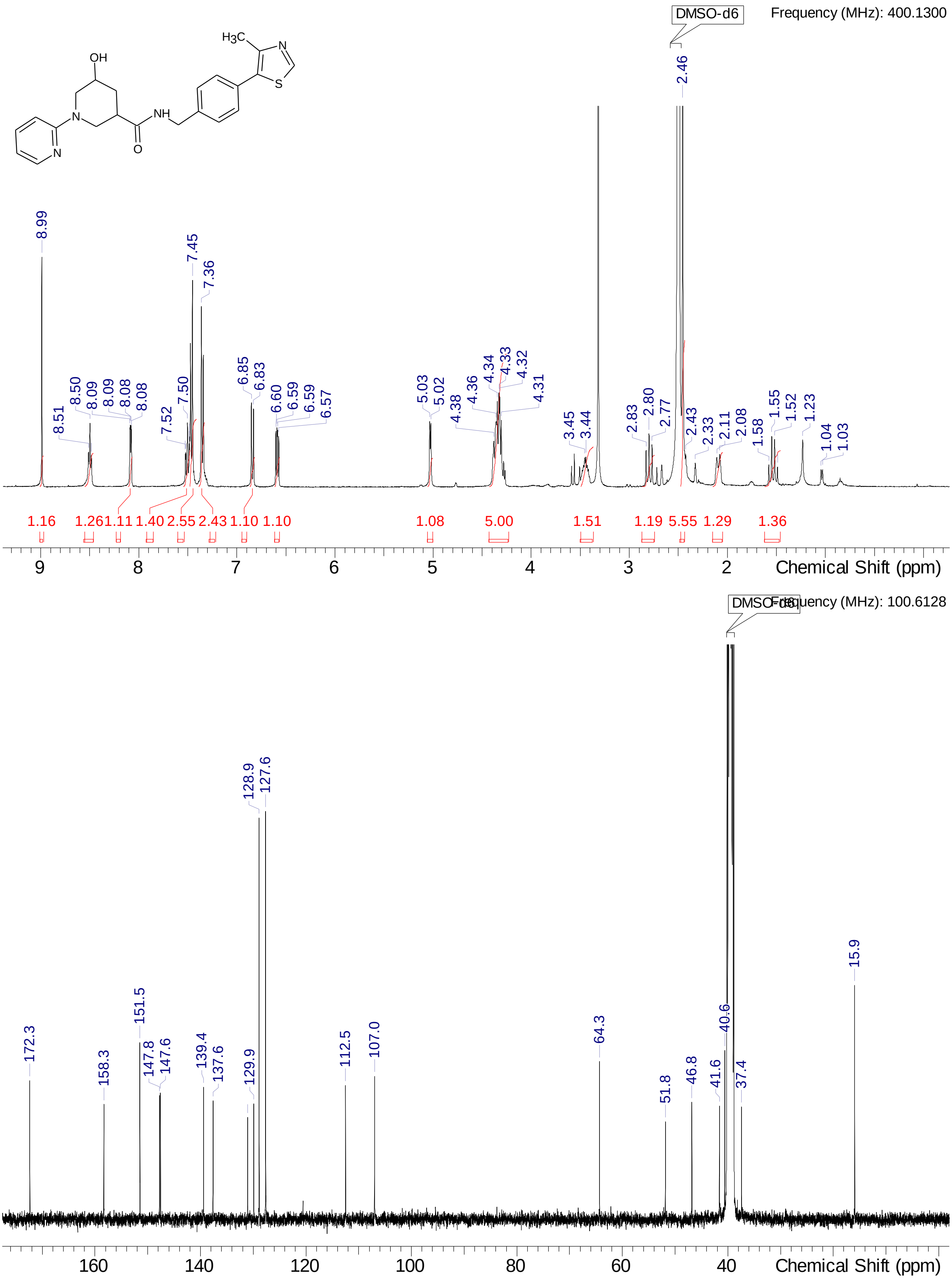
OH
N
NH
O
N
H3C
N
S
DMSO-d6
Frequency (MHz): 400.1300
8.99
8.51 8.50 8.09 8.09 8.08 8.08
7.52 7.50 7.45 7.36
6.85 6.83 6.60 6.59 6.59 6.57
5.03 5.02
4.38 4.36 4.34 4.33 4.32 4.31
3.45 3.44
2.83 2.80 2.77
2.46
2.43 2.33 2.11 2.08
1.58 1.55 1.52
1.23 1.04 1.03
1.16 1.26 1.11 1.40 2.55 2.43 1.10 1.10 1.08 5.00 1.51 1.19 5.55 1.29 1.36
9 8 7 6 5 4 3 2
Chemical Shift (ppm)
DMSO-d6
Frequency (MHz): 100.6128
172.3
158.3
151.5
147.8 147.6
139.4 137.6
129.9
128.9 127.6
112.5
107.0
64.3
51.8
46.8
41.6
40.6
37.4
15.9
160 140 120 100 80 60 40
Chemical Shift (ppm)

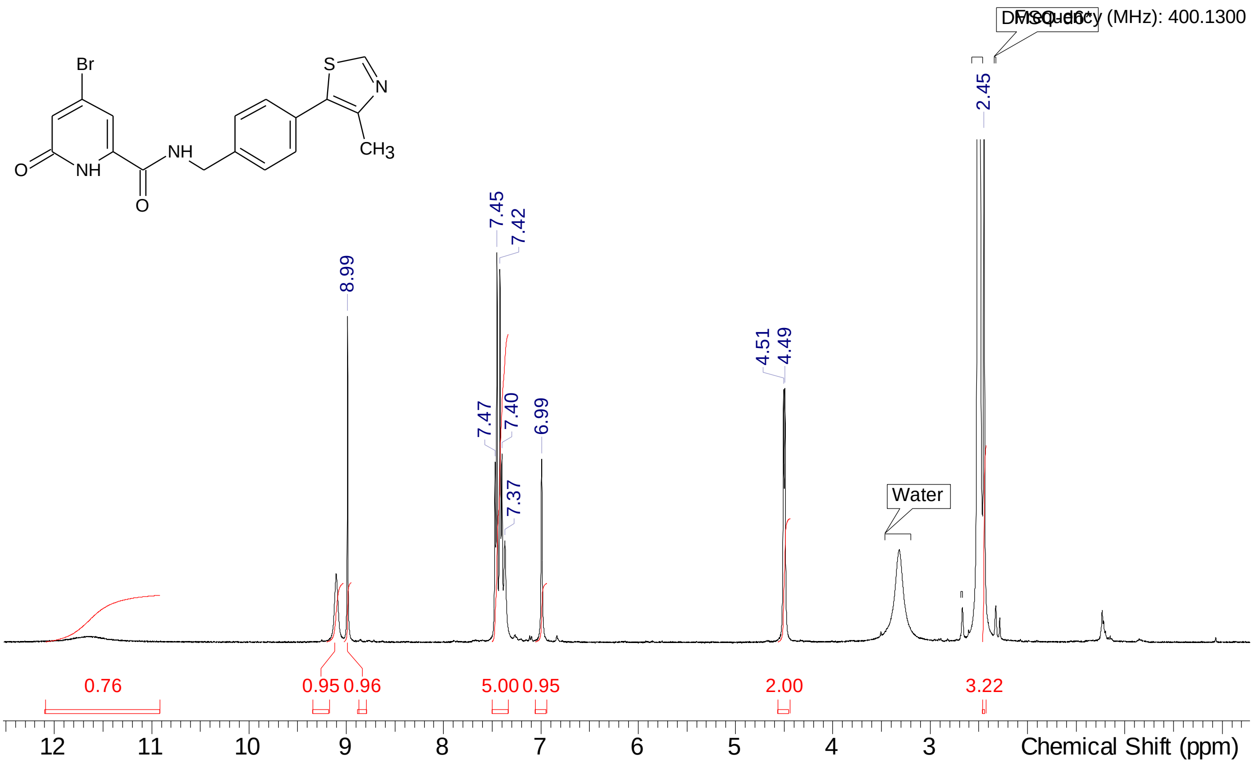
Br
S
N
NH
O
NH
O
CH3
DMSO-d6
Frequency (MHz): 400.1300
2.45
7.45
7.42
8.99
4.51
4.49
7.47
7.40
6.99
7.37
Water
0.76
0.95
0.96
5.00
0.95
2.00
3.22
12 11 10 9 8 7 6 5 4 3
Chemical Shift (ppm)

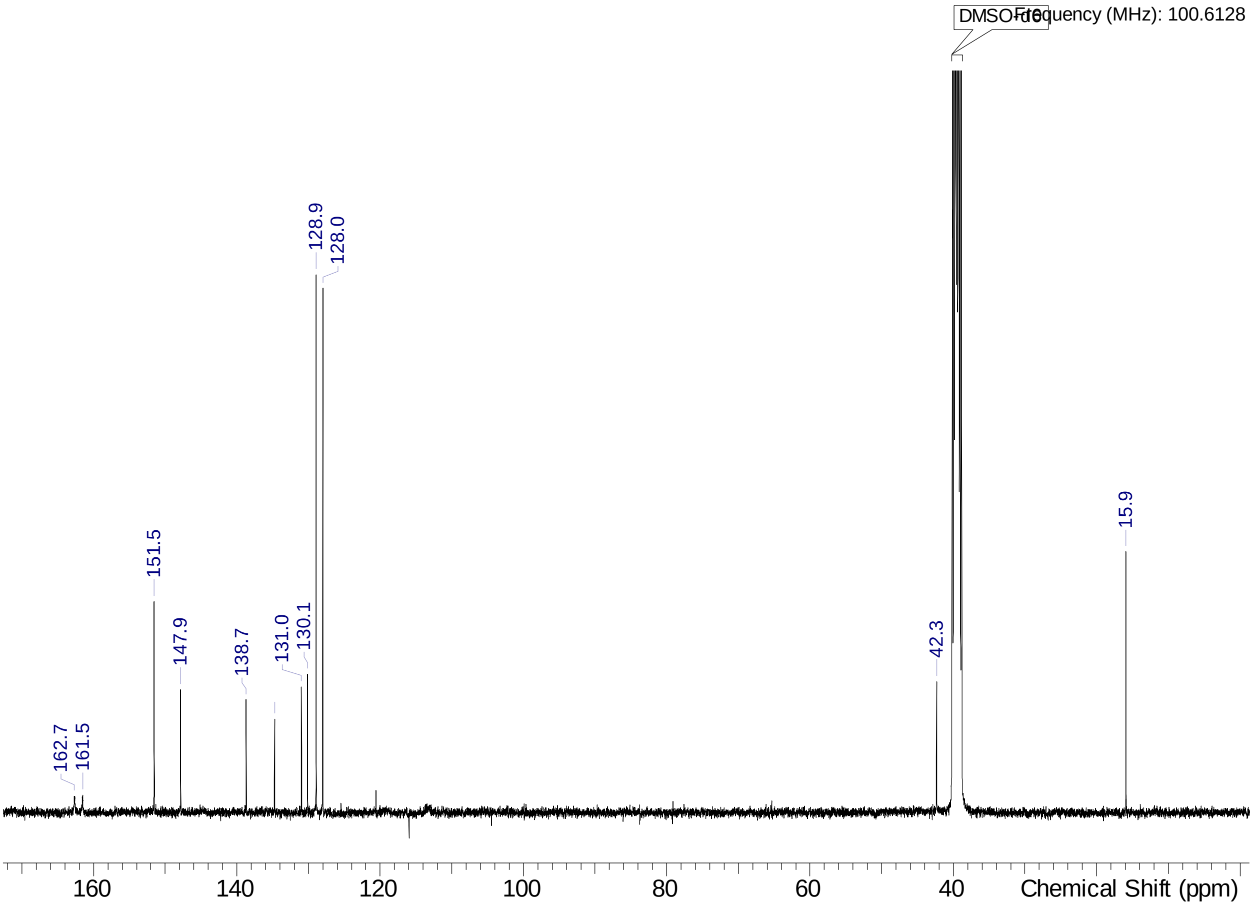
DMSO-d6
Frequency (MHz): 100.6128
128.9
128.0
151.5
147.9
138.7
131.0
130.1
162.7
161.5
42.3
15.9
160 140 120 100 80 60 40
Chemical Shift (ppm)

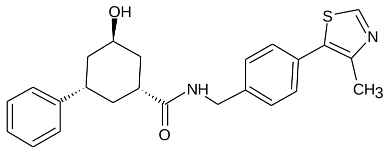
OH
NH
O
S
N
CH3

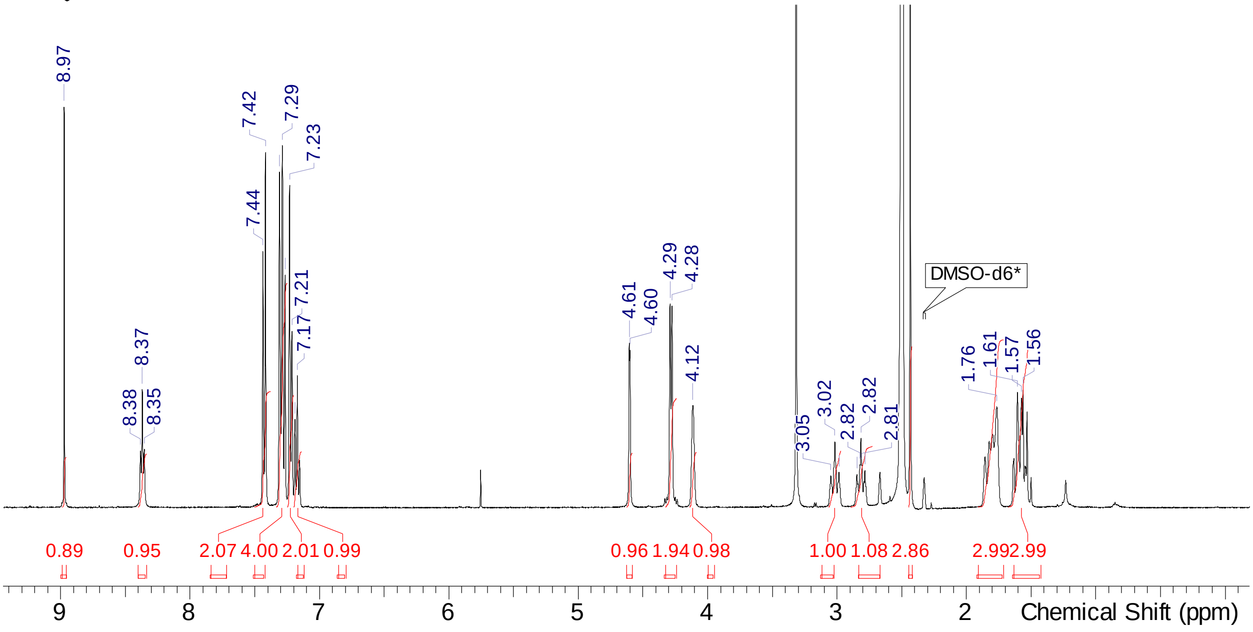
DMSO-d6*
Frequency (MHz): 400.1300
2.43
8.97
7.42
7.29
7.23
7.44
4.61
4.60
4.29
4.28
7.21
7.17
8.37
8.38
8.35
4.12
1.76
1.61
1.57
1.56
3.05
3.02
2.82
2.82
2.81
DMSO-d6*
0.89
0.95
2.07
4.00
2.01
0.99
0.96
1.94
0.98
1.00
1.08
2.86
2.99
2.99
Chemical Shift (ppm)

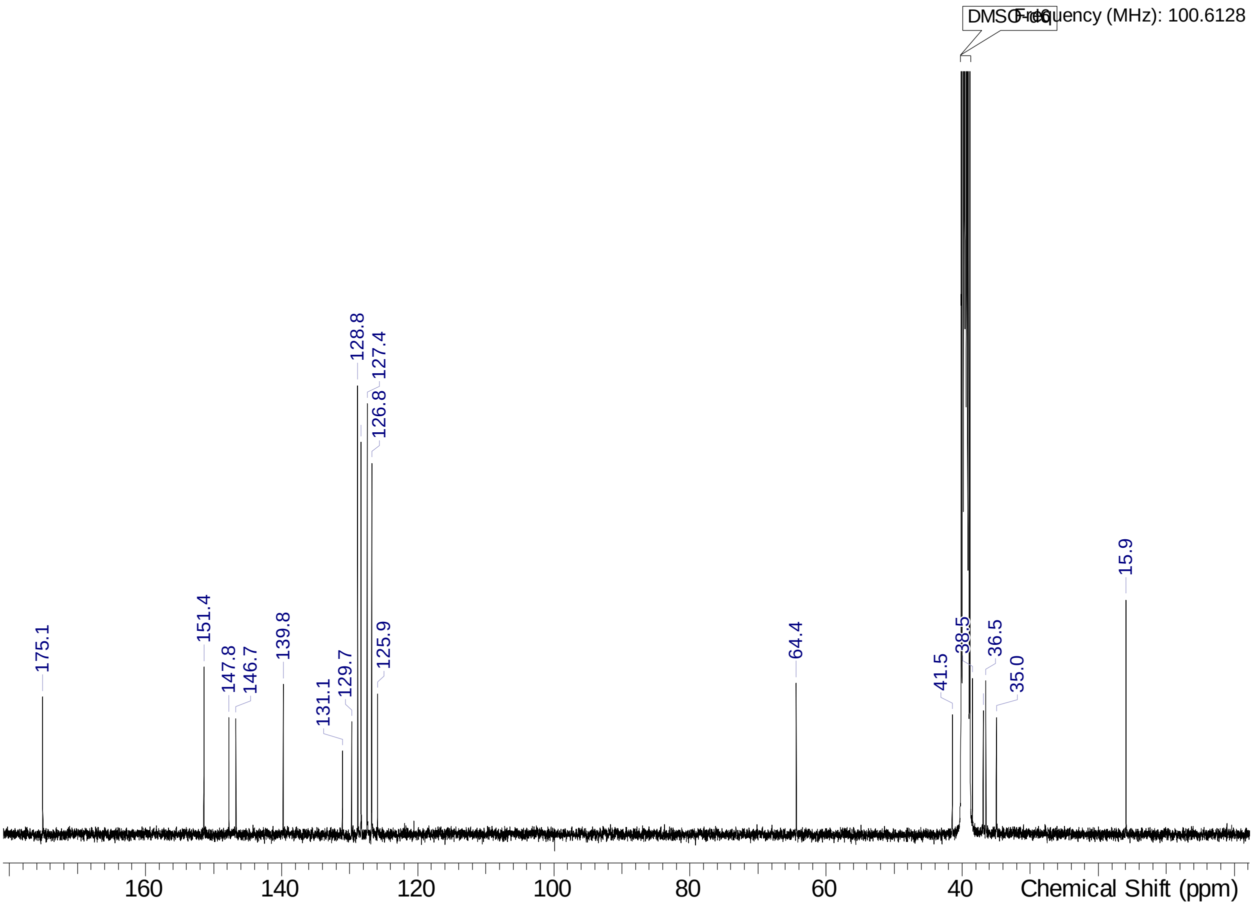
DMSO-d6
Frequency (MHz): 100.6128
128.8
127.4
126.8
175.1
151.4
147.8
146.7
139.8
131.1
129.7
125.9
64.4
41.5
38.5
36.5
35.0
15.9
Chemical Shift (ppm)

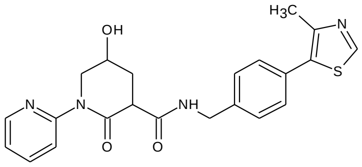
OH
N
N
O
O
NH
H3C
N
S

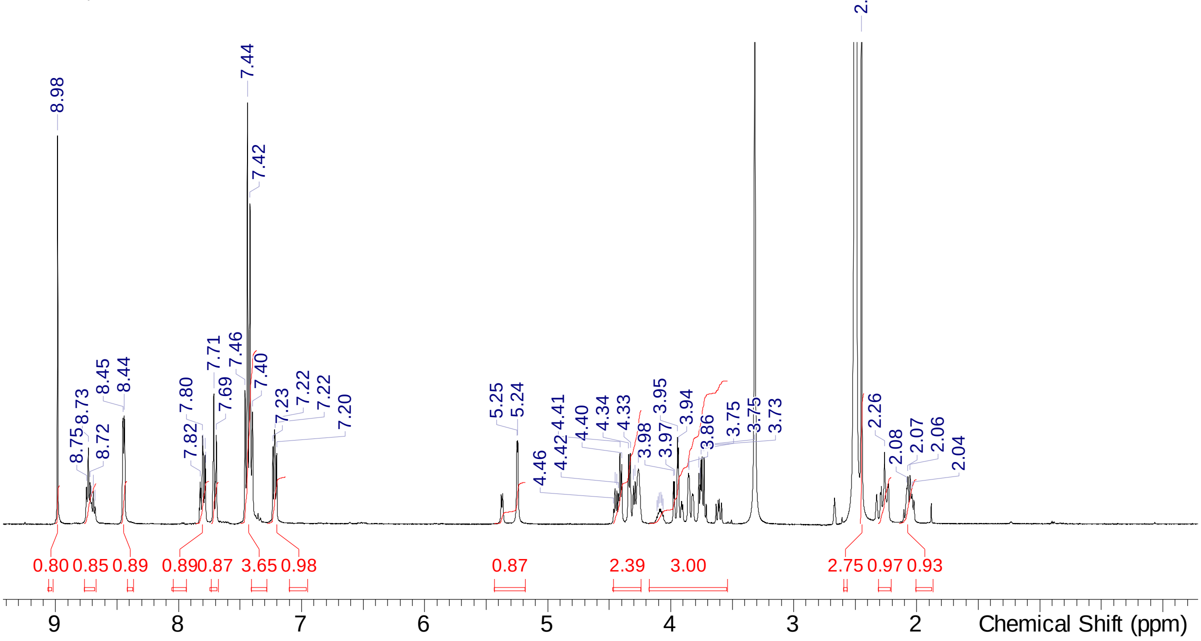
DMSO-d6
Frequency (MHz): 400.1300
8.98
8.75 8.73 8.72
8.45 8.44
7.82 7.80 7.71 7.69 7.46 7.44 7.42 7.40
7.23 7.22 7.22 7.20
5.25 5.24
4.46 4.42 4.41 4.40 4.34 4.33
3.98 3.97 3.95 3.94 3.86 3.75 3.75 3.73
2.45
2.26 2.08 2.07 2.06 2.04
0.80 0.85 0.89 0.89 0.87 3.65 0.98 0.87 2.39 3.00 2.75 0.97 0.93
9 8 7 6 5 4 3 2
Chemical Shift (ppm)

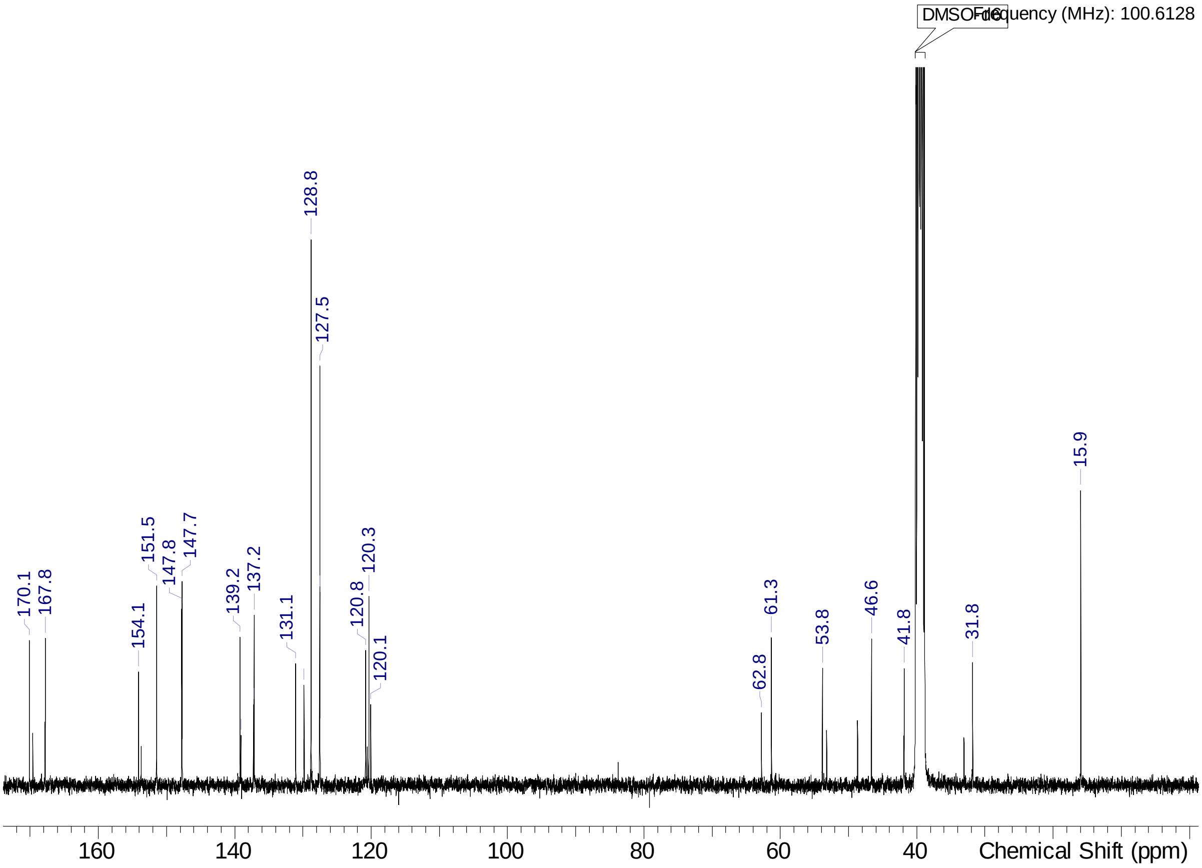
DMSO-d6
Frequency (MHz): 100.6128
170.1 167.8 154.1 151.5 147.8 147.7 139.2 137.2 131.1 128.8 127.5 120.8 120.3 120.1
62.8 61.3 53.8 46.6 41.8 31.8 15.9
160 140 120 100 80 60 40
Chemical Shift (ppm)

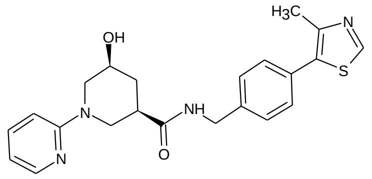
H3C
N
S
OH
N
N
NH
O

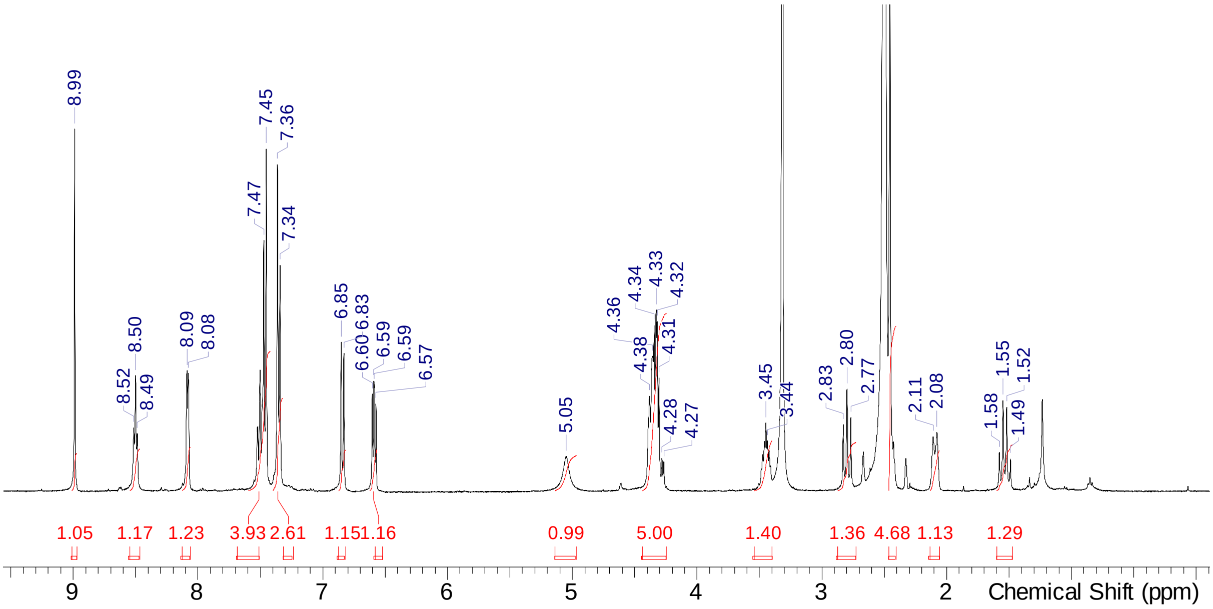
DMSO-d6
Frequency (MHz): 400.1300
8.99
8.52
8.50
8.49
8.09
8.08
7.47
7.45
7.36
7.34
6.85
6.83
6.60
6.59
6.59
6.57
5.05
4.38
4.36
4.34
4.33
4.32
4.31
4.28
4.27
3.45
3.44
2.83
2.80
2.77
2.46
2.11
2.08
1.58
1.55
1.52
1.49
1.05
1.17
1.23
3.93
2.61
1.15
1.16
0.99
5.00
1.40
1.36
4.68
1.13
1.29
9
8
7
6
5
4
3
2
Chemical Shift (ppm)

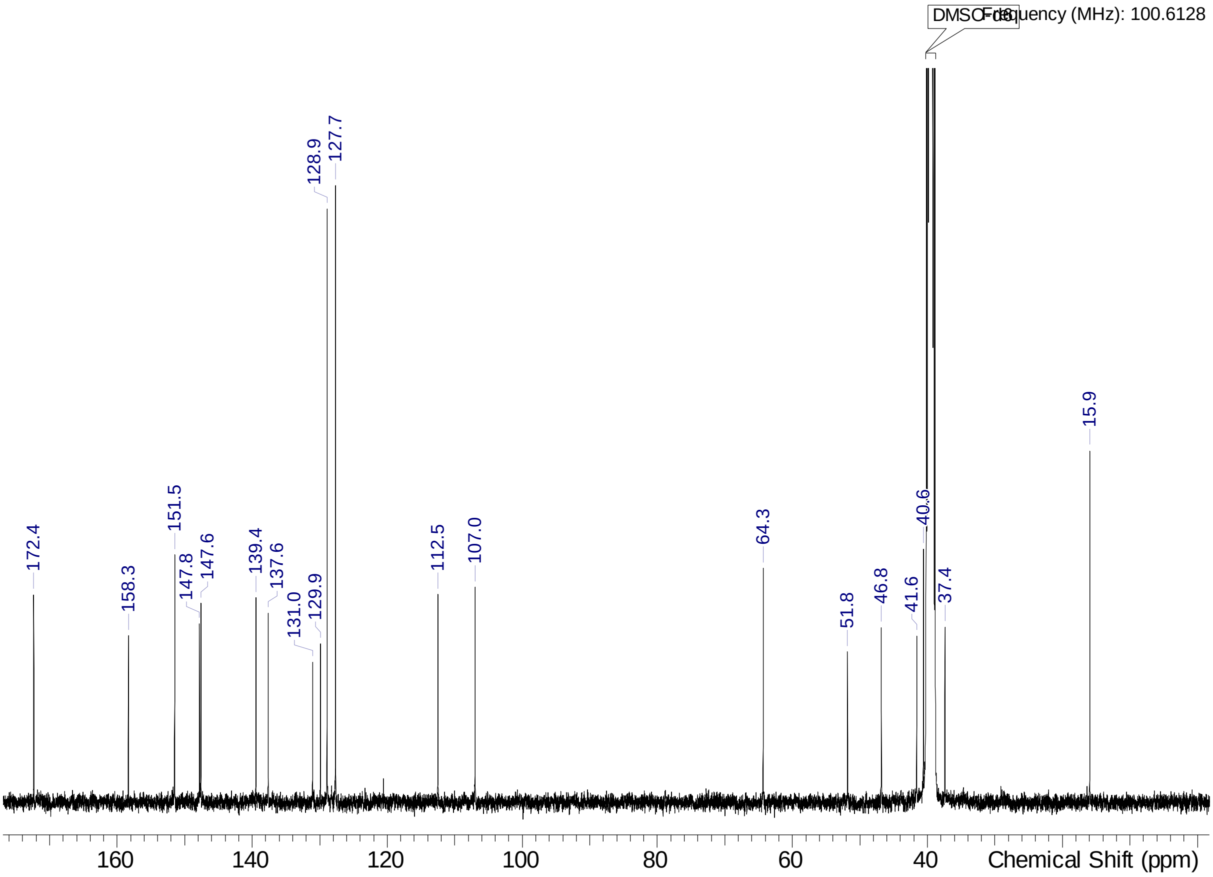
DMSO-d6
Frequency (MHz): 100.6128
172.4
158.3
151.5
147.8
147.6
139.4
137.6
131.0
129.9
128.9
127.7
112.5
107.0
64.3
51.8
46.8
41.6
40.6
37.4
15.9
160
140
120
100
80
60
40
Chemical Shift (ppm)

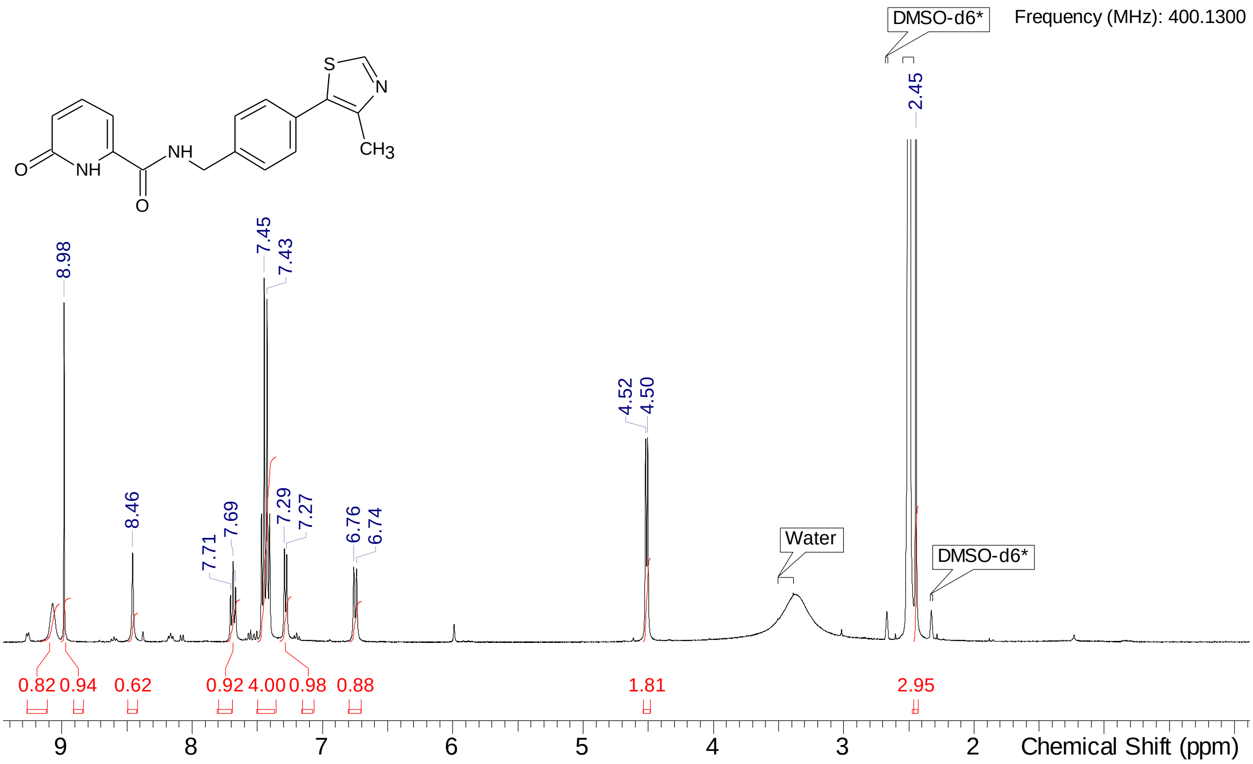
DMSO-d6*
Frequency (MHz): 400.1300
O
NH
O
NH
S
N
CH3
8.98
7.45
7.43
4.52
4.50
8.46
7.71
7.69
7.29
7.27
6.76
6.74
Water
2.45
DMSO-d6*
0.82 0.94 0.62 0.92 4.00 0.98 0.88 1.81 2.95
9 8 7 6 5 4 3 2
Chemical Shift (ppm)

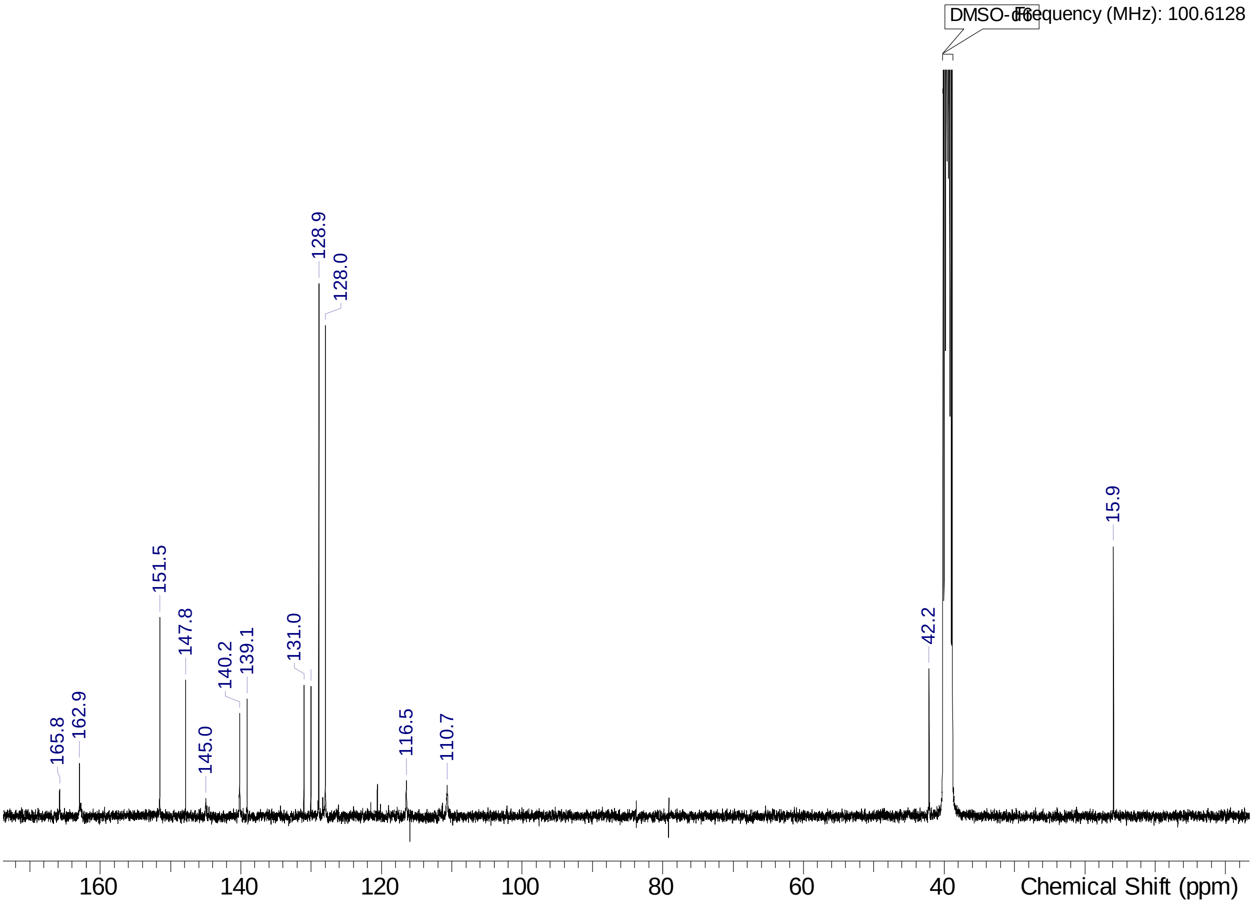
DMSO-d6
Frequency (MHz): 100.6128
128.9
128.0
151.5
147.8
140.2
139.1
131.0
165.8
162.9
145.0
116.5
110.7
42.2
15.9
160 140 120 100 80 60 40
Chemical Shift (ppm)

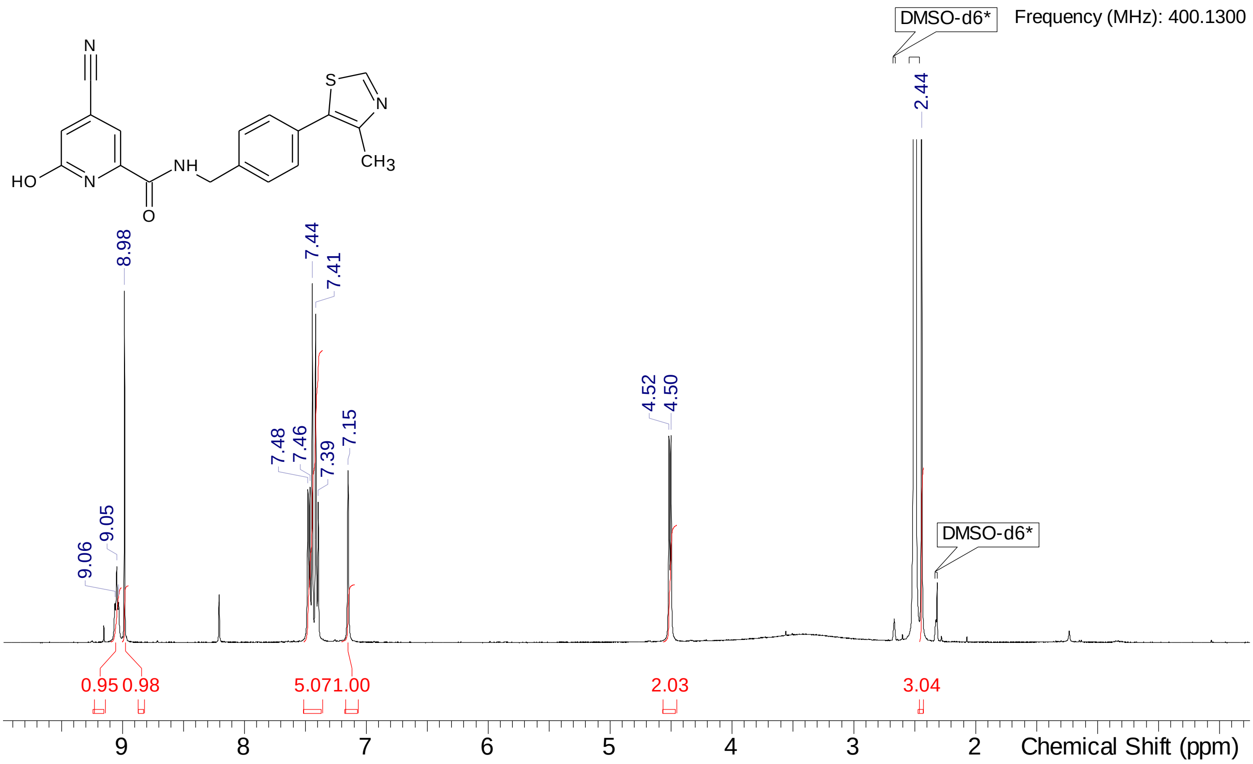
HO
N
NH
O
N
S
N
CH3
DMSO-d6*
Frequency (MHz): 400.1300
2.44
8.98
7.44
7.41
4.52
4.50
7.48
7.46
7.39
7.15
9.05
9.06
DMSO-d6*
0.95 0.98
5.07 1.00
2.03
3.04
Chemical Shift (ppm)

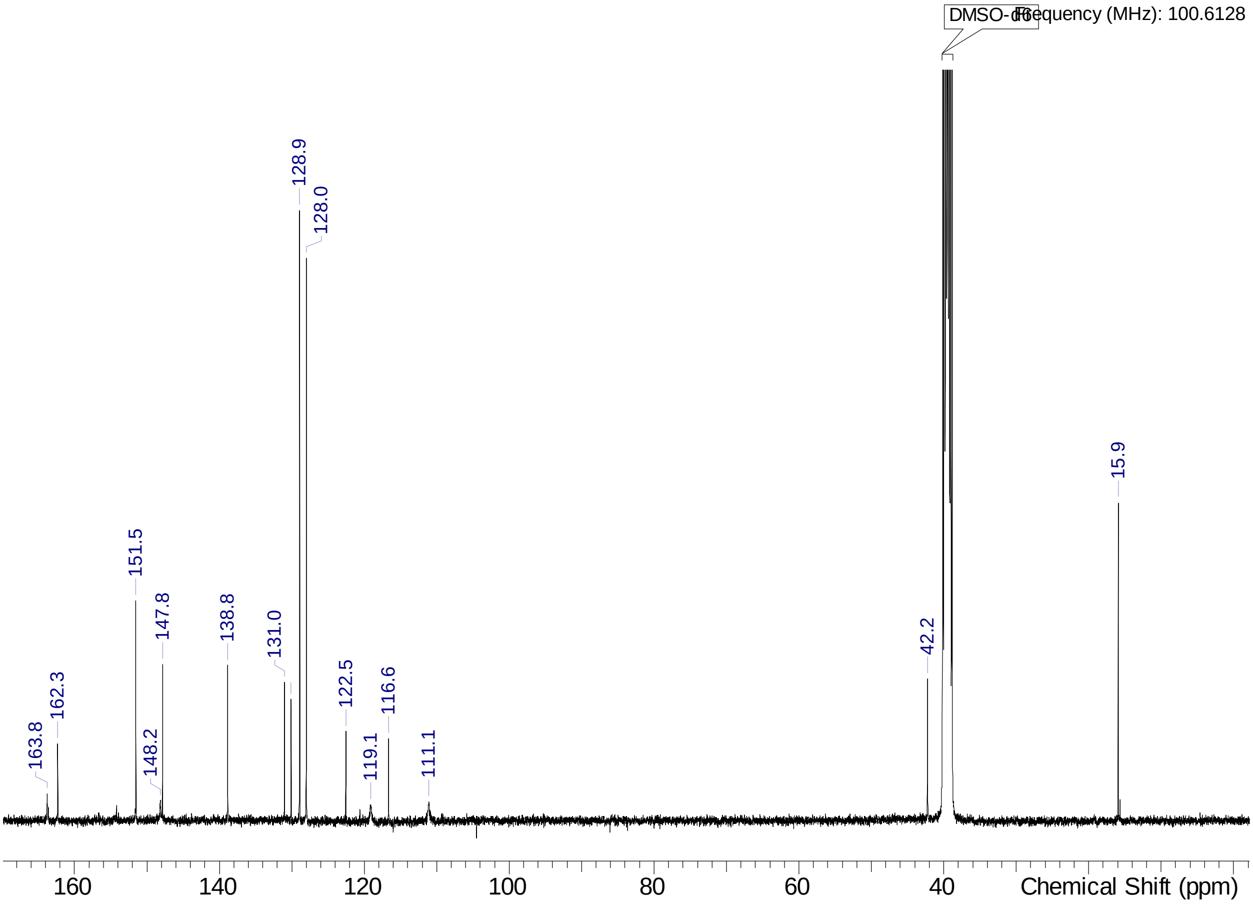
DMSO-d6
Frequency (MHz): 100.6128
128.9
128.0
15.9
151.5
147.8
138.8
131.0
42.2
162.3
122.5
116.6
163.8
148.2
119.1
111.1
Chemical Shift (ppm)

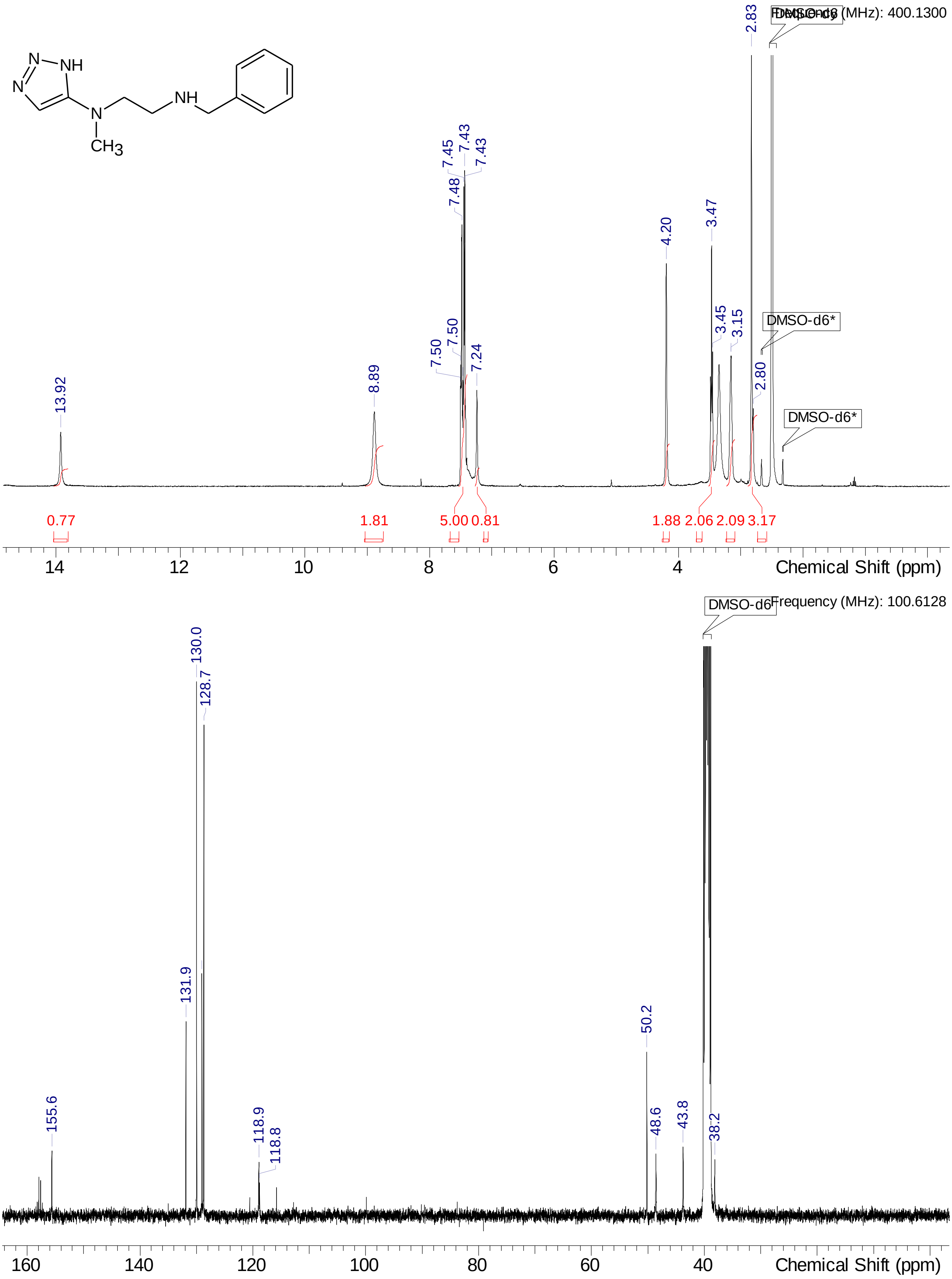

Frequency (MHz): 400.1300
DMSO-d6
DMSO-d6*
13.92
8.89
7.50
7.50
7.48
7.45
7.43
7.43
7.24
4.20
3.47
3.45
3.15
2.83
2.80
0.77
1.81
5.00
0.81
1.88
2.06
2.09
3.17
Chemical Shift (ppm)
Frequency (MHz): 100.6128
155.6
131.9
130.0
128.7
118.9
118.8
50.2
48.6
43.8
38.2
Chemical Shift (ppm)

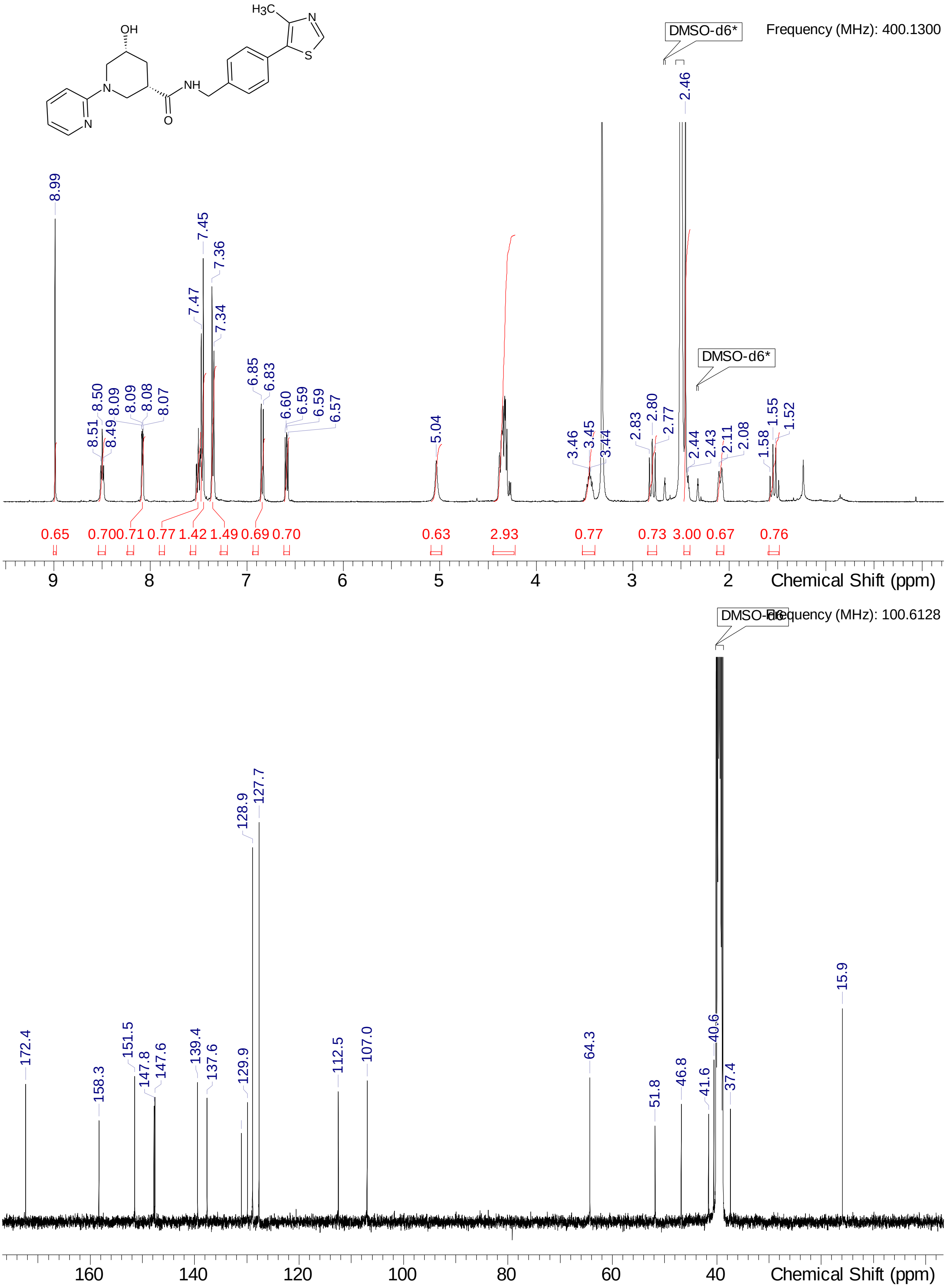
OH
NH
O
N
H3C
S
DMSO-d6*
Frequency (MHz): 400.1300
8.99
8.51 8.50 8.49 8.09 8.09 8.08 8.07
7.47 7.45 7.36 7.34
6.85 6.83 6.60 6.59 6.59 6.57
5.04
3.46 3.45 3.44
2.83 2.80 2.77
2.46
2.44 2.43 2.11 2.08
1.58 1.55 1.52
0.65 0.70 0.71 0.77 1.42 1.49 0.69 0.70 0.63 2.93 0.77 0.73 3.00 0.67 0.76
Chemical Shift (ppm)
DMSO-d6
Frequency (MHz): 100.6128
172.4
158.3
151.5 147.8 147.6
139.4 137.6
129.9 128.9 127.7
112.5 107.0
64.3
51.8 46.8 41.6 40.6 37.4
15.9
Chemical Shift (ppm)

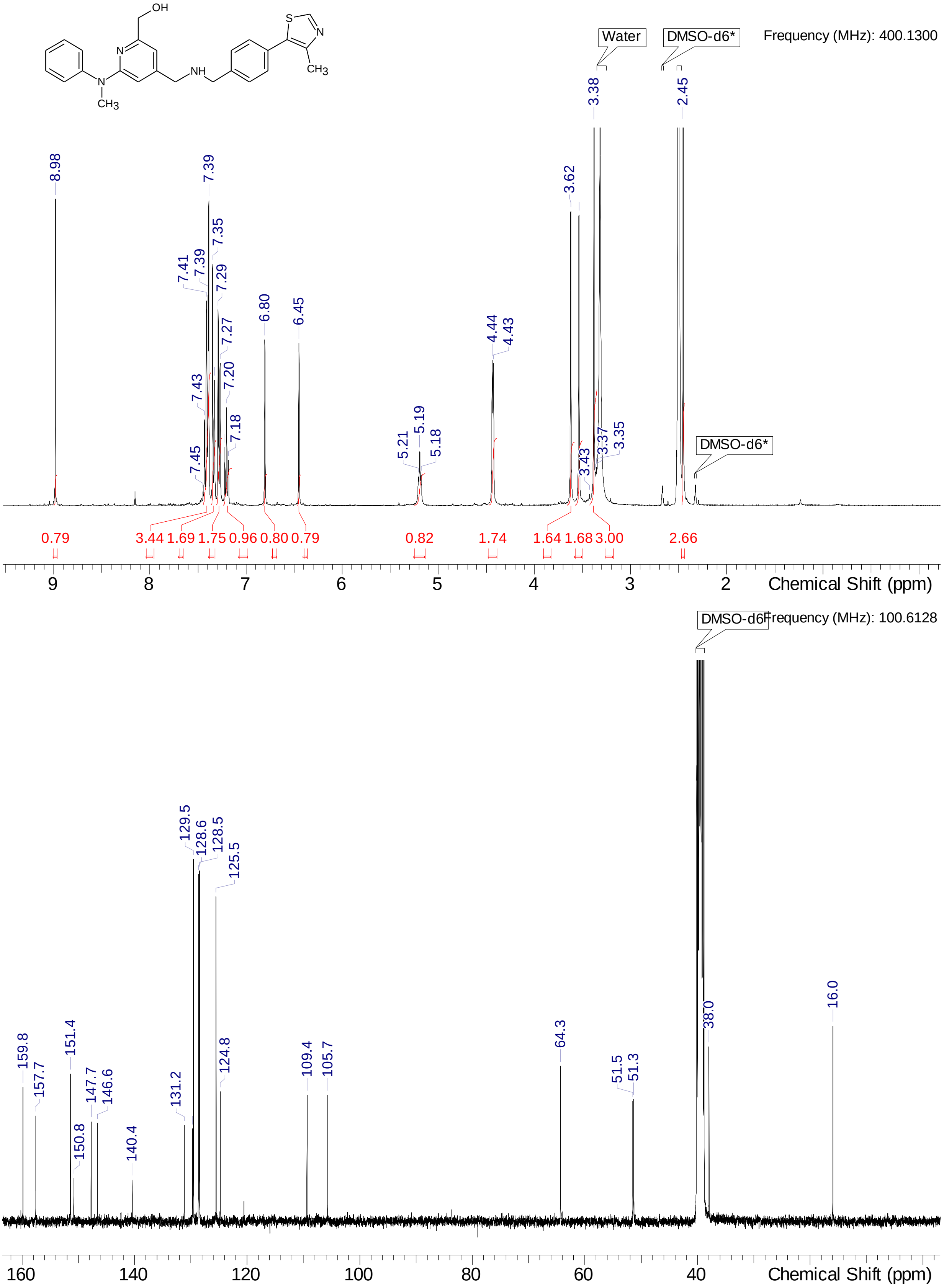
OH
N
N
CH3
NH
S
N
CH3
Water
DMSO-d6*
Frequency (MHz): 400.1300
8.98
7.45
7.43
7.41
7.39
7.39
7.35
7.29
7.27
7.20
7.18
6.80
6.45
5.21
5.19
5.18
4.44
4.43
3.62
3.43
3.38
3.37
3.35
2.45
DMSO-d6*
0.79
3.44
1.69
1.75
0.96
0.80
0.79
0.82
1.74
1.64
1.68
3.00
2.66
9
8
7
6
5
4
3
2
Chemical Shift (ppm)
DMSO-d6
Frequency (MHz): 100.6128
159.8
157.7
151.4
150.8
147.7
146.6
140.4
131.2
129.5
128.6
128.5
125.5
124.8
109.4
105.7
64.3
51.5
51.3
38.0
16.0
160
140
120
100
80
60
40
Chemical Shift (ppm)

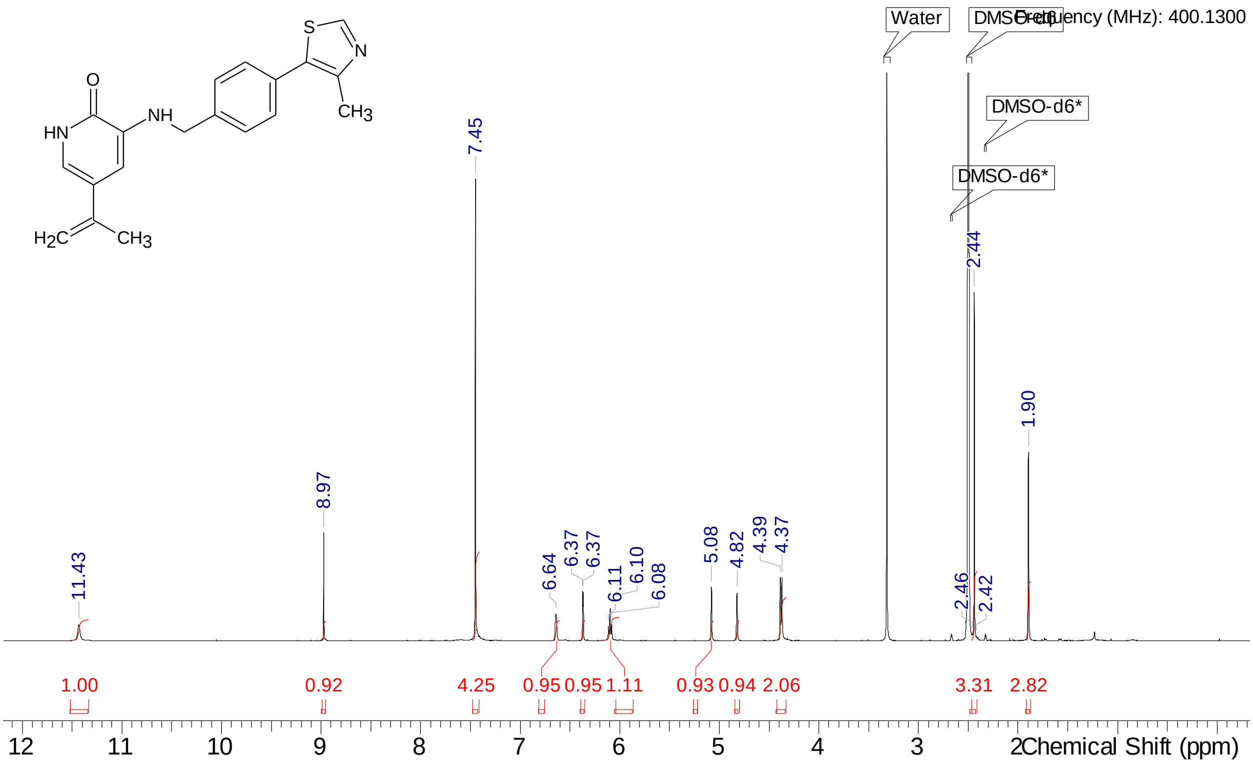
Water
DMSO-d6
Frequency (MHz): 400.1300
DMSO-d6*
DMSO-d6*
11.43
8.97
7.45
6.64
6.37
6.37
6.11
6.10
6.08
5.08
4.82
4.39
4.37
2.46
2.44
2.42
1.90
1.00
0.92
4.25
0.95
0.95
1.11
0.93
0.94
2.06
3.31
2.82
Chemical Shift (ppm)

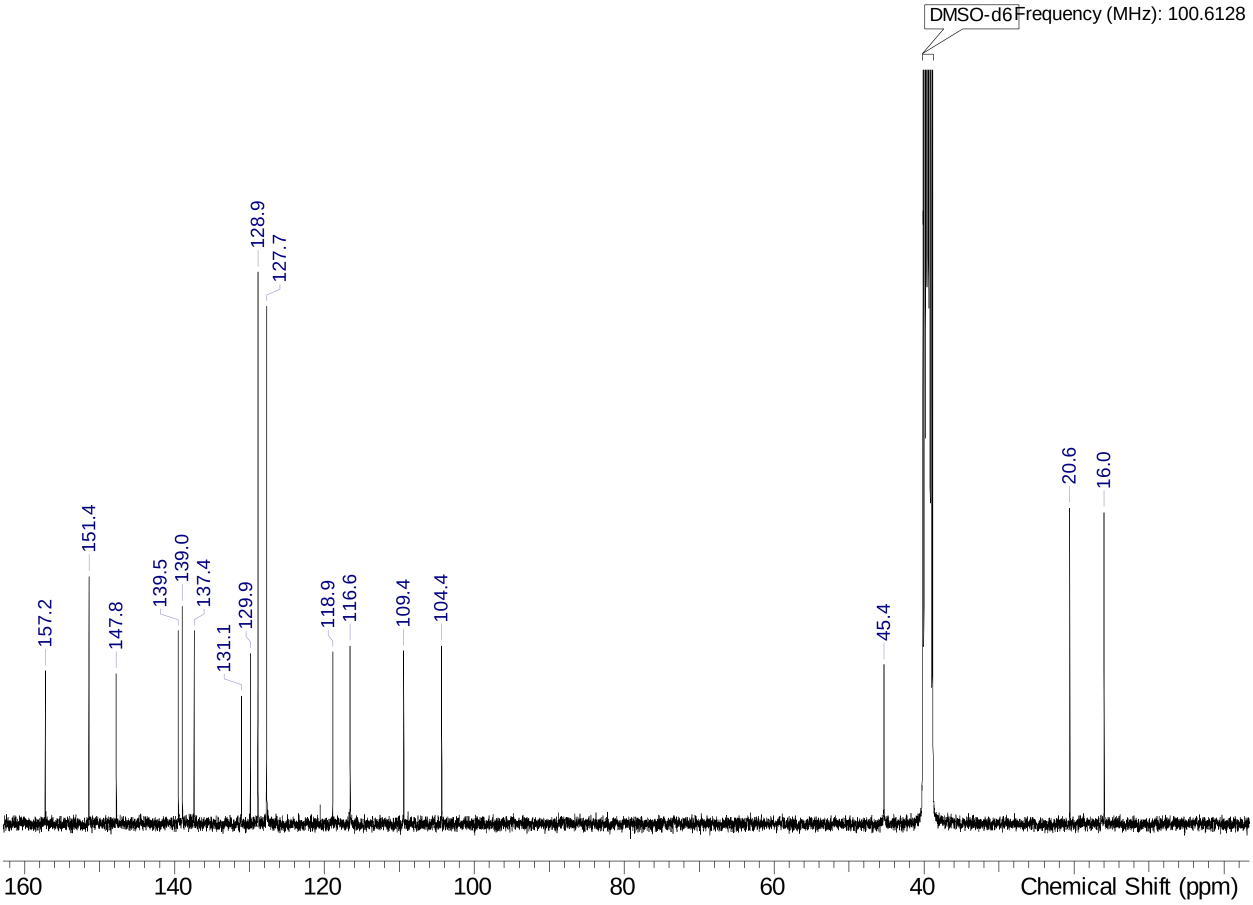
DMSO-d6
Frequency (MHz): 100.6128
157.2
151.4
147.8
139.5
139.0
137.4
131.1
129.9
128.9
127.7
118.9
116.6
109.4
104.4
45.4
20.6
16.0
Chemical Shift (ppm)

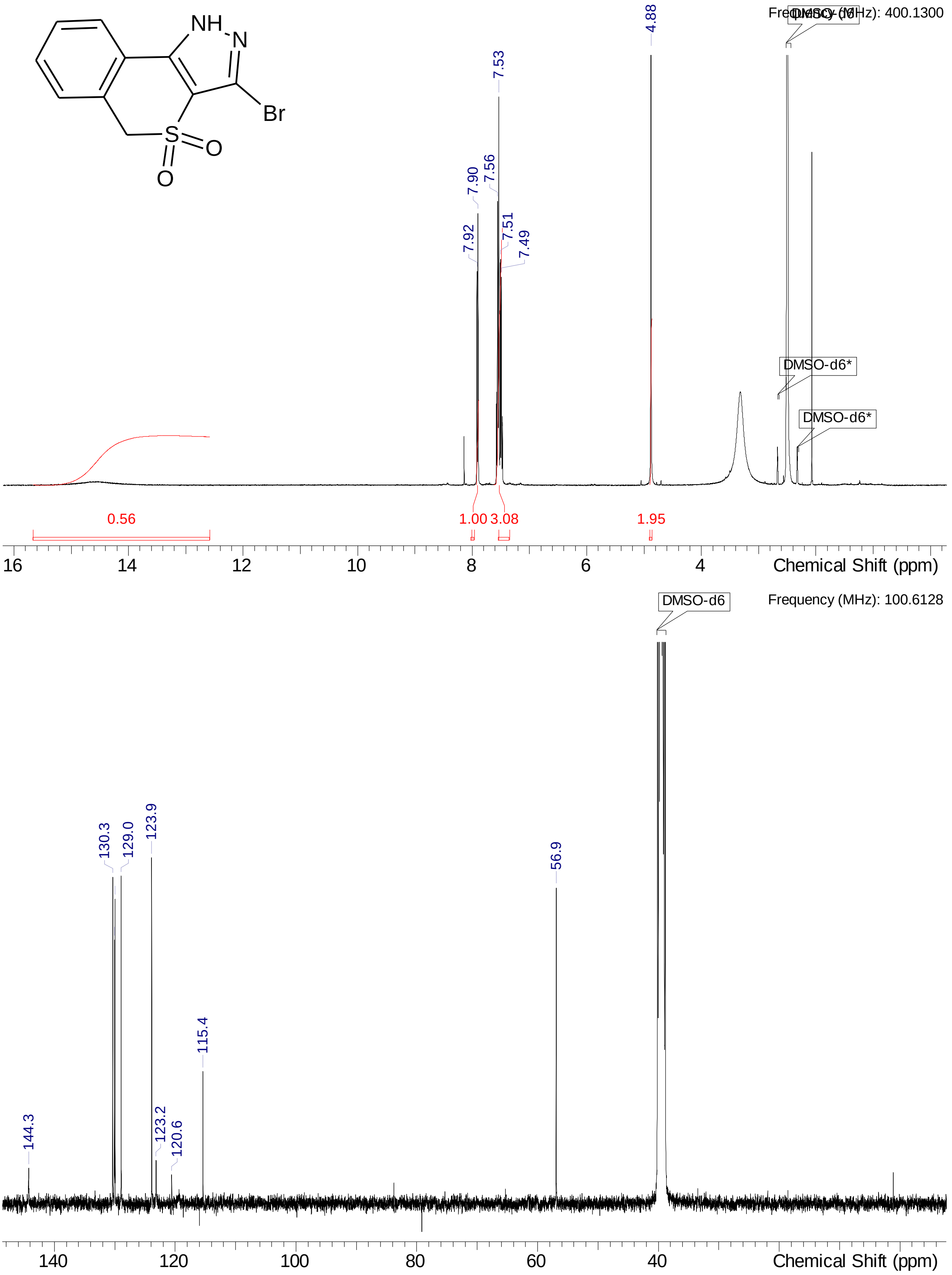
NH
N
Br
S
O
O
4.88
7.53
7.56
7.90
7.92
7.51
7.49
Frequency (MHz): 400.1300
DMSO-d6
DMSO-d6*
DMSO-d6*
0.56
1.00
3.08
1.95
16
14
12
10
8
6
4
Chemical Shift (ppm)
DMSO-d6
Frequency (MHz): 100.6128
130.3
129.0
123.9
56.9
115.4
144.3
123.2
120.6
140
120
100
80
60
40
Chemical Shift (ppm)

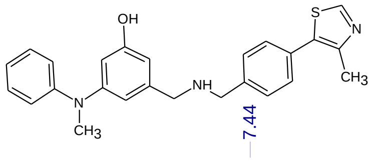
OH
NH
S
N
CH3
N
CH3

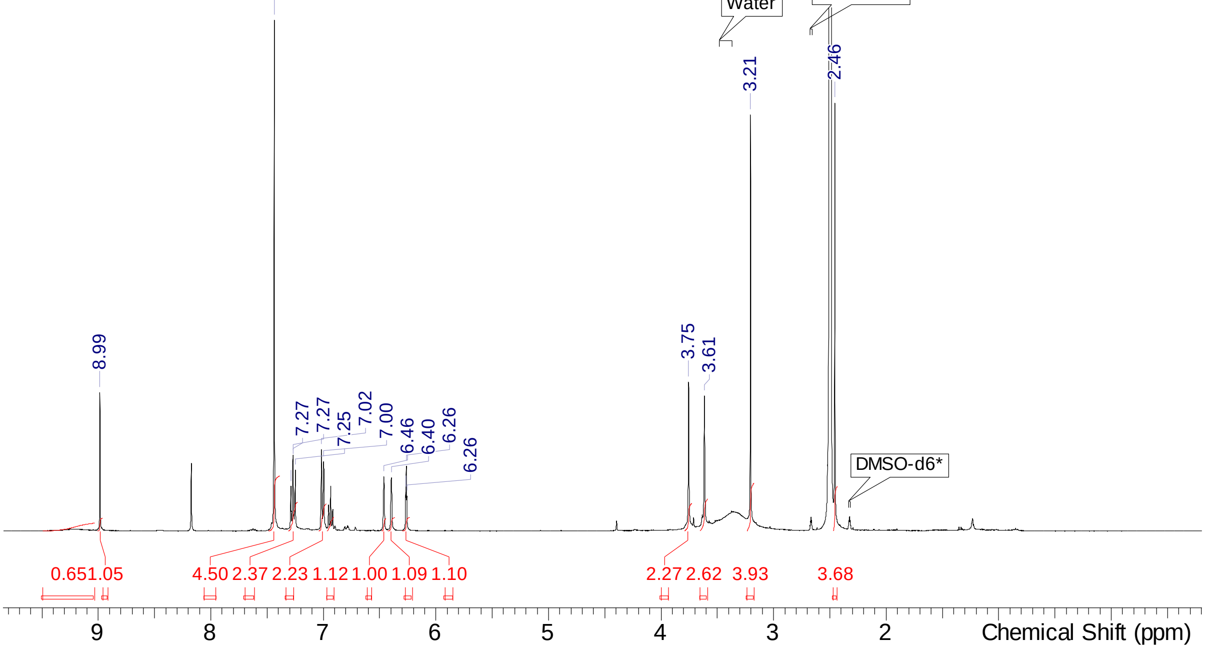
Frequency (MHz): 400.1300
Water
DMSO-d6*
7.44
3.21
2.46
8.99
3.75
3.61
7.27
7.27
7.25
7.02
7.00
6.46
6.40
6.26
6.26
DMSO-d6*
0.65
1.05
4.50
2.37
2.23
1.12
1.00
1.09
1.10
2.27
2.62
3.93
3.68
Chemical Shift (ppm)

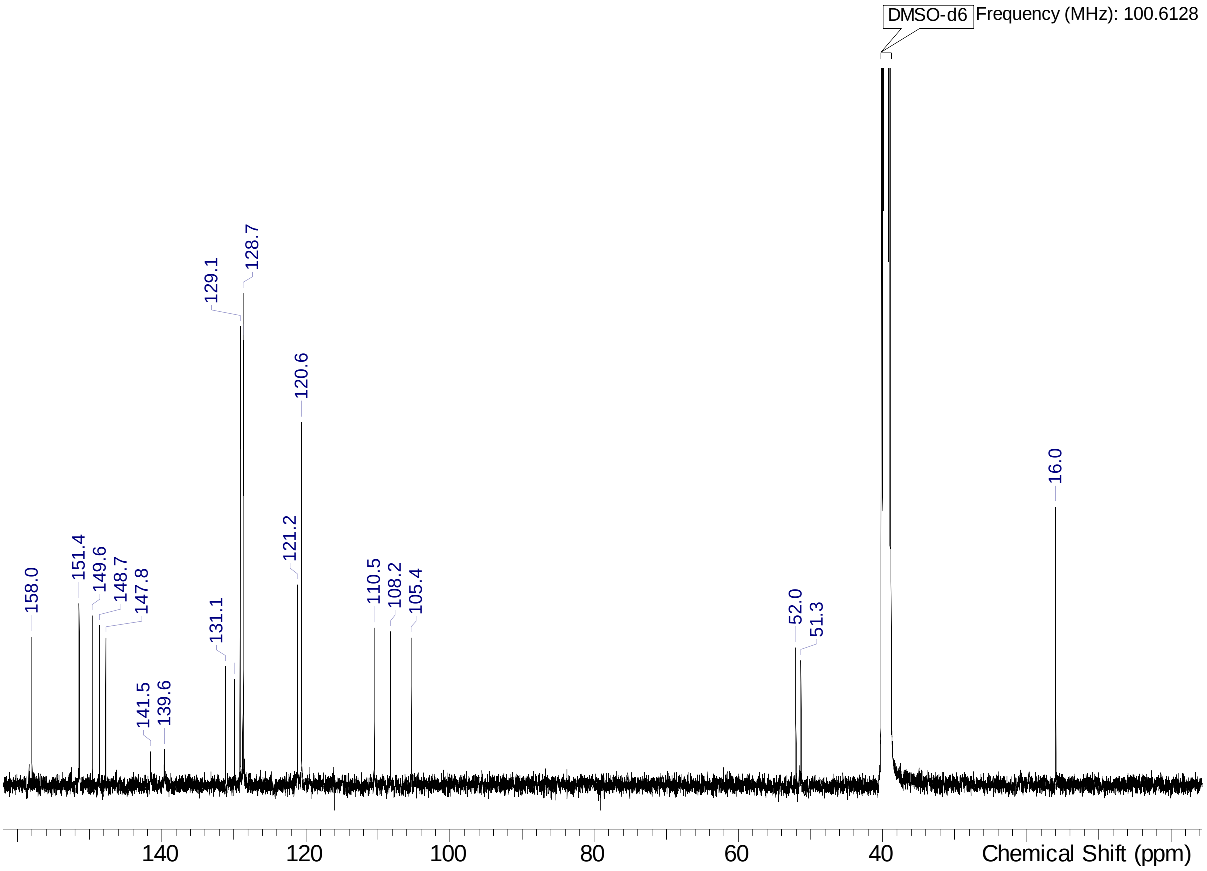
DMSO-d6
Frequency (MHz): 100.6128
129.1
128.7
120.6
16.0
121.2
158.0
151.4
149.6
148.7
147.8
131.1
110.5
108.2
105.4
52.0
51.3
141.5
139.6
Chemical Shift (ppm)

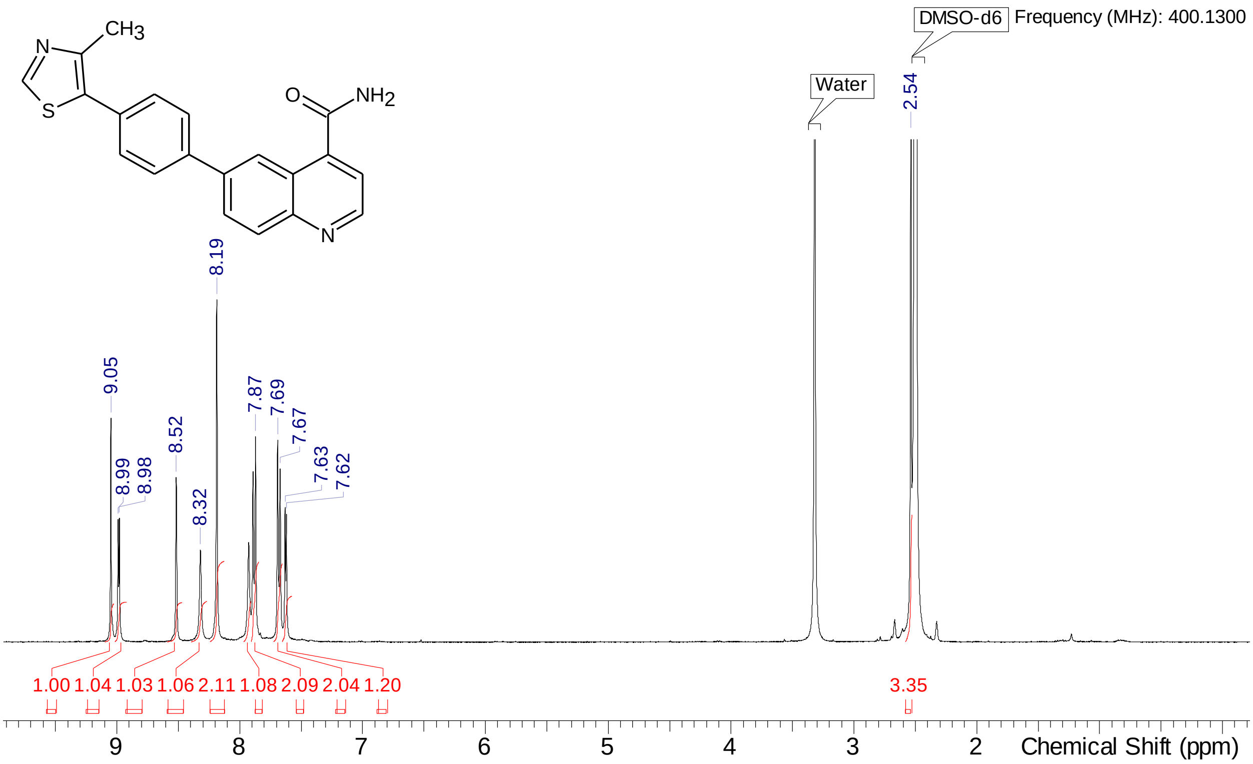
CH3
N
S
O
NH2
N
DMSO-d6 Frequency (MHz): 400.1300
Water
2.54
9.05
8.99
8.98
8.52
8.32
8.19
7.87
7.69
7.67
7.63
7.62
1.00 1.04 1.03 1.06 2.11 1.08 2.09 2.04 1.20
3.35
9 8 7 6 5 4 3 2 Chemical Shift (ppm)

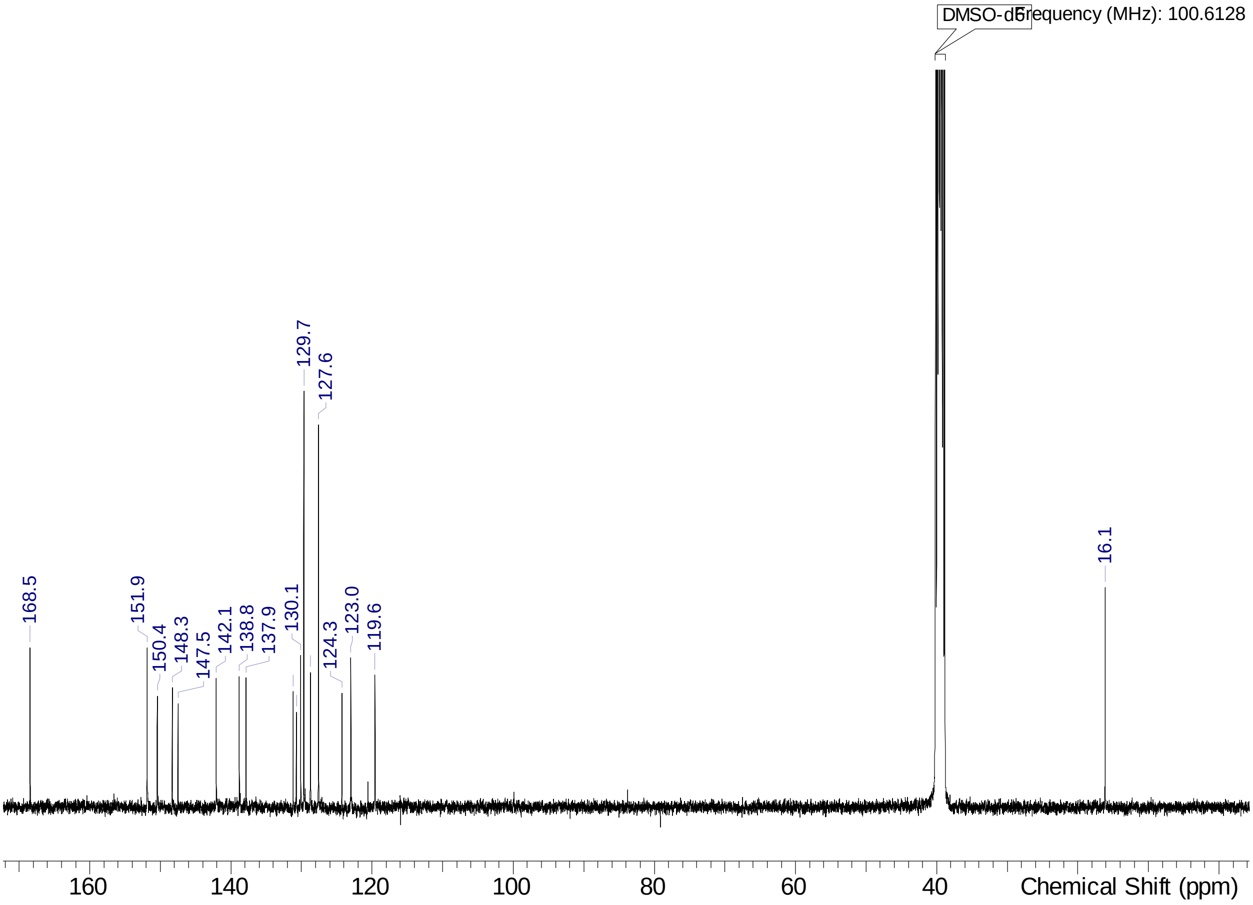
DMSO-d6 Frequency (MHz): 100.6128
168.5
151.9
150.4
148.3
147.5
142.1
138.8
137.9
130.1
129.7
127.6
124.3
123.0
119.6
16.1
160 140 120 100 80 60 40 Chemical Shift (ppm)

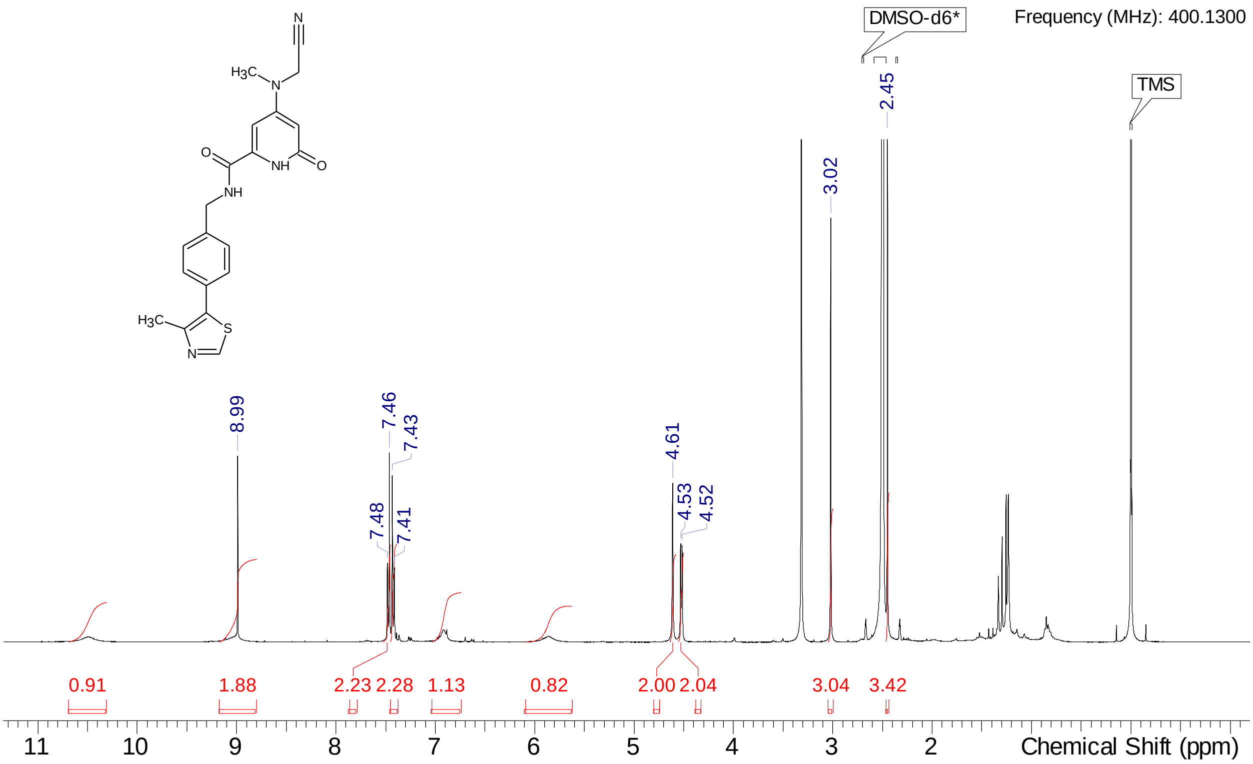
N
H3C
N
O
NH
O
NH
H3C
S
N
DMSO-d6*
Frequency (MHz): 400.1300
2.45
TMS
3.02
8.99
7.46
7.43
7.48
7.41
4.61
4.53
4.52
0.91
1.88
2.23
2.28
1.13
0.82
2.00
2.04
3.04
3.42
11
10
9
8
7
6
5
4
3
2
Chemical Shift (ppm)

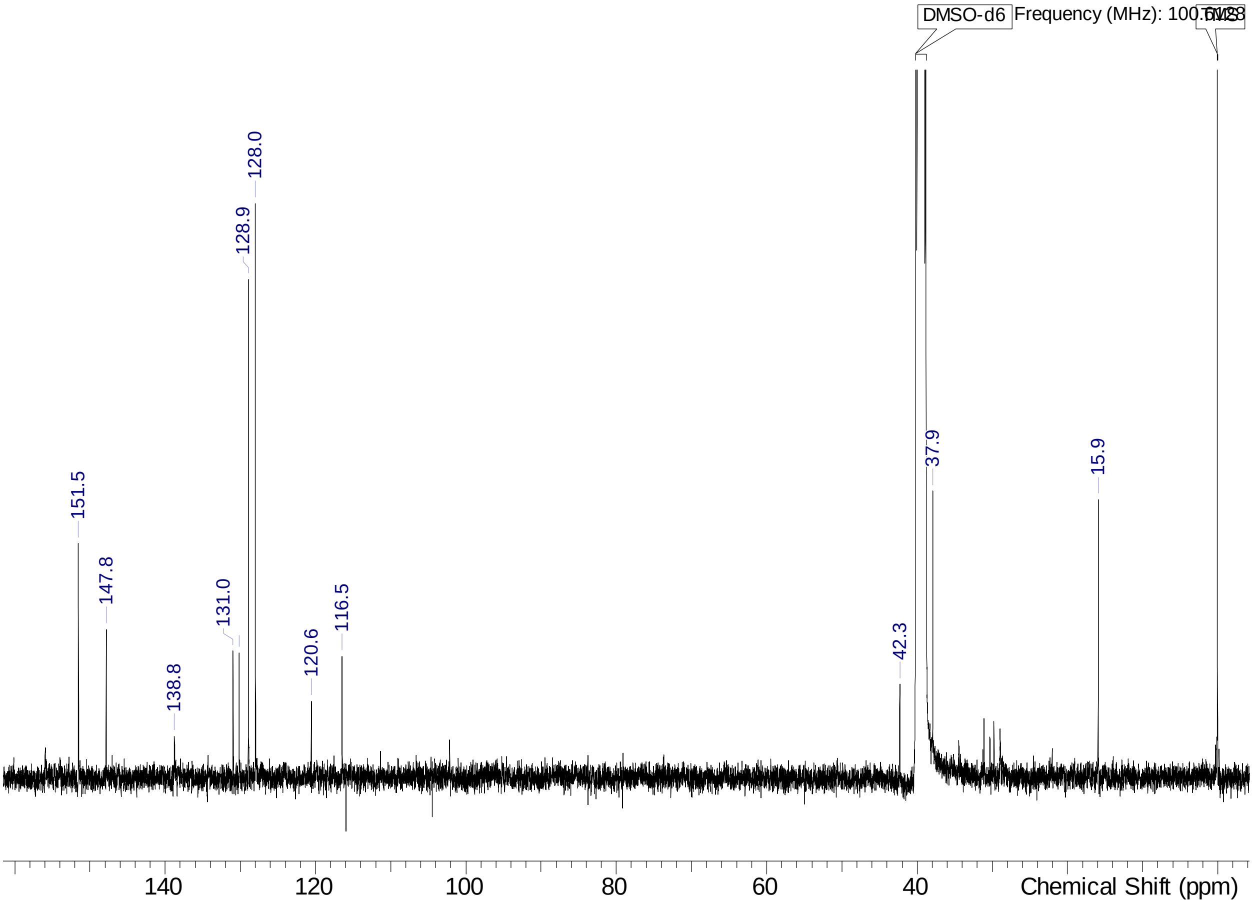
DMSO-d6
Frequency (MHz): 100.6128
TMS
128.0
128.9
151.5
147.8
138.8
131.0
120.6
116.5
42.3
37.9
15.9
140
120
100
80
60
40
Chemical Shift (ppm)

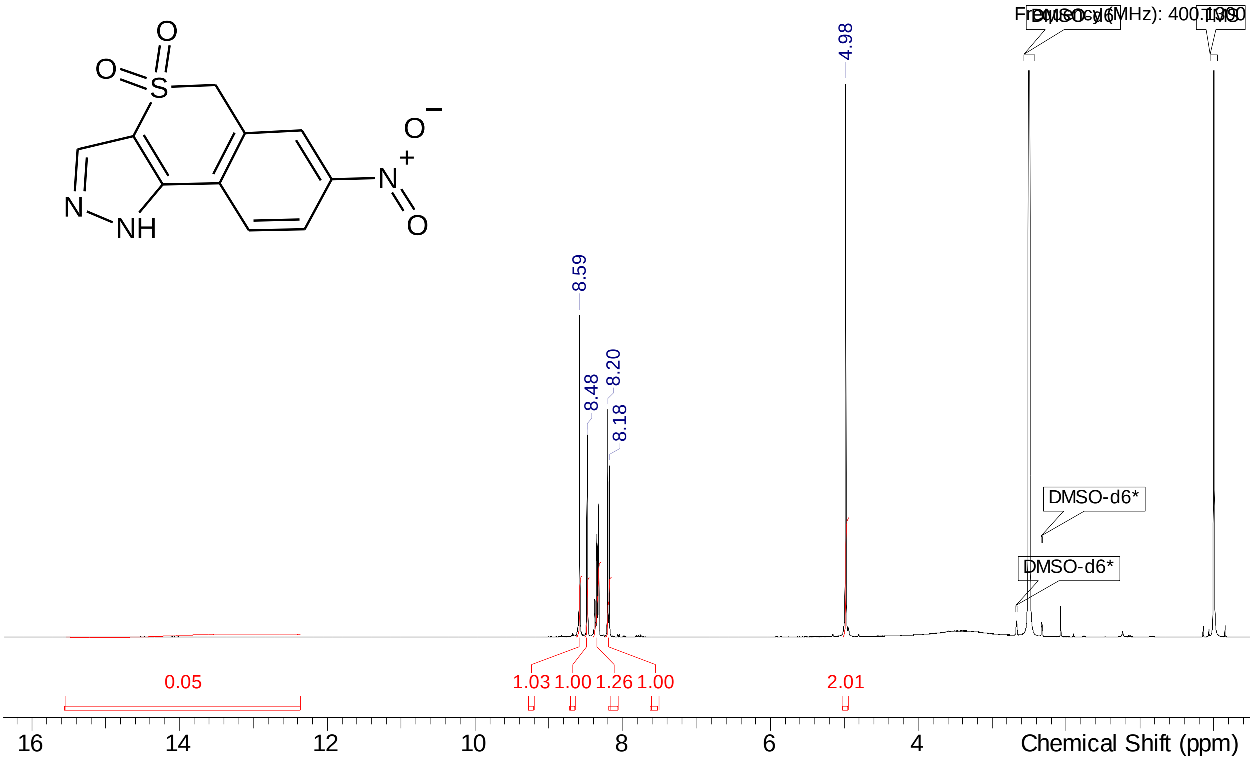
O
O
S
N
NH
N
+
O
−
O
Frequency (MHz): 400.130
DMSO-d6
TMS
4.98
8.59
8.48
8.20
8.18
DMSO-d6*
DMSO-d6*
0.05
1.03 1.00 1.26 1.00
2.01
16
14
12
10
8
6
4
Chemical Shift (ppm)

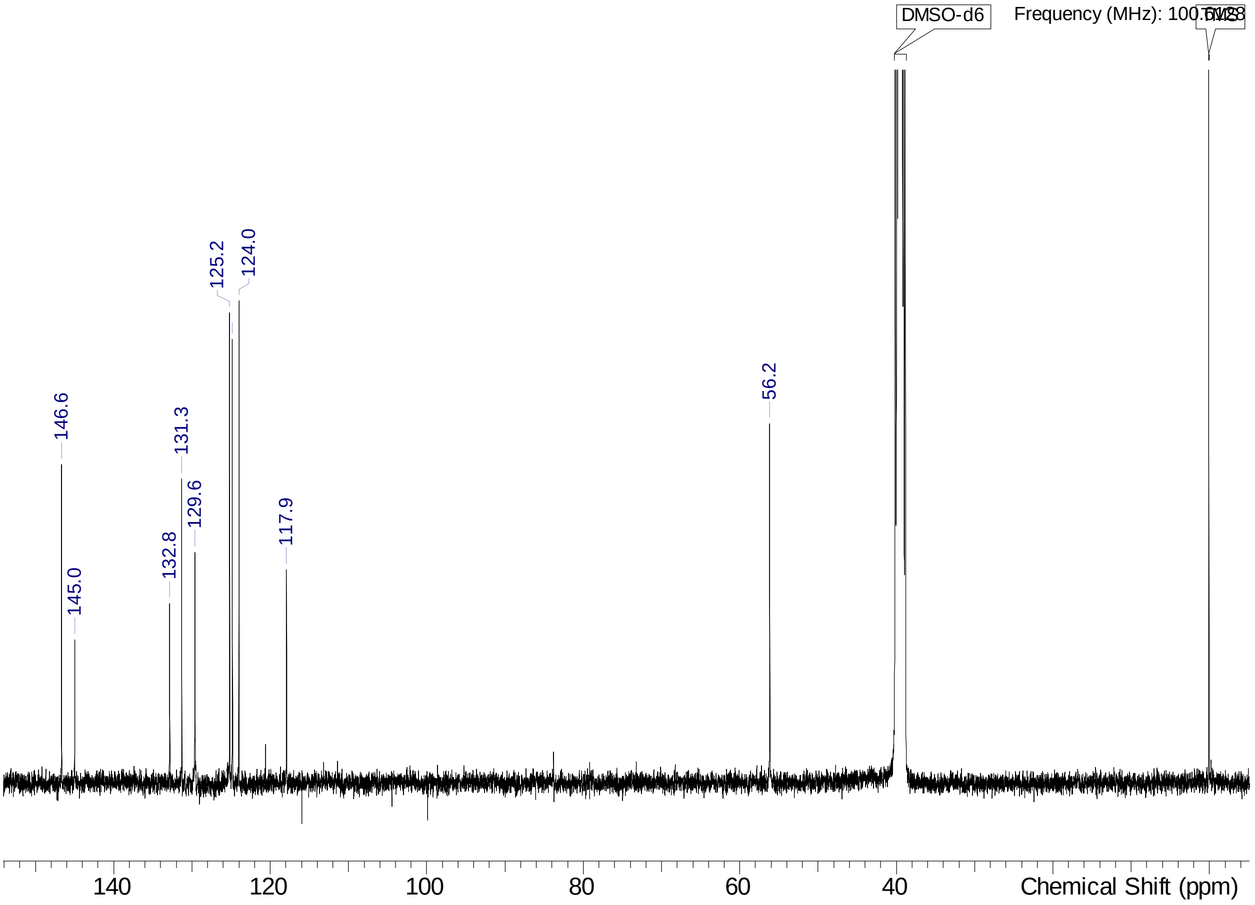
DMSO-d6
Frequency (MHz): 100.628
TMS
124.0
125.2
56.2
146.6
131.3
129.6
117.9
132.8
145.0
140
120
100
80
60
40
Chemical Shift (ppm)

Supplementary References

1. Bhardwaj, G. *et al.* Accurate de novo design of hyperstable constrained peptides. *Nature* **538**, 329–335 (2016).
