# Supplementary material for: Drugit: crowd-sourcing molecular design of non-peptidic VHL binders
Source: Nat Commun. 2025 Apr 14;16:3548. doi: 10.1038/s41467-025-58406-0 (PMC11997059; doi:10.1038/s41467-025-58406-0)
Supplement: Supplementary file 2 — Reporting Summary [file 41467_2025_58406_MOESM2_ESM.pdf]

nature portfolio

Corresponding author(s): Rocco Moretti; Jark Böttcher; Jens Meiler

Last updated by author(s): Feb 20, 2025

Reporting Summary

Nature Portfolio wishes to improve the reproducibility of the work that we publish. This form provides structure for consistency and transparency in reporting. For further information on Nature Portfolio policies, see our Editorial Policies and the Editorial Policy Checklist.

Statistics

For all statistical analyses, confirm that the following items are present in the figure legend, table legend, main text, or Methods section.

n/a	Confirmed	
x	☐	The exact sample size (*n*) for each experimental group/condition, given as a discrete number and unit of measurement
x	☐	A statement on whether measurements were taken from distinct samples or whether the same sample was measured repeatedly
x	☐	The statistical test(s) used AND whether they are one- or two-sided<br>*Only common tests should be described solely by name; describe more complex techniques in the Methods section.*
x	☐	A description of all covariates tested
x	☐	A description of any assumptions or corrections, such as tests of normality and adjustment for multiple comparisons
x	☐	A full description of the statistical parameters including central tendency (e.g. means) or other basic estimates (e.g. regression coefficient) AND variation (e.g. standard deviation) or associated estimates of uncertainty (e.g. confidence intervals)
x	☐	For null hypothesis testing, the test statistic (e.g. *F*, *t*, *r*) with confidence intervals, effect sizes, degrees of freedom and *P* value noted<br>*Give P values as exact values whenever suitable.*
x	☐	For Bayesian analysis, information on the choice of priors and Markov chain Monte Carlo settings
x	☐	For hierarchical and complex designs, identification of the appropriate level for tests and full reporting of outcomes
x	☐	Estimates of effect sizes (e.g. Cohen's *d*, Pearson's *r*), indicating how they were calculated

Our web collection on statistics for biologists contains articles on many of the points above.

Software and code

Policy information about availability of computer code

Data collection	Compounds were collected and filtered using RDKit (https://www.rdkit.org/) and Pipeline Pilot 2021 (Biovia, Dassault Systèmes). Further triaging was done in SeeSAR (SeeSAR version 12.1.0) as well as Flare (Cresset Discovery Services). Compound redocking was performed with Glide version 2021-4, build 135, Schrödinger, Inc. or Rosetta version 3.13 (Leaver-Fay et al, "ROSETTA3: an object-oriented software suite for the simulation and design of macromolecules" Methods Enzymol. 487:545-74 (2011)). Torsions were assessed with the SMARTScompare torsion quality tool (Penner, P. et al. The Torsion Library: Semiautomated Improvement of Torsion Rules with SMARTScompare. J. Chem. Inf. Model. 62, 1644–1653 (2022)). Free energy perturbations were performed with FEP+ (Schrödinger version 2021-4) and the ABFE by Biggin and collaborators (Alibay, I., Magarkar, A., Seeliger, D. & Biggin, P. C. Evaluating the use of absolute binding free energy in the fragment optimisation process. Commun. Chem. 5, 105 (2022)). X-ray crystallographic structures were refined with autoPROC (Vonrhein, C. et al. Data processing and analysis with the autoPROC toolbox. Acta Crystallogr. Sect. D: Biol. Crystallogr. 67, 293–302 (2011)), STARANISO (Tickle, I. STARANISO: use of a WebGL-based 3D interactive graphical display to represent and visualise data quality metrics for anisotropic macromolecular diffraction data. Acta Crystallogr. Sect. A Found. Adv. 75, e162–e162 (2019)), Coot (Emsley, P. & Cowtan, K. Coot: model-building tools for molecular graphics. Acta Crystallogr. Sect. D: Biol. Crystallogr. 60, 2126–2132 (2004)) and autoBUSTER (Global Phasing Ltd., Cambridge, United Kingdom, 2017).
Data analysis	(See Data collection)

For manuscripts utilizing custom algorithms or software that are central to the research but not yet described in published literature, software must be made available to editors and reviewers. We strongly encourage code deposition in a community repository (e.g. GitHub). See the Nature Portfolio guidelines for submitting code & software for further information.

Data

Policy information about availability of data

All manuscripts must include a data availability statement. This statement should provide the following information, where applicable:

- Accession codes, unique identifiers, or web links for publicly available datasets
- A description of any restrictions on data availability
- For clinical datasets or third party data, please ensure that the statement adheres to our policy

All player-design compounds, with coordinates corresponding to their highest-scoring poses have been deposited in the Zenodo database under the DOI 10.5281/zenodo.14902201 [https://doi.org/10.5281/zenodo.14902201]. The X-ray crystallography data for 1 in complex with VHL generated in this study has been deposited in the wwPDB database under accension code 8P0F [http://doi.org/10.2210/pdb8P0F/pdb] The previously published starting structure of VHL is availible in the wwPDB under accession code 5NVX [http://doi.org/10.2210/pdb5NVX/pdb].

Research involving human participants, their data, or biological material

Policy information about studies with human participants or human data. See also policy information about sex, gender (identity/presentation), and sexual orientation and race, ethnicity and racism.

Reporting on sex and gender	Information on the sex or gender of participants in the research were not collected or used.
Reporting on race, ethnicity, or other socially relevant groupings	Information on race, ethnicity or other socially relevant grouping of participants in the research were not collected or used.
Population characteristics	Additional human subject population characteristics of participants in the research were not collected or used. Participation was open and free to the public, and we did not control for participant demographics.
Recruitment	Participation was open and free to the public via signup at the website https://fold.it.
Ethics oversight	Participation in the Foldit project is overseen by the University of Washington, under STUDY00001238 “Scientific Discovery Games”.

Note that full information on the approval of the study protocol must also be provided in the manuscript.

Field-specific reporting

Please select the one below that is the best fit for your research. If you are not sure, read the appropriate sections before making your selection.

☒ Life sciences ☐ Behavioural & social sciences ☐ Ecological, evolutionary & environmental sciences

For a reference copy of the document with all sections, see nature.com/documents/nr-reporting-summary-flat.pdf

Life sciences study design

All studies must disclose on these points even when the disclosure is negative.

Sample size	Number of puzzles run was determined by the timeframe available for data collection. Number of compounds generated was determined by participant effort, and all compounds generated were used for subsequent analysis. The number of compounds selected for synthesis was limited by the budget allocated for synthesis.
Data exclusions	No data was excluded. Compound selections were filtered by the criteria listed in methods section.
Replication	Due to the nature of Foldit puzzles, independently replicating compound generation is not possible. Experimental determination of of activity was performed in technical replicates, according to standard practices.
Randomization	There was no randomized sample allocation in this work. All tested designs received identical treatment.
Blinding	Blinding was not relevant to this work, as all tested designs received identical treatment. Selection of compounds for testing was not blinded, as subjective estimation of compound quality was part of the selection criteria

Reporting for specific materials, systems and methods

We require information from authors about some types of materials, experimental systems and methods used in many studies. Here, indicate whether each material, system or method listed is relevant to your study. If you are not sure if a list item applies to your research, read the appropriate section before selecting a response.

Materials & experimental systems

n/a	Involved in the study
☒	☐ Antibodies
☒	☐ Eukaryotic cell lines
☒	☐ Palaeontology and archaeology
☒	☐ Animals and other organisms
☒	☐ Clinical data
☒	☐ Dual use research of concern
☒	☐ Plants

Methods

n/a	Involved in the study
☒	☐ ChIP-seq
☒	☐ Flow cytometry
☒	☐ MRI-based neuroimaging

Plants

Seed stocks

Report on the source of all seed stocks or other plant material used. If applicable, state the seed stock centre and catalogue number. If plant specimens were collected from the field, describe the collection location, date and sampling procedures.

Novel plant genotypes

Describe the methods by which all novel plant genotypes were produced. This includes those generated by transgenic approaches, gene editing, chemical/radiation-based mutagenesis and hybridization. For transgenic lines, describe the transformation method, the number of independent lines analyzed and the generation upon which experiments were performed. For gene-edited lines, describe the editor used, the endogenous sequence targeted for editing, the targeting guide RNA sequence (if applicable) and how the editor was applied.

Authentication

Describe any authentication procedures for each seed stock used or novel genotype generated. Describe any experiments used to assess the effect of a mutation and, where applicable, how potential secondary effects (e.g. second site T-DNA insertions, mosiacism, off-target gene editing) were examined.
